# Mechanism for Aromatic Nitration Catalyzed by Cytochrome P450 Enzymes from Rufomycin Biosynthesis

**DOI:** 10.1021/jacs.6c05584

**Published:** 2026-07-29

**Authors:** Alicia M. Kirk, Jemma Gullick, Yongwei Zhao, Ralf B. Schittenhelm, Ferran Feixas, Hiroya Tomita, Yohei Katsuyama, Yasuo Ohnishi, James J. De Voss, Max J. Cryle, Marc Garcia-Borràs

**Affiliations:** † School of Chemistry and Molecular Biosciences, The University of Queensland, Brisbane, QLD 4067, Australia; ‡ 226478 Scuola Normale Superiore, Pisa, PI 56126, Italy; § Department of Biochemistry and Molecular Biology, The Monash Biomedicine Discovery Institute, Monash University, Clayton, VIC 3800, Australia; ∥ ARC Centre of Excellence for Innovations in Peptide and Protein Science, Clayton, VIC 3800, Australia; ⊥ Graduate School of Agricultural and Life Sciences, The University of Tokyo, Bunkyo-ku, Tokyo 113-8657, Japan; # Proteomics and Metabolomics Platform, 2541 Monash University, Clayton, VIC 3800, Australia; ∇ Institut de Química Computacional i Catàlisi (IQCC) and Departament de Química, 16738 Universitat de Girona, Campus Montilivi, Girona 17003, Spain; ○ ICREA, Passeig Lluís Companys, 23, 08010 Barcelona, Spain; ◆ Collaborative Research Institute for Innovative Microbiology, The University of Tokyo, Bunkyo-ku, Tokyo 113-8657, Japan

## Abstract

During the biosynthesis of the peptide antibiotic rufomycin, the cytochrome P450 (P450) enzyme RufO catalyzes the aromatic nitration of a tyrosine residue in a ribosomal pentapeptide that serves as a precursor for nonribosomal peptide synthesis. To understand the mechanism of this unusual P450-mediated reaction, a series of pentapeptides were tested as substrates alongside molecular dynamics simulations and quantum mechanics/molecular mechanics (QM/MM, ONIOM approach) calculations. A new substrate-bound crystal structure of the homologue NsRufO was also obtained. These experiments revealed the intimate and necessary involvement of a histidine residue within the pentapeptide substrate of RufO in supporting effective nitration, with the protonation state of the intermediate compound II proposed to influence the production of nitrated tyrosine over unwanted nitrate formation. These findings provide key insights into the mechanism of P450-mediated nitration that explain how sequence differences in RufO and its homologues, along with the structure of the pentapeptide substrate, facilitate aromatic nitration of tyrosine by RufO.

## Graphical abstract

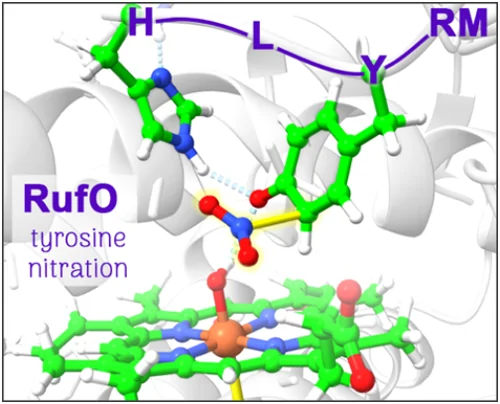

## Introduction

The rufomycins are peptide natural products of nonribosomal origin that exhibit potent antibiotic activity toward *Mycobacterium tuberculosis*. Among key tailoring enzymes found within the rufomycin biosynthetic gene cluster (BGC), RufO is a cytochrome P450 (P450) that has been implicated in the nitration of a Tyr residue within rufomycin, although the exact nature of this transformation has proved enigmatic until recently.^1^ The native substrate of RufO has now been identified as the ribosomally synthesized pentapeptide MRYLH, encoded by a *bytA* homologue found upstream of the *rufNO* operon in the rufomycin BGC.^2^ RufO and its homologues catalyze nitration of the MRYLH tyrosine residue at the 3-position, requiring nitric oxide (NO) as a cosubstrate; the latter is provided by the nitric oxide synthase (NOS) RufN (Figure 1).^1,3^ Subsequent digestion of the modified peptide by RufB releases 3-NO_2_-Tyr as a noncanonical precursor for incorporation into the nonribosomal peptide synthase (NRPS)-mediated biosynthesis pathway of rufomycin. Nitration of amino acid precursors had previously been observed only for NRPS pathways^4^ and not as part of a ribosomally synthesized and post-translationally modified peptide (RiPP), particularly one that acts as a source of a modified amino acid for an NRPS. The biosynthesis of rufomycin provides an intriguing example of the integration of both RiPP and NRPS pathways to generate a single natural product, in which the RiPP pathway provides a noncanonical amino acid for the NRPS machinery.

**Figure 1:**
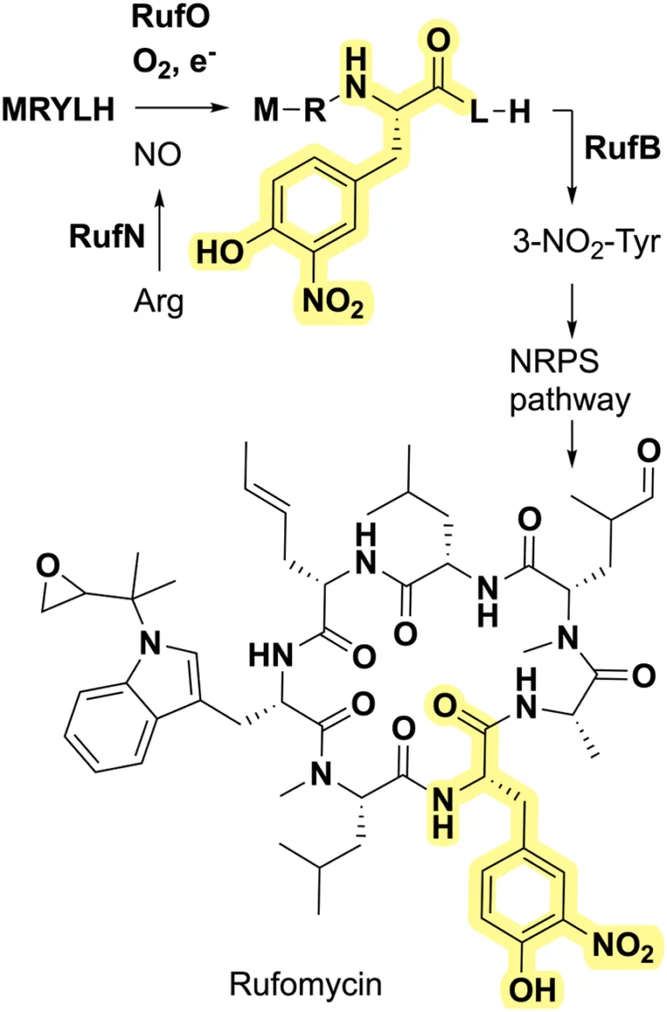
Generation of noncanonical precursor, 3-NO_2_-Tyr, from a RiPP in the biosynthesis of rufomycin involving RufN, RufO, and RufB. Nitrated tyrosine highlighted in yellow.

Within the vast catalogue of ∼1,000,000 sequences, P450s are known to catalyze an incredible array of distinct chemical transformations,^5–7^ with only three other P450s known to catalyze aromatic nitration: TxtE,^4,8^ IlaN,^9^ and Laj2.^10^ These are all also accompanied by a nitric oxide synthase: TxtD, IlaM, and Laj3, respectively. Additionally, a P450_BM3_ variant has been engineered to catalyze nitration, but it uses nitrite rather than NO as a nitrogen source.^11^ Previous investigations of TxtE, other model systems, and most recently on RufO have proposed the diffusion-controlled reaction of NO and ferric-superoxide to form a peroxynitrite intermediate.^12–15^ Unlike the canonical P450 cycle, this proposed mechanism requires only a single electron transferred from a reductase partner (Figure 2). Homolytic cleavage of the peroxynitrite would generate the nitrogen dioxide radical, ^•^NO_2_, and a deprotonated Compound II (dp-Cpd II), with subsequent steps remaining unclear.

**Figure 2:**
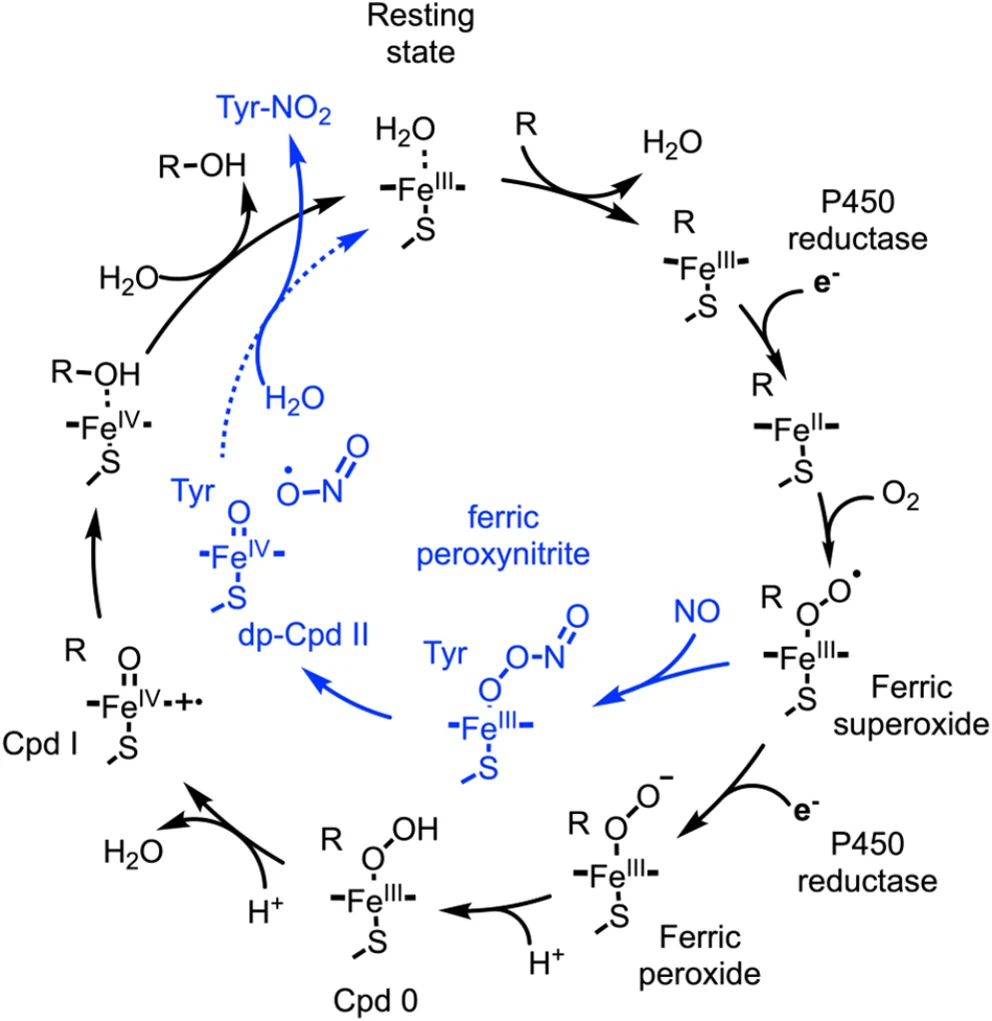
Divergence of the canonical P450 cycle (black) to generate a peroxynitrite intermediate (blue pathway) followed by homolytic O–O cleavage to form ^•^NO_2_. Note that the substrate, R, can refer to numerous aliphatic and aromatic molecules in the hydroxylation pathway (black), while for the nitration pathway (blue), we refer here to a tyrosine-containing substrate.

For tryptophan/indole nitration by either TxtE or a model heme catalyst, the following two mechanisms have been proposed: either ^•^NO_2_ adds directly to the aromatic ring followed by *ipso*-hydrogen abstraction to form the nitrated product, or alternatively in solution, one equivalent of ^•^NO_2_ activates the substrate (through abstraction of the indole N–H hydrogen) and a second equivalent then forms the nitrated product through radical recombination.^12,15^ Typically, accepted mechanisms of phenol and protein tyrosine nitration involve initial oxidation to generate a phenolic or tyrosyl radical, which then couples with ^•^NO_2_.^16^ We propose that initial oxidation of tyrosine is also a key step in RufO-catalyzed nitration.

Crystal structures of RufO (PDB ID: 8SPC,^17^ 8SPP,^18^ 9EBY,^13^ 9DUJ^13^) show a high structural similarity along with 45% sequence identity to the biaryltide cross-linking P450_Blt_ (PDB ID: 8U2M^19^), for which MRYLH is also a substrate, albeit one in which a cross-link is formed between the Tyr-3 and His-5 residues of this peptide.^20–22^ A key difference in the RufO active site is the mutation of the widely conserved I-helix alcohol residue Ser239 to Val. This change is thought to prevent the protonation of the ferric-superoxide/ferric-peroxide required to form Compound I and to potentially alter the redox potential of the substrate Tyr-3 residue.^2,13^ However, the P450_Blt_ S239V mutant cannot catalyze nitration of (nor cross-link) the MRYLH substrate in the presence of NO, suggesting this difference alone does not dictate the change in catalytic activity from cross-linking to nitration.^2^ Nolan and co-workers recently suggested that differences in the hydrogen-bonding network to the substrate His-5 may contribute to enzymatic selectivity by increasing the Tyr-3 to His-5 distance by 0.7 Å in the RufO-bound MRYLH substrate (based on aromatic ring centroids in the crystal structures), which could hinder peptide cross-linking.^13,19^ Complicating this further is data from previous studies that show RufO has the capacity to catalyze cross-linking of the non-native MRYLW substrate in addition to its capacity for catalyzing nitration of the native MRYLH substrate.^2,21,23^ Thus, the underlying mechanisms that allow these two homologous P450s to catalyze different chemistry remain elusive.

Now, through a combination of substrate turnover experiments, molecular dynamics (MD) simulations, and quantum mechanics (QM) calculations, we present results that strongly implicate tyrosyl radical formation as essential in the proposed mechanism of nitration and suggest that differences in the hydrogen-bonding network in RufO may facilitate a key proton-transfer step to avoid nitrate formation. Elucidating these mechanisms advances our understanding of selective nitro group incorporation in natural product biosynthesis and informs future efforts to engineer effective nitration biocatalysts with potential medicinal, pharmaceutical, and agrochemical applications.

## Results and Discussion

### Substrate Turnover

A series of peptides were subjected to *in vitro* nitration assays to further investigate RufO selectivity and gain insights into the mechanism of this P450. In a yet unexplained phenomenon, all RufO enzymes exclusively utilize the MRYLH peptide as the substrate.^2^ The reason for the lack of variation in the natural substrate is not obvious. Therefore, a series of mutants were generated across positions 2–5 of the analogous peptide Nle-RYLH (Nle being preferred to Met to avoid facile sulfoxidation that has a deleterious effect on binding to the P450) (Figure S1).^21^ Across all of the experiments conducted for this study, a dramatic reduction in the level of nitration by this enzyme was observed relative to previous studies with the substrate Nle-RYLH.^2^ This reduction in activity appeared to result from degradation of the NO source (diethylamine NONOate) despite best efforts to preserve it; similar activity levels have also recently been reported by Nolan et al.^13^ Further attempts to rectify this using new enzyme preparations also afforded the same results, and hence, the results are reported here relative to the nitration observed for the control peptide (Nle-RYLH) using the same batches of P450 and NO source. The enzymatic reaction of a RufO homologue, called NsRufO, with Nle-RYLH was also compared, displaying 46% conversion compared to that of RufO.

All changes to the substrate reduced activity relative to the control (Figure 3A and Figures S73 and S74). Truncation of the position 2 side chain to an alanine residue (Nle-**A**YLH) reduced activity to 17% of that observed with the native peptide. Similarly, mutation of position 4 from Leu-4 to the aromatic Tyr (Nle-RY**Y**H) reduced activity to 23% while maintaining selectivity for nitration of Tyr-3 (Figure 3E,3F). Mutation of the crucial Tyr-3 to Phe (Nle-R**F**LH) or O-Me-tyrosine (Nle-R-**(OMe)­Tyr**-LH) prevented nitration altogether, while nitration of the peptide containing *meta*-tyrosine (3-hydroxyphenylalanine) in the central position (Nle-R-***m*Tyr**-LH) was reduced to 48%. It is important to note that the lack of nitration of Nle-R**F**LH was not related to a loss of binding efficiency for this substrate compared to Nle-RYLH (Figure 3B,3C). It should be noted that a higher concentration of RufO was used in the binding assays compared to previous studies,^2^ as we assumed that the non-native substrates would display weaker affinities toward RufO; thus a higher apparent K_d_ was calculated for RufO with Nle-RYLH. In position 5, glutamic acid was tolerated to a small extent (Nle-RYL**E**, 13%), but not thiazolalanine (Nle-RYL-**Thz**, 0%) or glutamine (Nle-RYL**Q**, 0%), with the Nle-RYL-**Thz** peptide also exhibiting a 10-fold loss of binding affinity (Figure 3D). The tolerance of 5-Glu but not 5-Gln is curious, as Gln is typically seen as a closer replacement for His than Glu. Finally, and as introduced above, limited nitration was observed for tryptophan in position 5 (Nle-RYL**W**); instead, cross-linking occurred preferentially with this substrate. While Nle-RYLH displayed the tightest binding to RufO, as indicated by the lowest *K*_d_ value, all three of the peptides with alternative residues in position 3 (Nle-R**F**LH, Nle-R-**(OMe)­Tyr**-LH, and Nle-R-**mTyr**-LH) displayed increased binding efficiencies, whereas peptides with modifications in positions 2, 4, and 5 had decreased binding efficiencies (Table S1).

**Figure 3:**
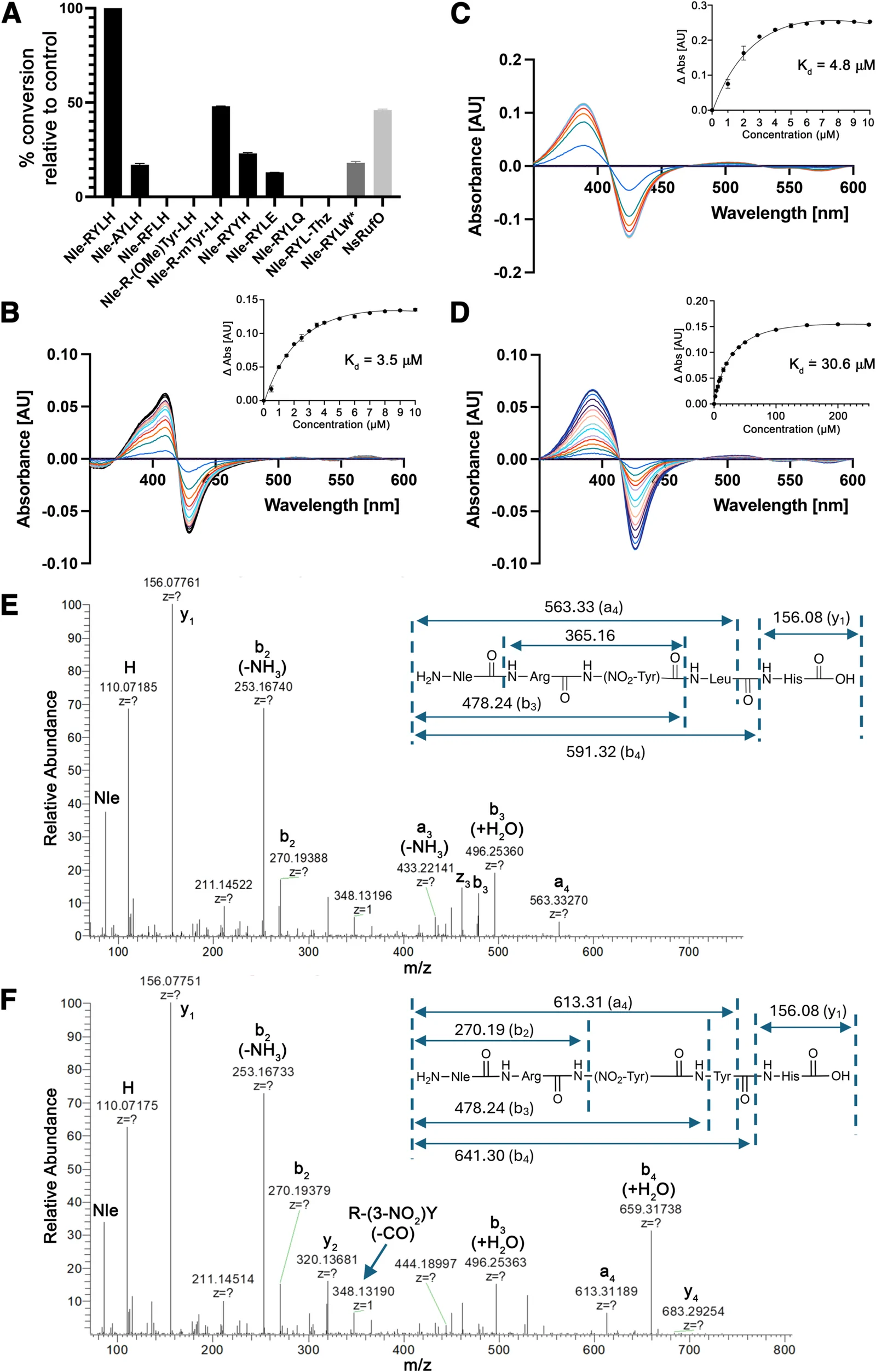
Exploration of *in vitro* activity of RufO with analogous substrates. (A) % nitration relative to control levels for the peptides tested in this study, except for Nle-RYLW* which shows % conversion to cross-linked product (relative to starting material) determined by area under the curve of TIC corresponding to the peptide *m*/*z* values, light gray bar shows NsRufO nitration of Nle-RYLH and error bars represent SD, *n* = 3, (B) binding data of RufO to Nle-RYLH, (C) binding data of RufO to Nle-R**F**LH, (D) binding data of RufO to Nle-RYL-**Thz**, (E) annotated MS^2^ spectrum of the nitrated product from nitration assay of RufO with Nle-RYLH substrate, and (F) annotated MS^2^ spectrum of the nitrated product from nitration assay of RufO with Nle-RY**Y**H.

The results obtained from the position 3 mutants of the Nle-RYLH substrate implicate the phenolic hydrogen as an important contributor to the mechanism of nitration. Somewhat counterintuitively, the histidine in position 5 also seems to be intimately involved in facilitating efficient nitration, as loss of this residue prevents or greatly decreases nitration, and replacement with a reactive coupling partner like tryptophan results in cross-linking. To assess whether altered binding or intrinsic reactivity could account for impaired catalysis across the mutant series, structure prediction, MD simulations, and reaction pathway QM calculations were employed.

### Predicted Binding Conformation of MRXXX to RufO

The Alphafold3 (AF3) server was used to predict the binding conformations of the pentapeptide substrates MR**Y**L**H** (**1**), MRYL**W** (**2**), MRYL**E** (**3**), MRYL**Q** (**4**), MR**F**LH (**5**), and MRY**Y**H (**6**) to RufO with the heme cofactor present.^24^ A typical P450 trigonal-prism fold was generated with high confidence with the substrate bound (Figures S83–S85). During the preparation of this manuscript, the structure of 1-bound RufO was published (PDB ID: 9EBY).^13^ The RMSD between the top AF3 predicted structure and 9EBY was 0.60 Å across all 390 pairs of residue backbones (including bound **1**) or 1.04 Å with the inclusion of all side chain atoms.

For the native substrate **1**, the predicted binding conformation was in general agreement with that observed in the crystal structure (PDB ID: 9EBY) and similar to the conformation found in the substrate **1**-bound crystal structure of P450_Blt_ (PDB ID: 8U2M) (Figure 4). Specifically, the substrate Tyr-3 and His-5 side chains were positioned over the heme, with the latter hydrogen bonded to the I-helix H235 residue along with hydrogen bonding between the substrate backbone and R231, R188, and Q84. The predicted E239 side chain rotamer aligned more closely to that seen in the P450_Blt_ crystal structure as opposed to the RufO crystal structures. The predicted H235 side chain position deviated slightly from that observed in the substrate-bound RufO crystal structure; however, the overall conformational change that occurs between the substrate-free and substrate-bound enzyme conformations was captured, including the shifts in R188 and H235.

**Figure 4:**
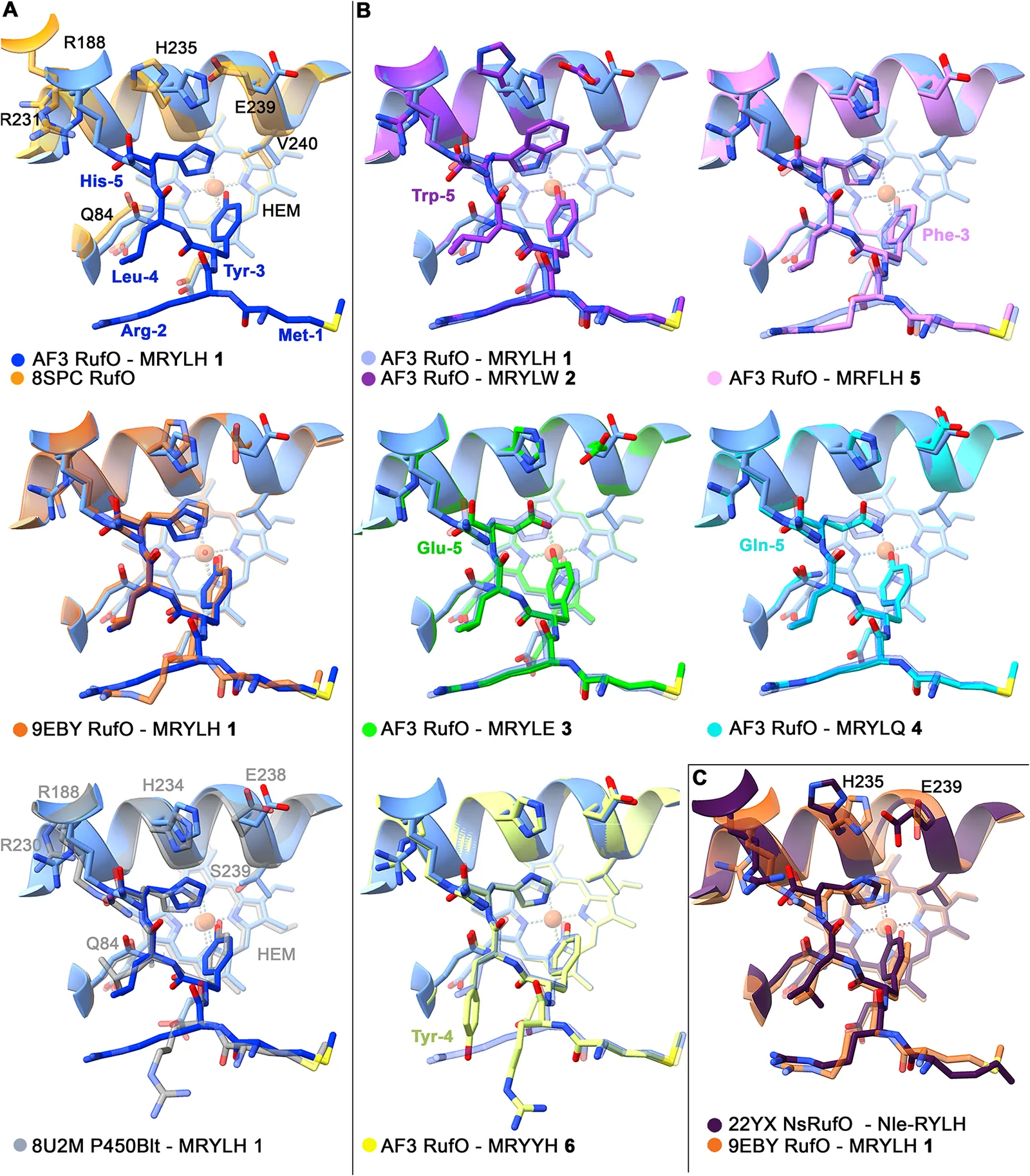
Alphfold3 (AF3) predicted structures of the substrate-bound RufO. (A) Comparison with crystal structures via overlay of AF3 **1**-bound RufO (blue) with substrate-free crystal structure (8SPC)^17^ (top); substrate-bound RufO (9EBY)^13^ (middle); and substrate-bound P450_Blt_ (8U2M)^19^ (bottom). (B) AF3 **1**-bound RufO (blue) via overlay with predicted binding structures of **2** (purple); **3** (green); **6** (yellow); **5** (pink); and **4** (cyan). (C) Overlay of substrate-bound RufO (9EBY,^13^ orange) and substrate-bound homologue NsRufO (22YX, purple). Substrate and key residues are labeled. No waters are shown.

For all of the other substrates, the predicted binding conformation was largely unchanged with respect to that predicted for **1** (Figure 4). The side chains of substrate positions 3 and 5 remained bound over the heme. To accommodate the larger substrate **2**, the H235 side chain rotated to adopt a conformation analogous to that found in the substrate-free RufO crystal structure, accompanied by rotation of the E329 side chain. For **6**, selectivity for nitration of Tyr-3 can be rationalized based on Tyr-4 facing away from the heme when bound. For **3** and **4**, H235 was able to hydrogen bond to Glu-5 and Gln-5, respectively, which both, in turn, could hydrogen bond to Tyr-3; no structural reasons for the reduction in nitration of these two substrates were immediately apparent.

In parallel with the computational mechanistic investigations that follow, a substrate-bound crystal structure of a RufO homologue, referred to as NsRufO, was pursued to obtain further experimental insights into substrate positioning. This crystal structure will be discussed further below; however, in brief, the Nle-RYLH substrate was found to be bound similarly to the conformation observed for **1**bound RufO. There were, however, variations in the conformations of the H235 and E239 side chains (Figure 4C).

### Quantum Mechanics Calculated Potential Reaction Mechanisms

Using a truncated model system and density functional theory (DFT), two potential nitration mechanisms were investigated: (A) direct radical addition of ^•^NO_2_ to tyrosine versus (B) radical recombination of ^•^NO_2_ with a tyrosyl radical (Figure 5). Both begin with homolytic cleavage of the *s-cis-*peroxynitrite intermediate (**TS1**) to form the radical ^•^NO_2_. Cleavage of the *s-trans*-peroxynitrite was calculated to occur via a pathway with a higher energy barrier (Figure S86). Here, unlike in the models of TxtE-catalyzed nitration of l-tryptophan,^8,14,15^ direct addition of ^•^NO_2_ to the aromatic carbon (**TS2**) (of phenol as opposed to tryptophan) in path A, with subsequent abstraction of the *ipso*-hydrogen (**TS3**), was calculated to be the highest energy pathway. Subsequent abstraction of the phenolic hydrogen (**TS4**) to form the tautomeric product could be an alternative. However, path B (blue) was found to be lower in energy, involving phenolic hydrogen abstraction by deprotonated Compound II (**TS5**) followed by energetically barrierless radical recombination in the gas phase (refer to Figures S87 and S88 for the calculated PES). The key involvement of the phenolic group in the mechanism of Path B agrees with the importance of the phenolic OH group demonstrated experimentally in the alternative substrates explored above, where a lack of reactivity resulted from its removal or its substitution to eliminate the abstractable hydrogen.

**Figure 5:**
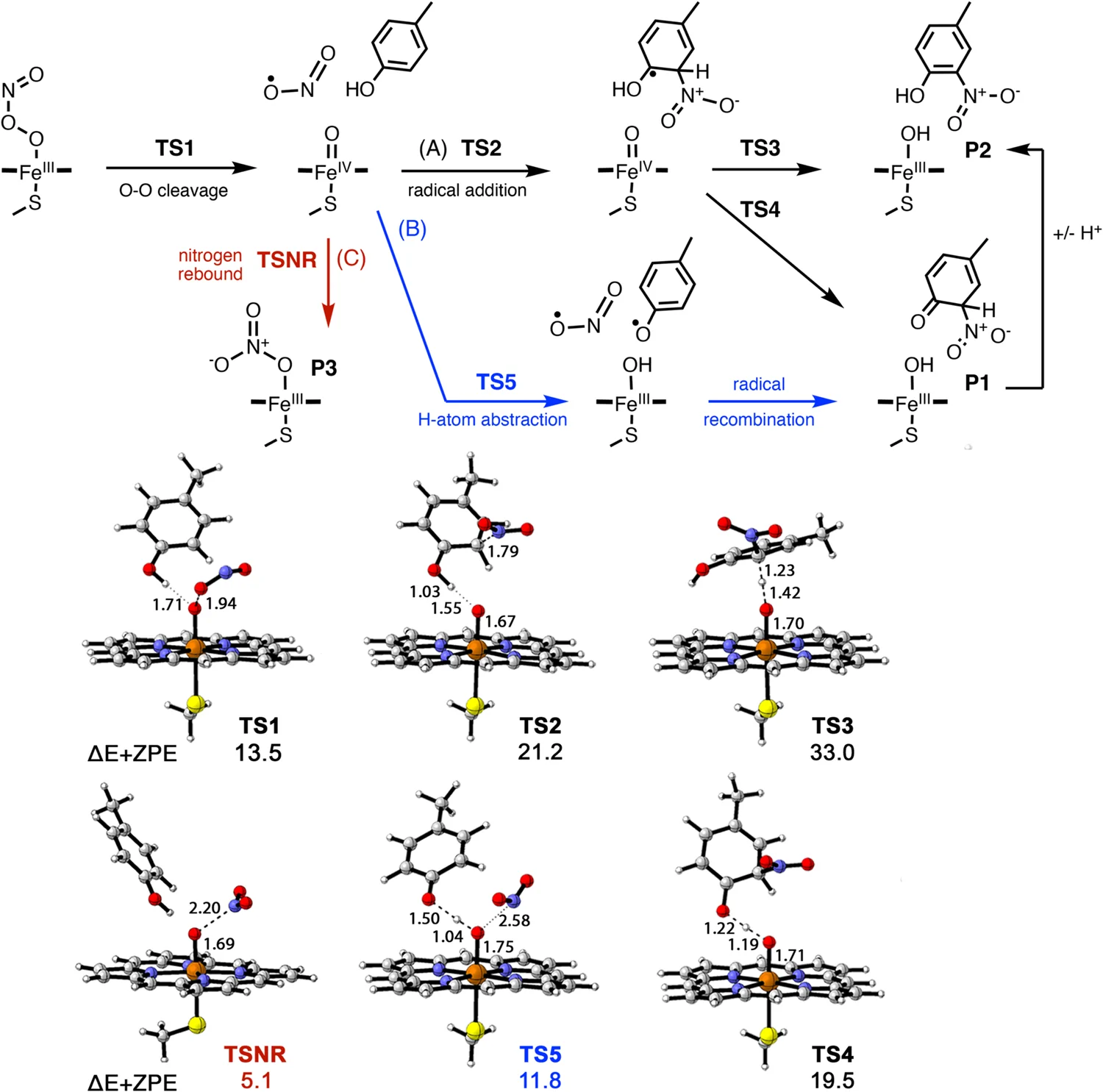
Potential mechanisms of nitration following formation of ^•^NO_2_ from homolytic cleavage of the ferric peroxynitrite. Path A (black): radical addition of ^•^NO_2_ followed by hydrogen atom abstraction. Path B (blue): phenolic hydrogen atom abstraction followed by radical recombination. Path C (red): N–O radical rebound to form nitrate as a byproduct. Potential energies calculated using a truncated model at the B3LYP-D3BJ/BS2//B3LYP/BS1 level of theory, S = 1/2, with zero-point-energy corrections (E+ZPE) are given in kcal mol^–1^. Key Mulliken spin densities and geometries for these structures are listed in Tables S2 and S3.

Furthermore, abstraction of a phenolic hydrogen would seem logical if RufO has diverged from the cross-linking P450_Blt_ for which a diradical mechanism involving abstraction of the phenolic hydrogen is proposed.^19^ Abstraction of phenolic hydrogen atoms by both Cpd I and Cpd II in the cross-linking mechanism of the P450 OxyB was also calculated to have extremely low energy barriers.^25^ Moreover, for a P450 TxtE model complex, nitration of tryptophan was also proposed to proceed through a diradical mechanism where a cage-escaped ^•^NO_2_ first abstracts the NH hydrogen of the indole ring.^12^ By analogy, we might postulate that for substrate **2** (MRYL**W**) with RufO, where cross-linking is mainly observed, ^•^NO_2_ could likewise abstract the N–**H** hydrogen of the tryptophan indole ring. If Cpd II abstracts the phenolic hydrogen, then this facilitates a diradical coupling mechanism. Abstraction of an indole N–**H** was calculated to be 7.0 kcal mol^–1^ lower in energy than abstraction of an imidazole hydrogen (Figure S89), which would be congruent with no cross-linking being observed for the native MRYL**H**.

Finally, as noted with other model complexes,^26^ heme–nitrate complexes are an alternative product resulting from those ^•^NO_2_ radicals that do not escape from the caged radical intermediate. Formation of nitrate as a byproduct through ^•^NO_2_ rebound (**TSNR**) was found to be low in energy and may be a competitive pathway subject to diffusion of the ^•^NO_2_ radical (Figure 5, red). Consistent with this possibility, nitrogen dioxygenases (NODs) convert nitric oxide to nitrate through a mechanism proposed to involve the formation of a heme–peroxynitrite intermediate.^26,27^ In NODs, homolytic O–O cleavage forms ^•^NO_2_, which then recombines with Fe­(IV)-oxo. Furthermore, nitric oxide synthases (NOSs) are reported to form nitrate upon reaction with peroxynitrite, again via isomerization of heme–peroxynitrite through ^•^NO_2_ formation and radical recombination.^28^ Therefore, nitrate formation seems a plausible competing pathway in RufO-catalyzed nitration given the same initial O–O cleavage is proposed, unless additional features of RufO suppress it.

Previous studies have demonstrated that H-bonding from a proximal histidine stabilizes tyrosine radicals.^29^ Moreover, small models of P450_Blt_ cross-linking indicated that histidine protonation states and H-bonding interactions altered the relative energetics of different coupling pathways.^19^ Whether involvement of the substrate His-5 residue and the larger H-bonding network influences the partition between nitration and nitrate formation is further explored using MD simulations and QM/MM modeling (see below). Refer to the SI (Figure S90) for a brief discussion of a comparative small-model study including imidazole.

### Molecular Dynamics Simulations

MD simulations of substrate-bound RufO in the superoxide and peroxynitrite intermediate forms were performed in triplicate (500 ns each independent trajectory), starting from the AF3 predicted bound structure with manual addition of dioxygen or peroxynitrite. The substrate remained bound throughout the entire duration of the simulations, indicating a stable binding conformation (see RMSD measurements in Figure S91). For substrate-bound P450_Blt_, only the superoxide intermediate was simulated. Key hydrogen-bonding patterns were analyzed as summarized in Tables 1 and S4.

**Table 1:**
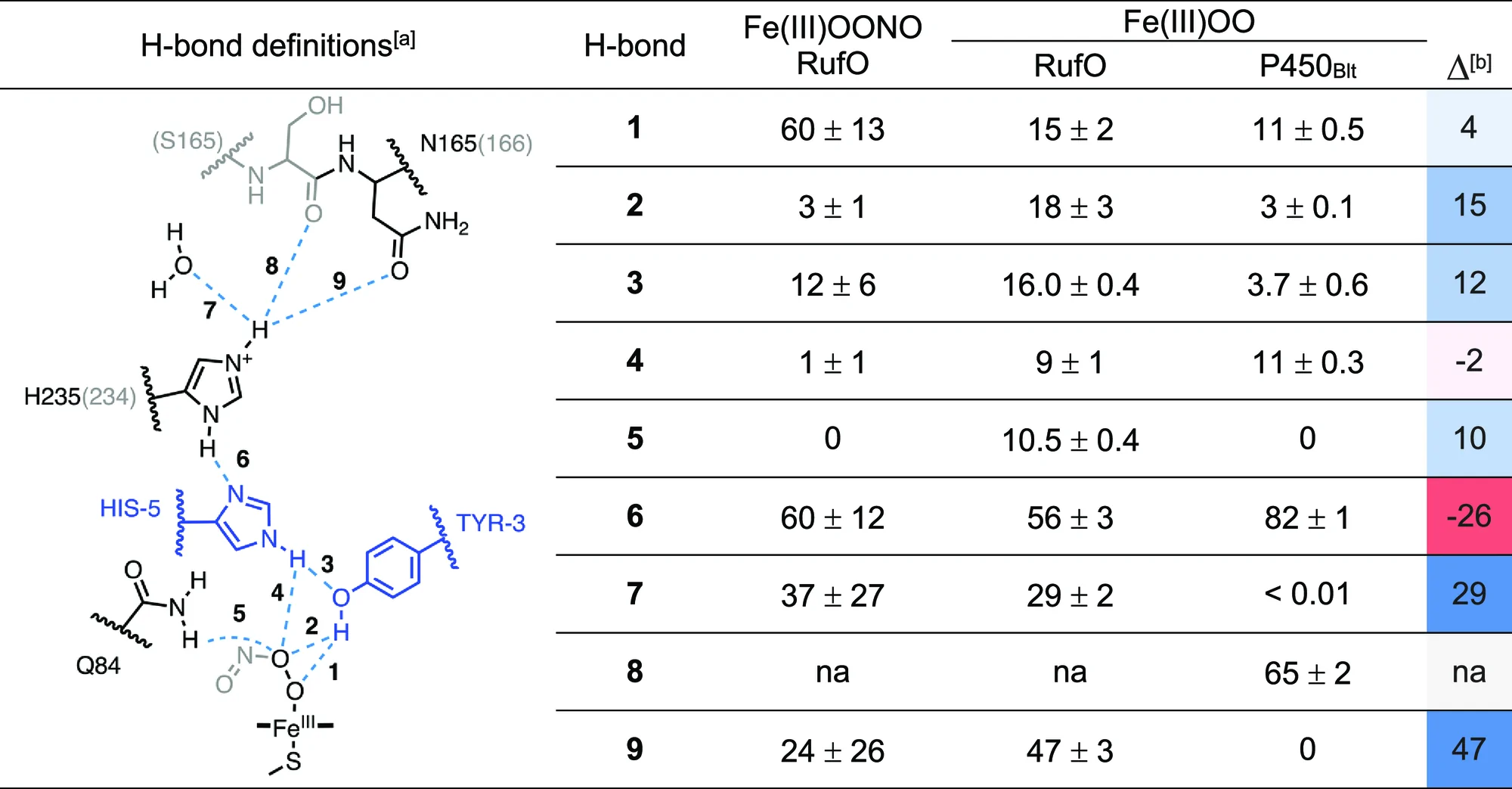
Percentage of MD Snapshots in Which Specified Hydrogen Bonds Are Present Based on an Acceptor–Donor Distance ≤ 3.0 Å and Donor-H-Acceptor angle ≥ 135°^c^

a Residue numbering in brackets refers to P450_Blt_.

b Δ represents the difference between RufO and P450_Blt_ for the superoxide result.

c Mean % ± standard deviation for triplicate runs reported.

H-bonding was observed from Tyr-3 to Fe–O more often for the peroxynitrite intermediate (Table 1, entry **1**): 60% (peroxynitrite) versus 15% and 11% (superoxides). A higher prevalence of an H-bond from His-5 to Tyr-3 was observed in RufO compared to that seen with P450_Blt_:12–16% versus 4% (Table 1, entry **3**). This appears to result from the slight change in the binding position of His-5 between the two enzymes. The transience of this H-bond is consistent with the His-5 to Tyr-3 contact observed in the substrate-bound RufO crystal structure (PDB ID: 9EBY), which, after hydrogen placement, should be regarded as a borderline preorganized contact rather than as evidence for a persistent hydrogen bond under the stricter quantitative criterion used for MD analysis. Furthermore, a reduction in H-bonding from H235(234) to His-5 in RufO, 56–60% versus 82% (Table 1, entry **6**), implies a greater flexibility in the positioning of H235 and His-5 in the modified H-bonding network for RufO. This is accompanied by greater solvent accessibility of H235:29–37% versus 0% (Table 1, entry **7**).

Representative snapshots of the most populated conformational cluster from accumulated MD simulations are shown in Figure 6. The larger peroxynitrite ligand, compared to the oxo ligand, did not appear to significantly alter the binding position of the substrate. Nor did the absence of an H-bond from S239 (V240) change the mean distance of the 3-Tyr hydroxyl to the heme Fe in the superoxo intermediate MD simulations: RufO 4.7 ± 0.4 Å versus P450 Blt 4.4 ± 0.3 Å. The 3-Tyr phenolic hydrogen remains well positioned for abstraction during the catalytic nitration mechanism in RufO.

**Figure 6:**
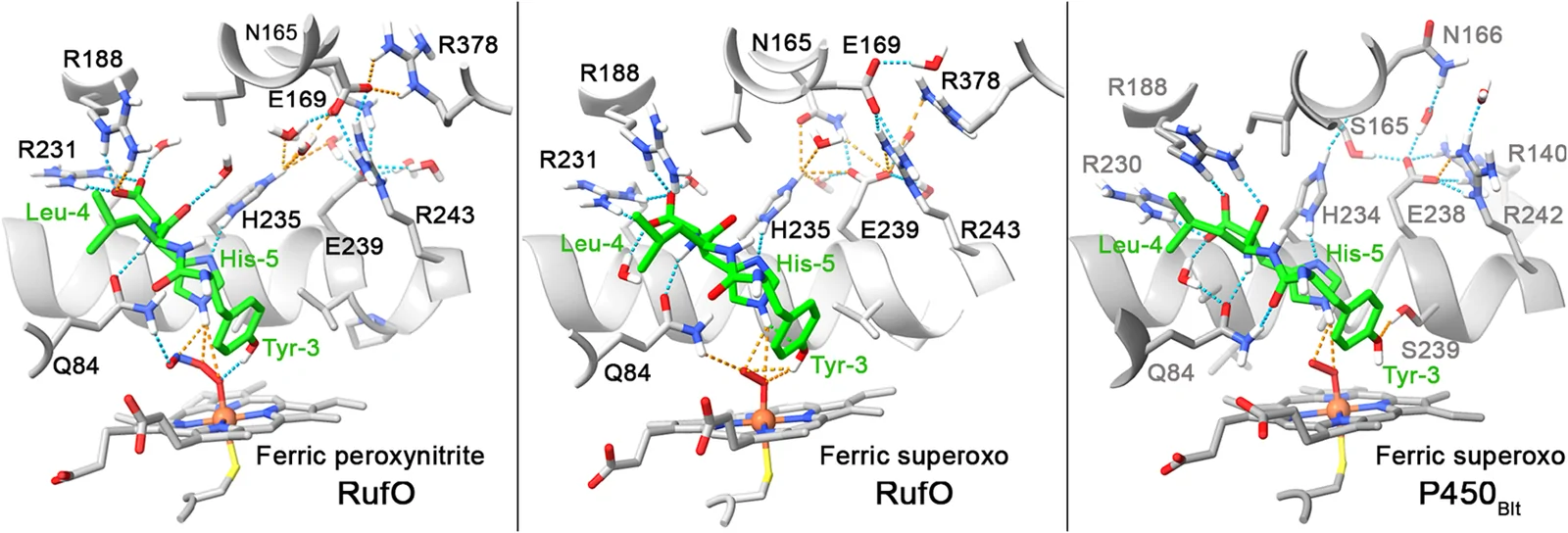
Representative snapshots of substrate (green) binding conformation for residues near the heme during MD simulations of RufO with the ferric peroxynitrite (left) and ferric superoxo (middle) intermediates and P450_Blt_ (right) with the ferric superoxo intermediate. Substrate Met-1 and Arg-2 are not shown for clarity. Key residues are labeled. Blue dotted lines indicate hydrogen bonds based on strict criteria (in ChimeraX); orange dotted lines indicate H-bonds with relaxed criteria (0.4 Å distance tolerance, 20° angle tolerance). ChimeraX contacts are used for structural visualization only and not for H-bond population analysis.

### Quantum Mechanics/Molecular Mechanics Calculations

To further explore the role of the protein environment on the RufO catalytic nitration mechanism, the energetic landscape of proposed mechanisms was calculated using QM/MM calculations based on the ONIOM^30,31^ approach as implemented in Gaussian 16 Software^32^ (Figure 7). The initial conformation was obtained by selecting a representative snapshot from the MD simulations based on clustering of the substrate and active site residues (Figure S92). The quantum mechanics (QM) model region was expanded compared to the small truncated DFT model to include the substrate His-5 and I-helix residue H235, while the rest of the protein and solvent were treated at the molecular mechanics (MM) level.

**Figure 7:**
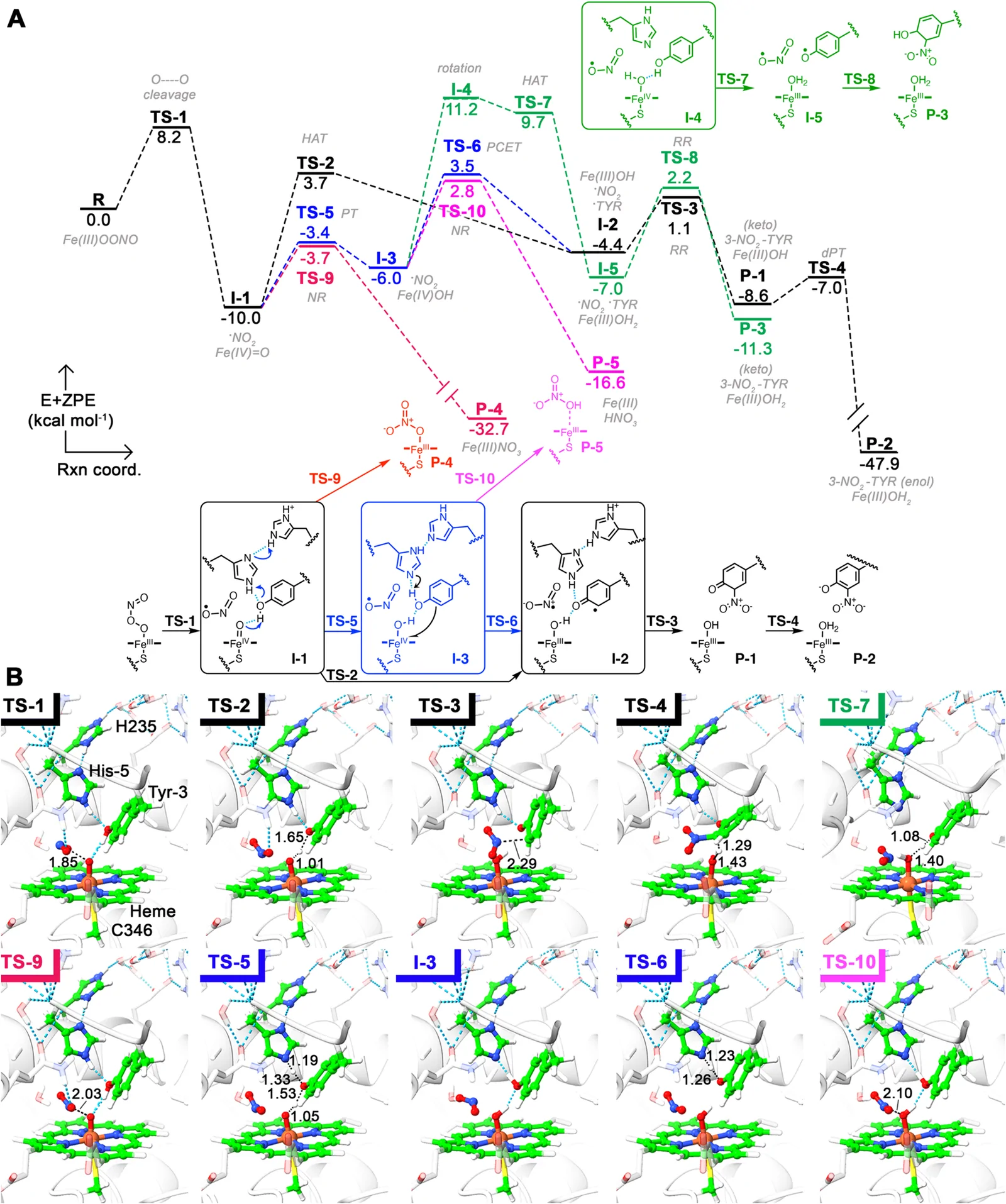
(A) Potential energy diagram of proposed mechanisms of RufO-catalyzed nitration of MRYLH calculated at the ONIOM­(B3LYP/BS4//B3LYP/BS3:AMBER­(FF14SB)) level of theory. Energies include zero-point-energy corrections (E+ZPE, kcal mol^–1^). Hydrogen atom transfer (HAT), radical recombination (RR), proton transfer (PT), proton-coupled electron transfer (PCET), deprotonation (dPT), and ^•^NO_2_ rebound (NR). (B) Geometries of key stationary points. High layer shown in green; MM layer in gray; H-bonds in blue dotted lines; TS bonds in black dotted lines. Key bond distances labeled in Å. Refer to Figures S93–S94 for geometries of all stationary points. Key Mulliken spin densities are listed in Table S6.

First, the energy pathway for a mechanism analogous to the small truncated DFT model reported in Figure 5 was calculated (Figure 7 black and Figure S93). A relatively small 8.2 kcal mol^–1^ barrier for homolytic O–O cleavage of the heme–peroxynitrite initiates the reaction (**TS-1**) with the resulting ^•^NO_2_ radical and dp-Cpd II (**I-1**) stabilized relative to the reactant complex. The substrate Tyr-3 is well-positioned in the active site to facilitate hydrogen atom abstraction (**TS-2**, DE+ZPE^‡^ = 13.7 kcal mol^–1^), forming a tyrosyl radical and Fe­(III)­OH (**I-2**). Radical recombination of ^•^NO_2_ and the tyrosyl radical (**TS-3**, DE+ZPE^‡^ = 5.5 kcal mol^–1^) forms a nitrated keto-product (**P-1**) before deprotonation facilitated by the Fe­(III)­OH (**TS-4**, DE+ZPE^‡^ = 1.6 kcal mol^–1^) and rearomatizes the product (**P-2**), which provides the major thermodynamic driving force of the reaction. Here, **P-2** corresponds to the phenolate form of the product, consistent with the reduction in p*K*_a_ upon tyrosine nitration.^33,34^ This mechanism seems energetically feasible; however, deprotonated Cpd II is reported to be very basic (Cpd II p*K*_a_ > 10).^35–37^ Therefore, alternative mechanisms were considered in which dp-Cpd II becomes protonated, considering Tyr-3 as a potential proton donor.

The transition state for an isolated proton transfer from Tyr-3 to Fe­(IV)=O (i.e., to form a phenolate and Fe­(IV)­OH) could not be located. Instead, an energetically favored proton relay transition state was found, where a proton is shuttled from protonated H235 through His-5 and Tyr-3 to Cpd II (**TS-5**, DE+ZPE = 6.6 kcal mol^–1^) (Figure 7 blue and Figure S94). In the resulting intermediate (**I-3**), Tyr-3 remains in the neutral phenol form; the relayed proton resides on Cpd II in the Fe­(IV)­OH species; both H235 and His-5 now take neutral forms. Therefore, the 4 kcal mol^–1^ energy difference from **I-1** to **I-3** reflects both the proton relay and active-site hydrogen-bond reorganization rather than an isolated Tyr-3 deprotonation/protonation equilibrium.

From **I-3**, further reorganization of the hydrogen-bonding to facilitate hydrogen abstraction from Tyr-3 forms a high-energy intermediate (**I-4**, DE+ZPE^‡^ = 17.2 kcal mol^–1^) that prevents this pathway (Figure 7 green). Alternatively, from **I-3**, a multisite proton-coupled electron transfer (PCET, **TS-6**, DE+ZPE^‡^ = 9.5 kcal mol^–1^, Figure 7 blue) can generate the key tyrosyl radical and Fe­(III)­OH (i.e., **I-2** in the initially described black pathway) and proceed as before through a diradical recombination. This alternative mechanism intimately involves His-5, which acts first as an acid to indirectly protonate Fe­(IV)=O and then as a base to deprotonate Tyr-3 during the PCET.

Preorganization of the protein environment to facilitate protonation of the Fe­(IV)=O intermediate might provide a mechanism to avoid dominant nitrate formation. Nitrate formation was considered as a competing pathway at two points along the reaction mechanisms. From **I-1**, a small barrier was identified for radical rebound of ^•^NO_2_ (**TS-9**) to irreversibly form nitrate (**P-4**) (Figure 7 red). **TS-9** was 7.4 kcal mol^–1^ lower in energy than **TS-2**, which in the absence of another pathway would imply exclusive nitrate formation; however, **TS-9** was similar in energy to proposed **TS-5**, enabling the subsequent nitration pathway via Cpd II protonation **I-3** and PCET **TS-6**. Furthermore, from **I-3**, ^•^NO_2_ rebound with Cpd II (**TS-10**) to form nitric acid (**P-5**) (Figure 7 pink) was within 0.7 kcal mol^–1^ of **TS-6**, suggesting the PCET would indeed be kinetically competitive. Nitric acid is also a much less thermodynamically favorable product. Given the computed barriers separating productive nitration from nitrate/nitric-acid formation in the present QM/MM model differ by less than 1 kcal mol^–1^, these calculations, rather than quantitatively proving kinetic suppression of nitrate formation, should be interpreted as supporting a plausible role of the Tyr-3/His-5/H235/water proton relay in biasing product formation. If protonation of the Fe­(IV)-oxo is required to avoid nitrate formation and to allow for productive nitration of Tyr-3, this would explain why the substrate NleRYL**Q** was not nitrated while NleRYL**E** demonstrated some nitration: Glu-5 can facilitate proton transfers while Gln-5 can only act as an H-bond donor.

### Crystal Structure of Substrate-Bound RufO Homologue NsRufO

To support the computational investigations, we attempted to obtain a bound structure of RufO in complex with a pentapeptide substrate (Nle-RYLH). Due to the low yield of RufO in *E. coli*, we turned to its homologue (NsRufO) from *Nonomurae solani* (with 46% identity), whose activity was confirmed with *in vivo* complementation using the Δ*rufO* strain constructed in the previous study (Figure S95).^1^ By soaking 100 mM substrate into the apo crystals obtained from PEG-containing conditions (0.1 M Tris-HCl, pH 8.0, 30% (w/v) PEG4000, 0.35 M lithium sulfate), yielding needle-shaped crystals with good diffraction, we successfully obtained the substrate-bound structure (space group *P*2_1_2_1_2) to a resolution of 1.59 Å (PDB ID: 22YX, Table S7).

NsRufO adopts the canonical P450 fold, with the thiolate-coordinated heme moiety at the center of the protein. Density surrounding the active site is well-defined, except for residues 178–180, which could not be resolved, consistent with relatively high flexibility between the F and G helices. The density of the substrate was found in the active site of NsRufO and was well resolved via fitting the Nle-RYLH pentapeptide (Figure 8a). Nle-RYLH is accommodated in the active-site pocket of NsRufO in an almost identical way to the structure of Nle-RYLH bound to RufO (PDB ID: 9DUJ). Specifically, Tyr-3 and His-5 are located directly above the heme, with Tyr-3 H-bonding to a water molecule W1 that remains coordinating to the heme iron (Figure 8b). The Arg-2 side chain forms a cation–pi interaction with F69, and the terminal carboxylate of the Nle-RYLH substrate forms a salt bridge with R231 and a water-mediated hydrogen bond with Q84. A slight shift in the position of R231 accommodates a 0.7 Å shift in the position of the His-5 terminal carboxylate compared to RufO, which creates a better H-bond alignment between His-5 and Tyr-3 (more linear N–H–O angle: 161° versus 134°; shorter N–O distance: 3.0 Å versus 3.2 Å). The Nle side chain is stabilized in a hydrophobic cage formed by L44, L48, T308, and M310. Direct interaction between the N-terminal substrate amine and NsRufO could not be observed, potentially due to the unresolved region (residues 178–180) of the protein backbone. Although the substrate conformation is highly similar to that seen in RufO, the I-helix H235 of NsRufO does not H-bond to His-5 as is seen in RufO.^13^ Instead, H235 adopts a conformation similar to that found in apo RufO,^17^ pointing “away” from the active site while His-5 interacts with E239, bridged by a water molecule W2. The different orientations of H235 could reflect distinct protonation states under varied crystallization buffer conditions and support the observation that this residue can explore different conformations; such states may represent different stages of the nitration reaction.

**Figure 8:**
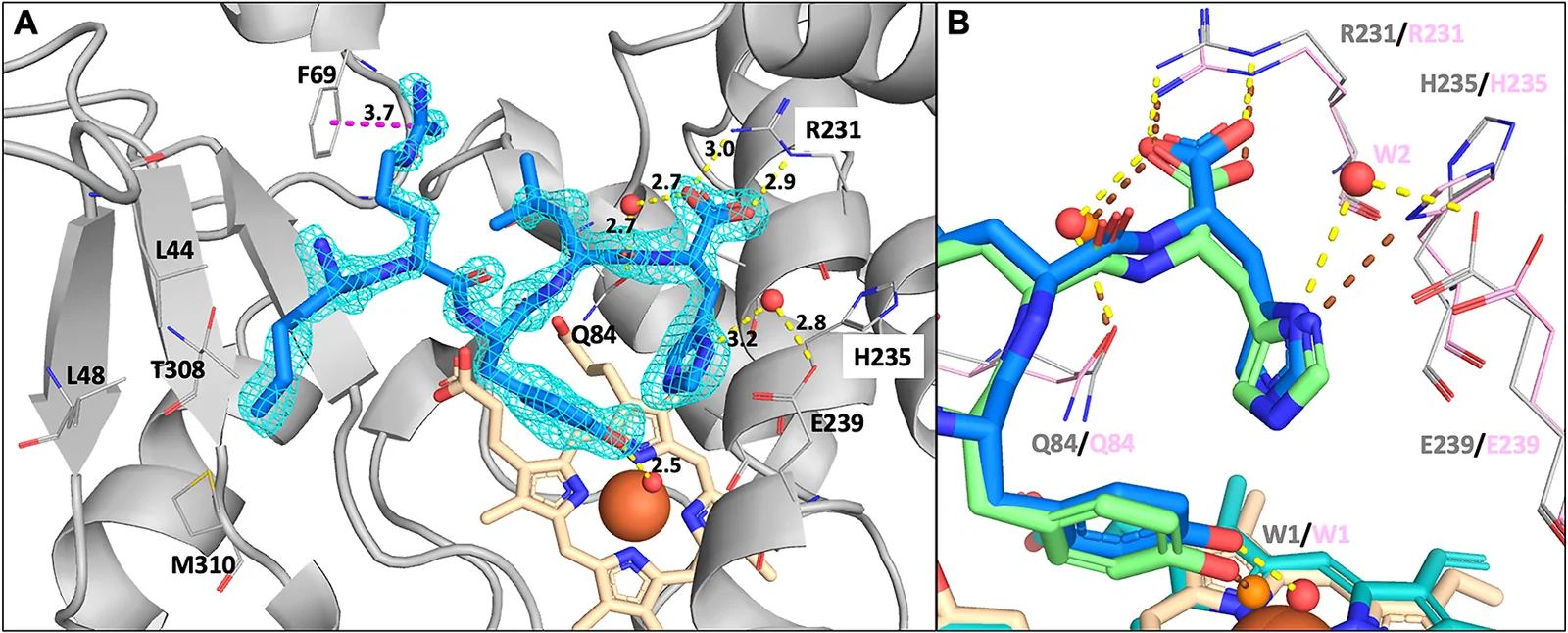
(A) Active site of Nle-RYLH substrate-bound NsRufO (PDB ID: 22YX) with key interactions: dotted lines show H-bonds (yellow) and cation–pi interactions (pink). The substrate is supported by density in the polder mFo-DFc map contoured at 3.5σ. (B) Comparison of the substrate-bound states of NsRufO and RufO (PDB ID: 9DUJ), focusing on the core region: NsRufO (gray) with Nle-RYLH (blue), NsRufO heme (tan), H-bonds (yellow), waters (red); RufO (pink) with Nle-RYLH (lime), RufO heme (cyan), H-bonds (brown), waters (orange).

### Protonation State of HIS-5

Based on the results obtained thus far, the most plausible mechanism for RufO-catalyzed MRYLH nitration is proposed to involve His-5 acting as a proton shuttle to facilitate protonation of Cpd II, followed by tyrosyl radical formation and radical recombination with ^•^NO_2_. However, it might be envisaged that a protonated His-5 could also directly participate as the proton source. Therefore, alternate protomeric forms of the substrate His-5 residue were explored through a series of classical MD simulations of RufO in the superoxide intermediate state in order to evaluate the feasibility of different protonation states. A comparison to the homologue NsRufO was also made. Furthermore, the protomeric forms of the nearby I-helix H235 and E239 residues were considered as changes in the local hydrogen bonding network were thought to influence the protonation state of His-5 (refer to Table S5 for summary of combinations employed).

First, we considered the acidic E239 residue. In the AF3 structure generated for RufO, E239 interacts with R243 and N165 and is therefore predicted to be deprotonated at pH 7 (H++ server).^38–40^ However, once protonated, E239 rotates and establishes an H-bond with D169, assuming a conformation similar to that found in the substrate-bound crystal structure (Figure 9A). Unsurprisingly, in the conformation from the substrate-bound crystal structure of RufO, E239 is predicted to be protonated (pH 7, H++ server). MD simulations initialized from the crystal structure and protonated form of E239 show that after 75 ns the initial H-bond between H235 and His-5 is lost in favor of H235 to E239 H-bonding; a water bridge forms between the F-helix D169 and His-5 while the substrate remains bound over the heme (Figure 9B). Comparatively, in the crystal structure of NsRufO, the I-helix H235 residue does not form an H-bond with His-5; instead, a water molecule bridges an interaction with the nearby, deprotonated (as predicted by H++ server at pH 7) E239. This indicates that the H-bond provided by H235 does not appear to be essential for the substrate positioning in RufO as it does in P450_Blt_.

**Figure 9:**
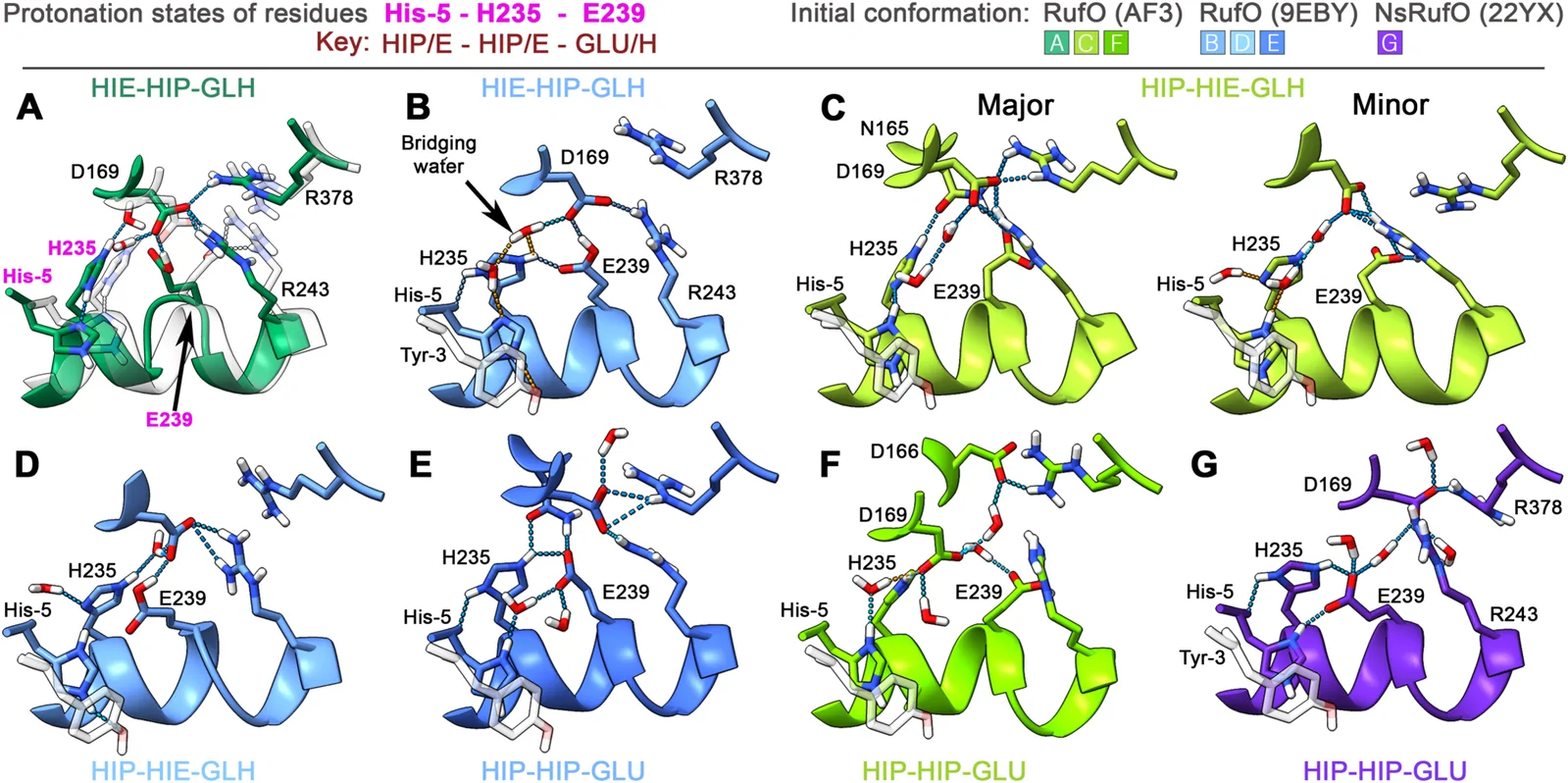
Snapshots from MD simulations of RufO and NsRufO with varied protonation states of residues His-5, H235, E239, denoted using the key HIX-HIX-GLX, where HIE is the histidine ε protonation state, HIP is protonated histidine, while GLU and GLH are deprotonated and protonated glutamic acid, respectively. H-bond showed with dashed lines. (A) Overlay of initial AF3 structure with E239 protonated, His-5 neutral, H235 protonated (HIE-HIP-GLH) with MD snapshot at 474.8 ns showing rotation of E239 to H-bond to D169. (B) MD snapshot at 446.6 ns from simulations initialized from the crystal structure 9EBY conformation with E239 protonated, showing a water-bridged interaction between His-5 and D169. (C) Representative snapshots of the two largest clusters after modeling a proton transfer from H235 to His-5: major (68%, 387.6 ns) and minor (15%, 264 ns). (D) Alternate conformation after proton transfer MD snapshot at 347.2 ns from simulations initialized from the 9EBY conformation. (E) Representative snapshots of major clusters in MD simulations with H235 and His-5 protonated initialized from the 9EBY conformation (blue, 27%, 178.6 ns) and (F) from AF3 (green, 70%, 178 ns). (G) Representative snapshot of the second-largest cluster occurring during MD simulations of NsRufO (32%, 85.2 ns).

Finally, protonated His-5 was considered. When neutral H235 and protonated His-5 are modeled, the His-5 H-bond to H235 is persistently maintained in the major conformational cluster (68%), alternating with minor conformations in which a water-bridged interaction again forms between His-5 and the acid residues D169 and/or E239 (Figure 9C). If E239 is also protonated, the major conformational cluster does not involve any H-bonding from His-5 to the I-helix residues, but E239 remains in proximity to the His-5 histidinyl cation (Figure 9D). If both His-5 and H235 are modeled as protonated, then a water bridge between His-5 and the acidic residues E239 and/or D169 is formed in the major conformational clusters (Figure 9E,F). Considering these protonation states for the NsRufO system, for the first 200 ns and last 30 ns of the MD simulation, a direct E239-His-5 interaction was observed, while otherwise bridged by water molecules (Figure 9G).

In all of the studied cases, the substrate remained bound in the active site over the heme. The population of the H-bond between His-5 and Tyr-3 remained modest, suggesting transient formation of catalytically competent conformations: 15% for HIP-HIE-GLH, 7% for HIP-HIP-GLU, and 11% for HIE-HIP-GLH. Taken together, these simulations indicate that upon binding, His-5 can adopt either a neutral or a protonated state, both of which are stabilized by interactions with the active-site residues E239 and D169, irrespective of whether H235 is neutral or protonated. These results further support the proposal that His-5 can participate directly in the nitration mechanism and suggest that, in both protonation states, it can act in tandem with H235 as part of a proton relay that facilitates the protonation of dp-Cpd II and the subsequent PCET pathway.

## Conclusion

RufO and its homologue NsRufO can catalyze tyrosine nitration of the pentapeptide substrate MRYLH. The proposed mechanism involves tyrosyl formation through either a hydrogen abstraction or a proton-coupled electron transfer. Radical recombination of the tyrosyl radical with ^•^NO_2_ generated from homolytic cleavage of heme–peroxynitrite results in a *keto* tautomer of 3-NO_2_-Tyr that must undergo tautomerization to provide the main thermodynamic driving force for the reaction. This contrasts with the proposed monoradical mechanism of TxtE-catalyzed tryptophan nitration. Loss of the abstractable phenolic hydrogen, as seen in potential substrates such as MRFLH, prevents nitration. Moreover, the imidazole of the substrate His-5 residue seems intimately involved through either H-bonding to Tyr-3 or as modeled here, as part of a proton relay. Based on the combined experimental evidence and extensive computational modeling, we propose that the hydrogen-bonding network involving the I-helix residues H235 and E239, together with an active role for His-5, operates cooperatively as a proton shuttle, a mechanism to disfavor nitrate formation and promote efficient tyrosine nitration. By elucidating the steps following heme–peroxynitrite formation, this study provides a stronger foundation for engineering P450s for selective peptide nitration, with the next challenge being to define the enzymatic features that determine peroxynitrite formation.

## Supplementary Material



## Data Availability

Optimized structures are deposited in the iochem-BD repository (doi: 10.19061/iochem-bd-6–633).

## Notes

Experimental and computational methods, data analysis, peptide characterization (LC-MS^2^, ^1^H NMR, ^13^C NMR, COSY, HMBC, and HSQC spectra, binding curves), crystallographic data summary, computed geometries, DFT coordinates, Mulliken spin densities, and complementation data (PDF)

## ¶

International Center for Biotechnology, The University of Osaka, Suita, 565–0871, Japan
