# Supplementary material for: Mechanism for Aromatic Nitration Catalyzed by Cytochrome P450 Enzymes from Rufomycin Biosynthesis
Source: J Am Chem Soc. 2026 Jul 29;148(39):41873–85. doi: 10.1021/jacs.6c05584 (PMC13651536; doi:10.1021/jacs.6c05584)
Supplement: Supplementary file 1 [file ja6c05584_si_001.pdf]

Supporting Information

Mechanism for Aromatic Nitration Catalyzed by Cytochrome P450 Enzymes from Rufomycin Biosynthesis

Alicia M. Kirk,[a,b,] Jemma Gullick,[c,d] Yongwei Zhao,[e] Ralf B. Schittenhelm,[f] Ferran Feixas,[g] Hiroya Tomita,[e,†] Yohei Katsuyama,[e,i] Yasuo Ohnishi,[e,i] James J. De Voss,[a,d] Max J. Cryle,[c,d,]* and Marc Garcia-Borràs[g,h,]**

[a] School of Chemistry and Molecular Biosciences, The University of Queensland, Brisbane, QLD 4067, Australia

[b] Scuola Normale Superiore, Pisa, PI 56126, Italy

[c] Department of Biochemistry and Molecular Biology, The Monash Biomedicine Discovery Institute, Monash University, Clayton, VIC 3800, Australia

[d] ARC Centre of Excellence for Innovations in Peptide and Protein Science, Clayton, VIC 3800, Australia

[e] The Graduate School of Agricultural and Life Sciences, The University of Tokyo, Bunkyo-ku, Tokyo 113-8657, Japan

[f] Proteomics and Metabolomics Platform, Monash University, Clayton, VIC 3800, Australia

[g] Institut de Química Computacional i Catàlisi (IQCC) and Departament de Química, Universitat de Girona, Campus Montilivi, Girona, 17003, Spain

[h] ICREA, Passeig Lluís Companys, 23, 08010 Barcelona, Spain

† Current address: International Center for Biotechnology, The University of Osaka, Suita, Japan, 565-0871

Corresponding Authors

*E-mail: alicia.kirk@sns.it (A.M.K.)

*E-mail: max.cryle@monash.edu (M.J.C.)

*E-mail: marc.garcia@udg.edu (M.G.B)

Table of Contents

Experimental Methods **1**
- Chemicals 1
- Peptide Synthesis 1
- Preparatory HPLC and LC-MS analysis of peptides 1
- NMR analysis 1
- Expression and Purification of RufO 1
- Substrate Turnover 2
- P450/Substrate Binding Assay 2
- High Resolution Mass Spectrometry (HRMS) 2
- Production of NsRufO 3
- Structural elucidation of NsRufO by X-ray crystallography 3
- Complementation of the Δ*rufO* strain using *NsrufO* 3

Computational Methods **4**
- Density Functional Theory Calculations 4
- Molecular Dynamics Simulations 4
- ONIOM Calculations 5

Structural characterization of peptides used for *in vitro* studies **6**

SDS-PAGE analysis of RufO and NsRufO **59**

Analysis of *in vitro* enzymatic assays with RufO and NsRufO **60**

Analysis of structural prediction of RufO **70**

Small DFT models of nitration catalysed by RufO **74**

Analysis of molecular dynamics simulations **78**

ONIOM model of nitration catalysed by RUFO **82**

Complementation of rufomycin production **86**

Coordinates **87**

References **102**

List of Figures

Figure S1. Structures of peptides in this study. 6
Figure S2. LC-MS characterization of Nle-RYLH....9
Figure S3. Annotated MS^2 spectrum of Nle-RYLH. 10
Figure S4. LC-MS characterization of Nle-**A**YLH....11
Figure S5. Annotated MS^2 spectrum of Nle-**A**YLH. 12
Figure S6. LC-MS characterization of Nle-R**F**LH....13
Figure S7. Annotated MS^2 spectrum of Nle-R**F**LH. 14
Figure S8. LC-MS characterization of Nle-R-**(OMe)Tyr**-LH....15
Figure S9. Annotated MS^2 spectrum of Nle-R-**(OMe)Tyr**-LH. 16
Figure S10. LC-MS characterization of Nle-R-**mTyr**-LH....17
Figure S11. Annotated MS^2 spectrum of Nle-R-**mTyr**-LH. 18
Figure S12. LC-MS characterization of Nle-RY**Y**H. 19
Figure S13. Annotated MS^2 spectrum of Nle-RY**Y**H....20
Figure S14. LC-MS characterization of Nle-RYL**E**....21
Figure S15. Annotated MS^2 spectrum of Nle-RYL**E**. 22
Figure S16. LC-MS characterization of Nle-RYL**Q**. 23
Figure S17. Annotated MS^2 spectrum of Nle-RYL**Q**. 24
Figure S18. LC-MS characterization of Nle-RYL-**Thz**....25
Figure S19. Annotated MS^2 spectrum of Nle-RYL-**Thz**. 26
Figure S20. LC-MS characterization of Nle-RYL**W**....27
Figure S21. Annotated MS^2 spectrum of Nle-RYL**W**. 28
Figure S22. ^{1}H NMR of Nle-RYLH in DMSO-d_6. 29
Figure S23. ^{13}C NMR of Nle-RYLH in DMSO-d_6....29
Figure S24. ^{1}H-^{1}H correlated spectroscopy spectra of Nle-RYLH in DMSO-d_6. 30
Figure S25. HSQC spectrum of Nle-RYLH in DMSO-d_6. 30
Figure S26. HMBC spectrum of Nle-RYLH in DMSO-d_6....31
Figure S27. ^{1}H NMR of Nle-AYLH in DMSO-d_6. 32
Figure S28. ^{13}C NMR of Nle-AYLH in DMSO-d_6....32
Figure S29. ^{1}H-^{1}H correlated spectroscopy spectrum of Nle-**A**YLH in DMSO-d_6. 33
Figure S30. HSQC spectrum of Nle-**A**YLH in DMSO-d_6. 33
Figure S31. HMBC spectrum of Nle-**A**YLH in DMSO-d_6....34
Figure S32. ^{1}H NMR of Nle-R**F**LH in DMSO-d_6. 35
Figure S33. ^{13}C NMR of Nle-R**F**LH in DMSO-d_6....35
Figure S34. ^{1}H-^{1}H correlated spectroscopy spectrum of Nle-R**F**LH in DMSO-d_6. 36
Figure S35. HSQC spectrum of Nle-R**F**LH in DMSO-d_6. 36
Figure S36. HMBC spectrum of Nle-R**F**LH in DMSO-d_6. 37
Figure S37. ^{1}H NMR of Nle-R-**(OMe)Tyr**-LH in DMSO-d_6. 38
Figure S38. ^{13}C NMR of Nle-R-**(OMe)Tyr**-LH in DMSO-d_6. 38
Figure S39. ^{1}H-^{1}H correlated spectroscopy spectrum of Nle-R-**(OMe)Tyr**-LH in DMSO-d_6. 39
Figure S40. HSQC spectrum of Nle-R-**(OMe)Tyr**-LH in DMSO-d_6. 39
Figure S41. HMBC spectrum of Nle-R-(OMe)Tyr-LH in DMSO-d_6. 40
Figure S42. ^{1}H NMR of Nle-R-**mTyr**-LH in DMSO-d_6. 41
Figure S43. ^{13}C NMR of Nle-R-mTyr-LH in DMSO-d_6. 41
Figure S44. ^{1}H-^{1}H correlated spectroscopy spectrum of Nle-R-**mTyr**-LH in DMSO-d_6. 42
Figure S45. HSQC spectrum of Nle-R-**mTyr**-LH in DMSO-d_6. 42
Figure S46. HMBC spectrum of Nle-R-**mTyr**-LH in DMSO-d_6. 43
Figure S47. ^{1}H NMR of Nle-RY**Y**H in DMSO-d_6....44
Figure S48. ^{13}C NMR of Nle-RY**Y**H in DMSO-d_6. 44
Figure S49. ^{1}H-^{1}H correlated spectroscopy spectrum of Nle-RY**Y**H in DMSO-d_6....45
Figure S50. HSQC spectrum of Nle-RY**Y**H in DMSO-d_6. 45
Figure S51. HMBC spectrum of Nle-RY**Y**H in DMSO-d_6. 46
Figure S52. ^{1}H NMR of Nle-RYL**E** in DMSO-d_6. 47
Figure S53. ^{13}C NMR of Nle-RYL**E** in DMSO-d_6....47
Figure S54. ^{1}H-^{1}H correlated spectroscopy spectrum of Nle-RYL**E** in DMSO-d_6. 48
Figure S55. HSQC spectrum of Nle-RYL**E** in DMSO-d_6. 48
Figure S56. HMBC spectrum of Nle-RYL**E** in DMSO-d_6. 49
Figure S57. ^{1}H NMR of Nle-RYL**Q** in DMSO-d_6. 50
Figure S58. ^{13}C NMR of Nle-RYL**Q** in DMSO-d_6. 50
Figure S59. ^{1}H-^{1}H correlated spectroscopy spectrum of Nle-RYL**Q** in DMSO-d_6. 51
Figure S60. HSQC spectrum of Nle-RYL**Q** in DMSO-d_6. 51

Figure S61. HMBC spectrum of Nle-RYL**Q** in DMSO-d_6. 52
Figure S62. ^{1}H NMR of Nle-RYL-**Thz** in DMSO-d_6. 53
Figure S63. ^{13}C NMR of Nle-RYL-**Thz** in DMSO-d_6. 53
Figure S64. ^{1}H-^{1}H correlated spectroscopy spectrum of Nle-RYL-**Thz** in DMSO-d_6. 54
Figure S65. HSQC spectrum of Nle-RYL-**Thz** in DMSO-d_6. 54
Figure S66. HMBC spectrum of Nle-RYL-**Thz** in DMSO-d_6. 55
Figure S67. ^{1}H NMR of Nle-RYL**W** in DMSO-d_6. 56
Figure S68. ^{13}C NMR of Nle-RYL**W** in DMSO-d_6. 56
Figure S69. ^{1}H-^{1}H correlated spectroscopy spectrum of Nle-RYL**W** in DMSO-d_6. 57
Figure S70. HSQC spectrum of Nle-RYL**W** in DMSO-d_6. 57
Figure S71. HMBC spectrum of Nle-RYL**W** in DMSO-d_6. 58
Figure S72. SDS-PAGE analysis of RufO and NsRufO. 59
Figure S73. *In vitro* enzymatic nitration and crosslinking results for RufO 60
Figure S74. *In vitro* nitration results for Nle-RYLH with a) RufO and b) NsRufO. 61
Figure S75. HRMS and MS^2 spectra for the nitrated product of the turnover of Nle-RYLH with RufO. 62
Figure S76. HRMS and MS^2 spectra for the nitrated product of the turnover of Nle-**A**YLH with RufO. 63
Figure S77. HRMS and MS^2 spectra for the nitrated product of the turnover of Nle-R-**mTyr**-LH with RufO. 64
Figure S78. HRMS and MS^2 spectra for the nitrated product of the turnover of Nle-RY**Y**H with RufO. 65
Figure S79. HRMS and MS^2 spectra for the nitrated product of the turnover of Nle-RYL**E** with RufO. 66
Figure S80. HRMS and MS^2 spectra for the crosslinked product of the turnover of Nle-RYL**W** with RufO. 67
Figure S81. Binding curves to RufO 68
Figure S82. Predicted structure of MRYLH bound RufO with heme cofactor present obtained using the Alphafold Server 70
Figure S83. Comparison of alphafold3 structure (blue) and PDB 8SPC (orange). 71
Figure S84. Comparison of $P450_{Blt}$ PDB 8U2M (grey) and RufO PDB 8SPC (orange). 72
Figure S85. Comparison of $P450_{Blt}$ PDB 8U2M (grey) and RufO alphafold prediction (blue). 73
Figure S86. Barrier for isomerisation of *cis*-peroxynitrite to *trans*-peroxynitrite 74
Figure S87. Gas phase radical recombination and isomerization compared with radical addition. 74
Figure S88. Potential mechanisms of nitration following formation of $^{\bullet}NO_2$ 75
Figure S89. Abstraction of N-**H** by $^{\bullet}NO_2$ radical, comparing methylated indole and imidazole 75
Figure S90. Phenol hydrogen abstraction by a) Cpd II with hydrogen bonding from imidazole, ...76
Figure S91. RMSD (Å) of protein (black) and substrate MRYLH (turquoise) for triplicate MD simulations 78
Figure S92. Representative snapshot from the major cluster in MD simulations chosen for ONIOM model 79
Figure S93. Geometries of stationary points calculated using ONIOM(DFT:MM) for the potential mechanisms 82
Figure S94. Geometries of stationary points calculated using ONIOM(DFT:MM) for alternate mechanisms 83
Figure S95. Complementation of rufomycin production in the *Streptomyces atratus* ATCC 14046 Δ*rufO* strain 86

List of Tables

Table S1. Comparison of ΔAbs_{max}, K_d and $\Delta Abs_{max}/K_d$ as an indication of binding efficiency of peptides to RufO. 69
Table S2. Key Mulliken spin densities for the different calculated species using the truncated model with DFT 77
Table S3. Key bond distances (Å) and bond angles (°) for the structures calculated using the truncated model with DFT 77
Table S4. Percentage of MD snapshots in which specified hydrogen bonds are present 80
Table S5. Summary of combination of protonation states explored in MD simulations of RufO and NsP450 81
Table S6. Key Mulliken spin densities and energies (Hartree) for the different calculated species using ONIOM(DFT:MM) 84
Table S7. Crystallographic data summary for NsRufO binding with the substrate (Nle-RYLH). 85
Table S8. Primers used in the production of NsRufO and complementation of the ΔRufO in this study. 86

Experimental Methods

Chemicals

Acetonitrile (ACN, Sigma-Aldrich), δ-aminovulinic acid hydrochloride (Sigma-Aldrich), Benzonase® nuclease (Sigma-Aldrich), *N,N*-diisopropylethylamine (DIPEA, Sigma-Aldrich), diethylamine NONOate (DEANO, Sigma-Aldrich), diethyl ether (Sigma-Aldrich), dimethylformamide (DMF, Thermo Fisher), Fmoc-AA-OH (Merck), formic acid (FA, Sigma-Aldrich), glucose (Sigma-Aldrich), glucose dehydrogenase (Sigma-Aldrich), HEPES (Sigma-Aldrich), imidazole (Sigma-Aldrich), isopropyl-β-D-thiogalactopyranoside (IPTG, Apollo Scientific), kanamycin sulfate (Sigma-Aldrich), magnesium chloride (Sigma-Aldrich), methanol (Scharlau), NADH (Apollo Scientific), Ni-NTA beads (Macherey-Nagel), sodium chloride (Sigma-Aldrich), triethylamine (TEA, Sigma-Aldrich), trifluoroacetic acid (TFA, Oakwood Chemical), triisopropyl silane (TIPS, Sigma-Aldrich), trizma base (Tris, Sigma-Aldrich), (1-cyano-2-ethoxy-2-oxoethylidenaminooxy)dimethylamino-morpholino-carbenium hexafluorophosphate (COMU, Oakwood).

Peptide Synthesis

Peptide synthesis was performed manually at a 0.1 mmol scale using (1-cyano-2-ethoxy-2-oxoethylidenaminooxy)dimethylamino-morpholino-carbenium hexafluorophosphate (COMU) and triethylamine (TEA) as coupling reagents. 2-chlorotrityl chloride resin (100 mg) was swelled with dichloromethane (DCM, 3 mL, 30 min) and the first Fmoc-protected amino acid (0.1 mmol) was coupled to the resin overnight using *N,N*-diisopropylethylamine (DIPEA). Unreacted chlorotrityl groups were capped using DMF/DIPEA/methanol (7:2:1, 5 mL, 15 min). Fmoc removal was achieved with 20% piperidine in DMF and subsequent amino acid coupling was performed using the appropriate Fmoc-amino acid (0.3 mmol), COMU (0.3 mmol) and TEA (0.6 mmol) at room temperature for one hour. Coupling reactions were repeated until the sequence was complete and a final deprotection was performed. Cleavage from resin was performed using TFA/TIS/H_2O (95: 2.5: 2.5, v: v': v'', 5 mL) with shaking for two hours at room temperature followed by filtration to separate the resin from TFA solution. The filtrate was concentrated under a stream of N_2 and the peptide was precipitated by addition of cold diethyl ether (10 volumes). This was then centrifuged and washed with diethyl ether (3 x 5 mL) affording crude peptide. Peptides were then purified using a preparatory HPLC (Shimadzu) and analysed using LC-MS (Shimadzu).

Preparatory HPLC and LC-MS analysis of peptides

Preparatory HPLC was conducted using an Agilent ZORBAX column (SB-C18, 7 μm, 21.2 x 250 mm) using a gradient of 0-60% in 35 minutes of acetonitrile + 0.1% TFA in water + 0.1% TFA and a flow rate of 10 mL/min.

LC-MS analysis of peptides was conducted on a Shimadzu high performance liquid chromatography system coupled to a mass spectrometer LCMS-2020 equipped with an SPD-20A Prominence photo diode array detector and a LC-20AD solvent delivery module. Peptides were analyzed on an Agilent C18 column (300SB-C18, 5 μm, 4.6 mm x 250 mm) using a 5-95% gradient in 20 minutes of acetonitrile + 0.1% formic acid in water + 0.1% formic acid and a flow rate of 1 mL/min. Mass spectrometry data was collected using ESI in both positive and negative mode.

NMR analysis

NMR spectra of all peptides except Nle-RYL-**Thz** were collected at 298 K on a Bruker Avance III NMR spectrometer equipped with a 9.4 T magnet and 5 mm BBFO probe, operating at 400 MHz (^{1}H), 100 MHz (^{13}C). NMR spectra of Nle-RYL-**Thz** were collected at 298 K on a Bruker Avance III NMR spectrometer equipped with a 14.1 T magnet and 5 mm TCI cryoprobe, operating at 600 MHz (^{1}H), 150 MHz (^{13}C). ^{1}H and ^{13}C chemical shifts are reported in ppm and are referenced to residual solvent signals.

Expression and Purification of RufO

Plasmid was transformed into *E. coli* ArcticExpress (DE3) competent cells (NEB) using heat shock methodology. Cultures of 1 L TB medium containing kanamycin (50 μg/mL) and δ-aminolevulinic acid (0.1 mM) were inoculated with 10 mL of pre-culture. Cultures were incubated at 37 °C until an OD_{600} was 0.8 then cultures were placed at 4 °C for 30 minutes. IPTG (0.05 mM) was added to each culture, and the cultures were incubated at 18 °C overnight. Cells were harvested by centrifugation, resuspended in lysis buffer of 50 mM Tris.HCl pH 8.0, 300 mM NaCl, 10 mM imidazole with Benzonase® (25 U/L cell culture) before lysis by cell disruption (Avestin Emulsiflex C5). Lysate

was clarified by centrifugation, and the supernatant was incubated with Ni-NTA agarose. RufO was eluted from Ni-NTA agarose using 500 mM imidazole. The protein was diluted to achieve a low salt concentration prior to anion exchange chromatography with a HiTrap Q HP AEX (Cytiva) column with a gradient of 10 mM – 1 M NaCl. The protein was then subjected to size exclusion chromatography using a Sepax SRT-10 Sec-300 column in a buffer of 50 mM Tris.HCl pH 7.4 and 100 mM NaCl. Concentration of the protein was determined by measuring the absorbance at 418 nm and purified protein were stored at -80 °C.

Substrate Turnover

All proteins were expressed and purified as previously described.[1] Small scale *in vitro* nitration reactions were performed as previously described.[2] Briefly, HEPES pH 7.0 (50 mM), NaCl (50 mM), RufO (0.5 μM), PuR (0.5 μM), PuxB A105V mutant[3] (2.5 μM), glucose (0.33%), glucose dehydrogenase (0.033 mg/mL), peptide substrate (50 μM), NADH (200 μM) and DEANO (diethylamine NONOate, 0.5 mM) were combined and incubated overnight at 30 ℃ shaking at 300 rpm. Methanol + 0.1% formic acid (3.5 volumes) was then added, the samples were centrifuged to remove salt and protein, the supernatant was transferred to a new tube and dried using a concentrator (Eppendorf). Samples were prepared for LC-MS and HRMS analysis by solvation in acetonitrile/water (50 μL, 1:4, 0.1% formic acid) and analysed on a Shimadzu high-performance liquid chromatography system with an Agilent ZORBAX 300SB-C_{18} 5 μm column, coupled to a mass spectrometer LC-MS-2020 (ESI, operating in both positive and negative mode) equipped with an SPD-20A Prominence photo diode array detector and an LC-20AD solvent delivery module. Turnover assays were analyzed on a gradient of 0-60% in 35 mins of acetonitrile + 0.1% formic acid in water + 0.1 % formic acid with a flow rate of 1 mL/min. HRMS analyses are described below. Reactions were analyzed by determining the % conversion to nitrated or crosslinked product based on TIC of the relevant m/z of the product compared to the starting material. All turnovers were conducted in triplicate with error bars showing SD in relevant figures.

P450/Substrate Binding Assay

Cytochrome P450-substrate binding interactions were measured by UV-visible spectroscopy with a Jasco V-750 spectrophotometer at 30 °C. RufO was diluted to 3 μM in Tris-HCl buffer (50 mM, pH 8.0) and split into two cuvettes (sample and reference cuvettes). A baseline spectra measurement between λ = 350 and 600 nm. Substrate was then titrated into the sample cuvette in a stepwise manner, and the same amount of buffer was added to the reference cuvette with spectra measured after a two-minute incubation for each substrate addition. The resulting amplitudes were calculated (ΔAbs = A_{max} – A_{min}) and plotted against substrate concentration. Titrations were conducted across a substrate concentration range of 0 – 200 μM or until ΔAbs reached a plateau. As A_{max} and A_{min} varied between peptides, wavelengths for determining ΔAbs were chosen based on A_{max} and A_{min} at the highest peptide concentration and used for all other concentrations of the same peptide. Curve fitting of the resulting data points was performed using GraphPad Prism 10 with a one-site binding model. Duplicate measurements taken where binding curve plateaued at less than 200 μM of substrate.

High Resolution Mass Spectrometry (HRMS)

HRMS was performed on an Orbitrap Eclipse Tribrid mass spectrometer (Thermo Scientific) coupled to a Neo UHPLC (Thermo Scientific), an Acclaim PepMap RSLC analytical column (75 µm x 50 cm, nanoViper, C18, 2 µm, 100 Å; Thermo Scientific) and an Acclaim PepMap 100 trap column (100 µm x 2 cm, nanoViper, C18, 5 µm, 100 Å; Thermo Scientific). Analytes were separated in increasing concentrations of 80% acetonitrile / 0.1% formic acid at a flow of 250 nL/min over 54 mins. The instrument was operated in alternating data-dependent acquisition (DDA) and parallel reaction monitoring (PRM) cycles in such that 10 ms2 scans were triggered per survey ms1 scan followed by several targeted ms2 scans to ensure fragmentation of predefined, sample-dependent m/z precursors. Each survey ms1 scan (375-1575 m/z) was acquired with a resolution of 60,000 and a normalised AGC (automatic gain control) target of 200%. Dynamic exclusion was set to 30 seconds after one occurrence. The 10 most intense ions were selected for HCD fragmentation (fixed normalized collision energy mode, 24-30% HCD Collision Energy) with a resolution of 15-30,000, an absolute AGC target of 5e4 and a dynamic scan range based on precursor m/z. Subsequent targeted ms2 scans were acquired with essentially identical settings. The raw data files were analysed with QualBrowser (XCalibur 3.0.63, Thermo Scientific) to view spectra and to generate extracted ion chromatograms.

Production of NsRufO

Nonomuraea solani JCM 30783 was purchased from RIKEN BRC Microbe division (JCM). *NsrufO* was amplified by PCR (using PrimeStar polymerase, primers NsP450-Nde-F and NsP450-Eco-R, following standard TaKaRa PCR protocol). The amplified DNA was digested with NdeI and EcoRI and cloned into pET26b NdeI and EcoRI sites using T4DNA ligase, resulting pET26b-NsRufO. The BL21(DE3) harboring pET26b-NsRufO (obtained using standard heat shock protocol) was inoculated into terrific broth (24 g/L yeast extract, 12 g/L tryptone, 0.8% glycerol, 0.94% K_2HPO_4, and 0.22% KH_2PO_4) with 5-aminolevulinic acid (80 mg/liter), $Fe(NH_4)_2(SO_4)_2$ (40 mg/liter), and kanamycin (60 mg/liter) and incubated at 37°C with shaking until OD_{600} reaches ~0.6. After the addition of 0.1 mM isopropyl 1-thio-β-d-galactopyranoside (IPTG), the culture was further incubated at 15°C for 24 hours. Protein purification was performed using His60 Ni Superflow Resin and Anion Exchange (AEX) chromatography. The bacteria were harvested via centrifugation (4000 rpm, 10 min/RT). The pellet was resuspended in lysis buffer (20 mM Tris-HCl, pH 8.0, 200 mM NaCl, 10% (v/v) glycerol) and lysed by ultrasonication on ice (25% amplitude, on 2s, off 2s). The lysate was clarified via centrifugation (13000 rpm, 45 min, 4°C) and the supernatant was then applied to 2 mL of His60 Ni Superflow resin. After being eluted by lysis buffer containing 0.5M imidazole, the protein fraction was buffer-exchanged into AEX buffer A (10 mM Tris-HCl, pH 8.0, 10 mM NaCl, 10% (v/v) glycerol) and loaded onto HiTrap Q HP column (1 mL, Cytiva). Elution was achieved with a gradient of 0% to 100% AEX buffer B (10 mM Tris-HCl, pH 8.0, 1 M NaCl, 10 % (v/v) glycerol) in 10 mins with 1 mL/min flow rate on an ÄKTA pure™ 150 system. The colored fractions were examined on SDS-PAGE. Clean fractions containing NsRufO were combined and buffer-exchanged into lysis buffer for crystallization use.

Structural elucidation of NsRufO by X-ray crystallography

The resulting protein solution was mixed with 1.0 μL of the crystallization buffer of the screening kit (Crystal Screen, Index, Wizard Ⅰ, Wizard Ⅱ) in a sitting drop vapor-diffusion method and incubated at 20°C for several days. A secondary screening to obtain good quality crystals was then performed using buffers with 0.2 M $LiSO_4$ 0.1 M Tris-HCl (pH 7.5) and varying concentrations of PEG4,000. The enzyme solution (2.0 μL) and buffer for crystallization (2.0 μL) were mixed in a hanging drop vapor-diffusion method and incubated at 20°C for several days. For soaking of the substrates, 100 mM of the substrate were added to the crystals and incubated at room temperature for 30 min before flash frozen with liquid nitrogen.

The diffraction datasets of NsRufO were collected at beamline BL-1A of the Photon Factory (KEK, Tsukuba, Japan), with an X-ray wavelength of 1.007 Å. The datasets were processed using XDS and scaled using AIMLESS.[4] The crystal structure of NsRufO was determined by molecular replacement with PHENIX.PHASER[5] using the structure predicted by AlphaFold2[6] and refined using PHENIX.REFINE.[7] The molecular models were built using COOT.[8] The geometry of the refined crystal structure was assessed using MolProbity in PHENIX.

Complementation of the Δ*rufO* strain using *NsrufO*

The *NsrufO* gene was amplified by PCR using genomic DNA of *N. solani* as a template (using PrimeStar polymerase, primers NsP450-pTYM-IF-F and NsP450-pTYM-IF-R, following standard TaKaRa PCR protocol) and cloned into the pTYM19ep, which was also amplified with a PCR (PrimeStar polymerase, primers NsP450-pTYM-IF-F2 and NsP450-pTYM-IF-R2, following standard TaKaRa PCR protocol) under the control of the *ermE** promoter via Infusion cloning, resulting in pTYM19ep-*NsrufO*. The PCR followed the standard TaKaRa PCR protocol, and the primers sequences are listed in Table S8. The plasmid was conjugated into the *Streptomyces atratus* ΔrufO strain with *E. coli* ET12567/pUZ8002 following the method as described previously. [9] The production and extraction of rufomycin carried out by using the same method as described previously.[9] The extracted metabolites were analyzed by liquid chromatography-electrospray ionization mass spectrometry (LC-ESIMS) using an LC-2040C 3D Plus system (Shimazu Corp., Kyoto, Japan) equipped with a COSMOCORE 2.6C18 Packed column (2.1 mm ID×100 mm, Nacalai Tesque) coupled with a model LCMS-8040 liquid chromatography-mass spectrometer (LC-MS) (Shimadzu Corp.). The genomic DNA isolation, *rufO* depletion, and transformation are conducted using the same method as described previously.[9]

Computational Methods

Density Functional Theory Calculations

RufO was truncated to iron(III) porphine ligated by SMe and peroxynitrite. 4-Methylphenol was used as a model substrate. All calculations were performed using Gaussian16 (rev C01)[10] Geometry optimization was performed using B3LYP[11-12] with the LanL2DZ basis set and pseudopotential[13] on Fe and the 6-31G(d)[14-15] basis set for all other elements, to be called BS1. TS structures were confirmed by the presence of 1 imaginary frequency and via intrinsic reaction coordinate (IRC) calculations through which subsequent intermediates were also identified. Intermediate structures were confirmed to have 0 imaginary frequencies. Zero-point energy (SPE) corrections were applied to all species. Single point (SP) calculations using B3LYP-D3BJ[16] and the extended 6-311+G(d,p)[17-20] for H, C, N, O, S atoms while retaining LanL2DZ for Fe (i.e. BS2), were used to improve the reported energies. The lowest energy spin state is reported which is the doublet spin state throughout. Note the quartet spin state was similar in energy for the Cpd II intermediate. Altering the initial scf guess was occasionally required to find the appropriate lowest energy solution.

Molecular Dynamics Simulations

Alphafold3[6] was used to generate the initial substrate bound structure of RufO with the missing FG loop included and the peptide substrate docked. The first 31 residues of low confidence prediction were removed. Either the superoxo or peroxynitrite ligand was then manually added to the heme. For $P450_{Blt}$, the substrate bound crystal structure (PDB ID: 8U2M)[21] was used for the initial conformation. The H++ server (version 4.0, http://biophysics.cs.vt.edu/H++)[22-24] was then used to identify the protonation state of titratable residues at pH 7.4. Note the I-helix histidine 235 was assigned the HIP protonation state.

Parameters for the 6-coordinate ferric-superoxo intermediate were sourced from Shahrokh and coworkers.[25] Parameters for the 6-coordinate ferric-peroxynitrite intermediate were generated using the MCPB.py module.[26] Charges were fit using a two-stage multi-fit RESP method to the electrostatic potential calculated in Gaussian16 through the Merz-Singh-Kollman scheme[27-28] at the B3LYP/BS1 level of theory with implicit water (SMD model[29]) in multiple molecular rotations of the geometry optimized structure of the large model. A 1.4 Å radius for Fe was used. The Seminario method[30] was chosen to generate necessary bond and angle parameters. Symmetry across the porphyrin N-Fe bonds was imposed. Additional bond, angle and dihedral terms for OONO were calculated using *CartHess2FC.py*. The Amber 14 forcefield with Stony Brook modification (ff14SB)[31] was employed for the protein. Using *tleap* in AmberTools24, the system was solvated in a 10 Å box of TIP3P water[32] and the charge was neutralized through the addition of Na+ ions. Simulations were performed with AMBER24 using *pmemd*.[33-34]

The potential energy of the system was minimized, first with 500 kcal mol^{-1} Å^{-2} restraints on the protein, cofactor and ligand, and then releasing those restraints. Reimposing 210 kcal mol^{-1} Å^{-2} restraints, the system was then heated to 300K in six sequential 20 ps steps of 50 K using a 2 fs time step, employing the Langevin thermostat (γ = 1.0) under constant volume (NVT) and periodic boundary conditions. Long-range interactions were treated with the particle mesh Ewald (PME) method.[35] A 10 Å cutoff was used for short-range interactions. SHAKE was used to constrain bonds involving hydrogen.[36] The restraints were released, and the system equilibrated for 2.2 ns under constant pressure (NPT) at 1 bar employing the Berendsen barostat (taup = 2.0). Production runs were then performed in triplicate for 500 ns under NVT. Trajectories were analyzed using *cpptraj*[37] in AmberTools24. All hydrogen-bond occupancies reported quantitatively in the manuscript are defined using a donor–acceptor distance ≤3.0 Å and a donor–H–acceptor angle ≥135°; relaxed ChimeraX contacts are used only for structural visualization and not for population estimates. Hierarchical clustering was performed based on protein RSMD and then specifically residues Q84, R231, H235, the heme and the pentapeptide substrate with epsilon 3.0. Representative snapshots were selected from the most populated cluster.

To explore other protonation states, a further series of single run MD simulations were also performed for MRYLH bound RufO and analogue NsP450 with the ferric superoxo intermediate, each for 500 ns. Initial starting configurations were taken from both the RufO AF3 predicted structure and crystal structure (PDB ID: 9EBY), and NsP450 crystal structure (PDB ID: 22YX) (refer to Table S5). System preparation and simulations were performed as described above.

ONIOM Calculations

Representative snapshots from MD simulations were used to obtain starting conformations. The MolUP VMD plugin was used for the preparation of input files.[38-39] Water molecules >3 Å from the protein were stripped. The high layer was defined as the heme with truncated sidechains, coordinating cysteine truncated to SMe, and the sidechains of H235 and substrate His-5 and Tyr-3. Residues and water outside 12 Å of the high layer were frozen while those within were free to move.

Using Gaussian 16,[10] a two-layer ONIOM(DFT:AMBER) method was employed.[40-41] For the high layer, B3LYP with the 6-31G(d) basis for H, C, O, N, S with the SDD basis set and pseudopotential[42] for Fe (i.e. BS3) was used. The coupled, quadratic macro step was employed, along with the YQC algorithm to assist with SCF convergence. Symmetry and archiving were turned off. For the low layer, the forcefield parameters employed in the MD simulations were used: the Amber topology file was converted to Gaussian readable input using the command line utility in MolUP. Geometries were optimized first with mechanical embedding. Following previously validated methodology,[43] water molecules were then frozen before further geometry optimization using electronic embedding, an effective method to avoid micro-iteration errors. Relaxed scanning and redundant coordinates were used to assist in the location of TS structures. Minima and TS structures were verified based on the number of imaginary frequencies present in the calculated normal modes (0 and 1, respectively). Zero-point energy corrections were applied to reported energies. IRC calculations were used to connect TS and subsequent intermediates. Single point calculations using the Def2TZVP[44] basis set on all atoms (i.e. BS4) were used to refine the calculated energies.

USCF ChimeraX[45-46] and the SEQCROW[47-48] plugin were used for analyses and image generation.

A data set collection of computational results is available in the ioChem-BD repository[49-50] and can be accessed via http://dx.doi.org/10.19061/iochem-bd-6-633.

Structural characterization of peptides used for *in vitro* studies

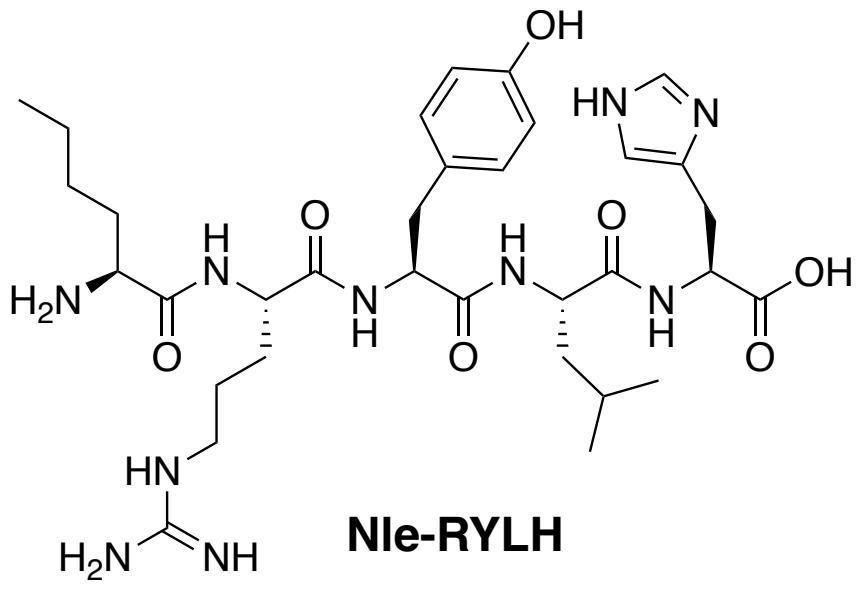

Nle-RYLH

Chemical Formula: $C_{33}H_{52}N_{10}O_7$
Exact Mass: 700.40204

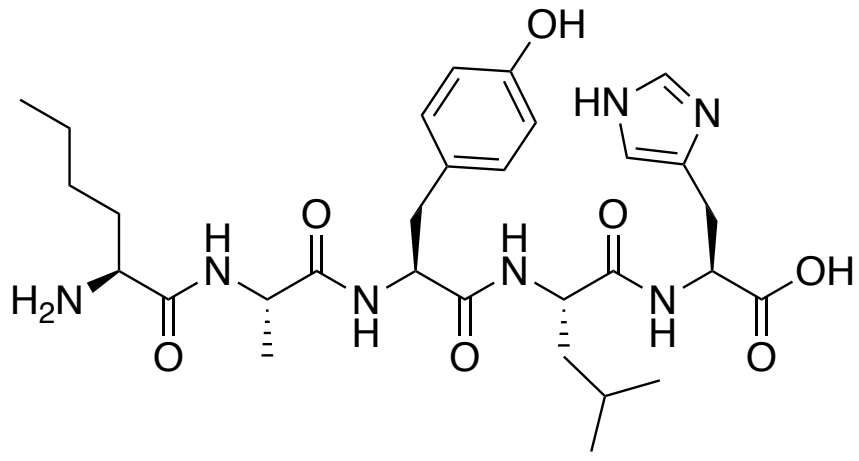

Nle-AYLH

Chemical Formula: $C_{30}H_{45}N_7O_7$
Exact Mass: 615.33805

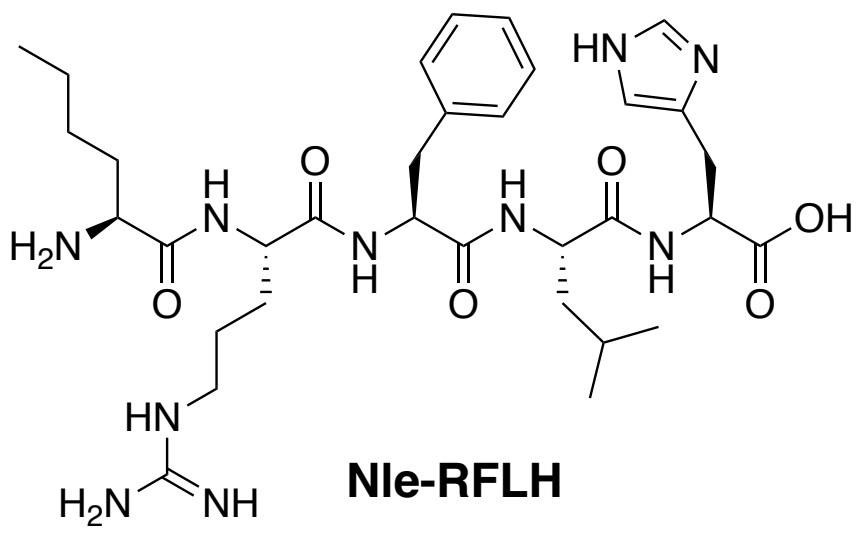

Nle-RFLH

Chemical Formula: $C_{33}H_{52}N_{10}O_6$
Exact Mass: 684.40713

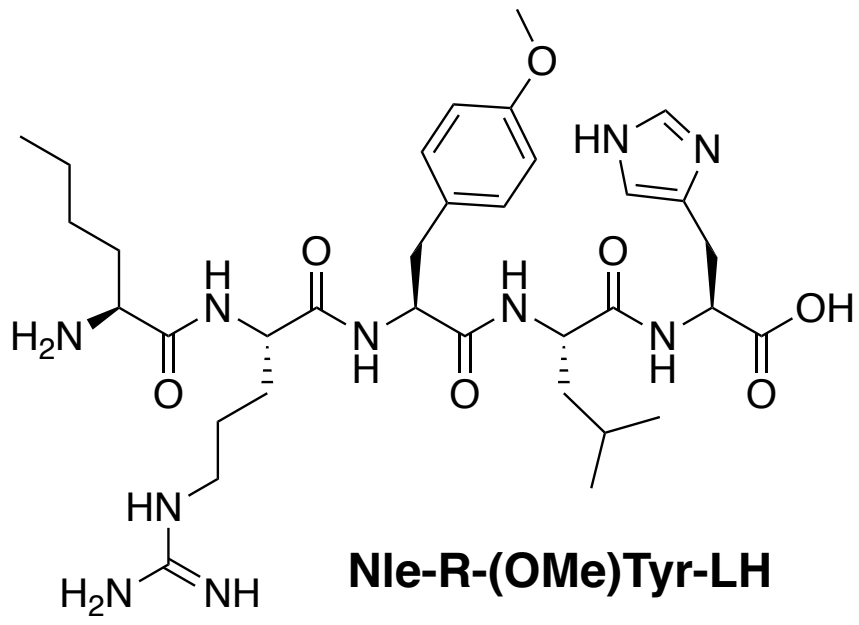

Nle-R-(OMe)Tyr-LH

Chemical Formula: $C_{34}H_{54}N_{10}O_7$
Exact Mass: 714.41769

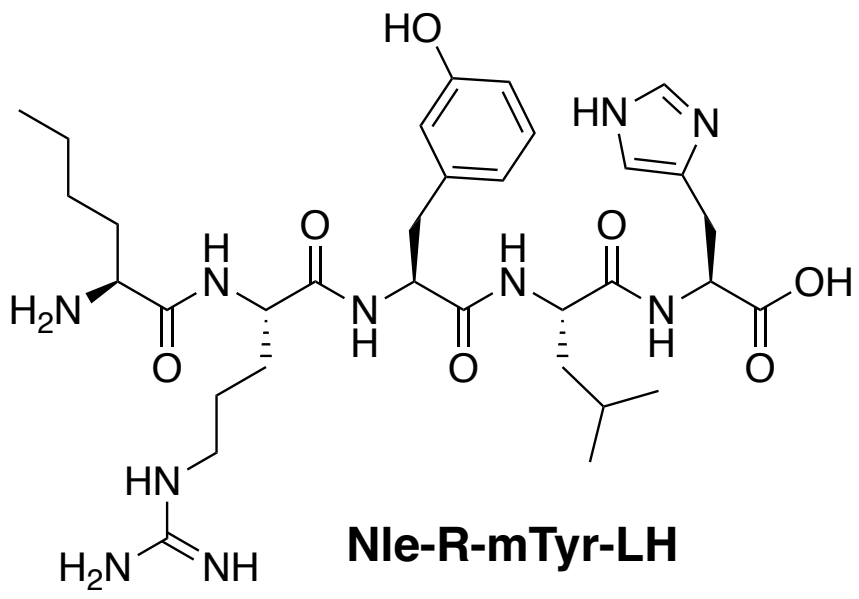

Nle-R-mTyr-LH

Chemical Formula: $C_{33}H_{52}N_{10}O_7$
Exact Mass: 700.40204

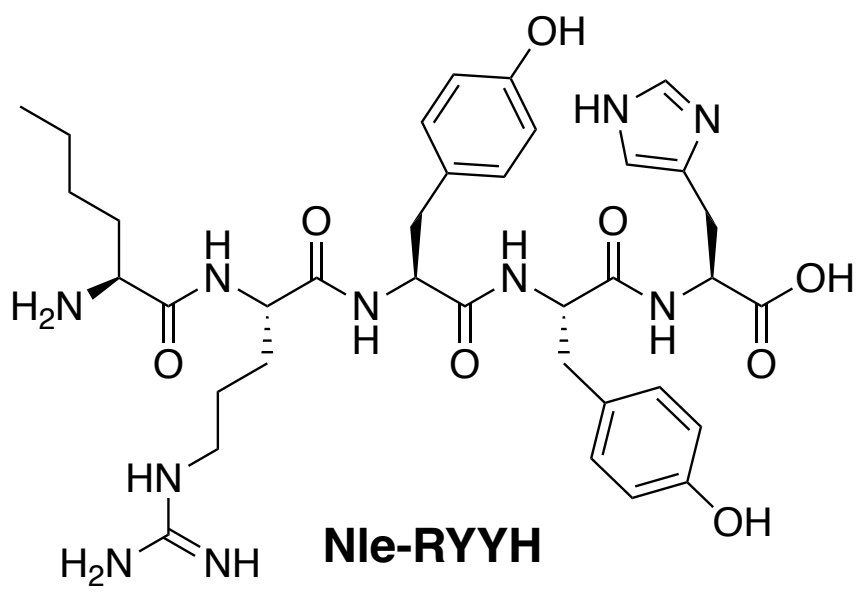

Nle-RYYH

Chemical Formula: $C_{36}H_{50}N_{10}O_8$
Exact Mass: 750.38131

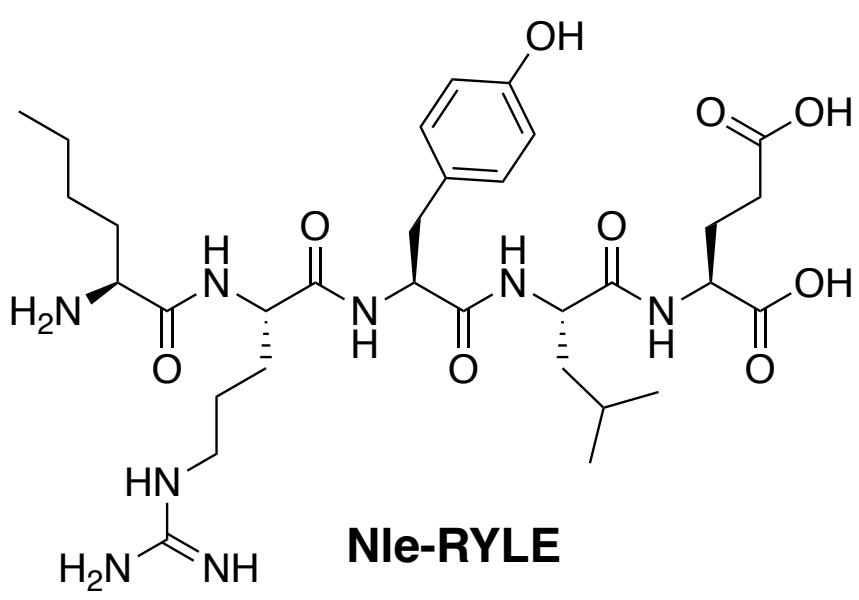

Nle-RYLE

Chemical Formula: $C_{32}H_{52}N_8O_9$
Exact Mass: 692.38573

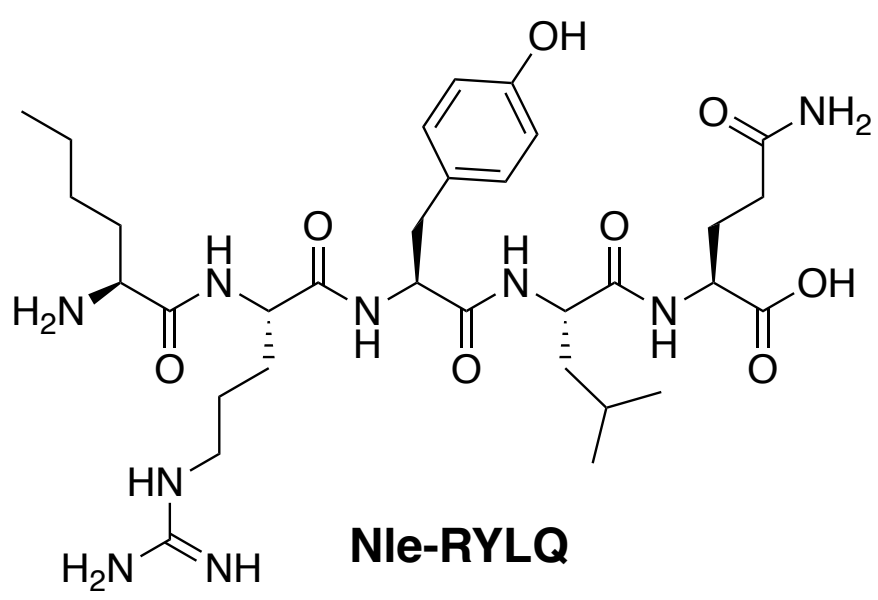

Nle-RYLQ

Chemical Formula: $C_{32}H_{53}N_9O_8$
Exact Mass: 691.40171

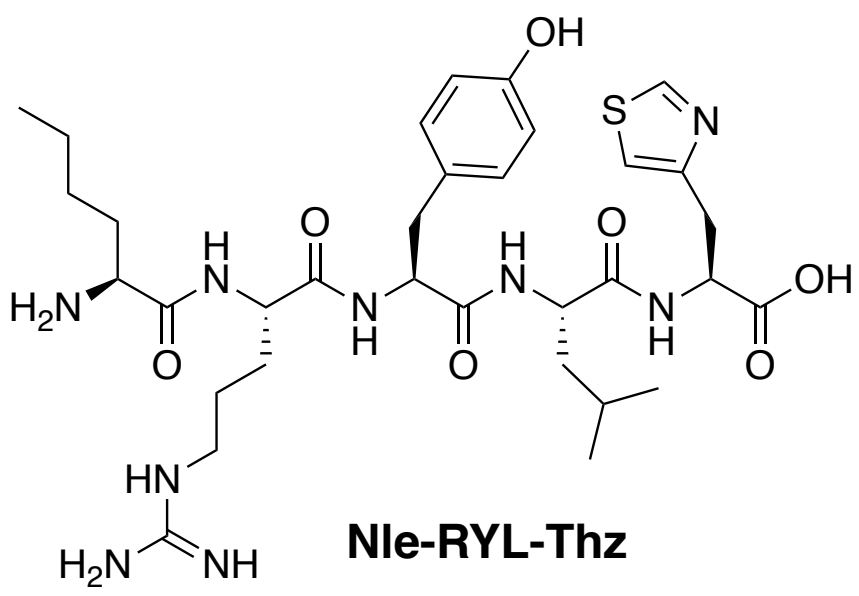

Nle-RYL-Thz

Chemical Formula: $C_{33}H_{51}N_9O_7S$
Exact Mass: 717.36322

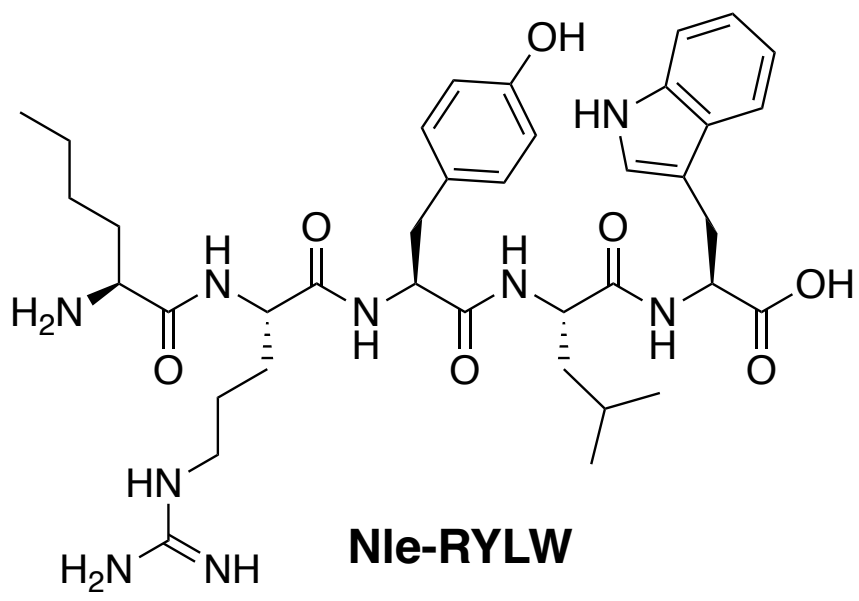

Nle-RYLW

Chemical Formula: $C_{38}H_{55}N_9O_7$
Exact Mass: 749.42245

Figure S1. Structures of peptides in this study.

Nle-RYLH. ^{1}H-NMR (400 MHz, DMSO-d_6): δ 8.61 (d, J = 8.0 Hz, 1 H), 8.38 (s, 1 H), 8.20 (d, J = 7.8 Hz, 1 H), 8.16 (d, J = 8.0 Hz, 1 H), 8.09 (d, J = 8.2 Hz, 1 H), 7.94 (bs, 1 H), 7.14 (s, 1 H), 7.00 (d, J = 8.5 Hz, 2 H), 6.60 (d, J = 8.5 Hz, 2 H), 4.42-4.49 (m, 2 H), 4.30-4.40 (m, 1 H), 4.21-4.26 (m, 1 H), 3.77 (t, J = 6.2 Hz, 1 H), 3.05-3.10 (m, 3 H), 2.86-2.99 (m, 2 H), 2.64-2.70 (m, 1 H), 1.60-1.66 (m, 3 H), 1.38-1.57 (m, 6 H), 1.21-1.25 (m, 4 H), 0.80-0.87 (m, 9 H). ^{13}C{^{1}H}-NMR (100 MHz, DMSO-d_6): δ 172.3, 171.9, 171.0, 170.6, 168.5, 156.9, 155.8, 134.0, 130.0, 127.6, 118.7, 115.7, 114.8, 54.0, 52.2, 51.7, 51.2, 40.7, 40.5, 36.6, 30.9, 29.4, 26.2, 24.7, 24.0, 23.0, 21.7, 21.6, 13.7. HRMS (ESI) m/z: [M+H]$^+$ Calcd. for $C_{33}H_{53}N_{10}O_7^+$ 701.4093; Found 701.4086; error 1.0 ppm.

Nle-AYLH. ^{1}H-NMR (400 MHz, DMSO-d_6): δ 8.53 (d, J = 7.6 Hz, 1 H), 8.47 (bs, 1 H), 8.27 (d, J = 7.9 Hz, 1 H), 8.02 (dd, J = 8.2 Hz, 2.5 Hz, 2 H), 7.18 (s, 1 H), 6.99 (d, J = 8.5 Hz, 2 H), 6.60 (d, J = 8.5 Hz, 2 H), 4.34-4.52 (m, 3 H), 4.28 (q, J = 7.8 Hz, 1 H), 3.73 (t, J = 6.6 Hz, 1 H), 3.07 (dd, J = 15.1 Hz, 5.5 Hz, 1 H), 2.93-2.99 (m, 1 H), 2.88 (dd, J = 14.3 Hz, 4.6 Hz, 1 H), 2.63-2.69 (m, 1 H), 1.54-1.62 (m, 3 H), 1.42 (t, J = 7.2 Hz, 2 H), 1.18-1.25 (m, 7 H), 0.81-0.88 (m, 9 H). ^{13}C{^{1}H}-NMR (100 MHz, DMSO-d_6): δ 172.1, 171.8, 171.5, 170.9, 168.1, 155.8, 134.1, 130.0, 127.6, 114.8, 53.9, 52.1, 51.4, 50.9, 48.0, 40.8, 30.9, 27.1, 26.2, 24.0, 23.0, 21.7, 21.6, 18.5, 13.7. HRMS (ESI) m/z: [M+H]$^+$ Calcd. for $C_{30}H_{46}N_7O_7^+$ 616.3453; Found 616.3458; error 0.8 ppm.

Nle-RFLH. ^{1}H-NMR (400 MHz, DMSO-d_6): δ 8.59 (s, 1 H), 8.29 (d, J = 8.0 Hz, 1 H), 8.25 (d, J = 8.0 Hz, 1 H), 8.16 (d, 8.4 Hz, 1 H), 7.98 (bs, 1 H), 7.16-7.25 (m, 6 H), 4.47-4.58 (M, 2 H), 4.22-4.32 (m, 2 H), 3.77 (t, J = 6.5 Hz, 1 H), 2.95-3.15 (m, 5 H), 2.77 (dd, J = 14.1 Hz, 9.9 Hz, 1 H), 1.38-1.63 (m, 9 H), 1.20-1.22 (m, 4 H), 0.87 (t, J = 6.5 Hz, 3 H), 0.78-0.82 (m, 6 H). ^{13}C{^{1}H}-NMR (100 MHz, DMSO-d_6): δ 172.2, 172.0, 170.9, 170.8, 168.5, 157.0, 137.7, 133.9, 130.4, 129.2, 127.9, 126.2, 118.6, 117.0, 115.6, 53.7, 52.2, 51.5, 51.3, 40.7, 40.5, 39.7, 37.4, 30.9, 29.4, 26.9, 26.2, 24.7, 24.1, 23.0, 22.2, 21.8, 21.6, 13.7. HRMS (ESI) m/z: [M+H]$^+$ Calcd. for $C_{33}H_{53}N_{10}O_6^+$ 685.4144; Found 685.4141; error 0.4 ppm.

Nle-R-(OMe)Tyr-LH. ^{1}H-NMR (400 MHz, DMSO-d_6): δ 8.58-8.63 (m, 1 H), 8.27 (d, J = 7.9 Hz, 1 H), 8.20 (d, J = 8.0 Hz, 1 H), 8.10 (d, J = 8.1 Hz, 1 H), 7.86 (bs, 1 H), 7.23 (s, 1 H), 7.15 (d, J = 8.5 Hz, 2 H), 6.76 (d, J = 8.7 Hz, 2 H), 4.47-4.53 (m, 2 H), 4.32 (bs, 1 H), 4.22-4.28 (m, 1 H), 3.77 (bs, 1 H), 3.69 (s, 3 H), 2.89-3.14 (m, 5 H), 2.67-2.73 (m, 1 H), 1.40-1.62 (m, 9 H), 1.19-1.22 (m, 4 H), 0.87 (d, J = 6.6 Hz, 3 H), 0.80 (m, 6 H). ^{13}C{^{1}H}-NMR (100 MHz, DMSO-d_6): δ 172.2, 171.9, 170.9, 170.7, 168.4, 157.8, 156.8, 133.9, 130.2, 129.4, 115.7, 113.3, 54.9, 53.9, 52.2, 51.4, 51.2, 40.7, 40.5, 30.9, 29.4, 26.9, 26.2, 24.7, 24.0, 23.0, 21.7, 21.6, 13.7. HRMS (ESI) m/z: [M+H]$^+$ Calcd. for $C_{34}H_{55}N_{10}O_7^+$ 715.4250; Found 715.4250; error 0.0 ppm.

Nle-R-mTyr-LH. ^{1}H-NMR (400 MHz, DMSO-d_6): δ 8.62 (bs, 1 H), 8.28 (bs, 1 H), 8.13-8.20 (m, 3 H), 7.11 (s, 1 H), 6.98 (t, J = 7.8 Hz, 1 H), 6.64-6.67 (m, 2 H), 6.56 (dd, J = 8.0 Hz, 2.5 Hz, 1 H), 4.49-4.55 (m, 1 H), 4.43 (q, J = 5.4 Hz, 1 H), 4.32 (bs, 1 H), 4.23 (q, J = 6.4 Hz, 1 H), 3.77 (bs, 1 H), 3.04-3.09 (m, 3 H), 2.90-2.98 (m, 2 H), 2.66 (dd, J = 14.3 Hz, 10.4 Hz, 1 H), 1.41-1.63 (m, 9 H), 1.21-1.25 (m, 4 H), 0.87 (d, J = 6.6 Hz, 3 H), 0.80-0.83 (m, 6 H). ^{13}C{^{1}H}-NMR (100 MHz, DMSO-d_6): δ 172.4, 171.8, 171.0, 170.6, 168.5, 157.1, 156.8, 138.9, 134.1, 128.8, 119.6, 118.7, 116.2, 113.2, 53.8, 52.2, 51.7, 51.3, 40.7, 40.5, 30.9, 26.2, 24.6, 24.0, 23.0, 21.8, 21.6, 13.7. HRMS (ESI) m/z: [M+H]$^+$ Calcd. for $C_{33}H_{53}N_{10}O_7^+$ 701.4093; Found 701.4089; error 0.6 ppm.

Nle-RYYH. ^{1}H-NMR (400 MHz, DMSO-d_6): δ 8.61 (d, J = 6.5 Hz, 1 H), 8.27 (bs, 2 H), 8.20 (d, J = 7.8 Hz, 1 H), 8.14 (d, J = 8.9 Hz, 1 H), 7.26 (bs, 3 H), 7.12 (s, 1 H), 7.03 (d, J = 8.5 Hz, 2 H), 6.88 (d, J = 8.5 Hz, 2 H), 6.64 (d, J = 8.5 Hz, 2 H), 6.55 (d, J = 8.5 Hz, 2 H), 4.30-4.46 (m, 5 H), 3.76 (t, J = 5.5 Hz, 1 H), 2.92-3.11 (m, 5 H), 2.84-2.87 (m, 1 H), 2.68-2.78 (m, 3 H), 2.56-2.62 (m, 1 H), 1.63-1.66 (m, 3 H), 1.43-1.55 (m, 3 H), 1.20-1.23 (m, 4 H), 0.80 (t, J = 6.7 Hz, 3 H). ^{13}C{^{1}H}-NMR (100 MHz, DMSO-d_6): δ 172.6, 171.1, 171.0, 170.5, 168.5, 156.9, 155.9, 155.7, 134.1, 130.2, 129.9, 127.7, 127.6, 118.7, 115.7, 114.9, 114.8, 54.8, 54.4, 52.2, 43.7, 40.4, 36.6, 30.9, 29.3, 27.7, 26.2, 24.7, 22.2, 21.7, 13.7.HRMS (ESI) m/z: [M+H]$^+$ Calcd. for $C_{36}H_{50}N_{10}O_8^+$ 751.3886; Found 751.3877; error 1.2 ppm.

Nle-RYLE. ^{1}H-NMR (400 MHz, DMSO-d_6): δ 8.65 (d, J = 8.8 Hz, 1 H), 8.09 (d, J = 8.2 Hz, 2 H), 7.96 (d, J = 9.4 Hz, 1 H), 7.30 (bs, 2 H), 6.99 (d, J = 8.5 Hz, 2 H), 6.61 (d, J = 8.5 Hz, 2 H), 4.45 (q, J = 4.8 Hz, 1 H), 4.33 (q, J = 5.5 Hz, 1 H), 4.25 (q, J = 7.6 Hz, 1H), 3.12 (q, J = 5.0 Hz, 1 H), 3.77 (t, J = 6.2 Hz, 1 H), 3.06 (bs, 2 H), 2.85-2.90 (m, 1 H), 2.68-2.74 (m, 1 H), 2.20-2.30 (m, 2 H), 1.93-1.97 (m, 1 H), 1.77-1.83 (m, 1 H), 1.60-1.68 (m, 2 H), 1.50-

1.58 (m, 3 H), 1.46 (t, *J* = 7.3 Hz, 3 H), 1.22-1.25 (m, 4 H), 0.87 (d, *J* = 6.5 Hz, 3 H), 0.82 (t, *J* = 6.6 Hz, 6 H). $^{13}C\{^{1}H\}$-NMR (100 MHz, DMSO-d_6): δ 174.0, 173.4, 171.8, 170.8, 170.6, 168.6, 156.9, 155.8, 127.5, 114.8, 54.2, 52.2, 52.2, 51.6, 51.2, 40.8, 40.5, 36.7, 35.8, 30.9, 30.1, 29.3, 26.6, 26.2, 24.5, 24.0, 23.1, 21.8, 21.6, 13.7. HRMS (ESI) *m/z*: $[M+H]^+$ Calcd. for $C_{32}H_{53}N_8O_9^+$ 693.3930; Found 693.3935; error 0.7 ppm.

Nle-RYLQ. ^{1}H-NMR (400 MHz, DMSO-d_6): δ 8.76 (d, *J* = 6.6 Hz, 1 H), 8.17 (d, *J* = 8.9 Hz, 1 H), 8.09 (d, *J* = 8.5 Hz, 1 H), 7.79 (bs, 1 H), 7.29 (bs, 1 H), 6.98 (d, *J* = 8.5 Hz, 2 H), 6.73 (bs, 1 H), 6.61 (d, *J* = 8.5 Hz, 2 H), 4.45 (q, *J* = 5.5 Hz, 1 H), 4.28-4.36 (m ,1 H), 4.14-4.18 (m, 1H), 3.98 (q, *J* = 5.4 Hz, 1 H), 3.75 (t, *J* = 6.5 Hz, 1 H), 2.99-3.04 (m, 3 H), 2.83-2.89 (m, 1 H), 2.71-2.76 (m, 1 H), 1.88-2.13 (m, 3 H), 1.75-1.82 (m, 2 H), 1.60-1.68 (m, 2 H), 1.44-1.55 (m, 6 H), 1.22-1.25 (m, 4 H), 0.87 (d, *J* = 6.2 Hz, 3 H), 0.81 (t, *J* = 6.5 Hz, 6 H). $^{13}C\{^{1}H\}$-NMR (100 MHz, DMSO-d_6): δ 175.5, 174.2, 174.0, 171.4, 170.7, 170.6, 168.8, 157.1, 155.9, 130.0, 127.3, 114.8, 54.6, 52.4, 52.3, 51.6, 43.7, 40.8, 37.0, 31.5, 31.0, 29.0, 27.7, 26.3, 24.3, 24.0, 23.1, 22.2, 21.8, 21.4, 13.7. HRMS (ESI) *m/z*: $[M+H]^+$ Calcd. for $C_{32}H_{54}N_9O_8^+$ 692.4090; Found 692.4102; error 1.7 ppm.

Nle-RYL-Thz. ^{1}H-NMR (600 MHz, DMSO-d_6): δ 8.99 (d, *J* = 2.0 Hz, 1 H), 8.53 (d, *J* = 8.2 Hz, 1 H), 8.16 (d, *J* = 7.8 Hz, 1 H), 8.08-8.12 (m, 4 H), 8.03 (d, *J* = 8.1 Hz, 1 H), 7.72 (t, *J* = 5.7 Hz, 1 H), 7.35 (d, *J* = 2.0 Hz, 1 H), 6.97 (d, *J* = 8.6 Hz, 2 H), 6.60 (d, *J* = 8.5 Hz, 2 H), 4.56-4.60 (m, 1 H), 4.44-4.48 (m, 1 H), 4.34-4.37 (m, 1 H), 4.27-4.31 (m, 1 H), 3.76 (q, *J* = 5.6 Hz, 2 H), 3.24 (dd, *J* = 15.1 Hz, 5.2 Hz, 1 H), 3.11-3.15 (m, 1 H), 3.07 (q, *J* = 5.9 Hz, 2 H), 2.85-2.88 (m, 1 H), 2.65 (dd, *J* = 14.3 Hz, 9.1 Hz, 1 H), 1.60-1.65 (m, 3 H), 1.54-1.59 (m, 1 H), 1.38-1.52 (m, 5 H), 1.21-1.24 (m, 4 H), 0.86 (d, *J* = 6.6 Hz, 3 H), 0.80-0.82 (m, 6 H). $^{13}C\{^{1}H\}$-NMR (150 MHz, DMSO-d_6): δ 172.5, 171.8, 170.8, 170.6, 168.4, 156.9, 155.8, 153.6, 152.9, 130.0, 127.5, 115.6, 114.8, 53.8, 52.1, 52.1, 51.8, 50.9, 41.0, 40.5, 36.6, 32.4, 30.9, 29.5, 26.2, 24.9, 24.0, 23.0, 21.7, 21.7, 13.7. HRMS (ESI) *m/z*: $[M+H]^+$ Calcd. for $C_{33}H_{52}N_9O_7S^+$ 718.3705; Found 718.3705; error 0.0 ppm.

Nle-RYLW. ^{1}H-NMR (400 MHz, DMSO-d_6): δ 10.85 (s, 1 H), 9.15 (s, 1 H), 8.53 (d, *J* = 8.2 Hz, 1 H), 8.04-8.09 (m, 5 H), 7.60 (bs, 1 H), 7.52 (d, *J* = 7.9 Hz, 1 H), 7.32 (d, *J* = 8.0 Hz, 1 H), 7.13 (d, *J* = 2.5 Hz, 1 H), 7.05 (t, *J* = 8.2 Hz, 1 H), 695-6.99 (m, 3 H), 6.59 (d, *J* = 8.5 Hz, 2 H), 4.43-4.50 (m, 2 H), 4.31-4.38 (m, 2 H), 3.75 (bs, 1 H), 3.14-3.20 (m, 1 H), 3.05-3.10 (m, 3 H), 2.86 (dd, *J* = 14.7 Hz, 3.6 Hz, 1 H), 2.63-2.69 (m, 1 H), 1.56-1.64 (m, 4 H), 1.38-1.50 (m, 5 H), 0.87 (d, *J* = 6.6 Hz, 3 H), 0.80-0.83 (m, 6 H). $^{13}C\{^{1}H\}$-NMR (100 MHz, DMSO-d_6): δ 173.1, 171.9, 170.7, 170.6, 168.4, 158.4, 158.1, 156.8, 155.8, 136.1, 130.0, 127.6, 127.3, 123.5, 120.9, 118.7, 118.4, 118.2, 114.8, 111.3, 109.6, 92.4, 53.8, 52.9, 52.1, 51.0, 41.0, 40.5, 36.5, 30.9, 29.6, 27.1, 26.2, 24.8, 24.1, 23.1, 21.7, 21.6 ,13.7. HRMS (ESI) *m/z*: $[M+H]^+$ Calcd. for $C_{38}H_{56}N_9O_7^+$ 750.4297; Found 750.4294; error 0.5 ppm.

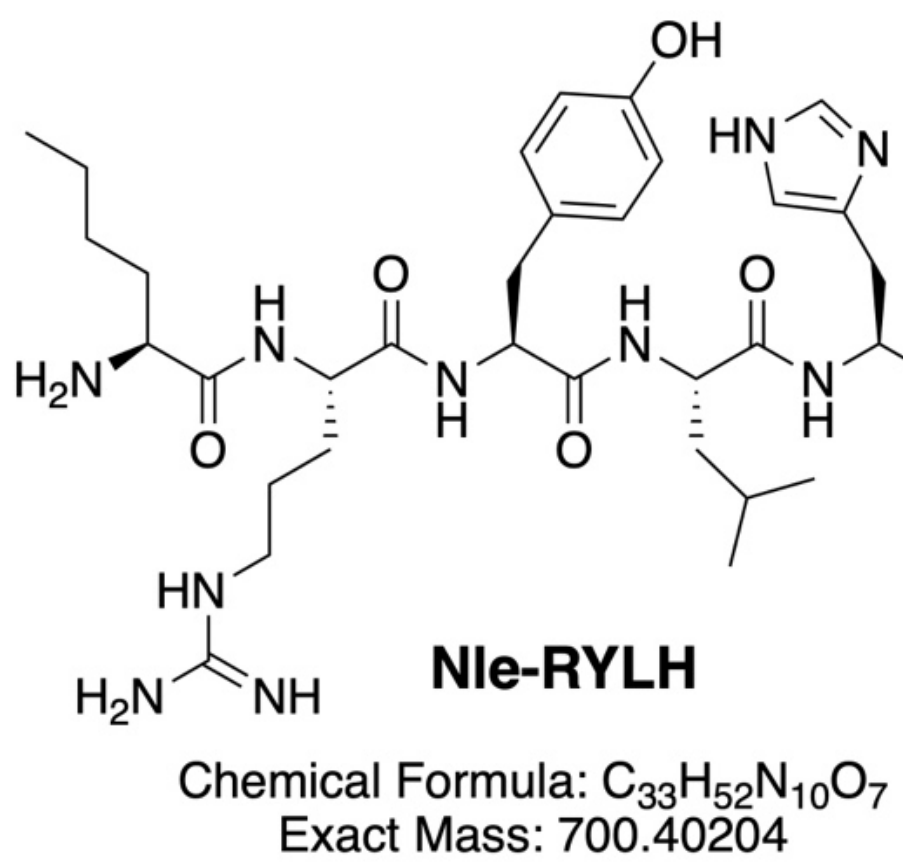

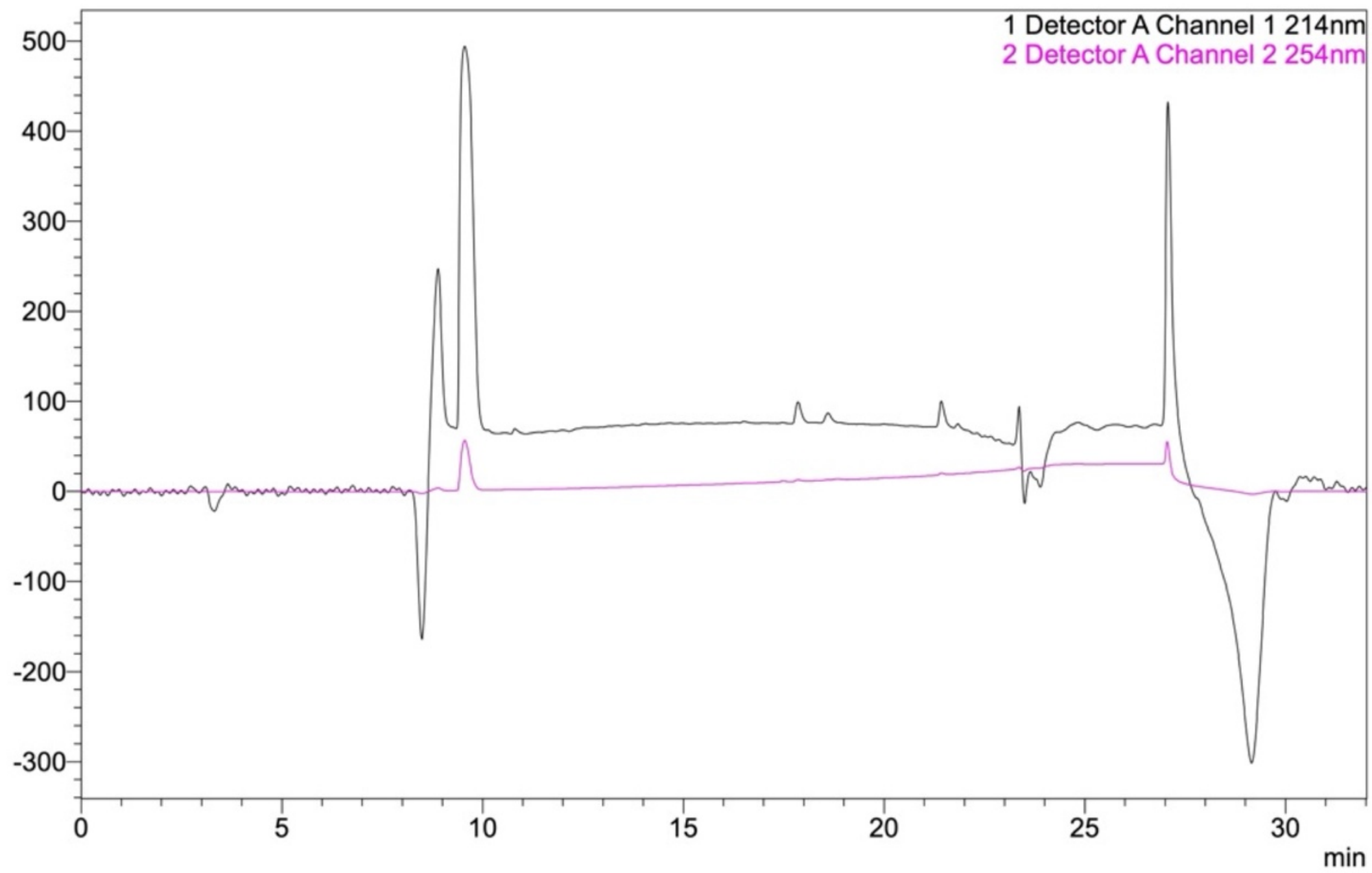

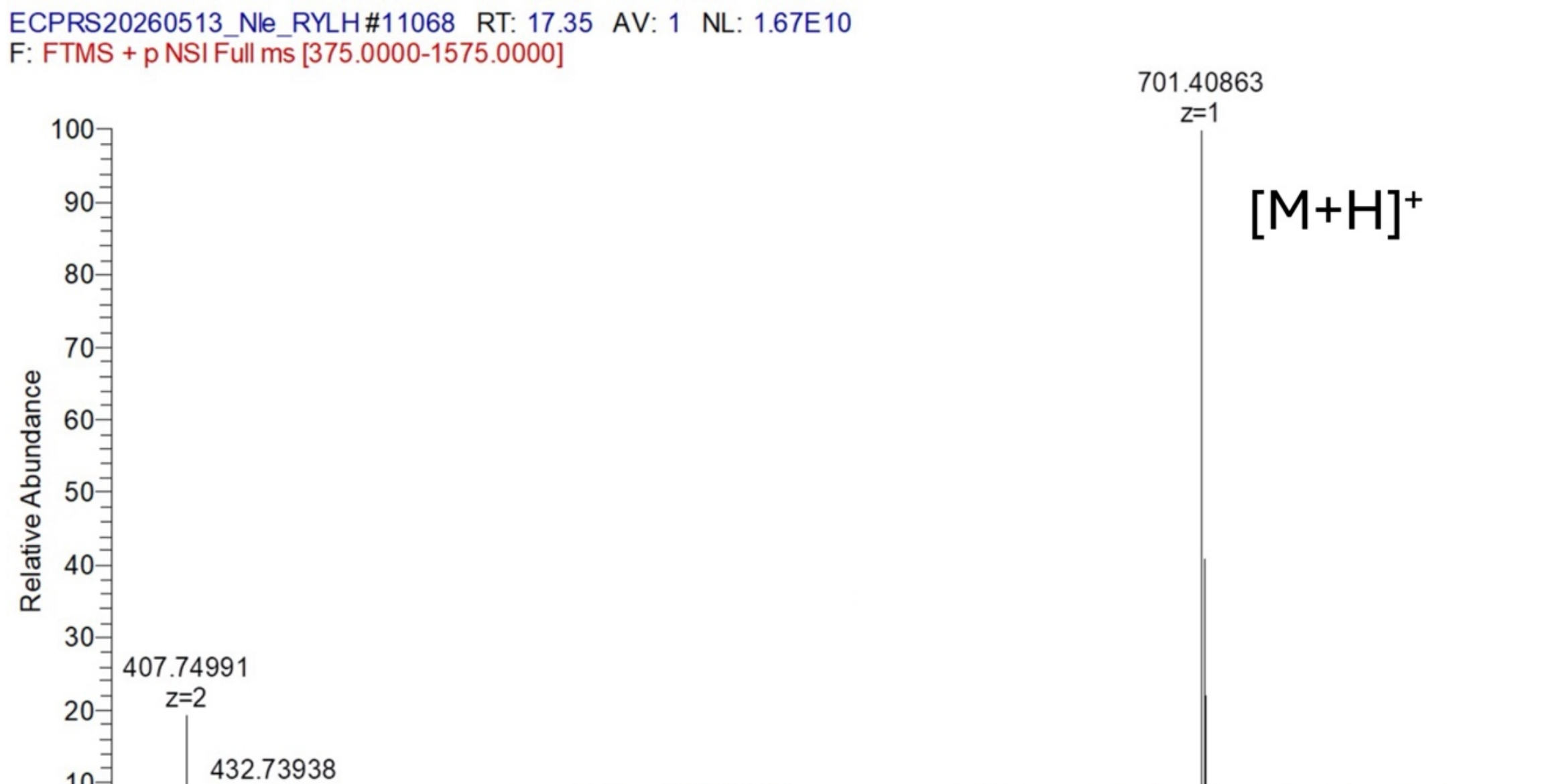

Figure S2. LC-MS characterization of Nle-RYLH.

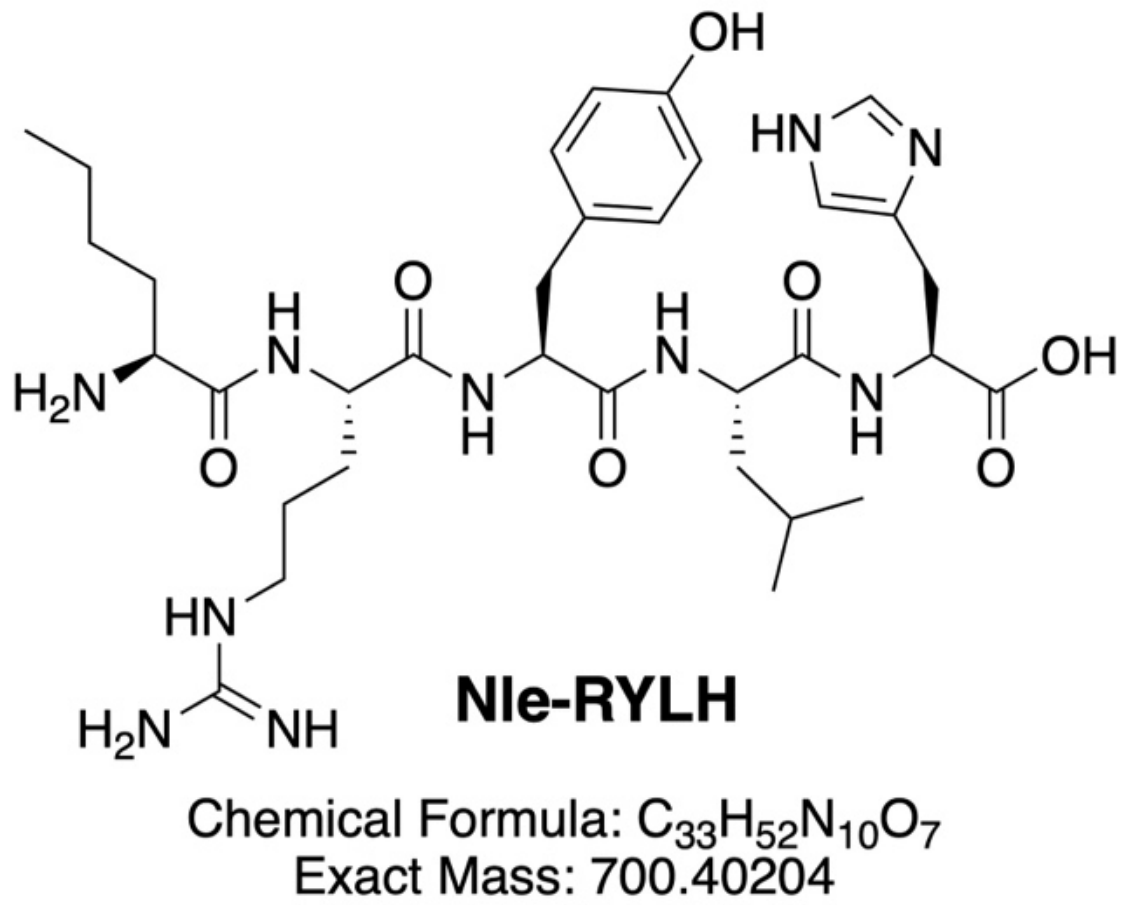

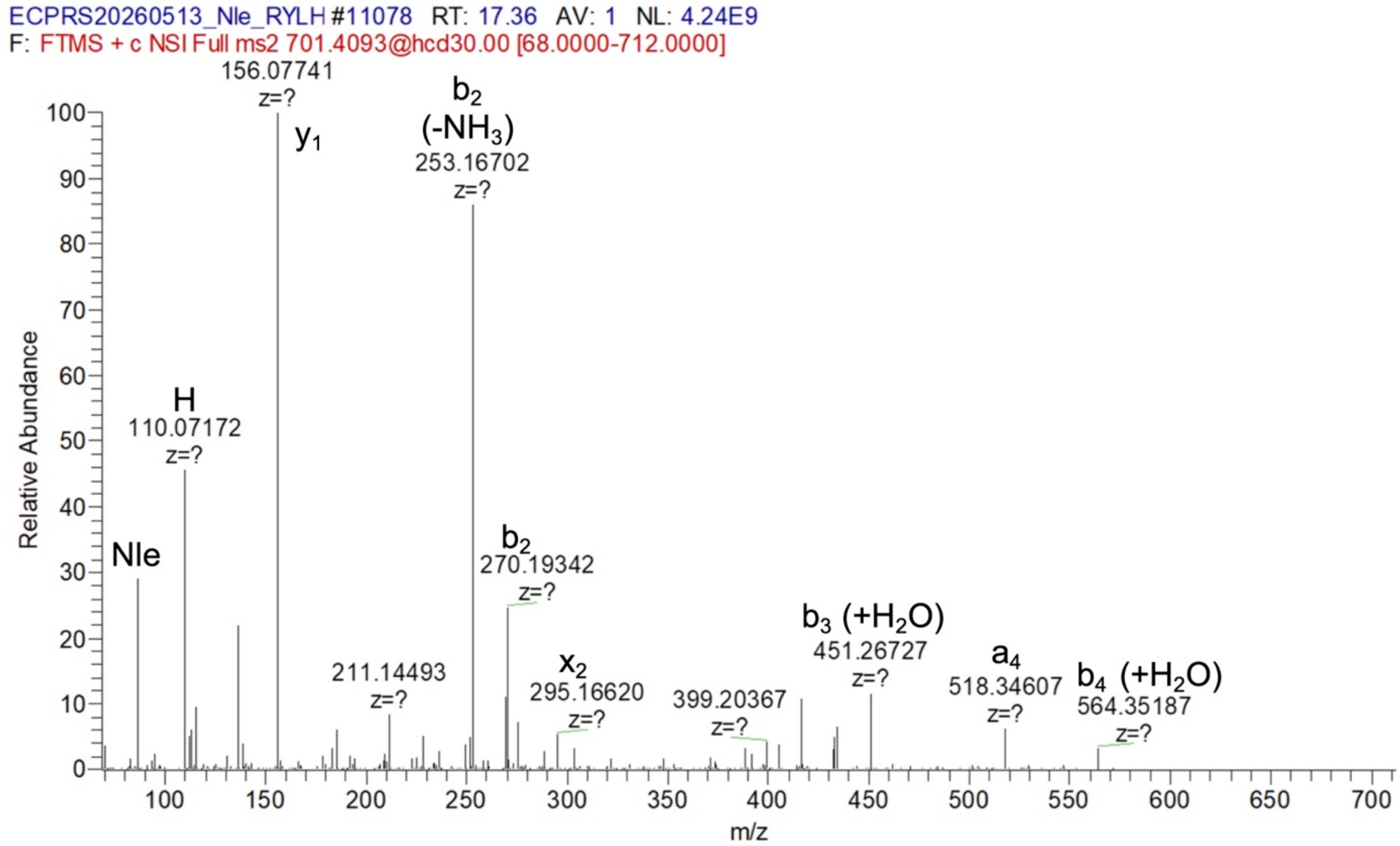

Figure S3. Annotated MS^2 spectrum of Nle-RYLH.

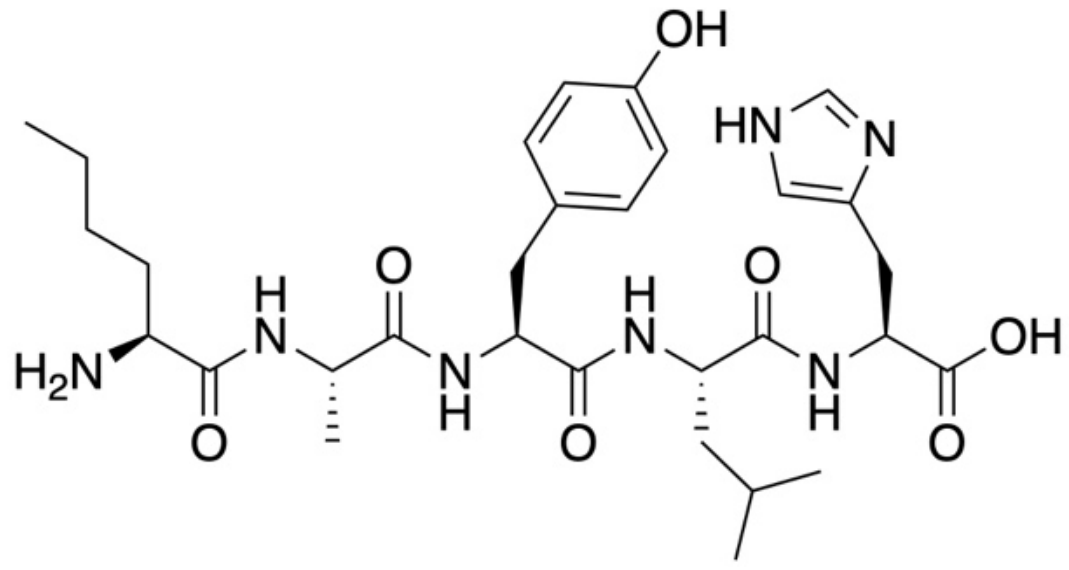

Nle-AYLH

Chemical Formula: $C_{30}H_{45}N_7O_7$
Exact Mass: 615.33805

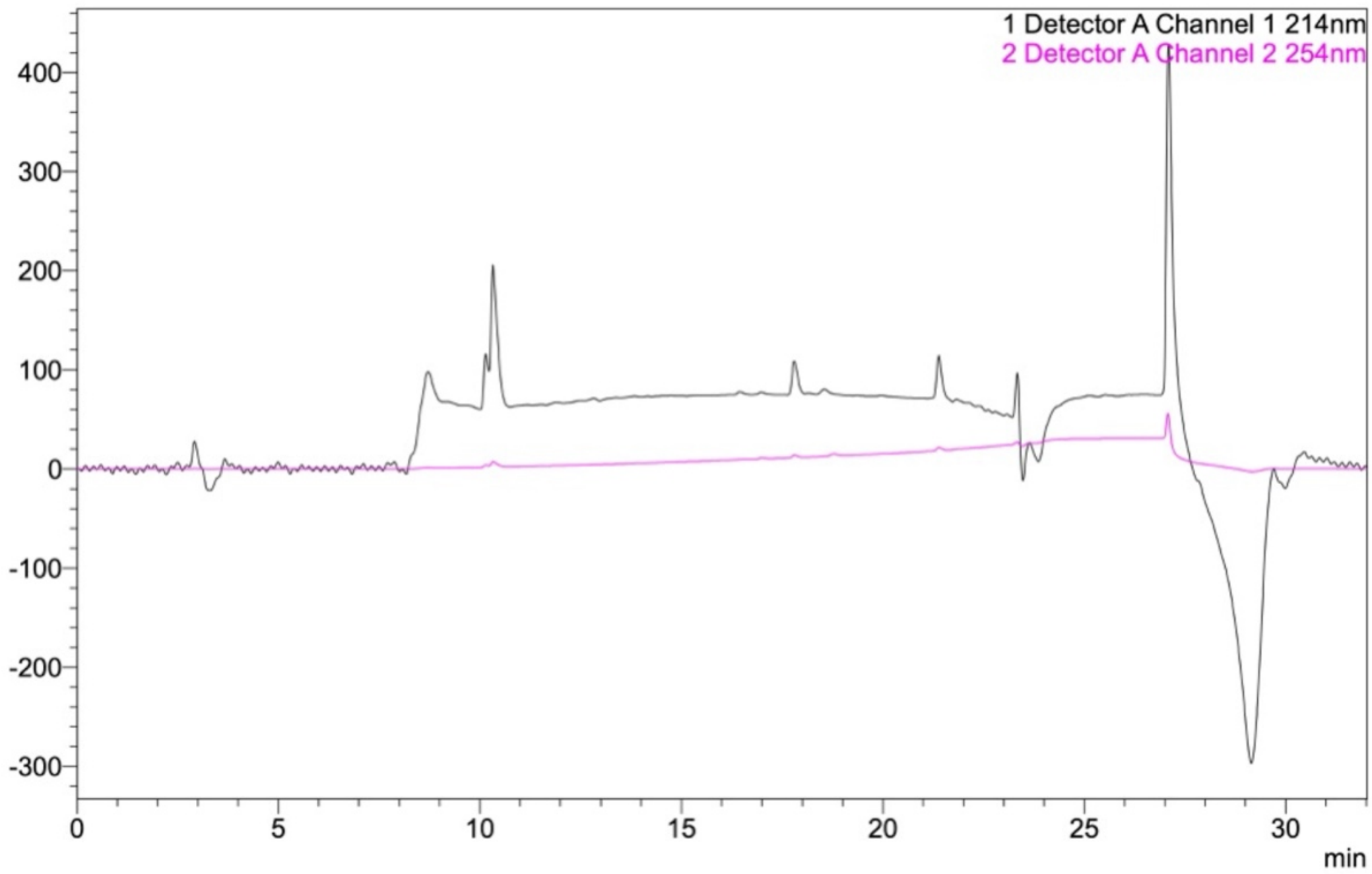

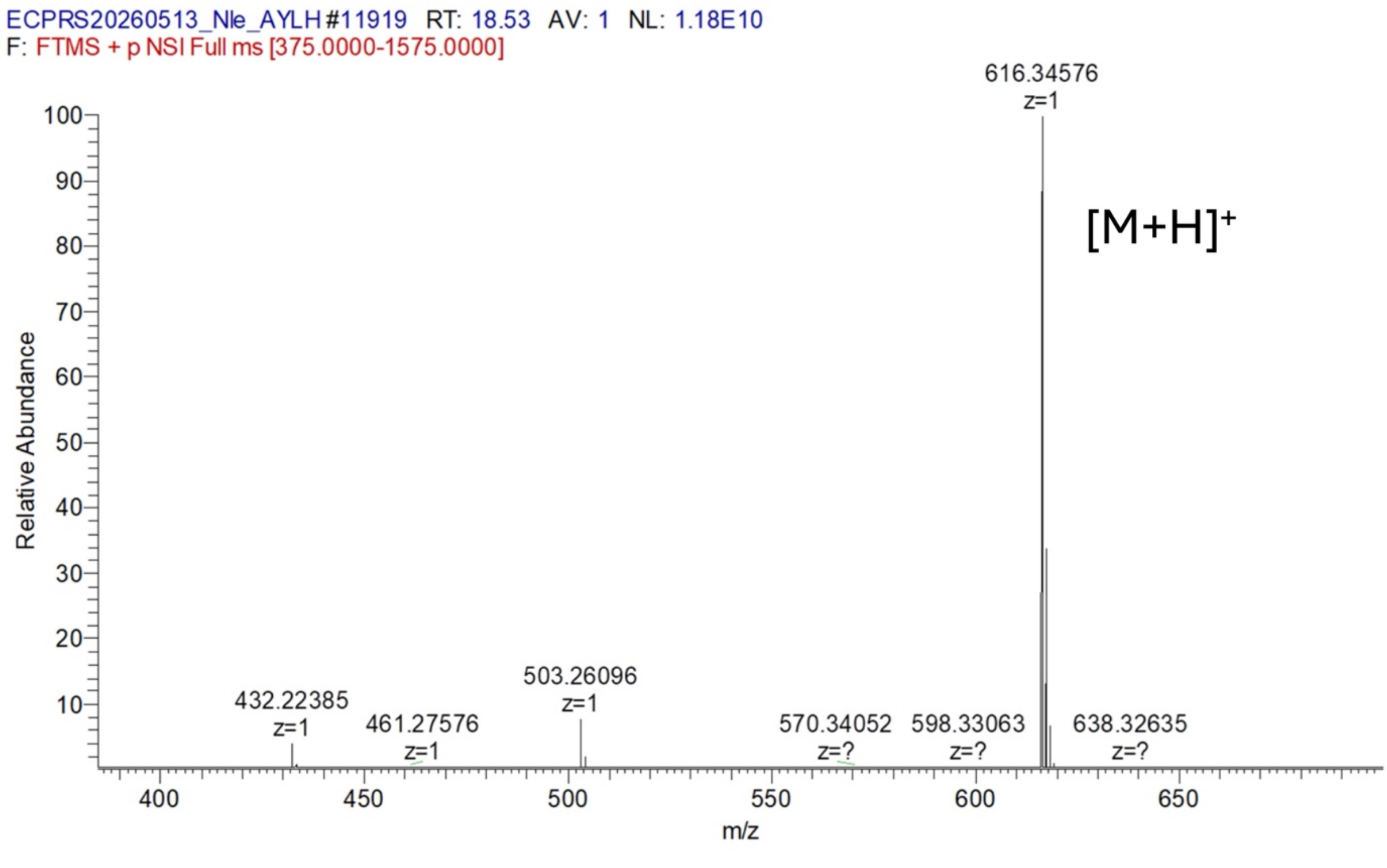

Figure S4. LC-MS characterization of Nle-**A**YLH.

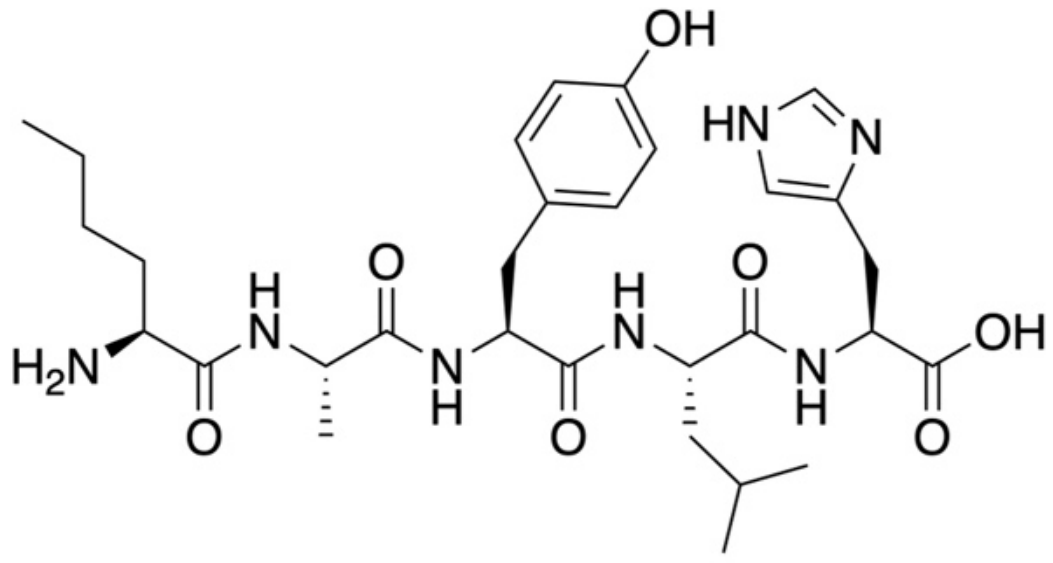

Nle-AYLH

Chemical Formula: $C_{30}H_{45}N_7O_7$
Exact Mass: 615.33805

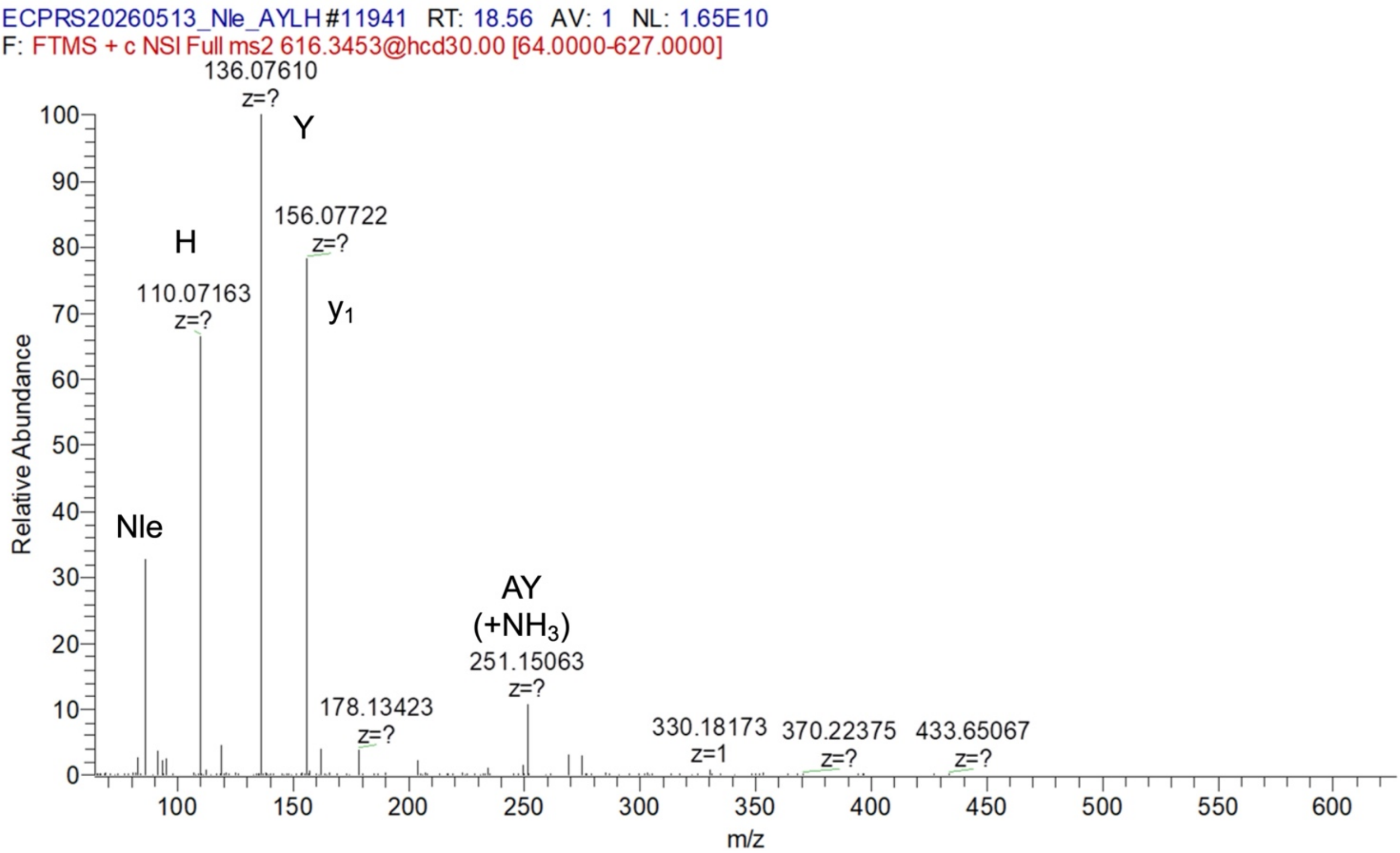

Figure S5. Annotated MS^2 spectrum of Nle-**A**YLH.

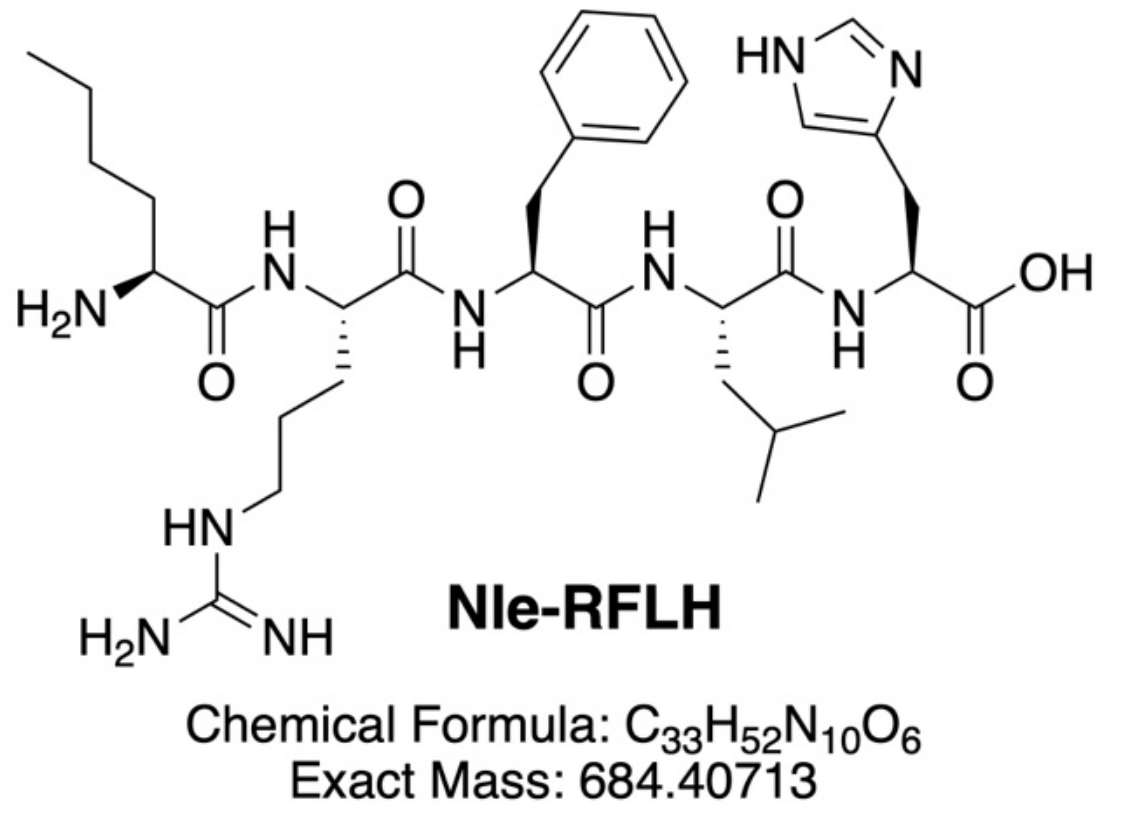

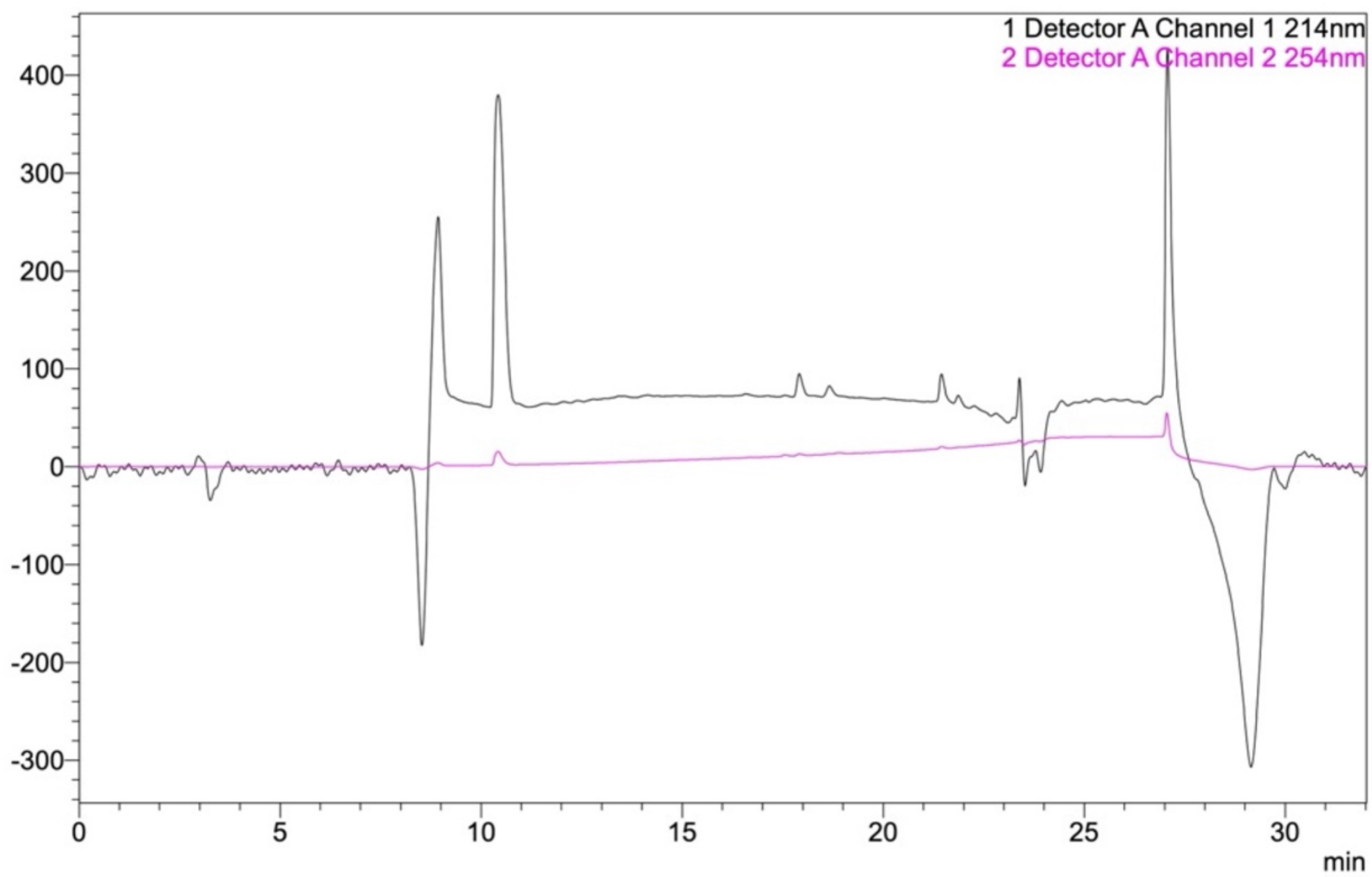

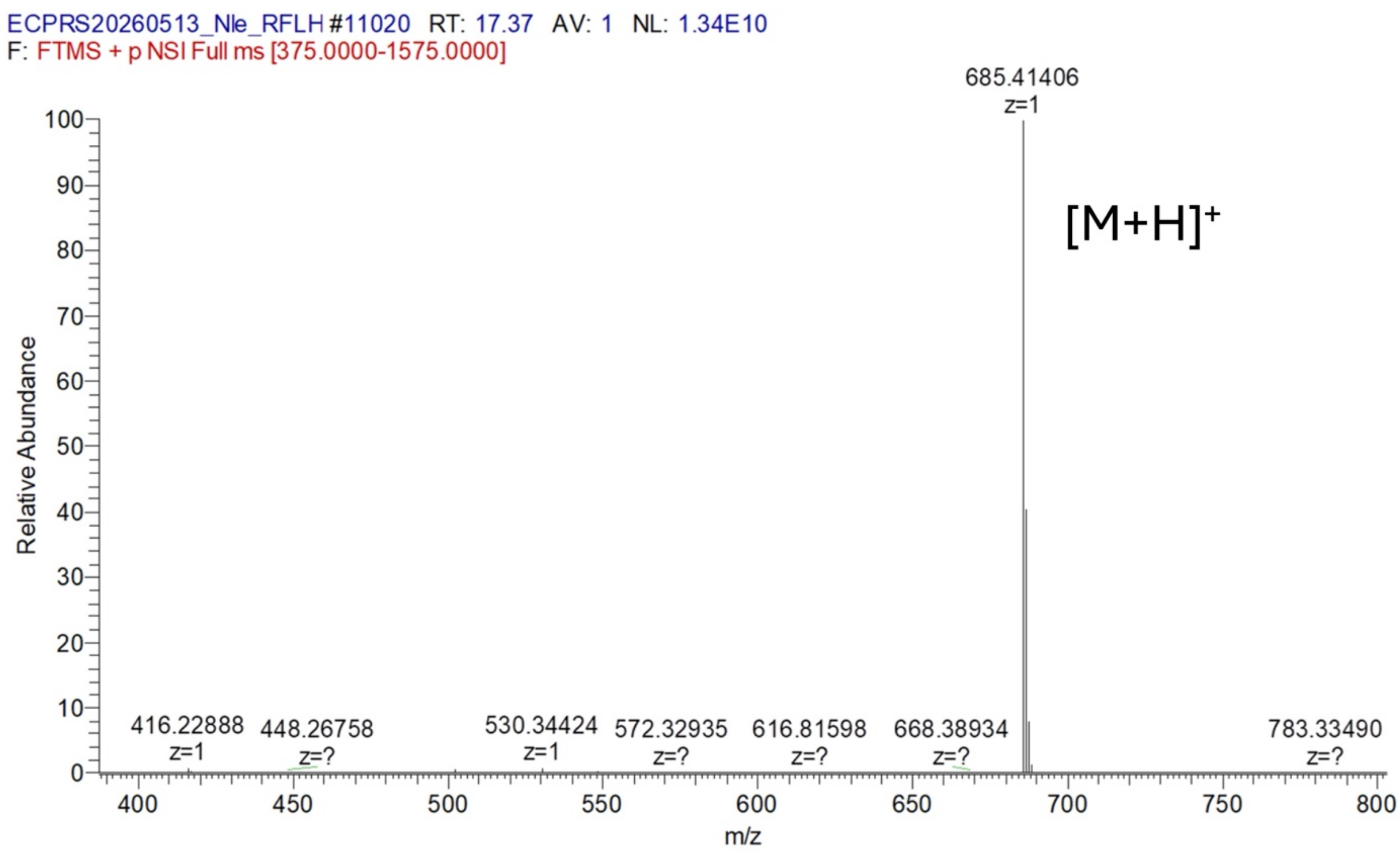

Figure S6. LC-MS characterization of Nle-R**F**LH.

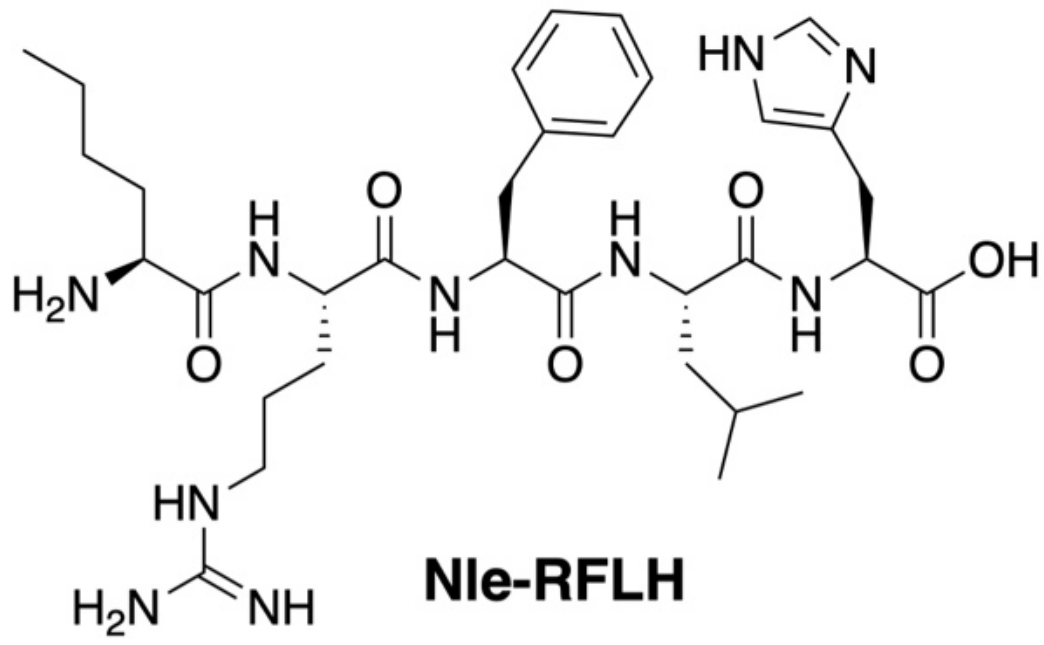

Chemical Formula: $C_{33}H_{52}N_{10}O_6$
Exact Mass: 684.40713

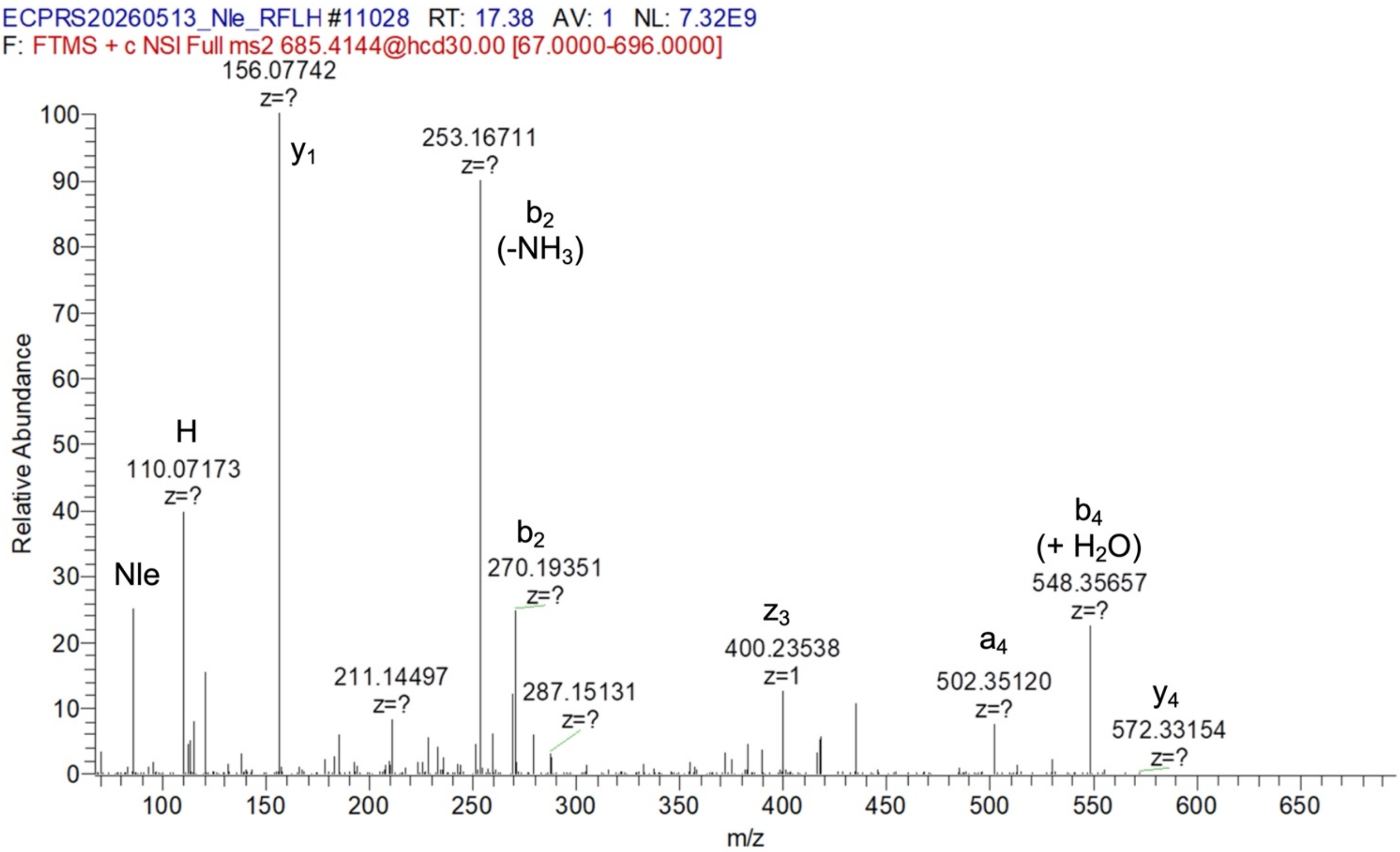

Figure S7. Annotated MS^2 spectrum of Nle-R**F**LH.

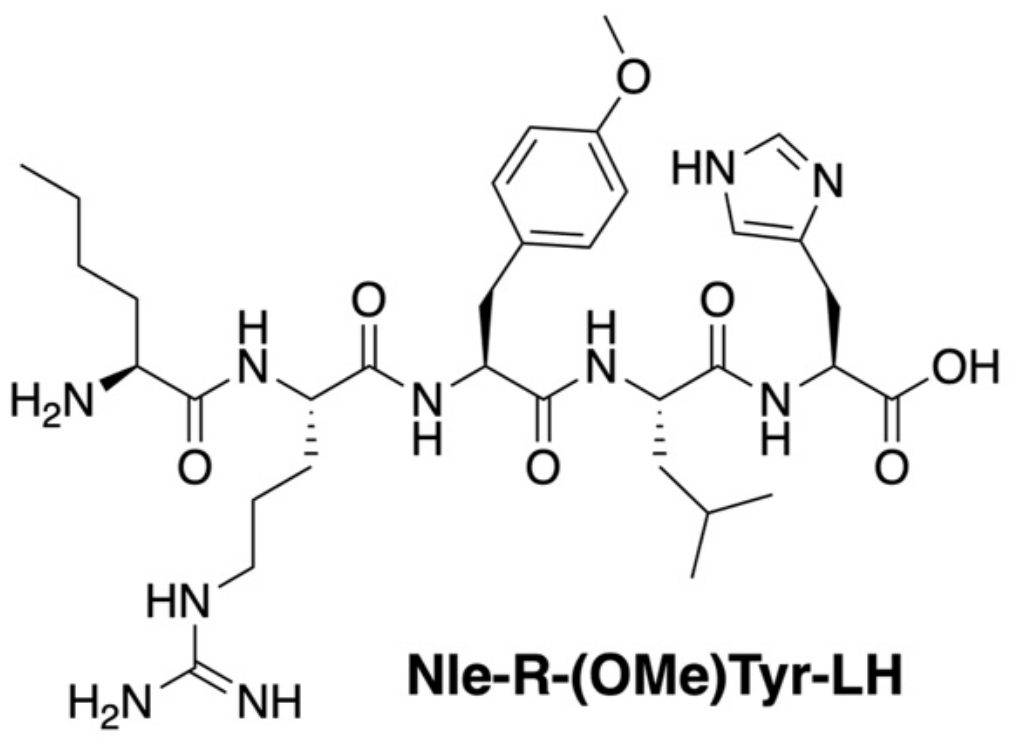

Nle-R-(OMe)Tyr-LH

Chemical Formula: $C_{34}H_{54}N_{10}O_7$
Exact Mass: 714.41769

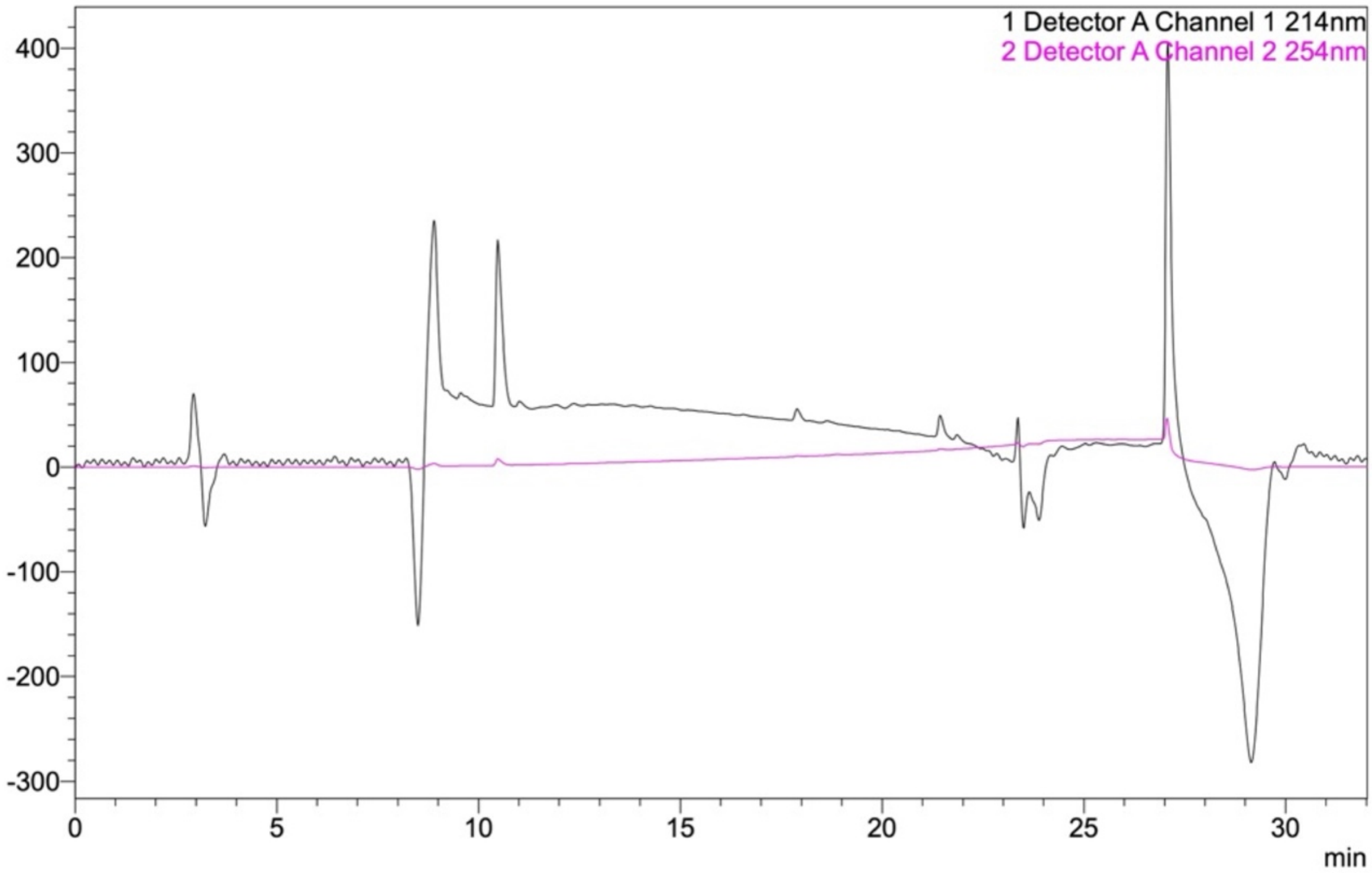

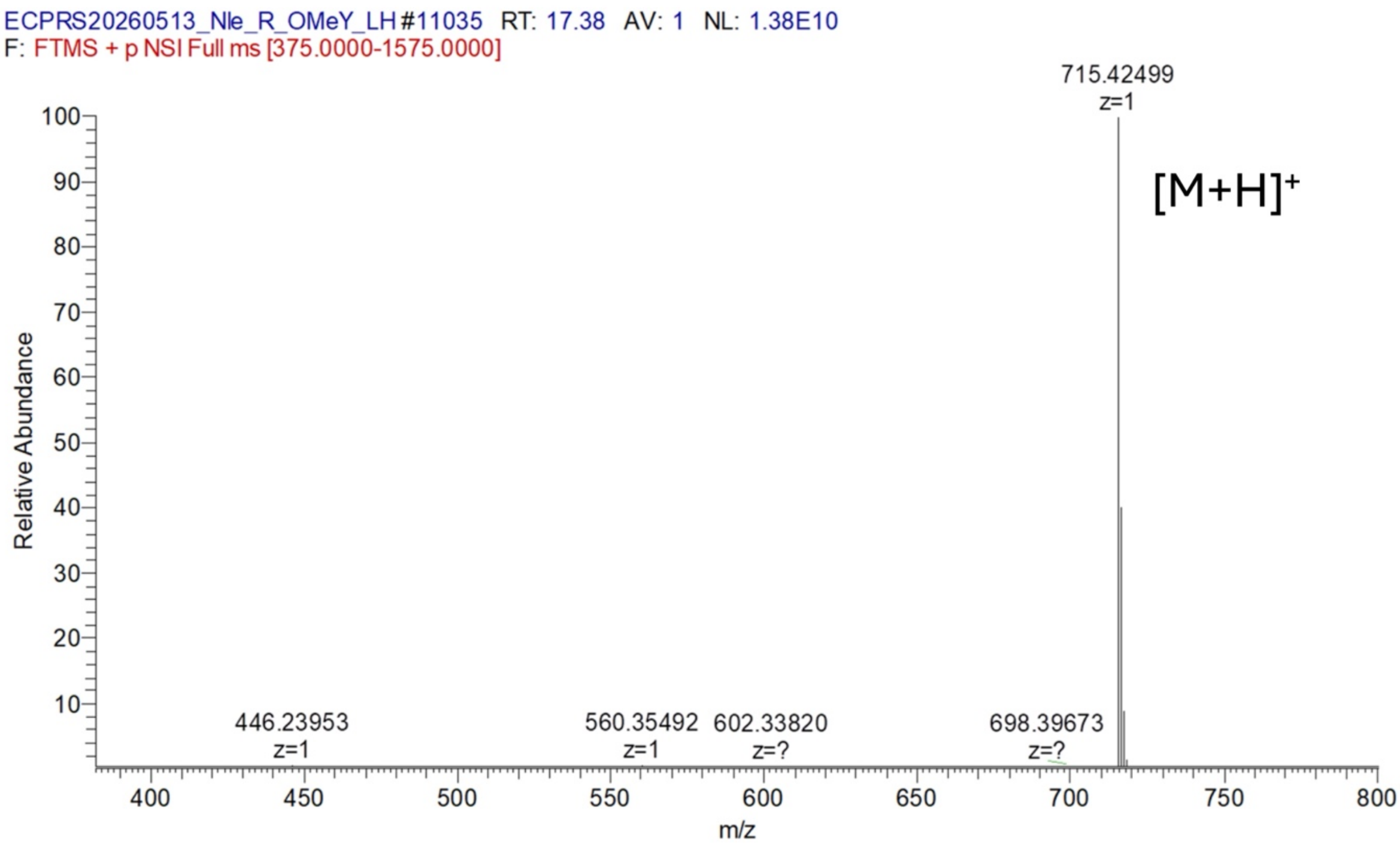

Figure S8. LC-MS characterization of Nle-R-**(OMe)Tyr**-LH.

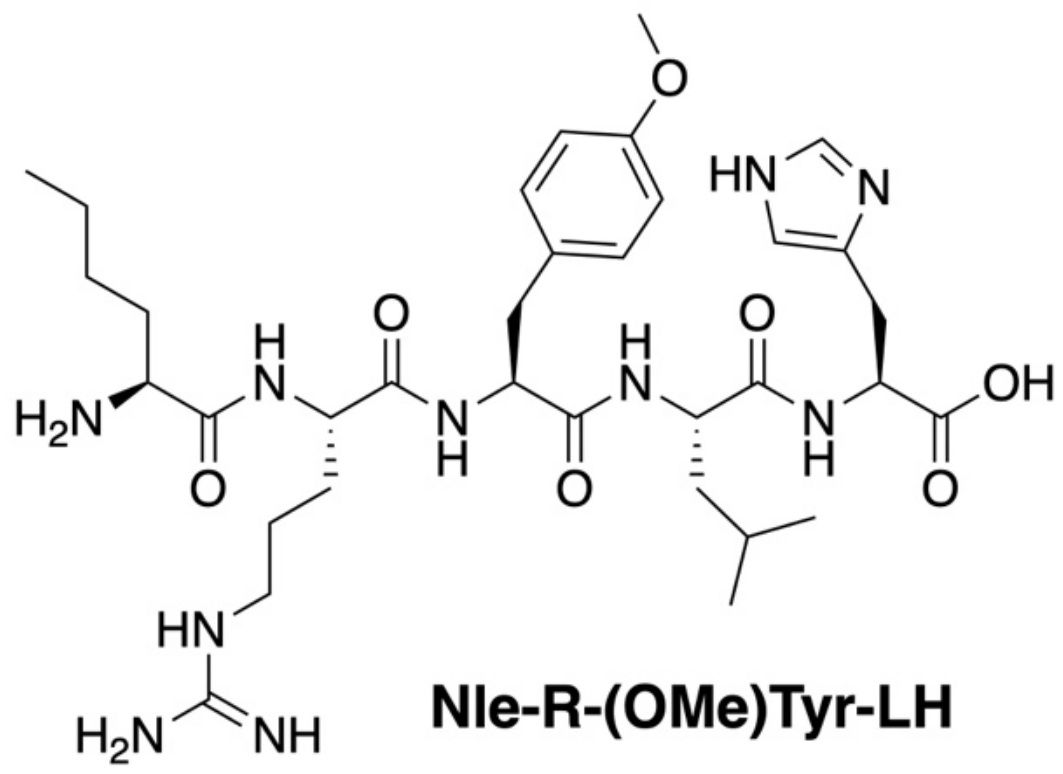

Nle-R-(OMe)Tyr-LH

Chemical Formula: $C_{34}H_{54}N_{10}O_7$
Exact Mass: 714.41769

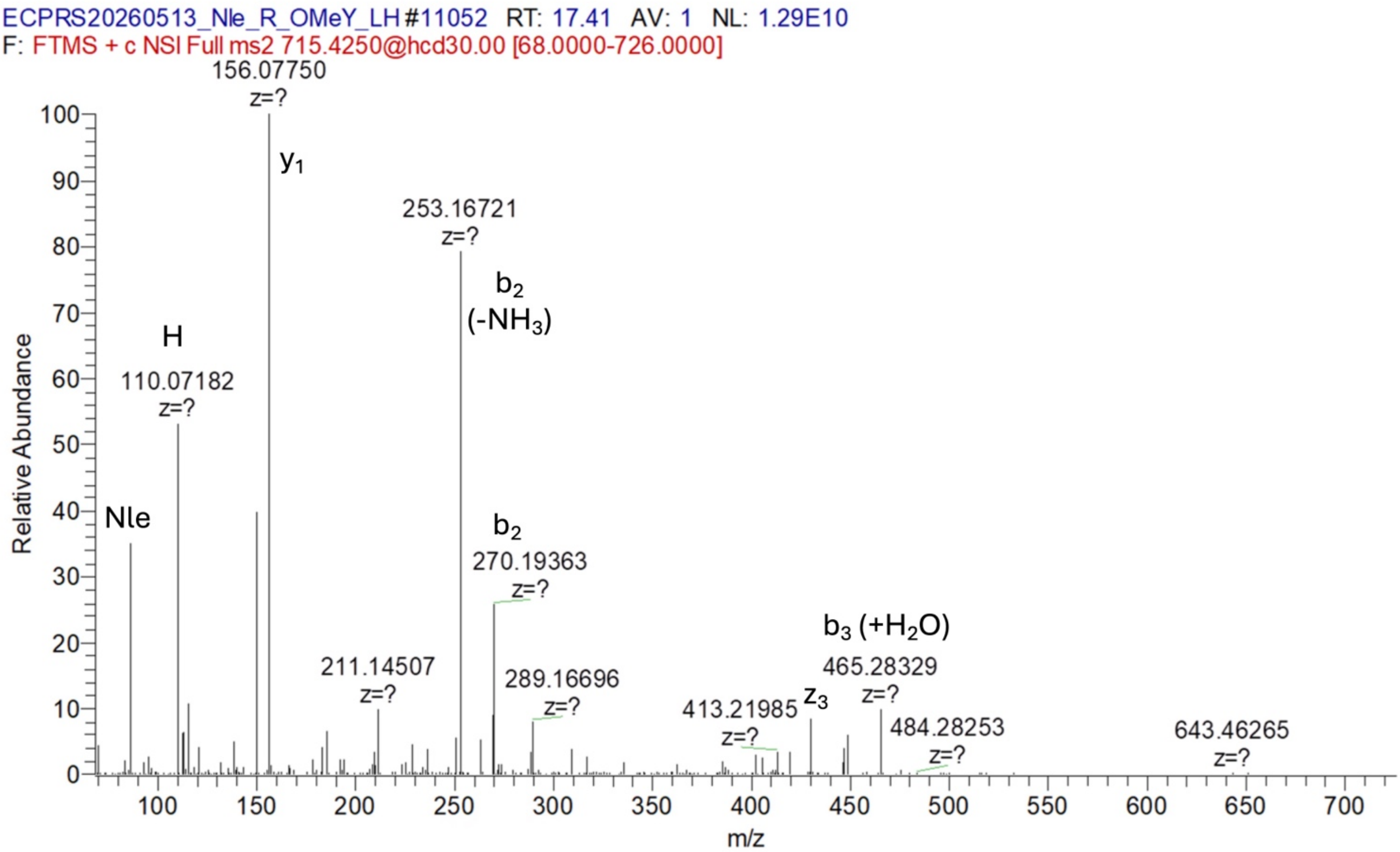

Figure S9. Annotated MS^2 spectrum of Nle-R-**(OMe)Tyr**-LH.

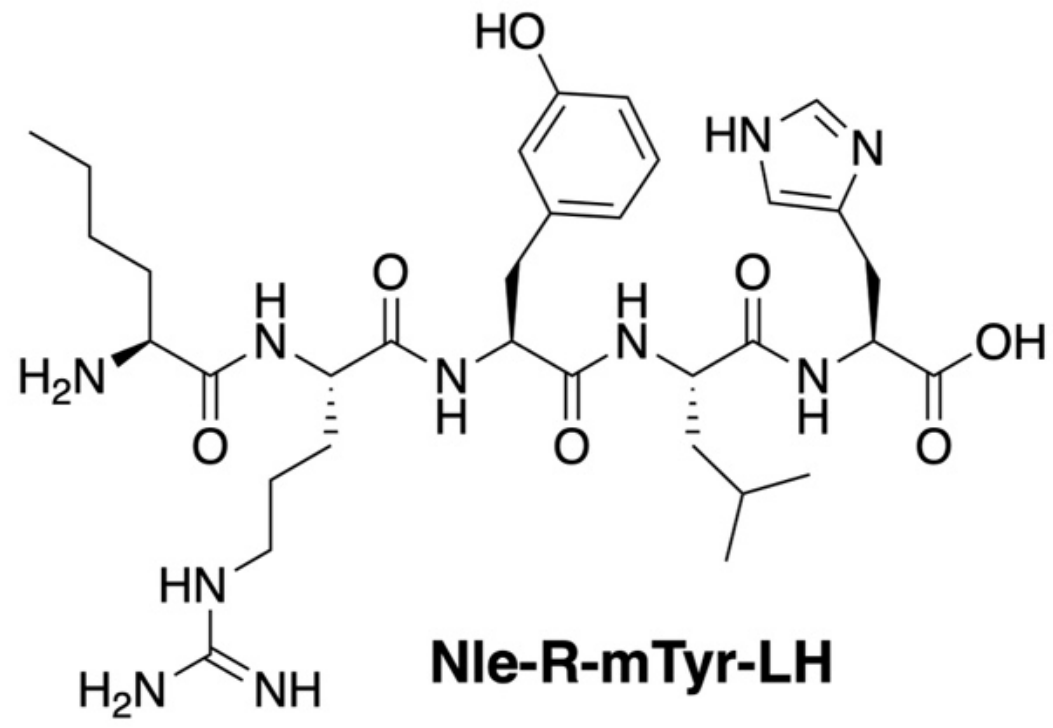

Nle-R-mTyr-LH

Chemical Formula: $C_{33}H_{52}N_{10}O_7$
Exact Mass: 700.40204

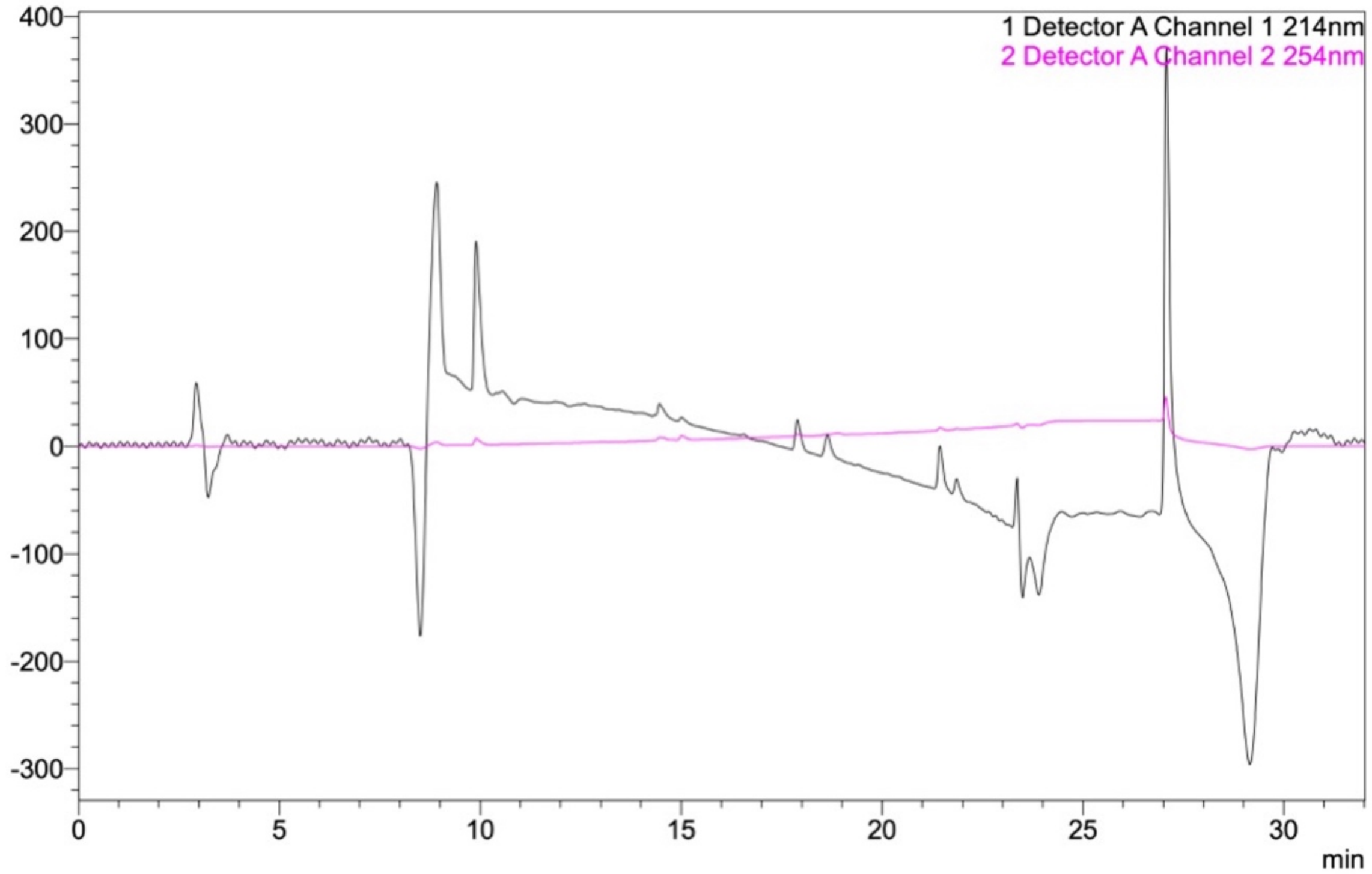

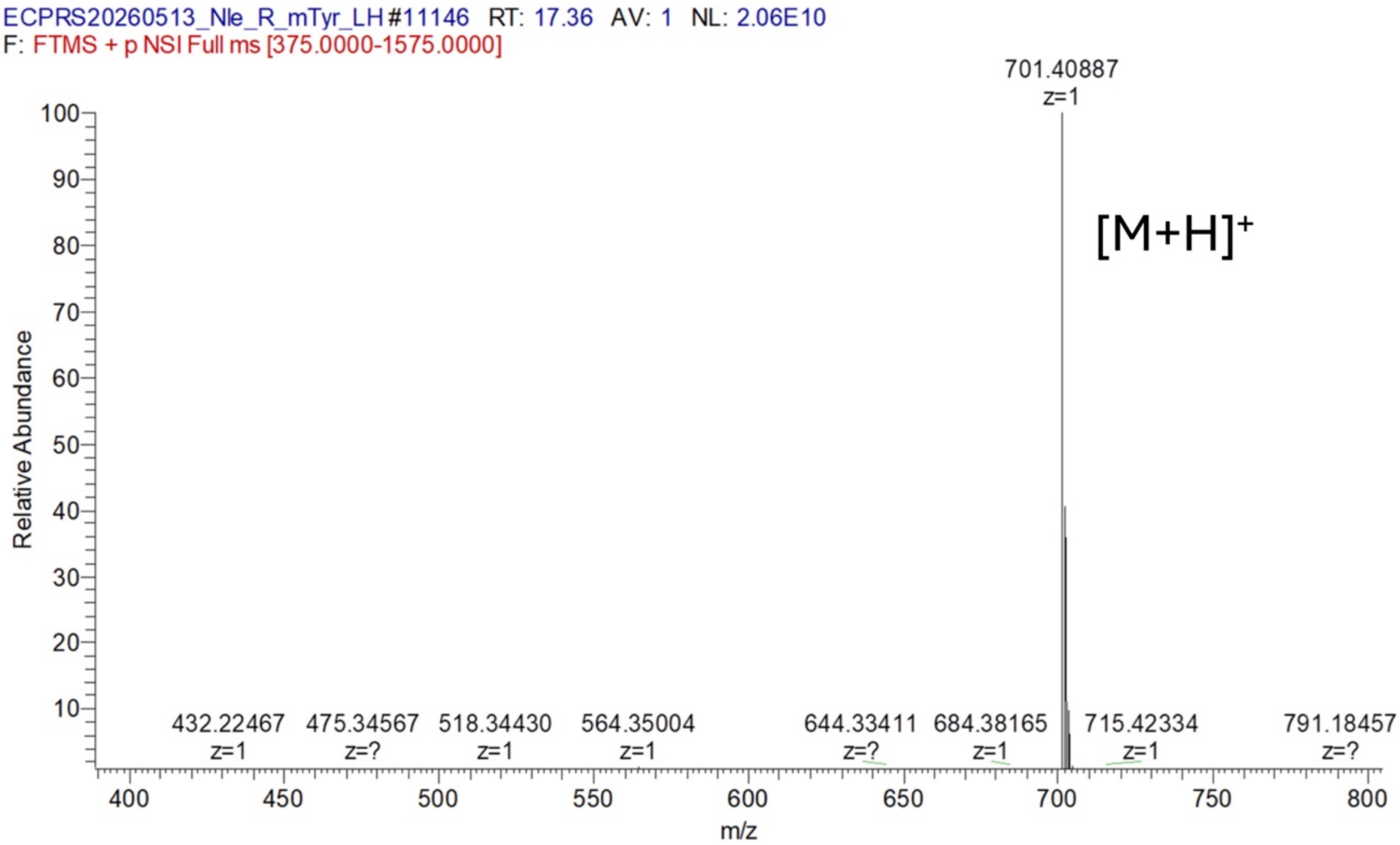

Figure S10. LC-MS characterization of Nle-R-**mTyr**-LH.

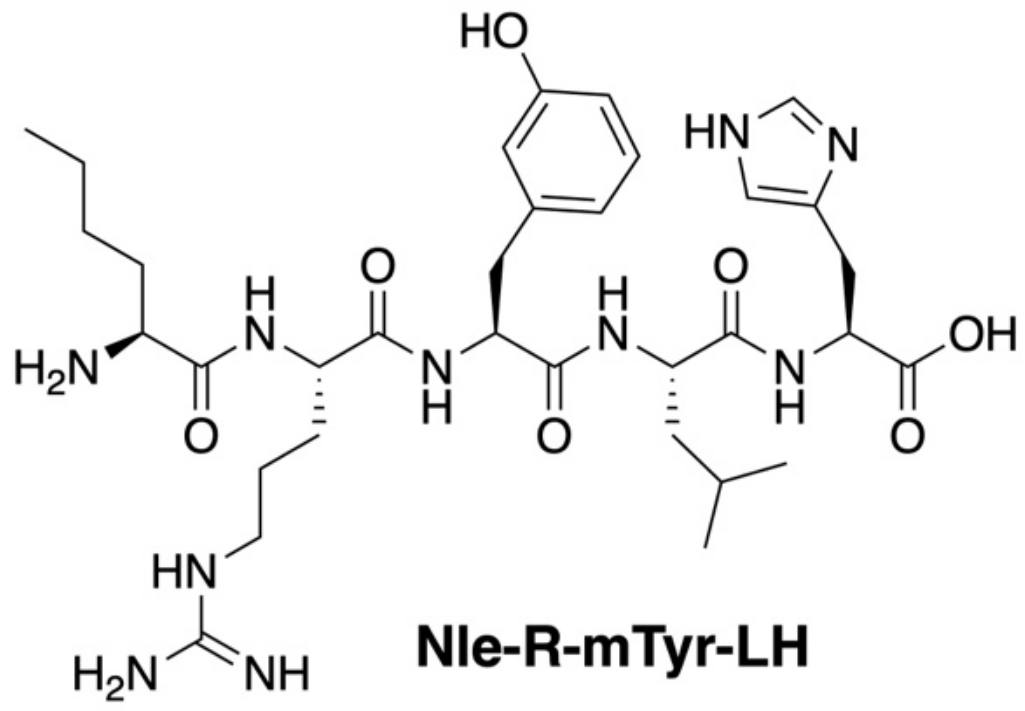

Nle-R-mTyr-LH

Chemical Formula: $C_{33}H_{52}N_{10}O_7$
Exact Mass: 700.40204

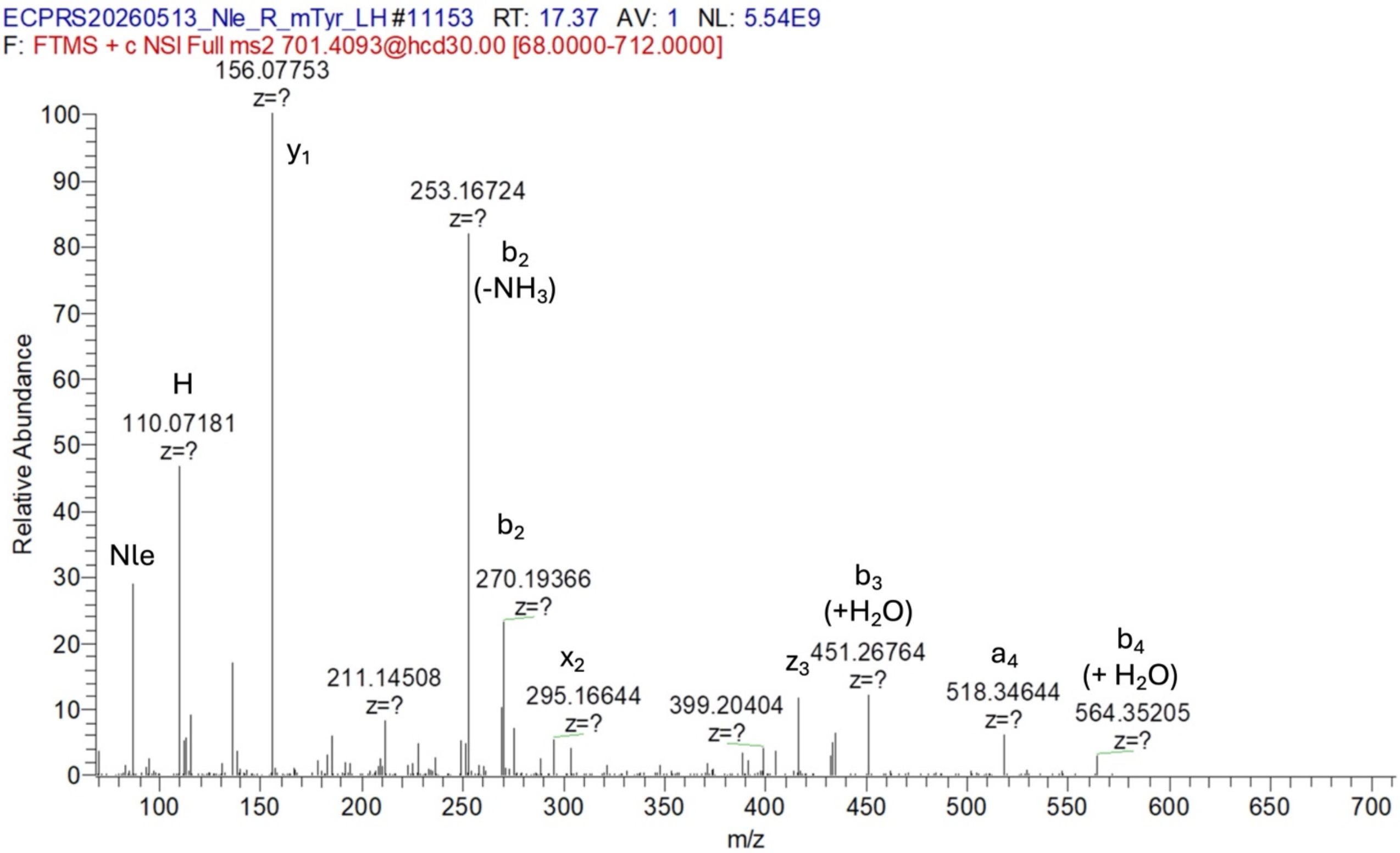

Figure S11. Annotated MS^2 spectrum of Nle-R-**mTyr**-LH.

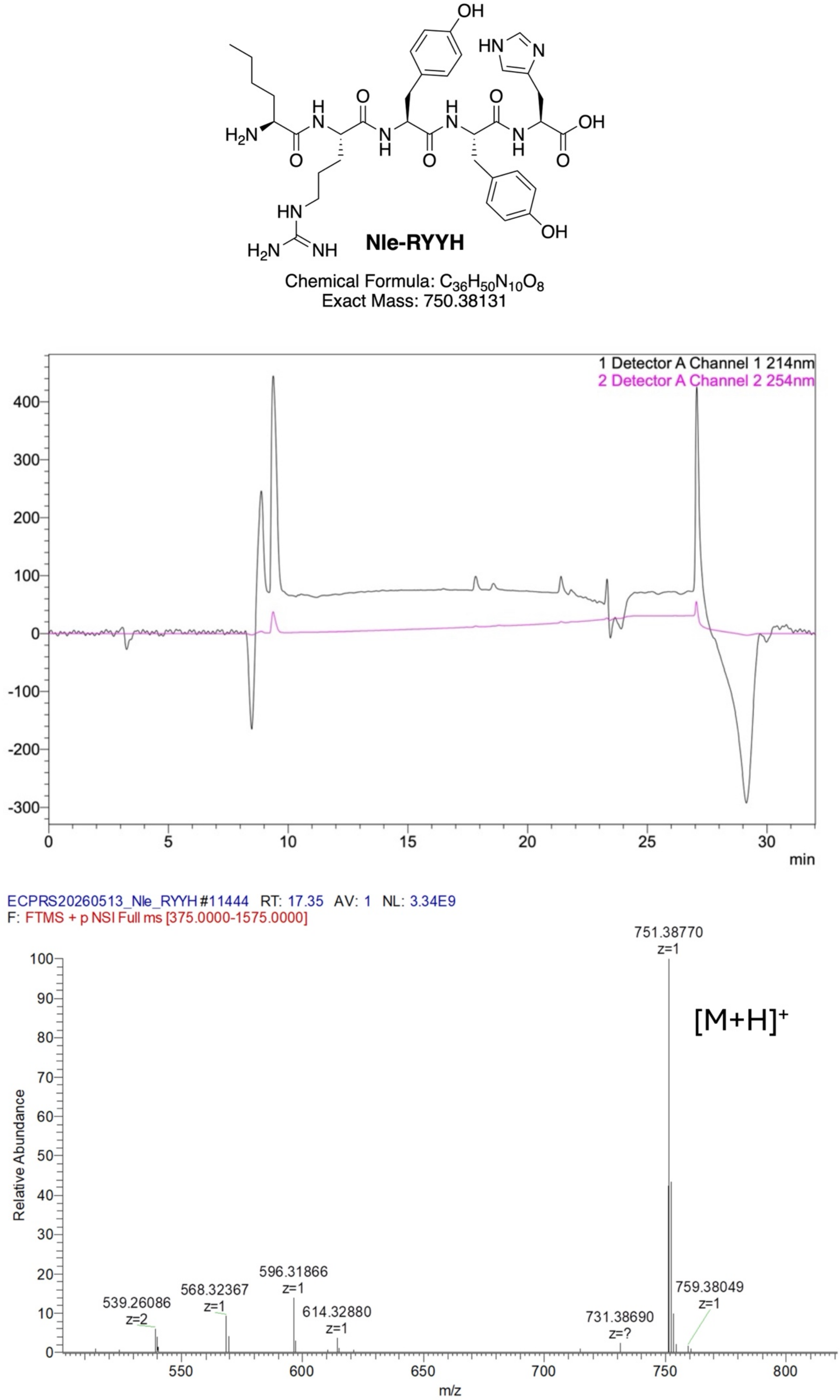

Figure S12. LC-MS characterization of Nle-RY**Y**H.

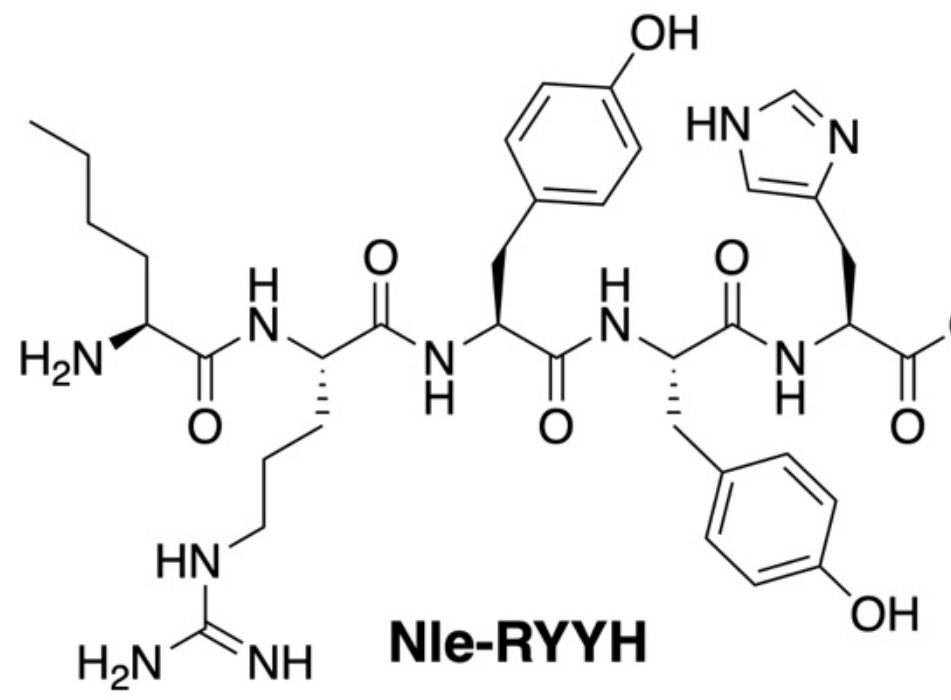

Nle-RYYH

Chemical Formula: $C_{36}H_{50}N_{10}O_8$
Exact Mass: 750.38131

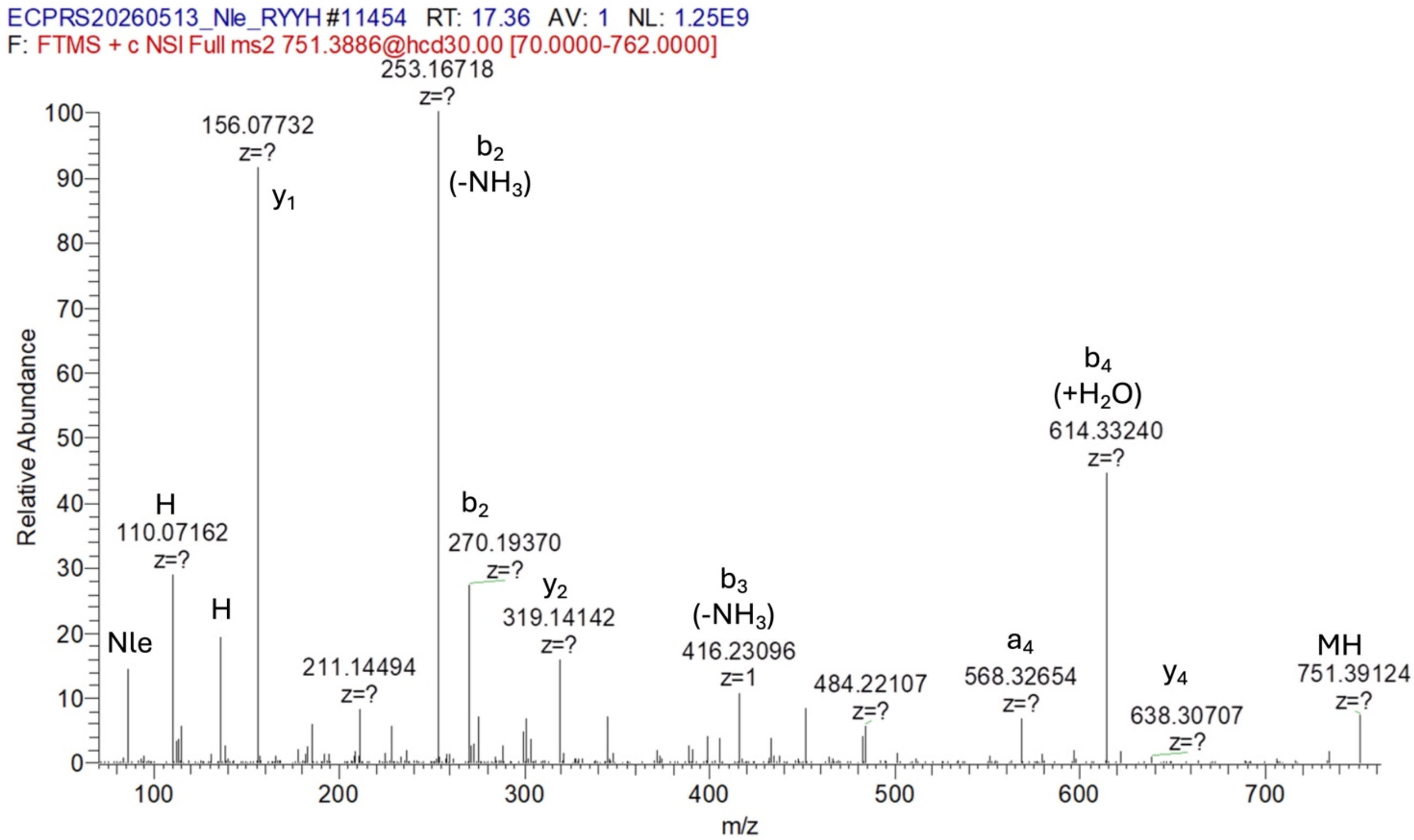

Figure S13. Annotated MS[2] spectrum of Nle-RY**Y**H.

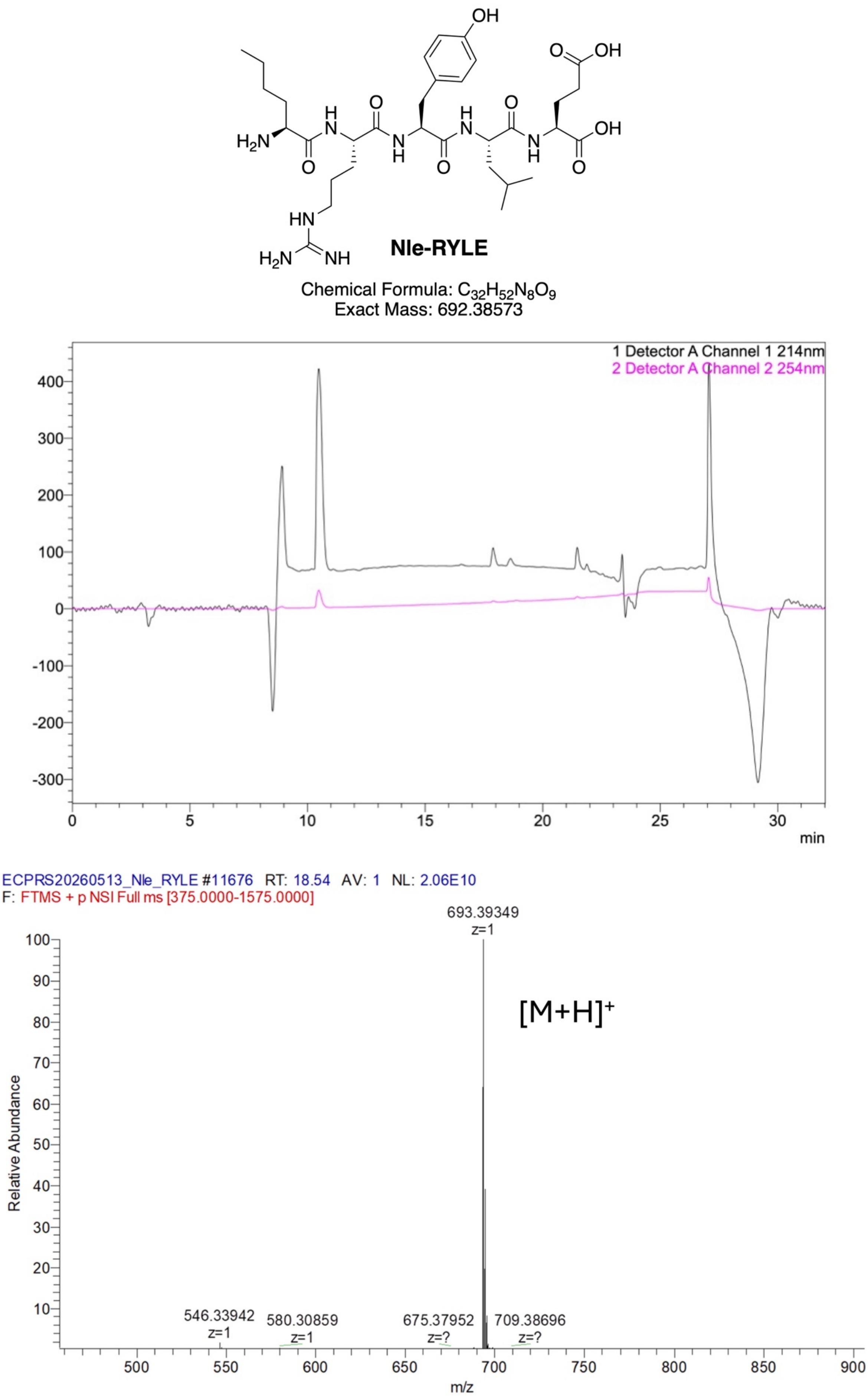

Figure S14. LC-MS characterization of Nle-RYLE.

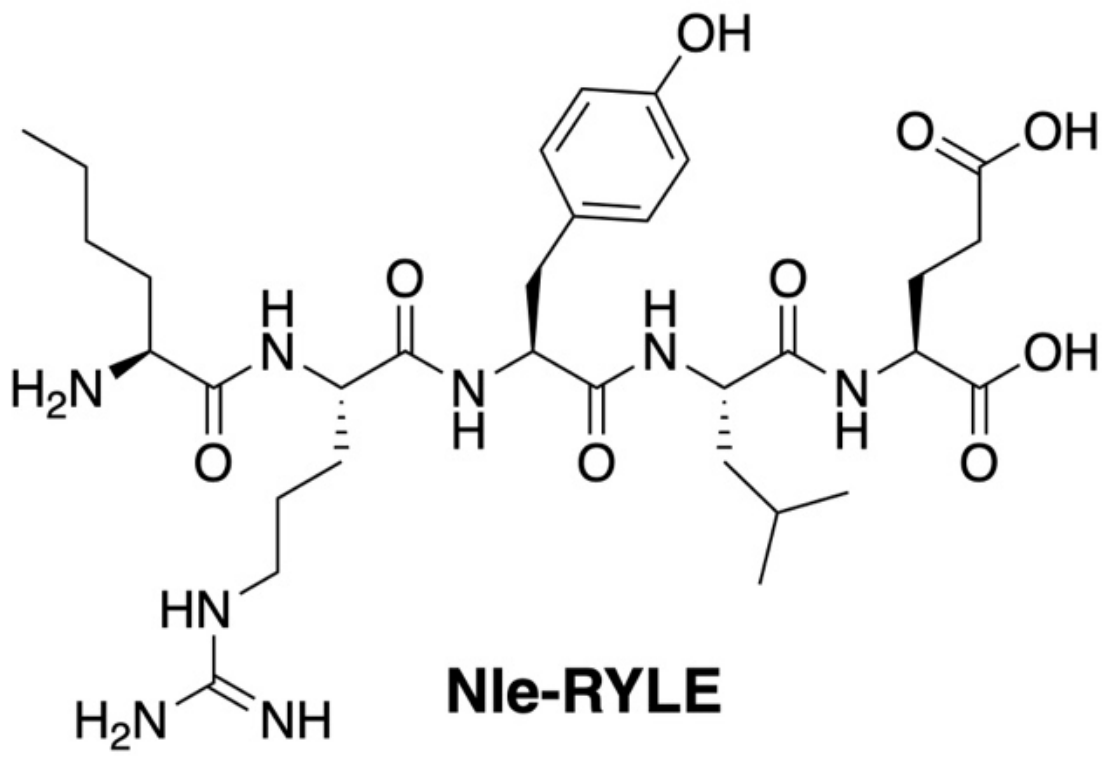

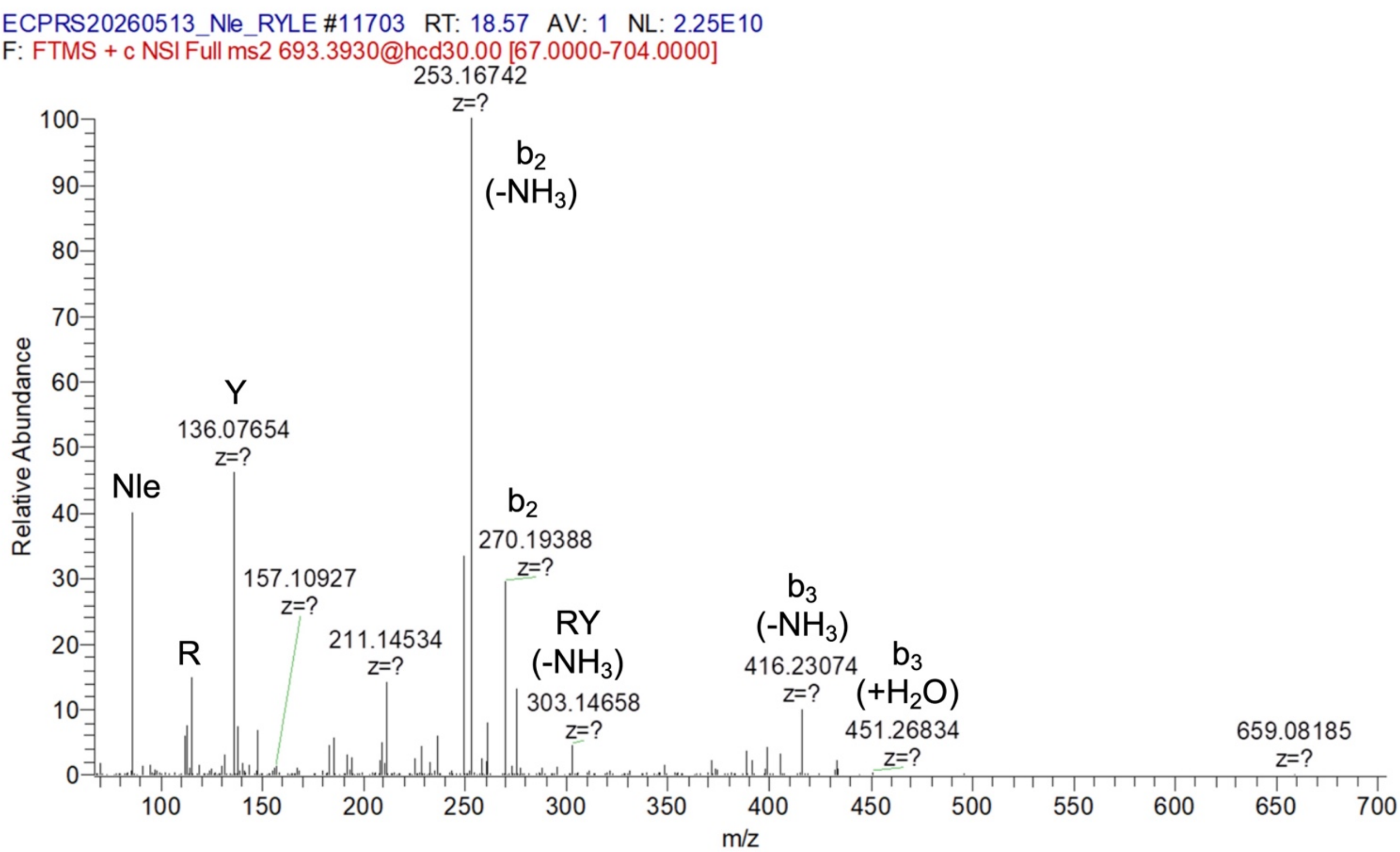

Figure S15. Annotated MS^2 spectrum of Nle-RYL**E**.

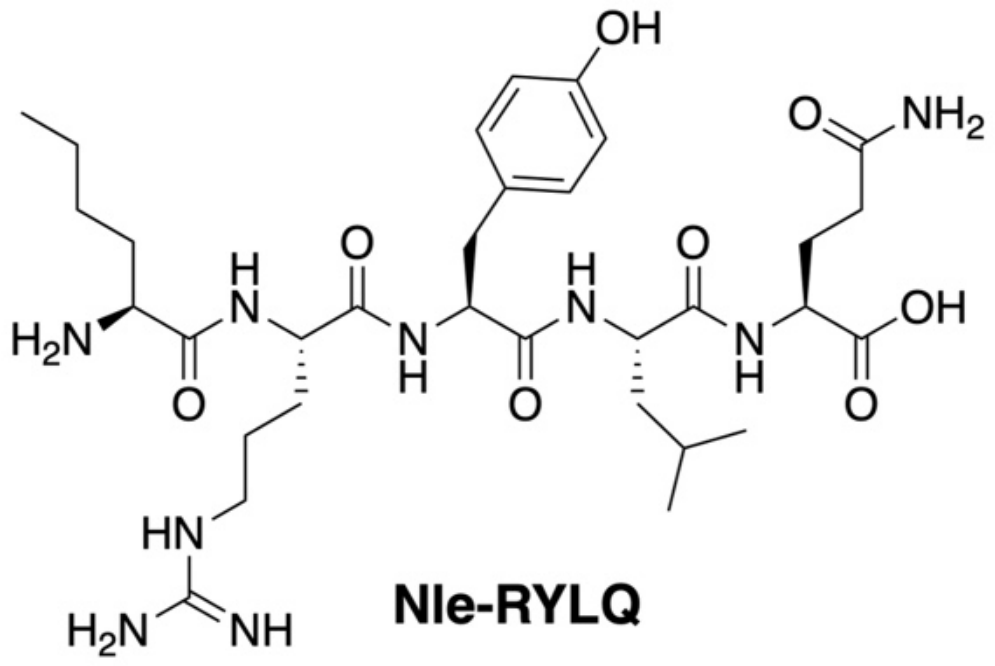

Nle-RYLQ

Chemical Formula: $C_{32}H_{53}N_9O_8$
Exact Mass: 691.40171

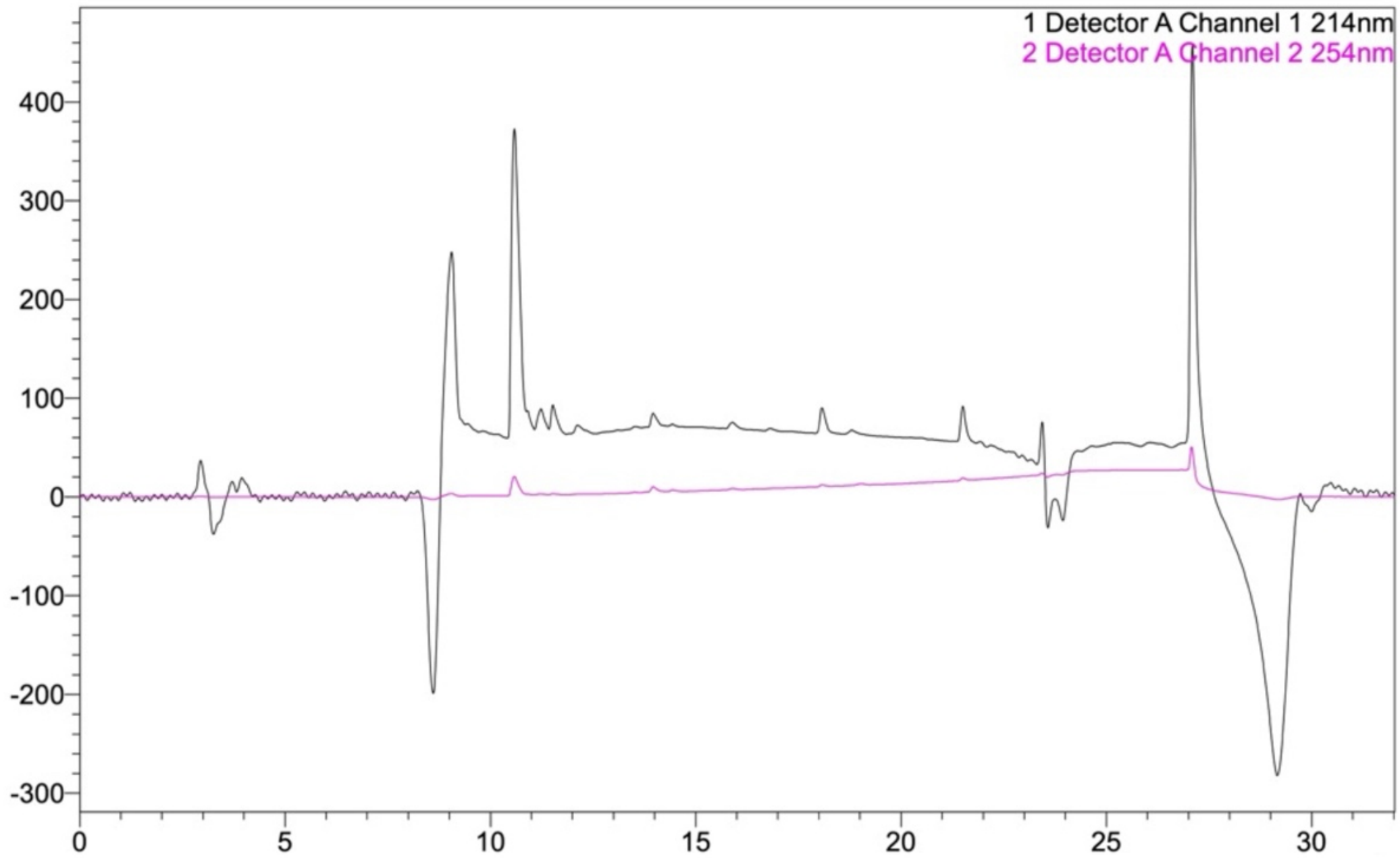

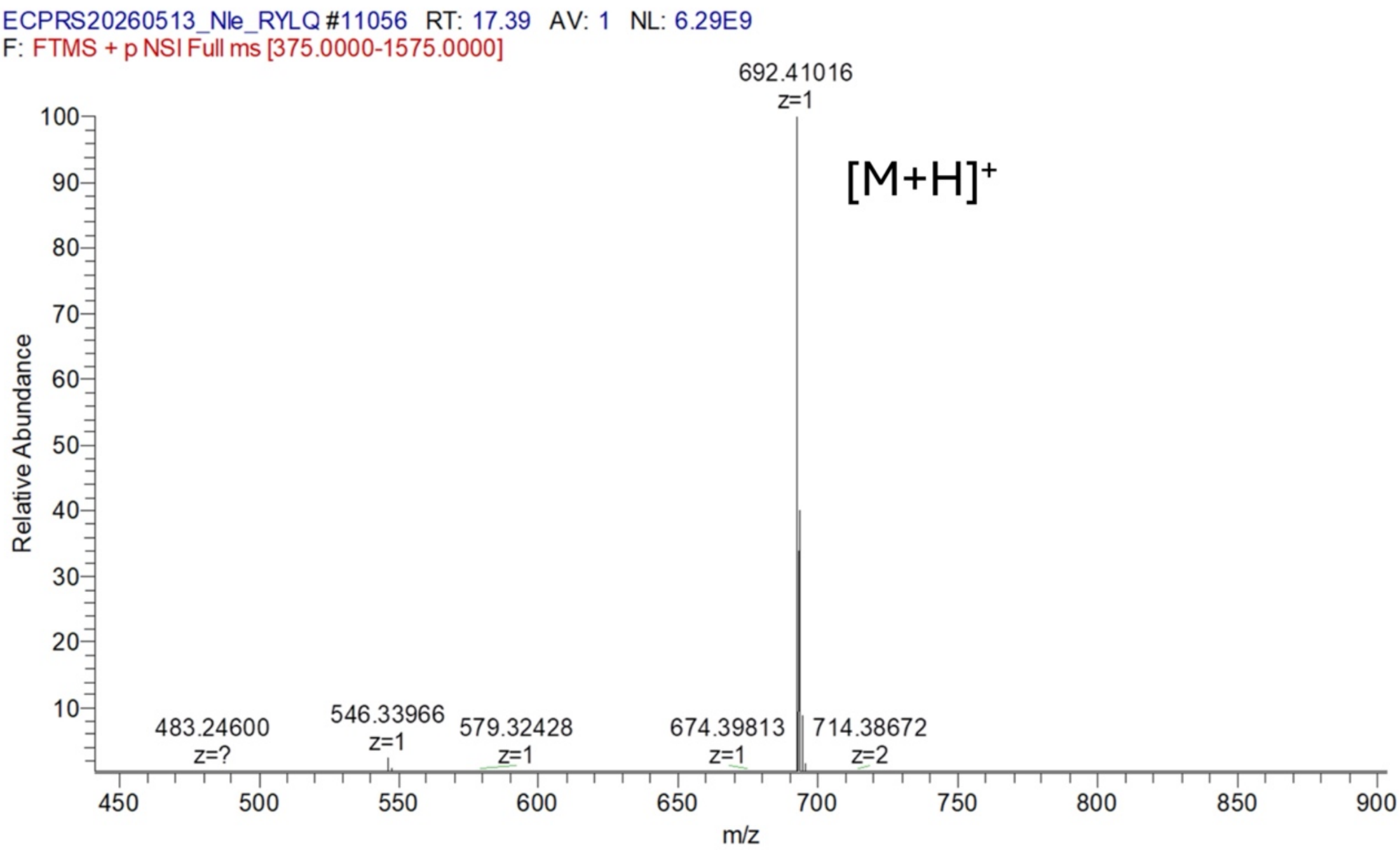

Figure S16. LC-MS characterization of Nle-RYL**Q**.

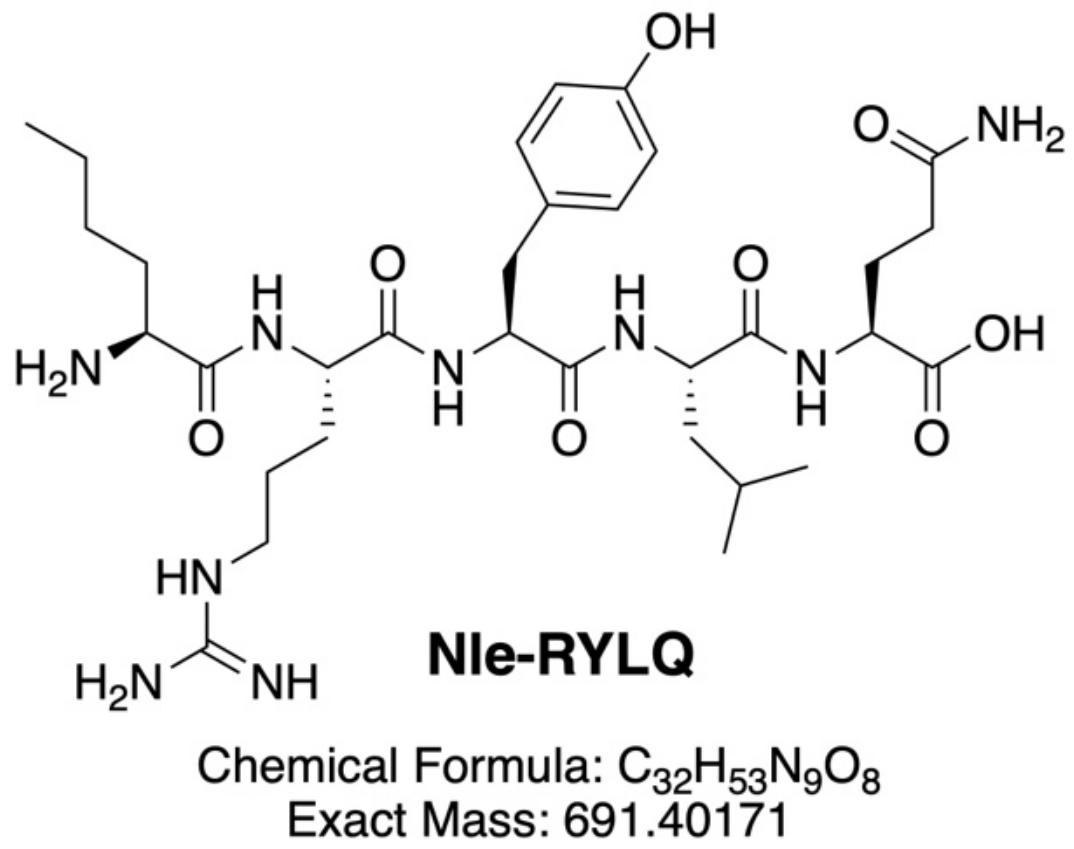

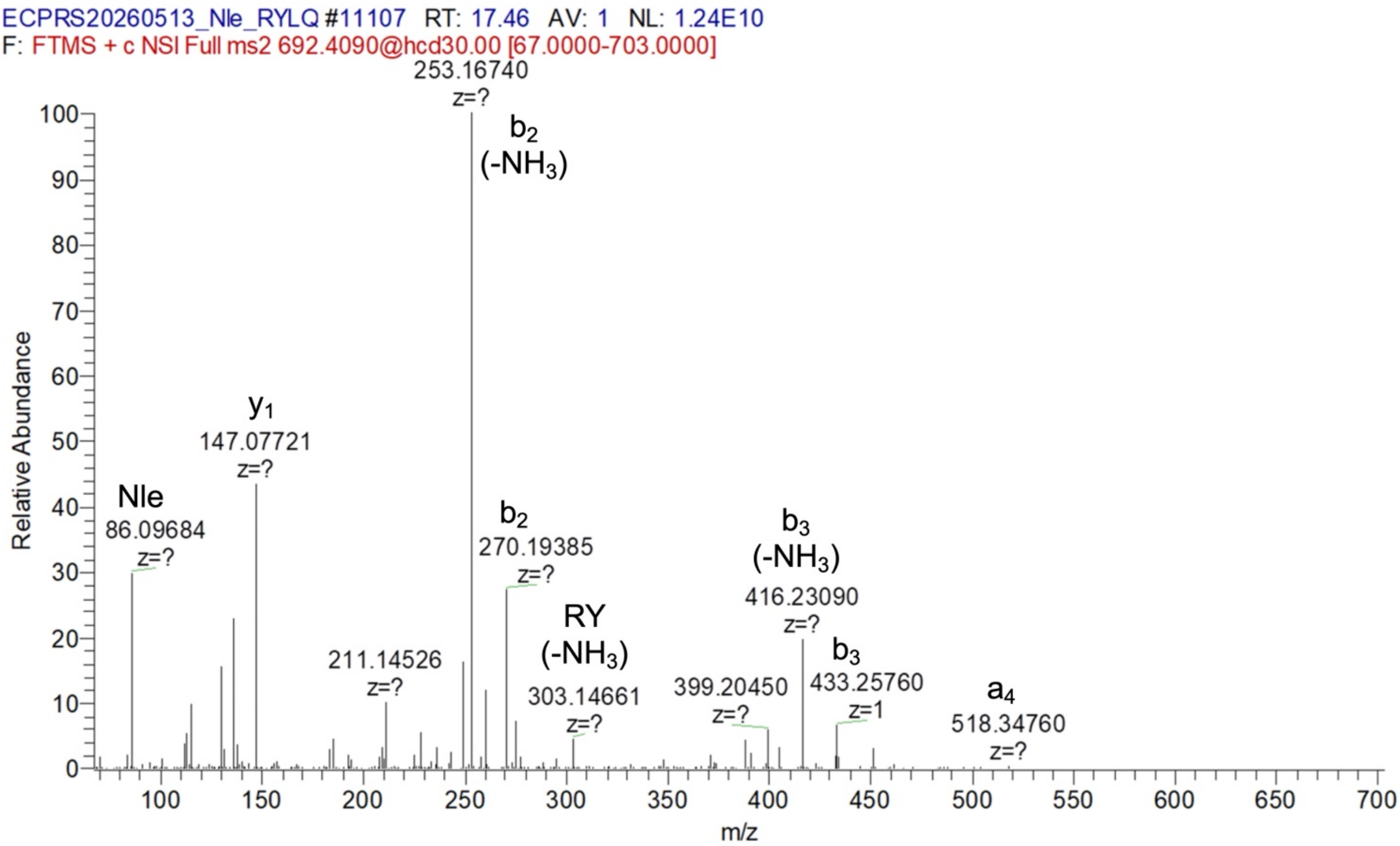

Figure S17. Annotated MS^2 spectrum of Nle-RYL**Q**.

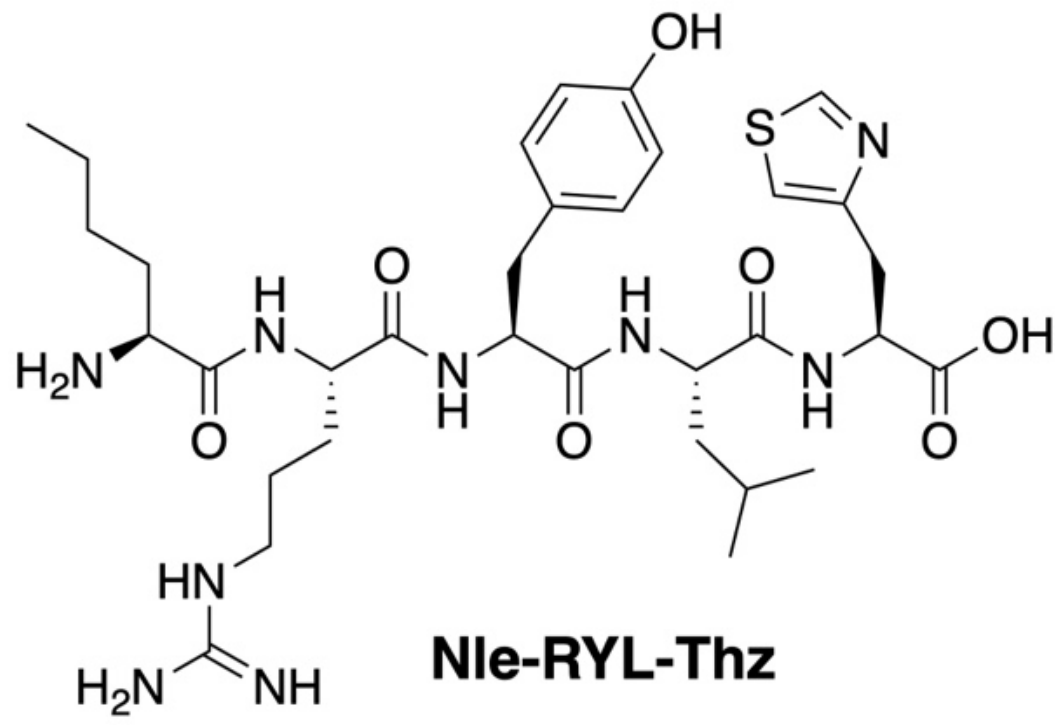

Nle-RYL-Thz

Chemical Formula: $C_{33}H_{51}N_{9}O_{7}S$
Exact Mass: 717.36322

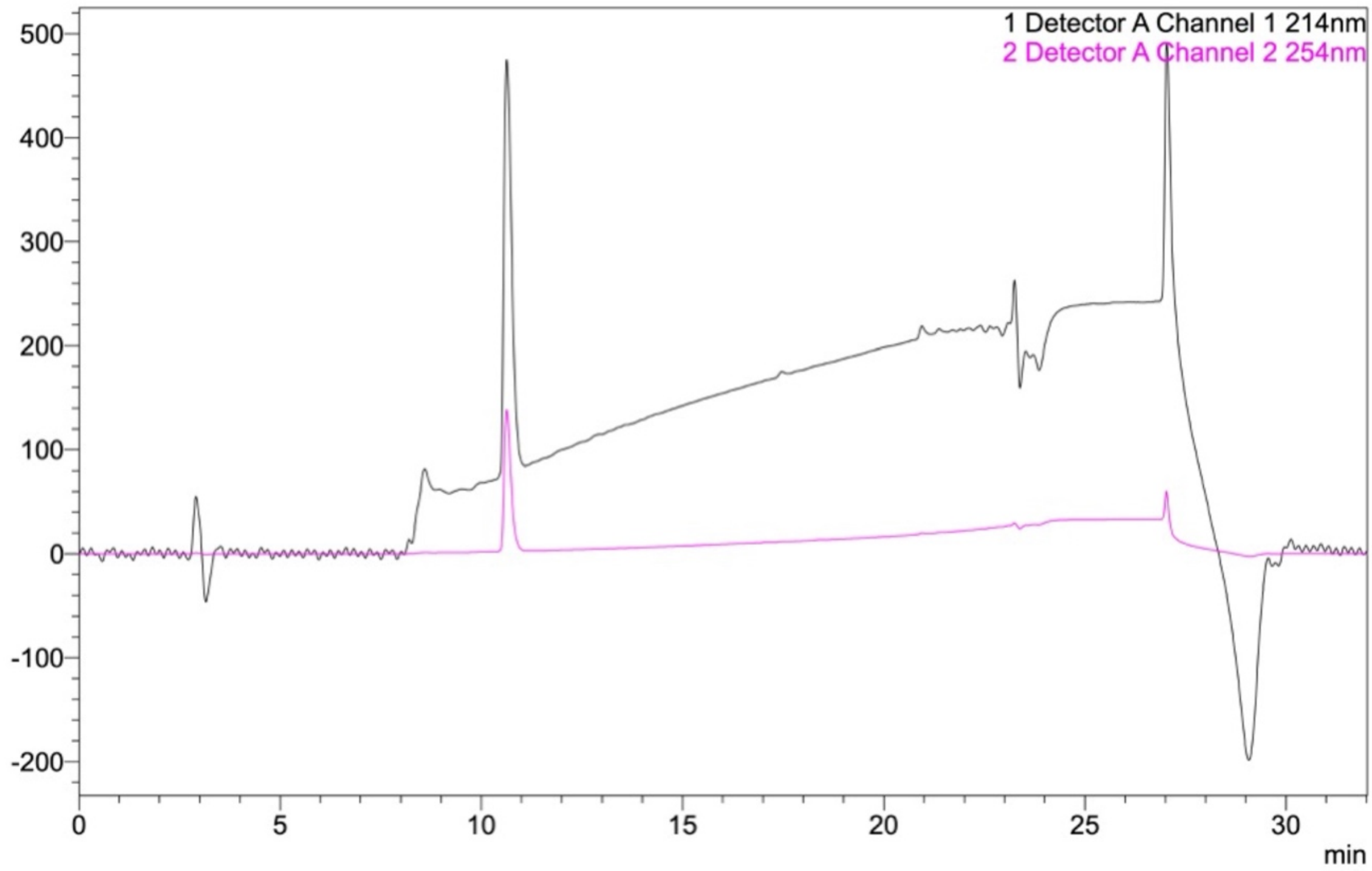

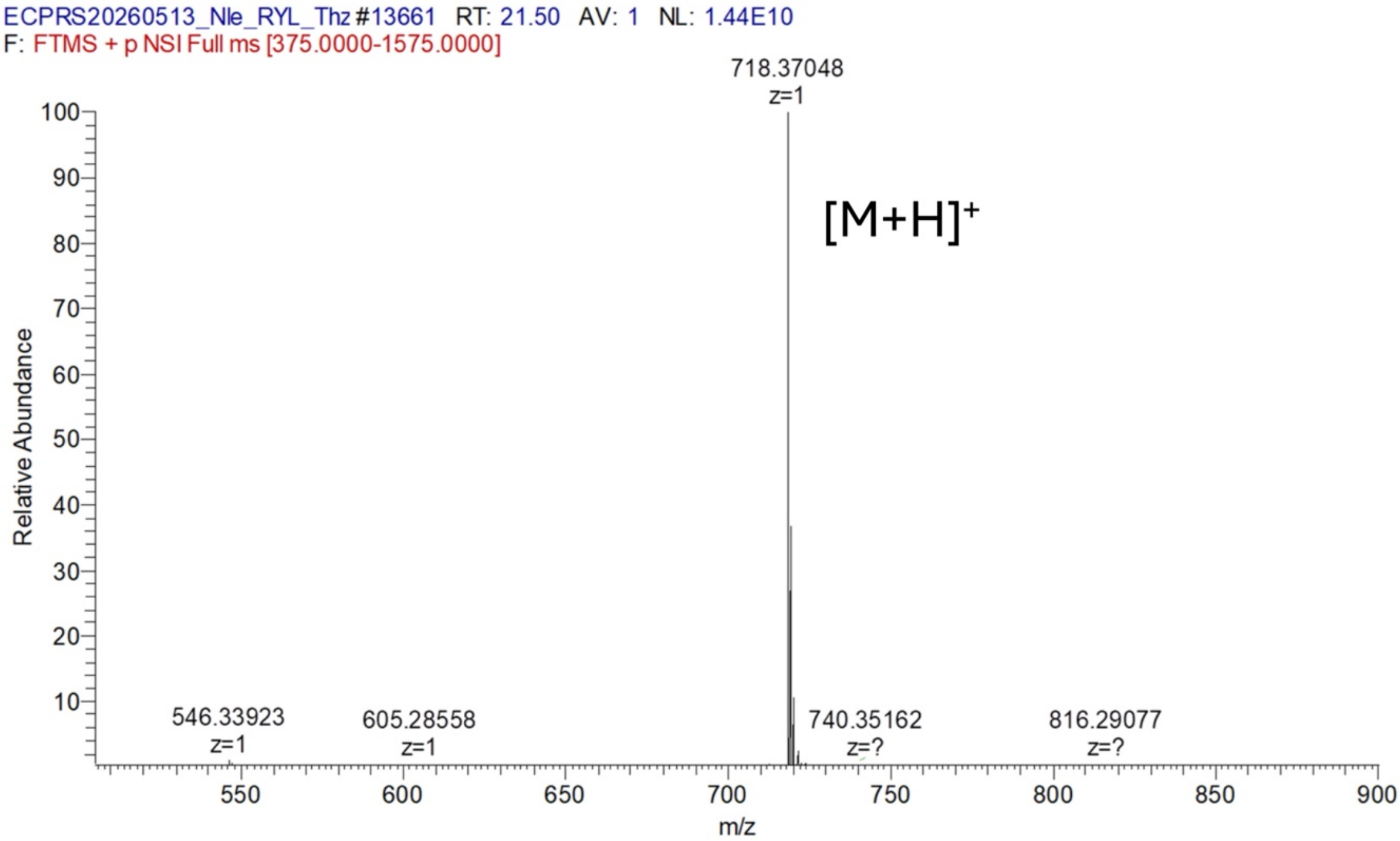

Figure S18. LC-MS characterization of Nle-RYL-**Thz**.

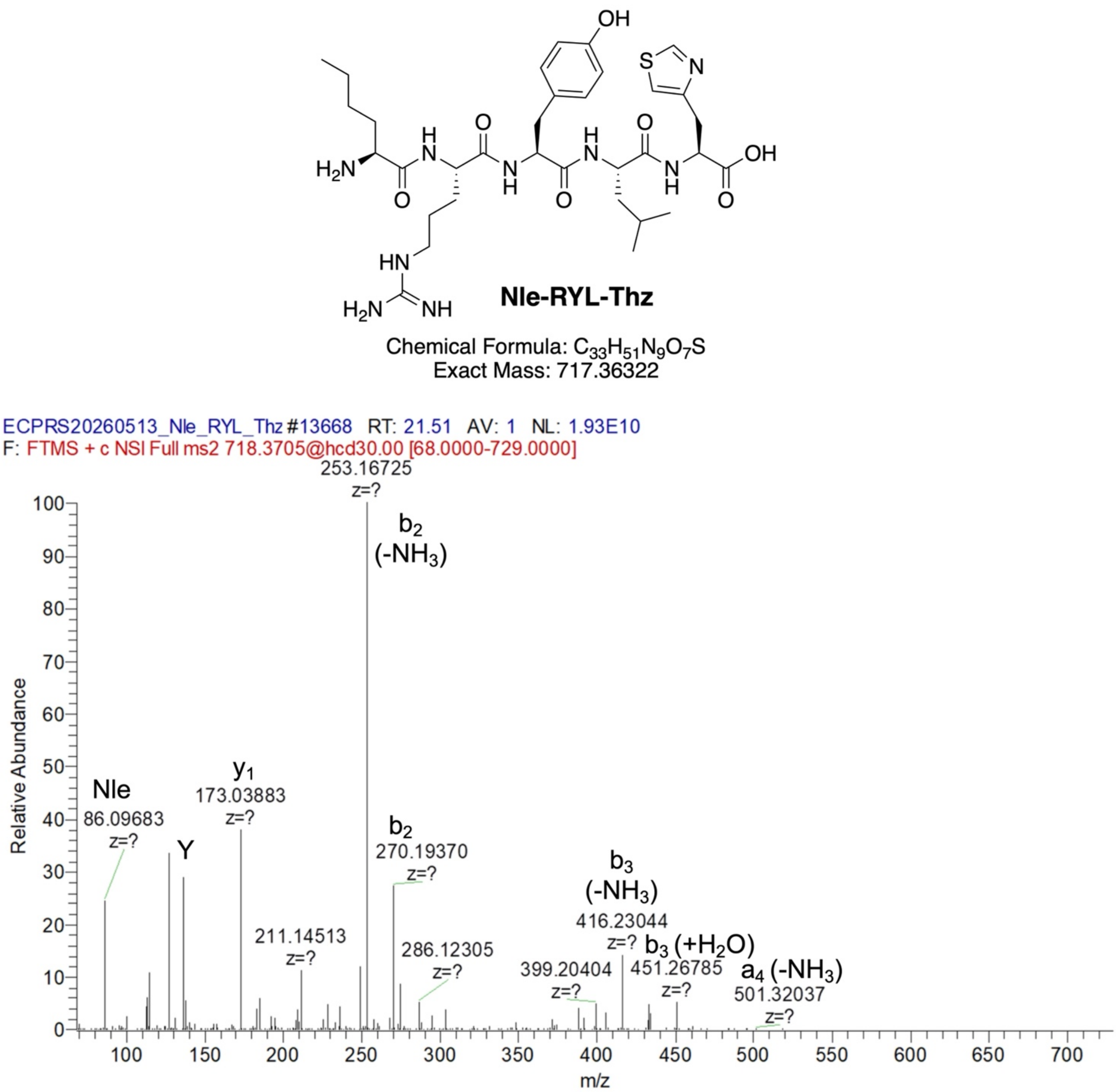

Figure S19. Annotated MS[2] spectrum of Nle-RYL-**Thz**.

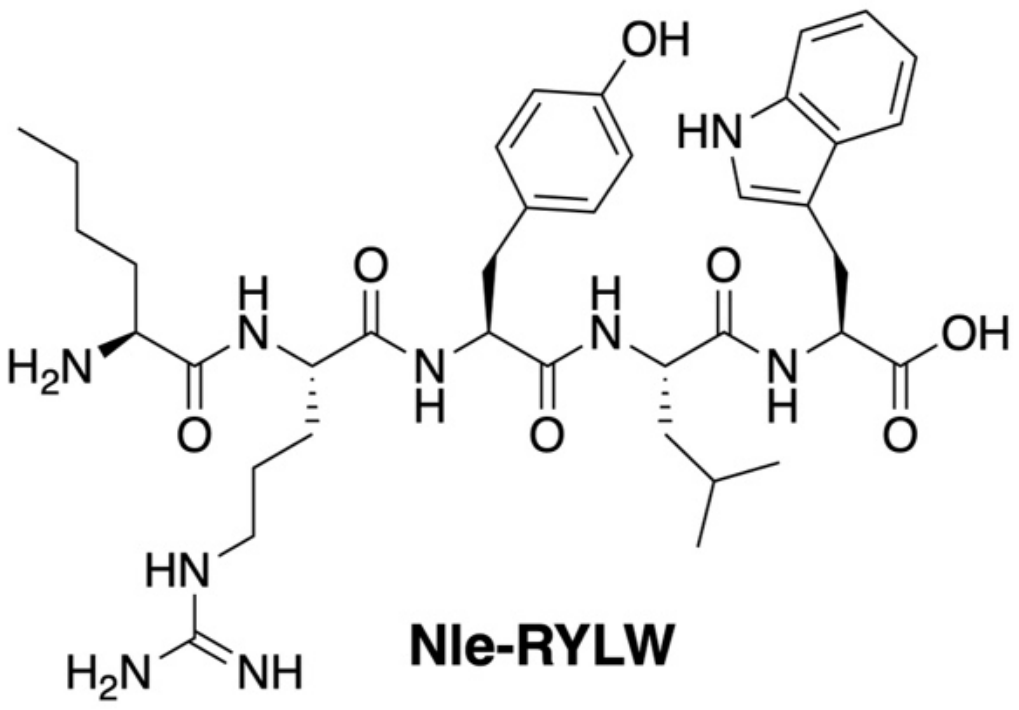

Nle-RYLW

Chemical Formula: $C_{38}H_{55}N_9O_7$
Exact Mass: 749.42245

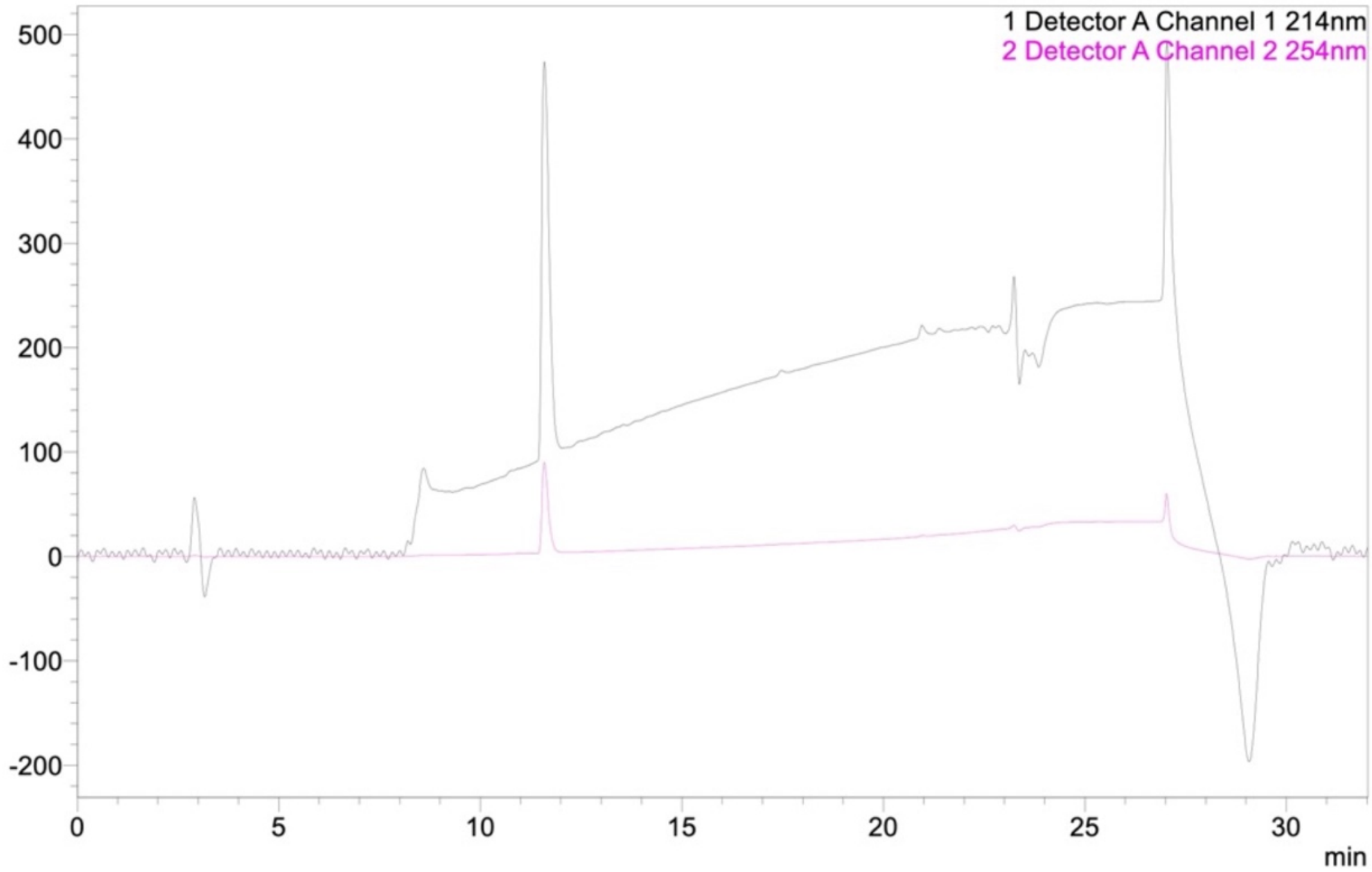

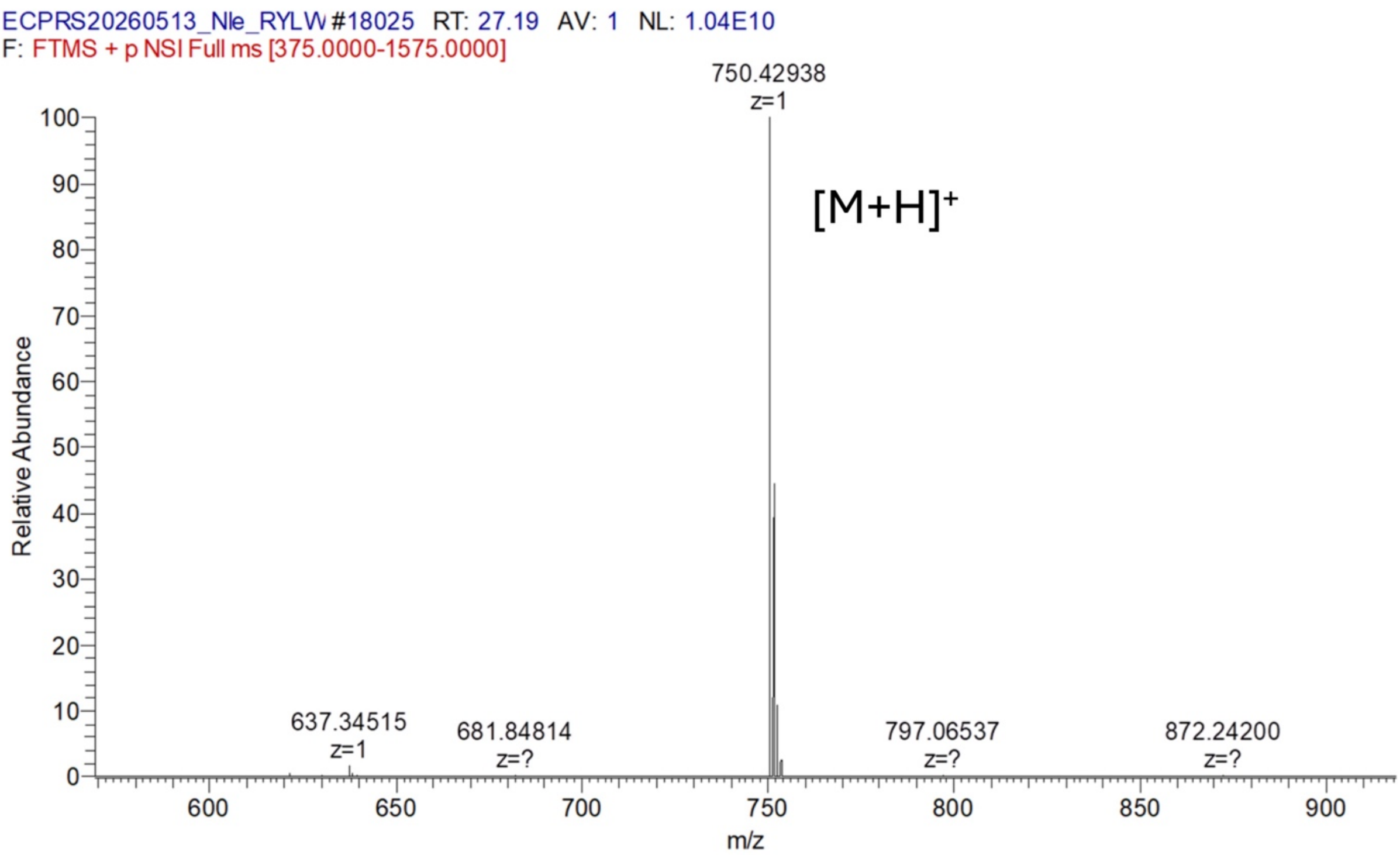

Figure S20. LC-MS characterization of Nle-RYL**W**.

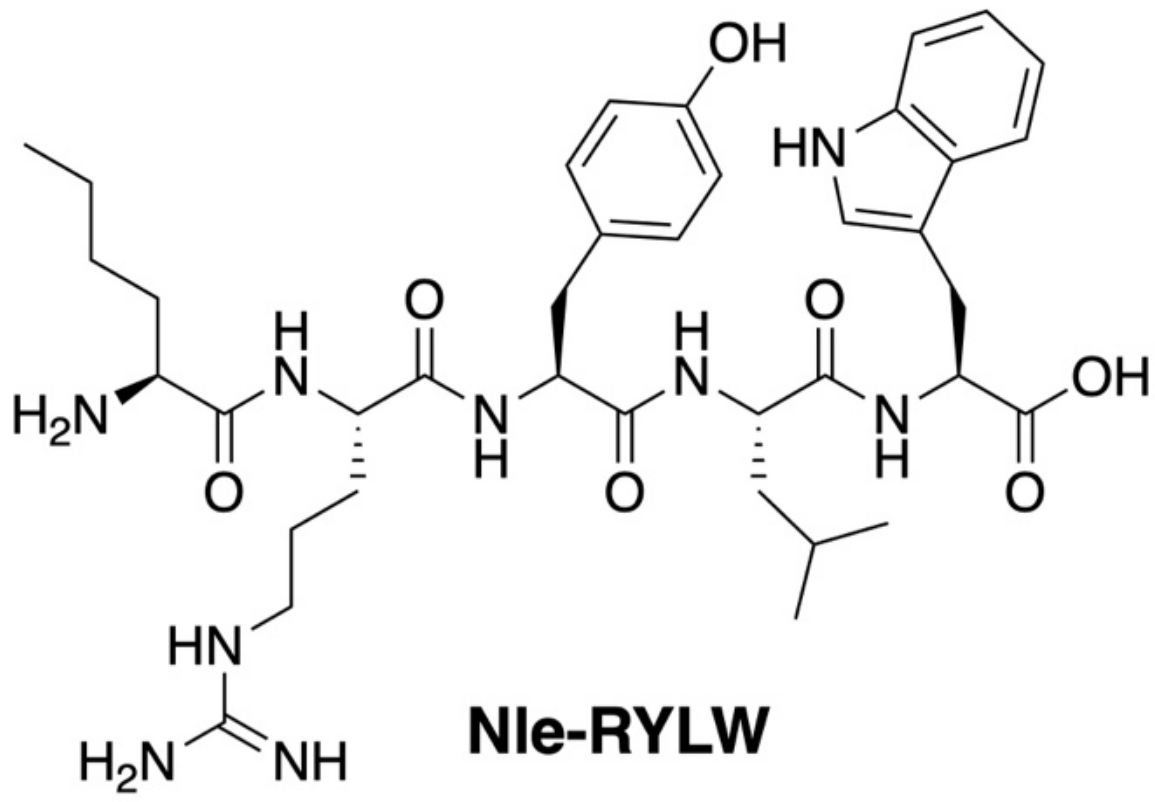

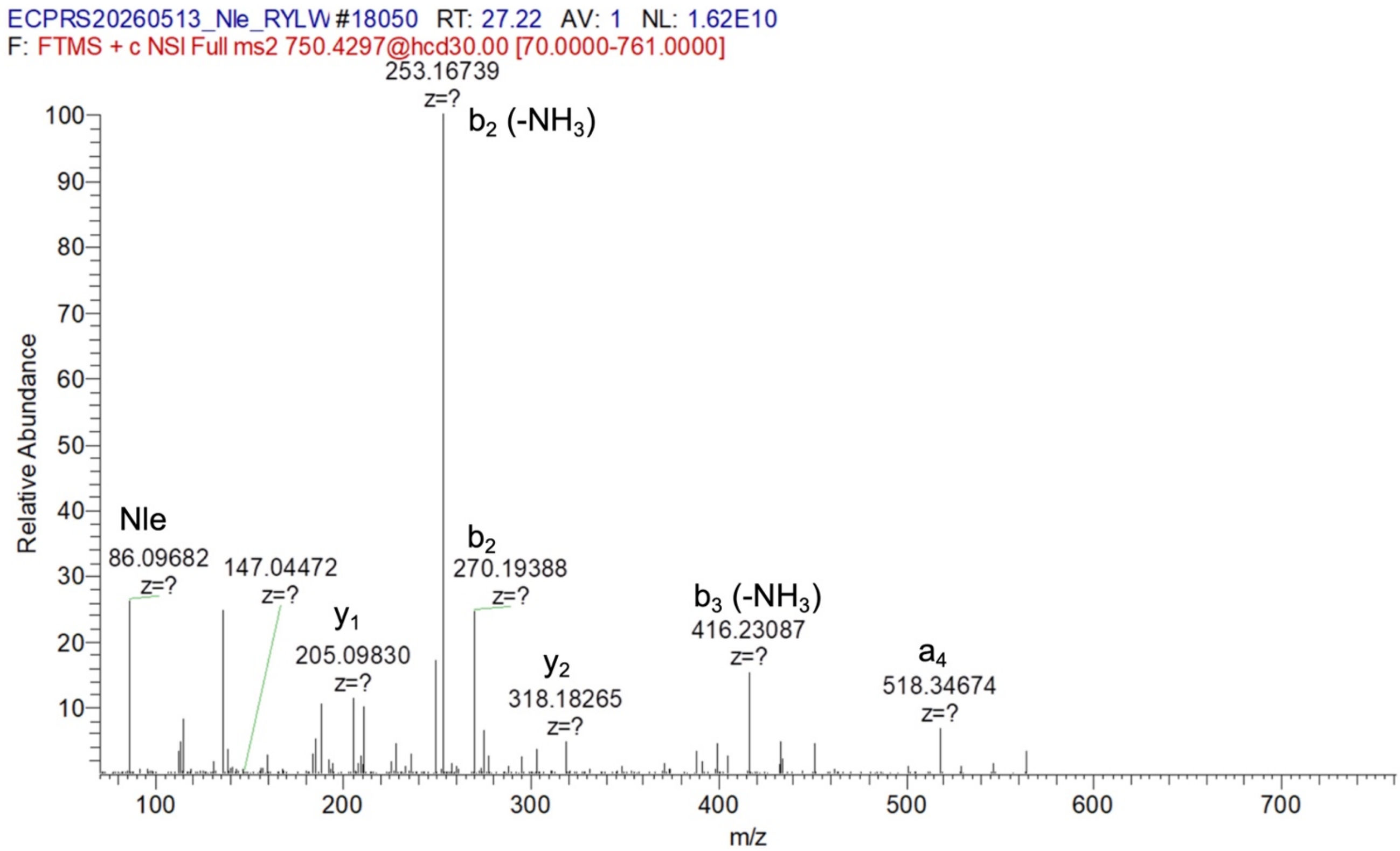

Figure S21. Annotated MS^2 spectrum of Nle-RYL**W**.

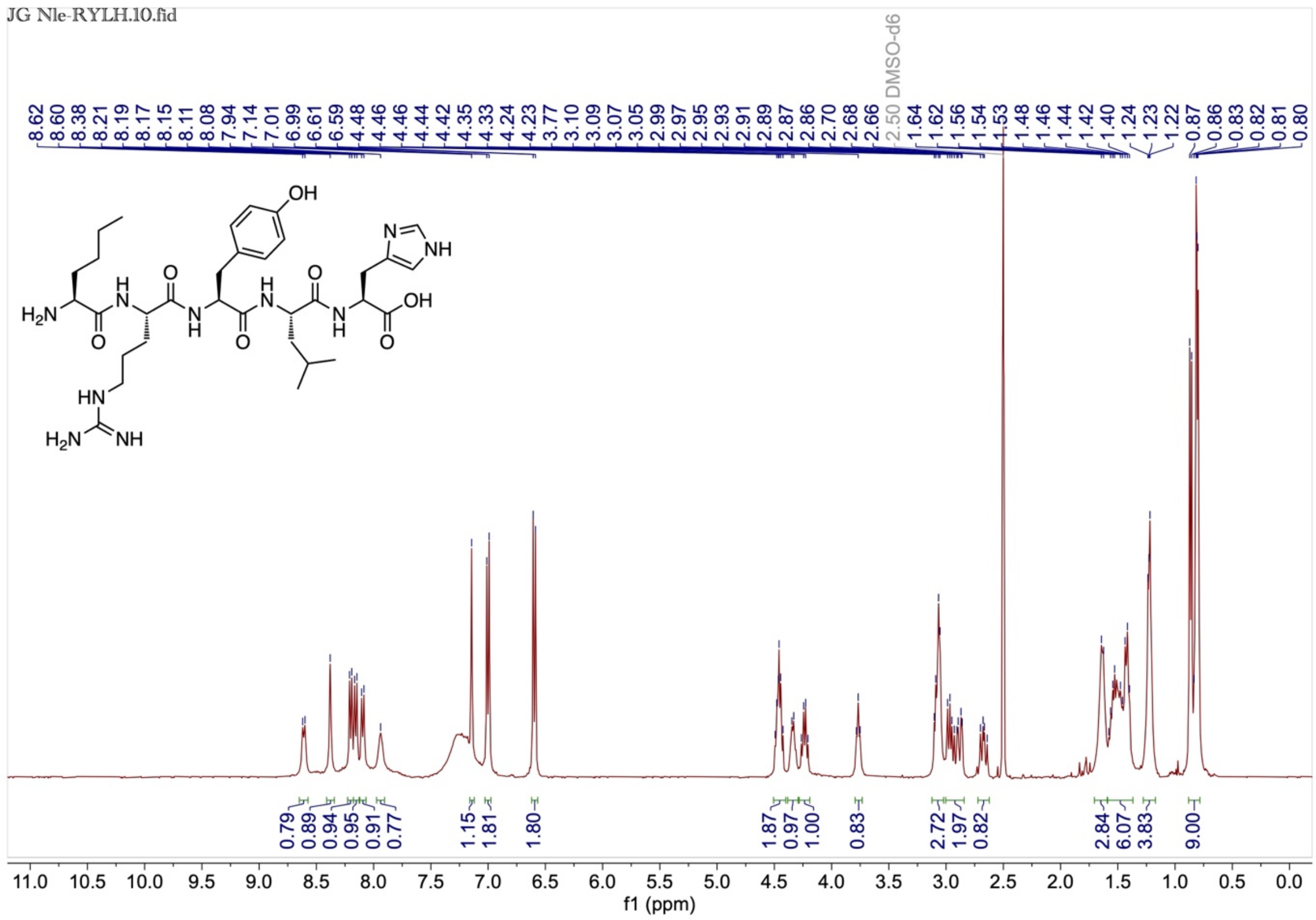

Figure S22. ^{1}H NMR of Nle-RYLH in DMSO-d_6.

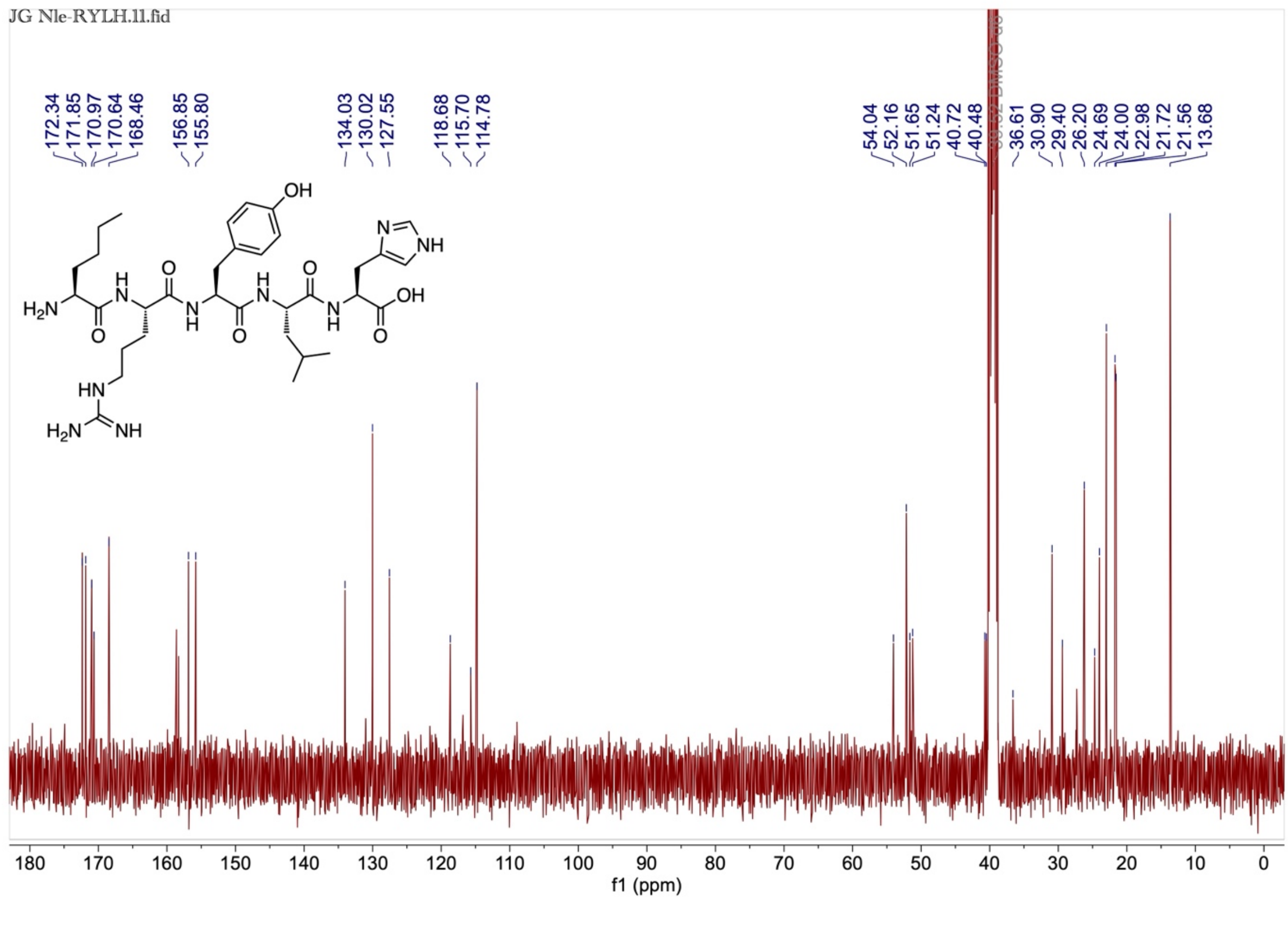

Figure S23. ^{13}C NMR of Nle-RYLH in DMSO-d_6.

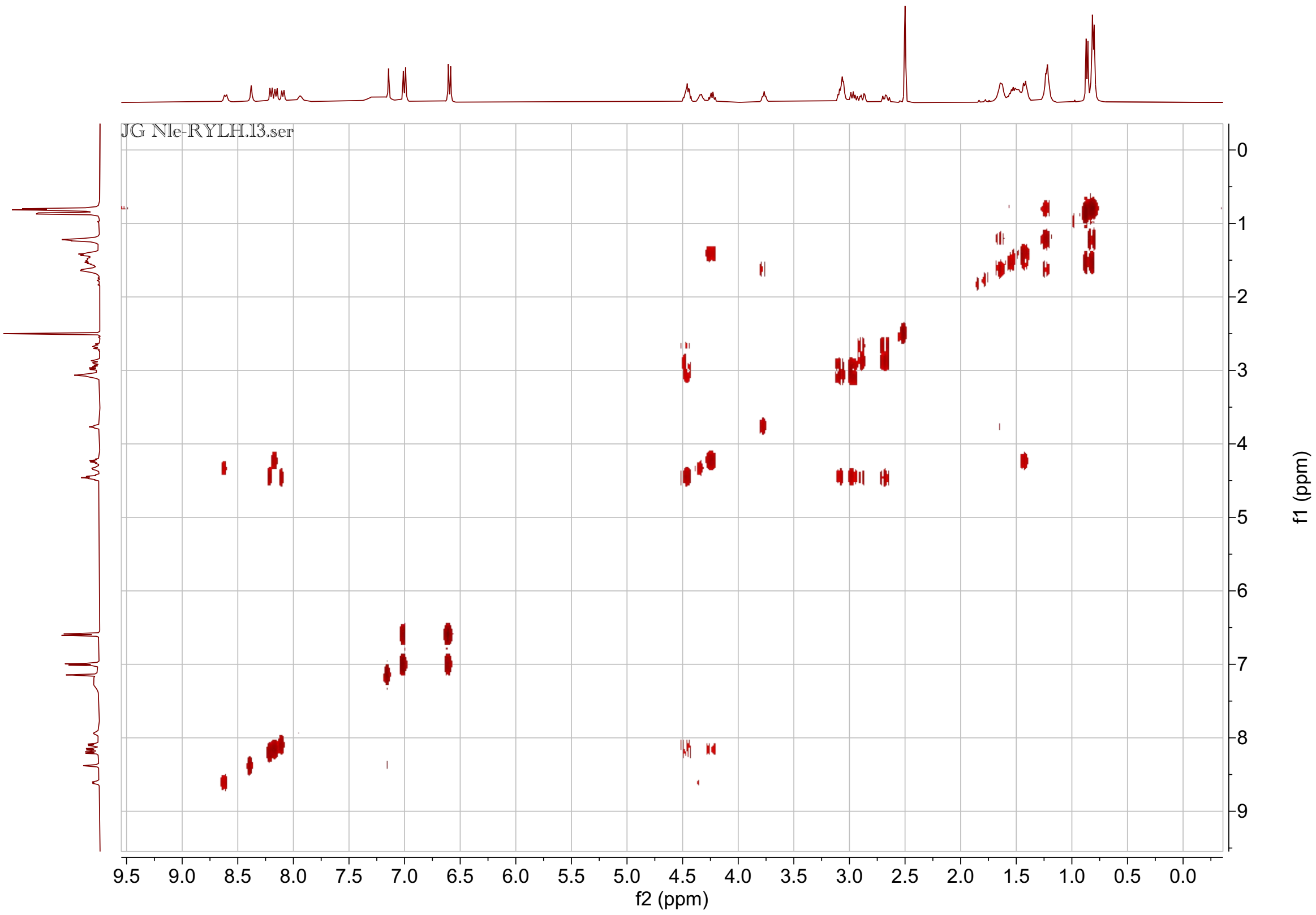

Figure S24. ^1H-^1H correlated spectroscopy spectra of Nle-RYLH in DMSO-d_6.

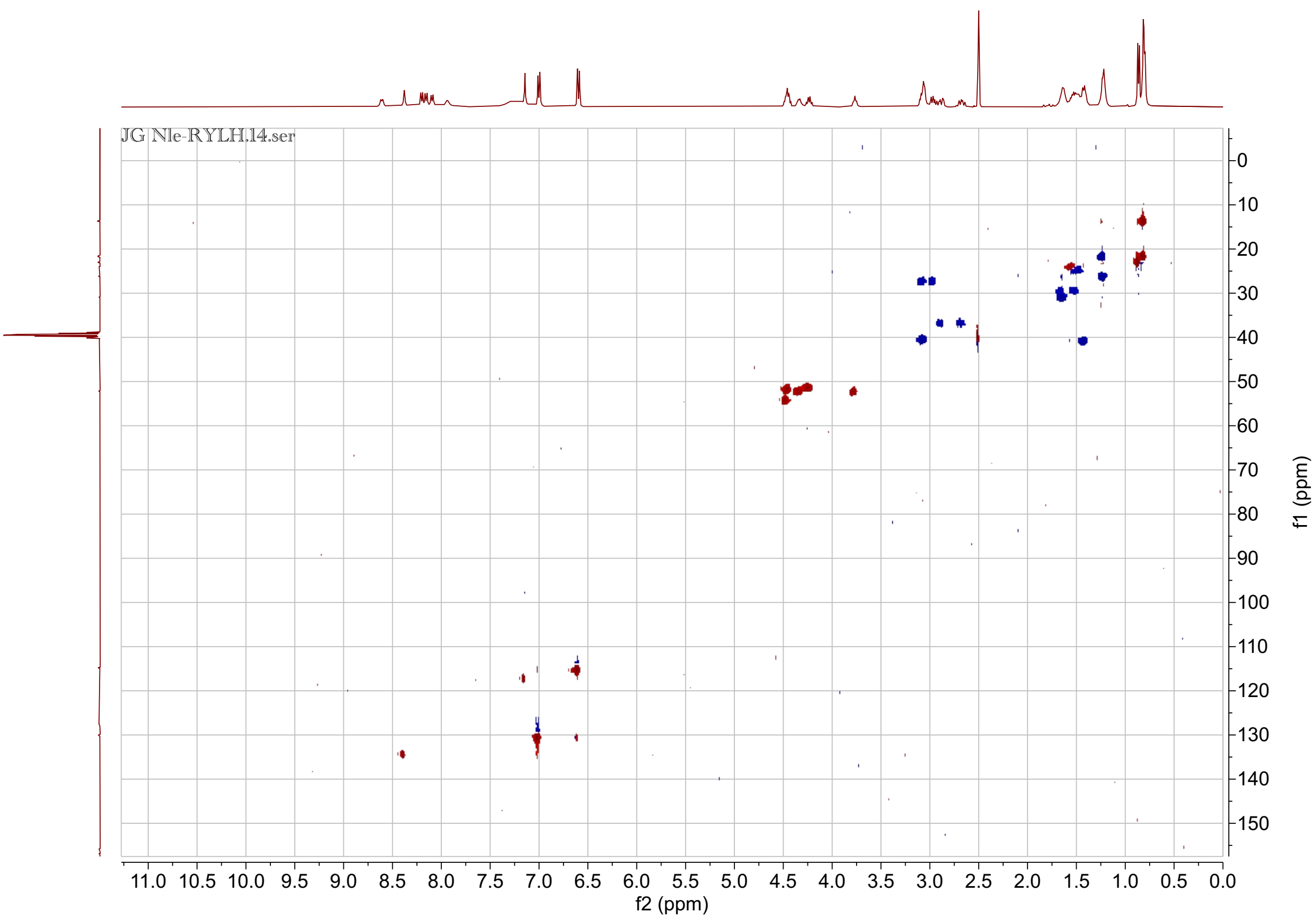

Figure S25. HSQC spectrum of Nle-RYLH in DMSO-d_6.

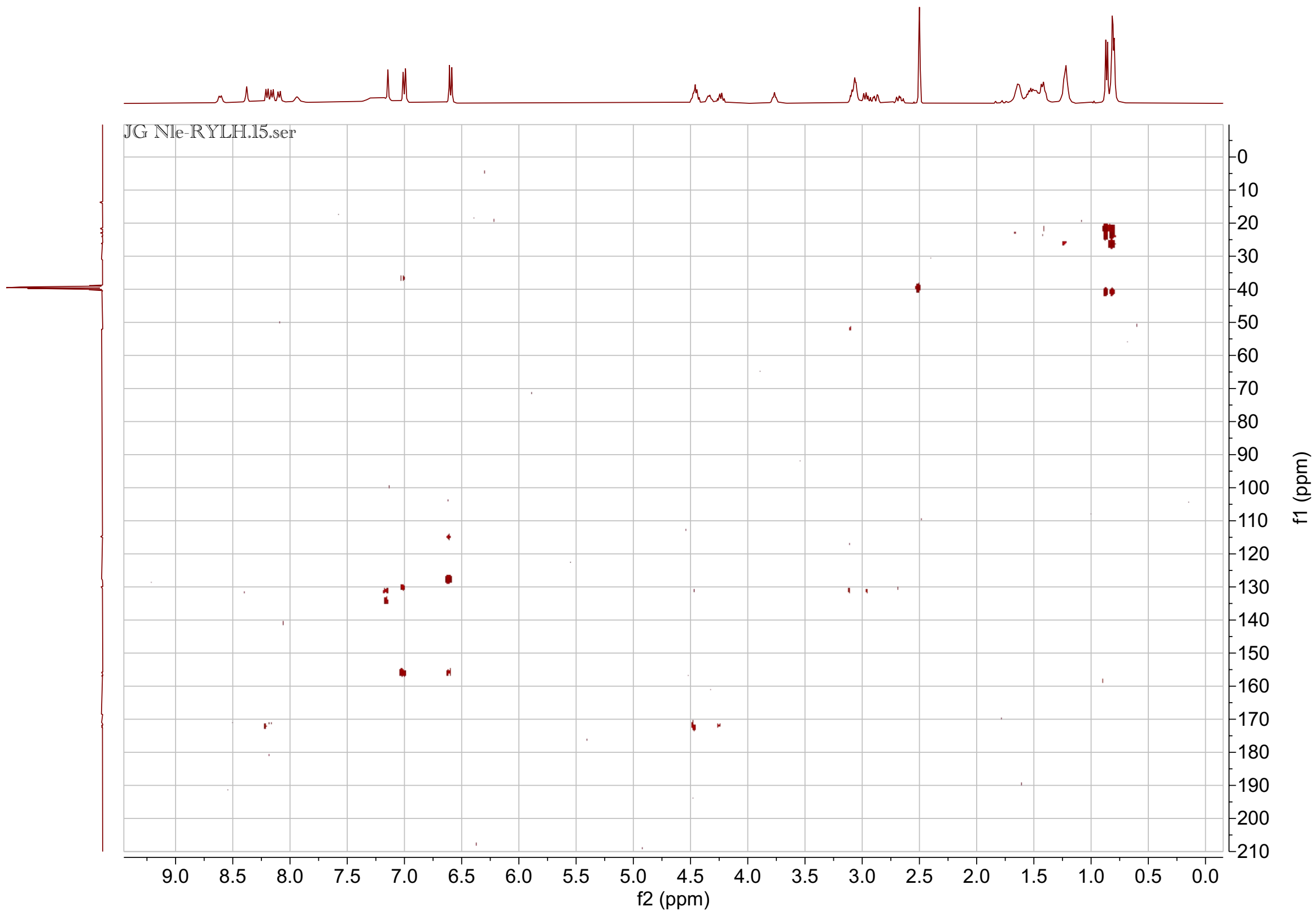

Figure S26. HMBC spectrum of Nle-RYLH in DMSO-d_6.

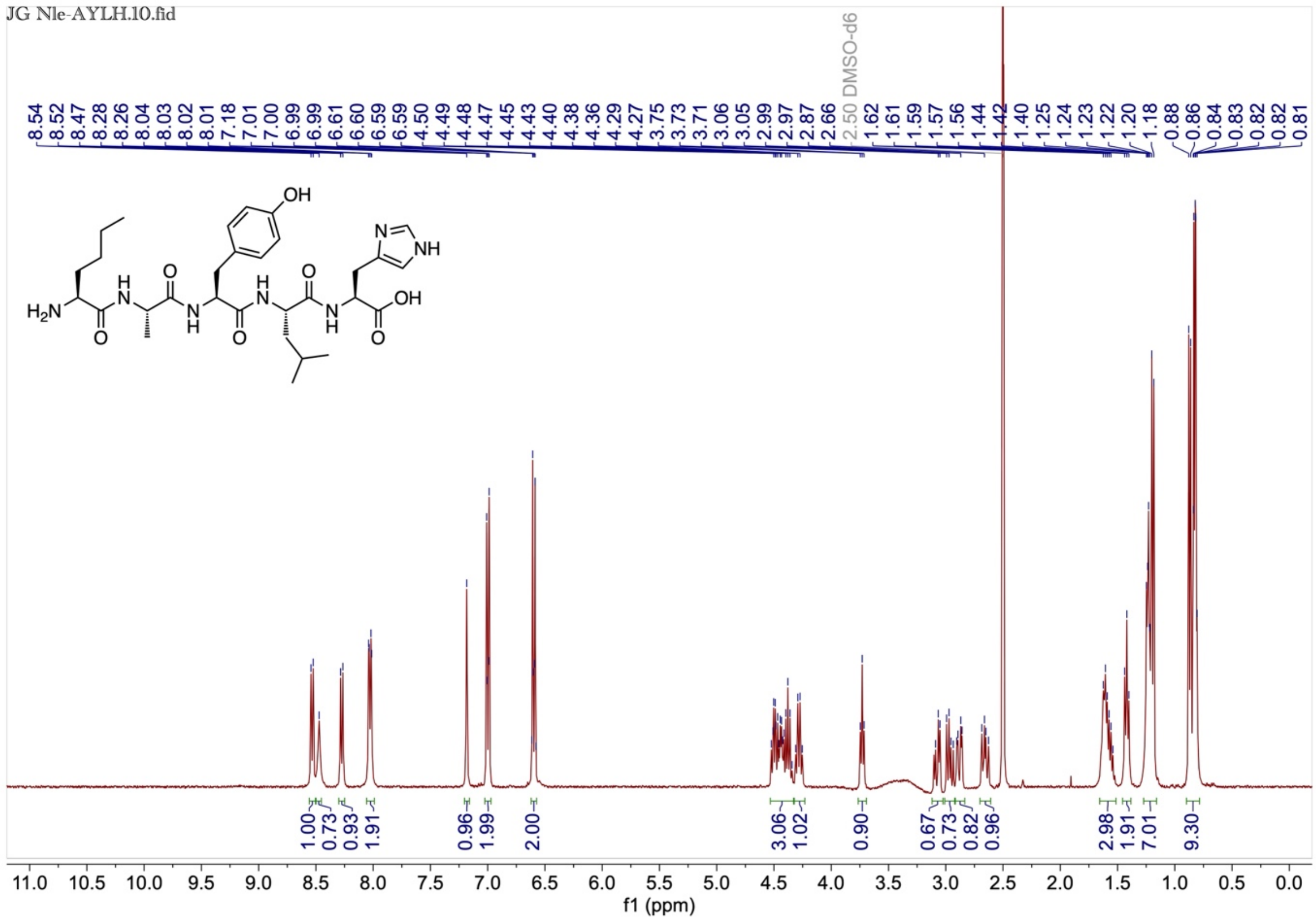

Figure S27. ^{1}H NMR of Nle-AYLH in DMSO-d$_6$.

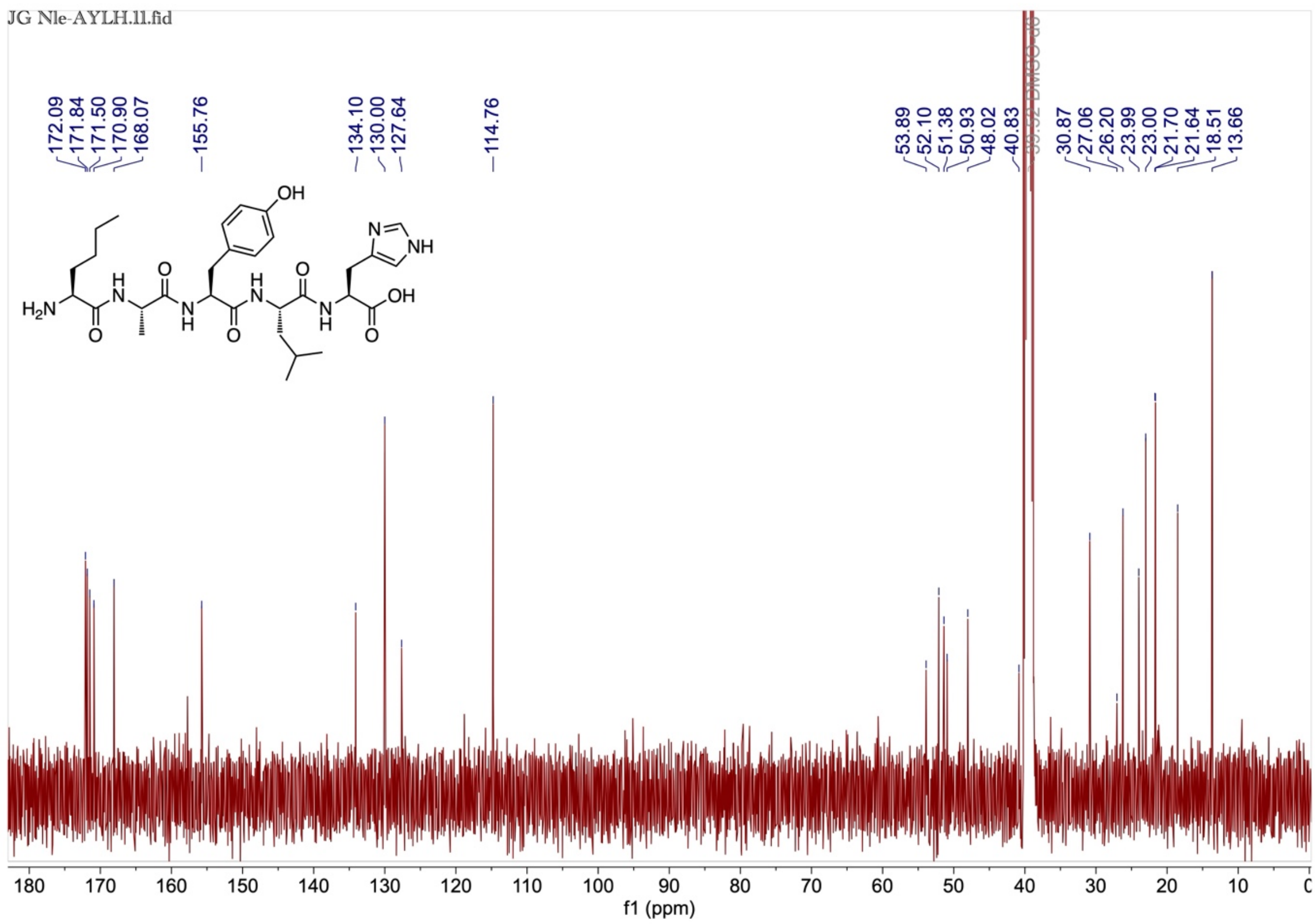

Figure S28. ^{13}C NMR of Nle-AYLH in DMSO-d$_6$.

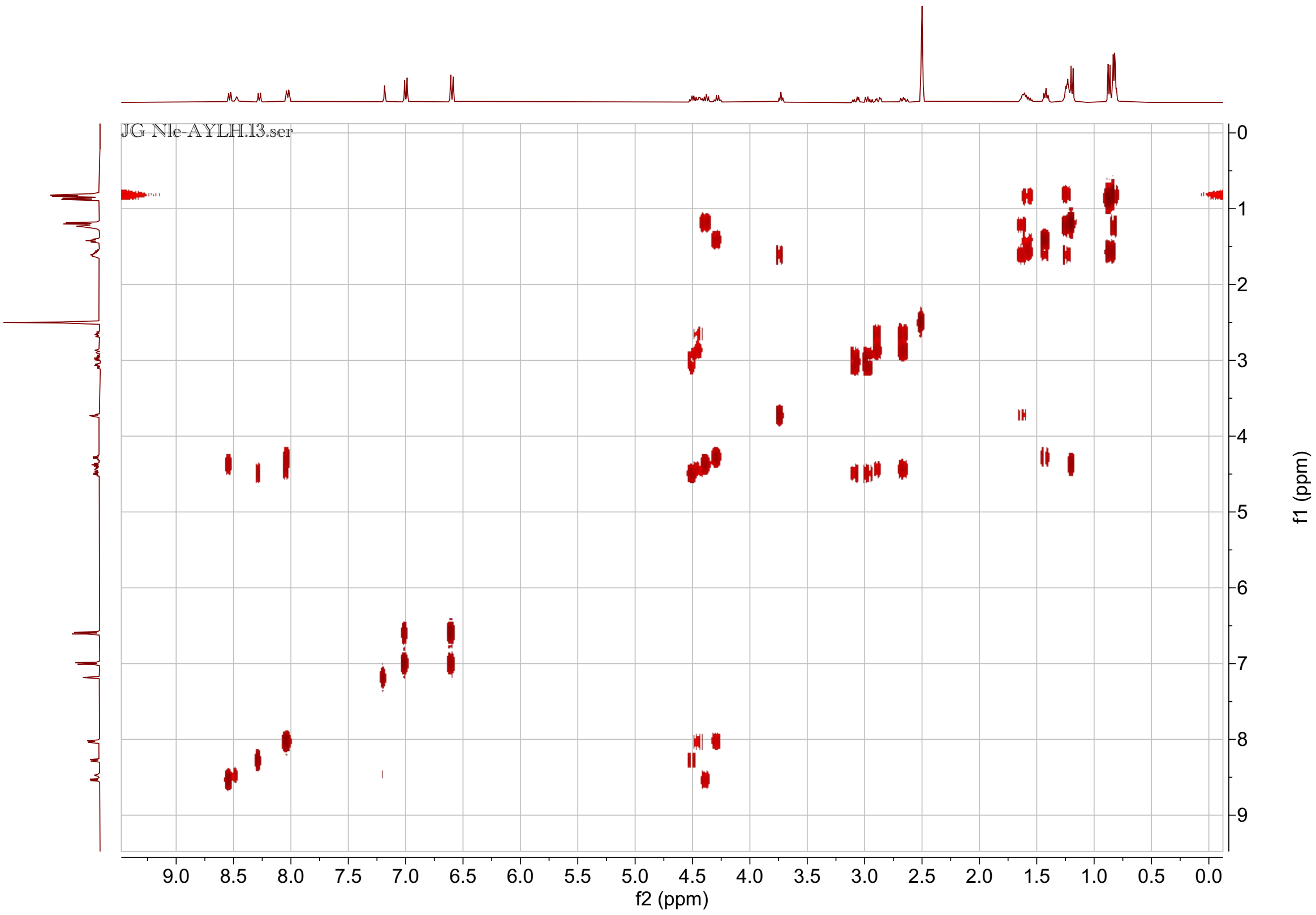

Figure S29. ^{1}H-^{1}H correlated spectroscopy spectrum of Nle-**A**YLH in DMSO-d_6.

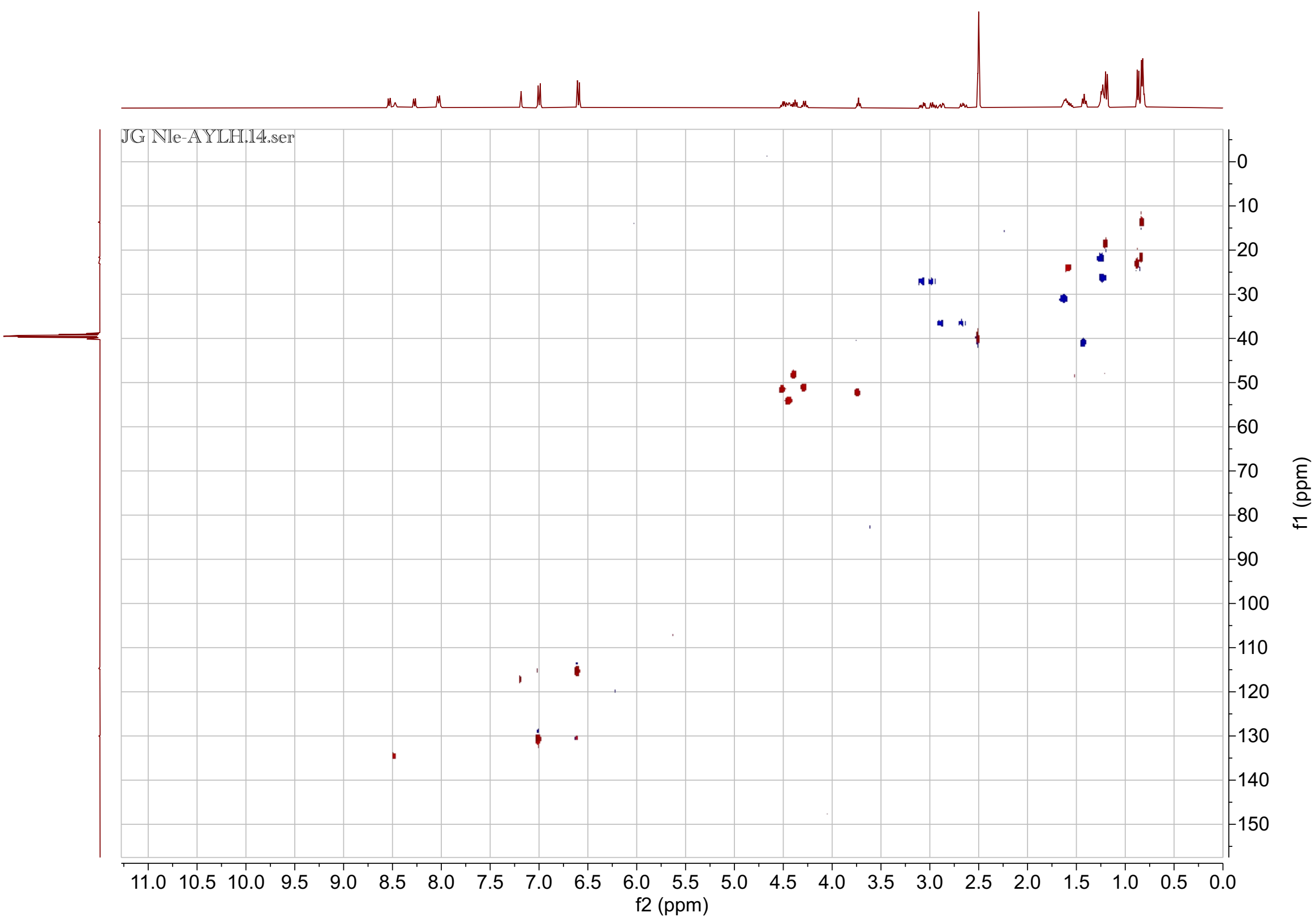

Figure S30. HSQC spectrum of Nle-**A**YLH in DMSO-d_6.

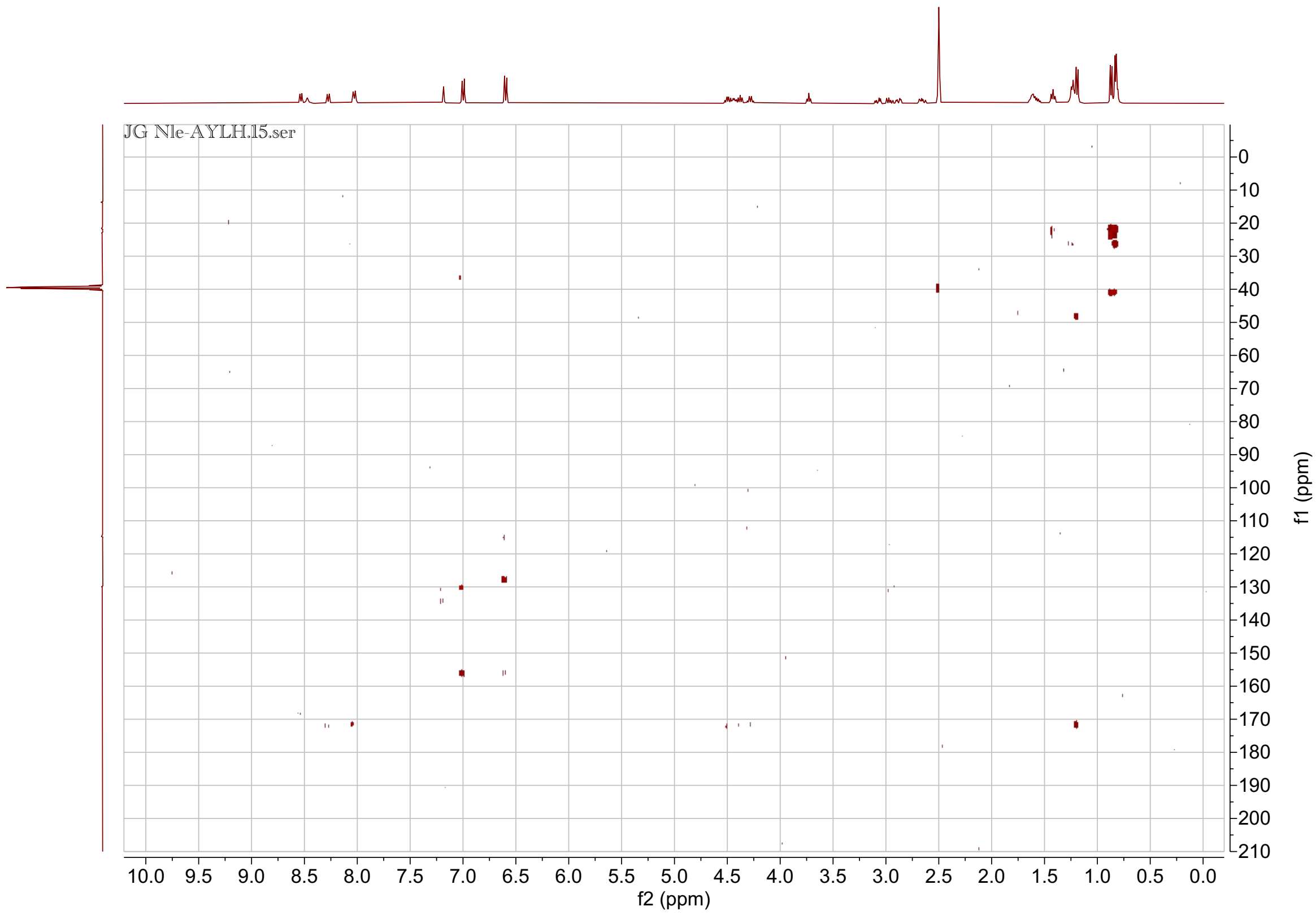

Figure S31. HMBC spectrum of Nle-**A**YLH in DMSO-d$_6$.

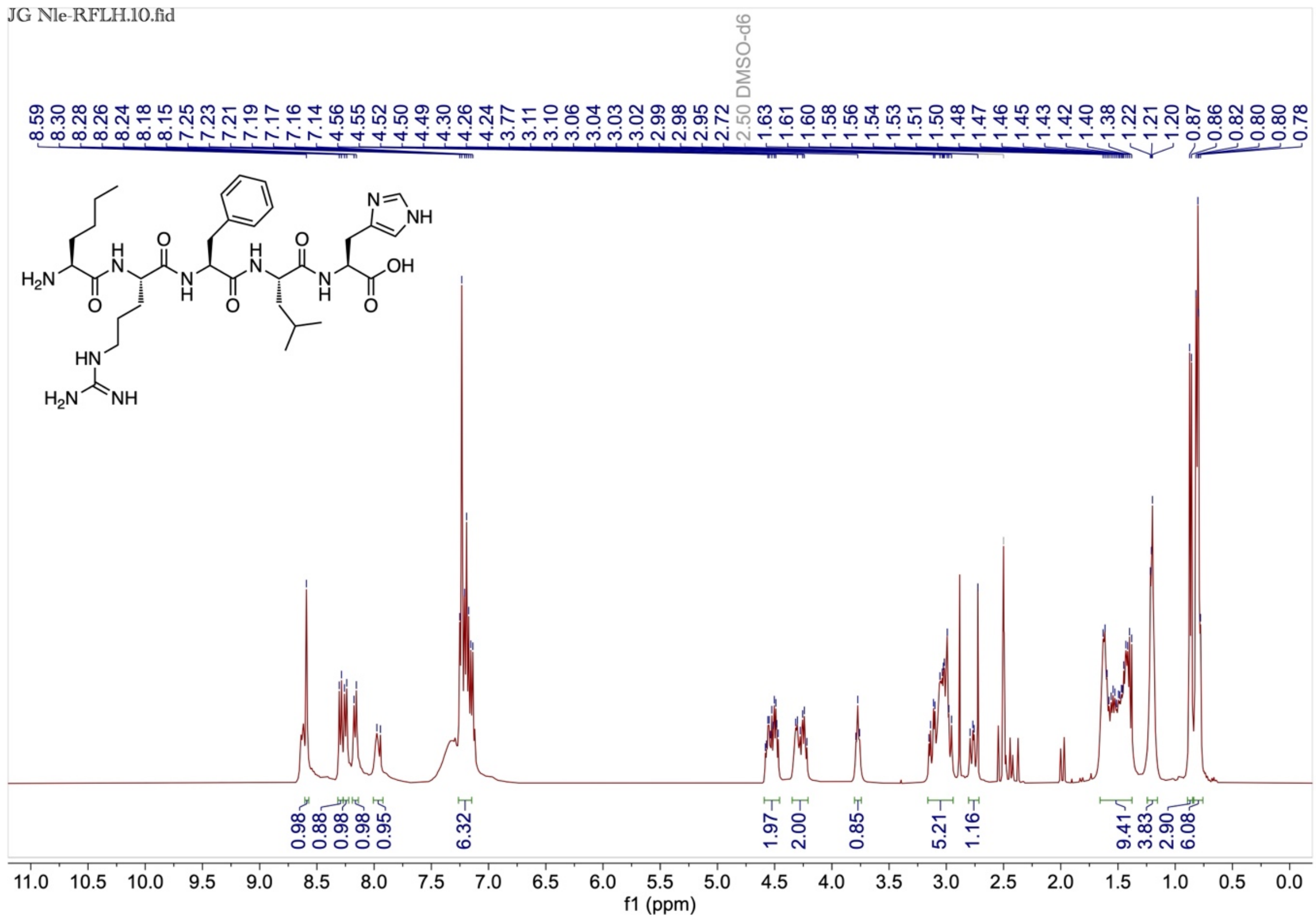

Figure S32. ^{1}H NMR of Nle-R**F**LH in DMSO-d_6.

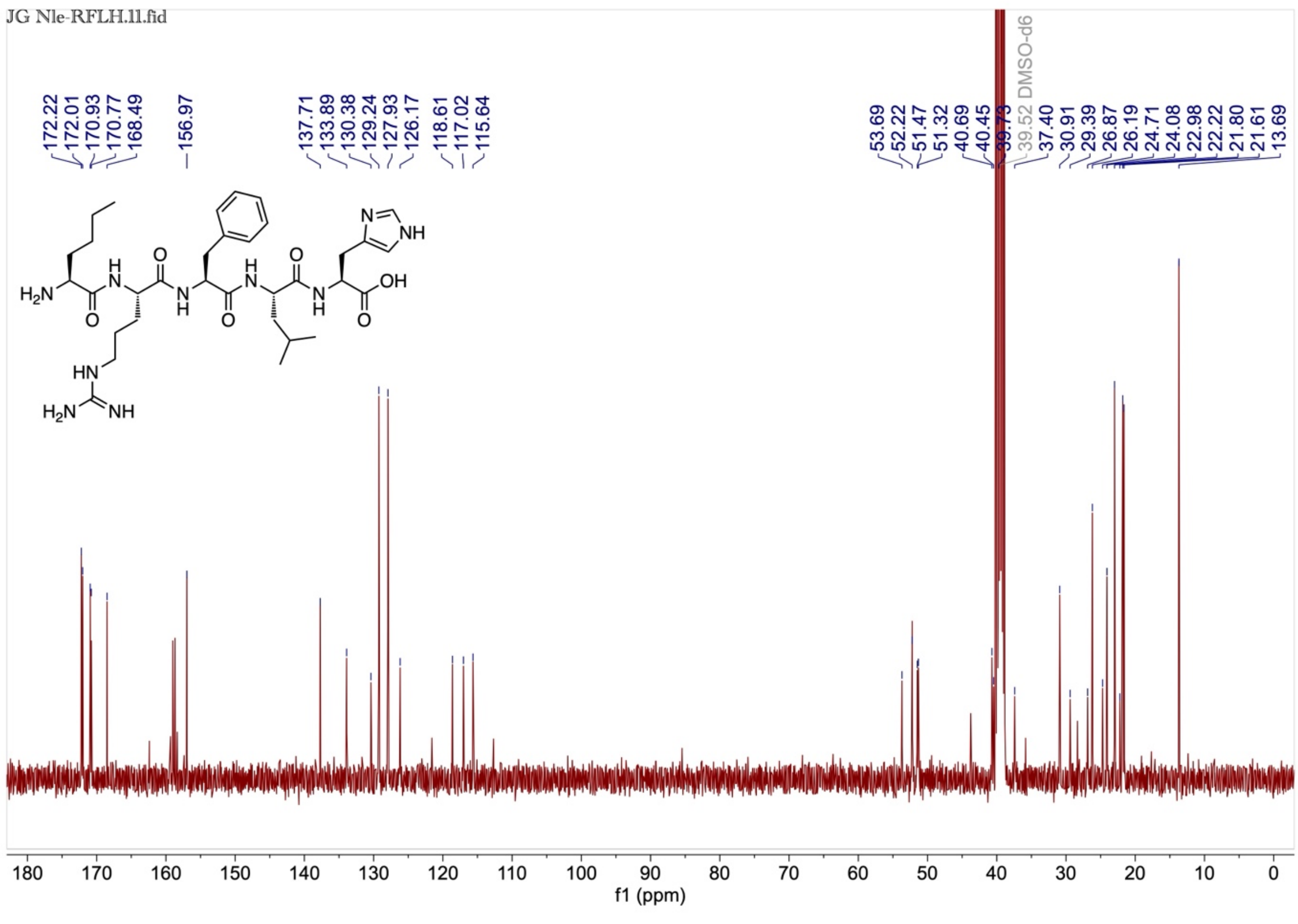

Figure S33. ^{13}C NMR of Nle-R**F**LH in DMSO-d_6.

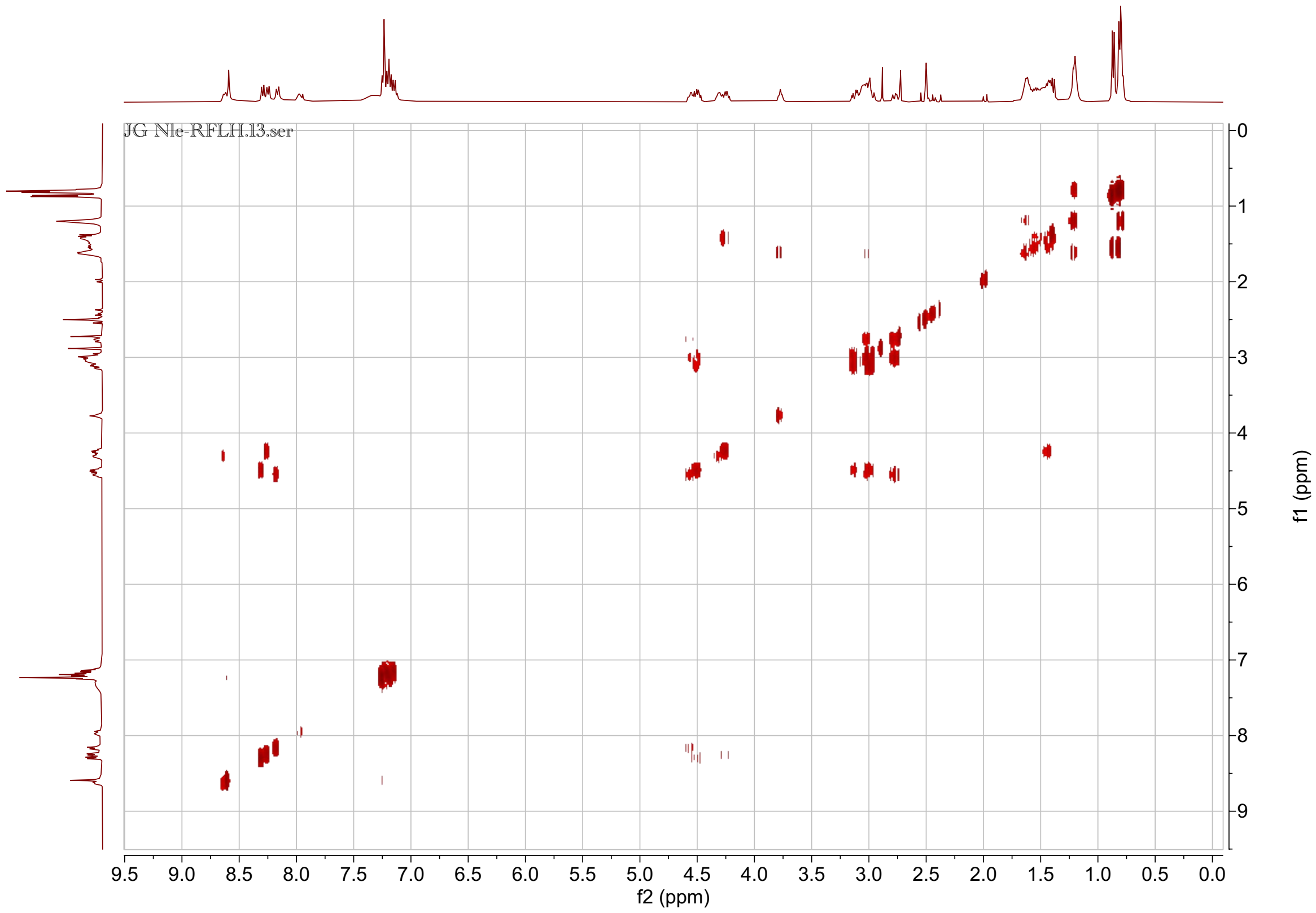

Figure S34. ^{1}H-^{1}H correlated spectroscopy spectrum of Nle-R**F**LH in DMSO-d6.

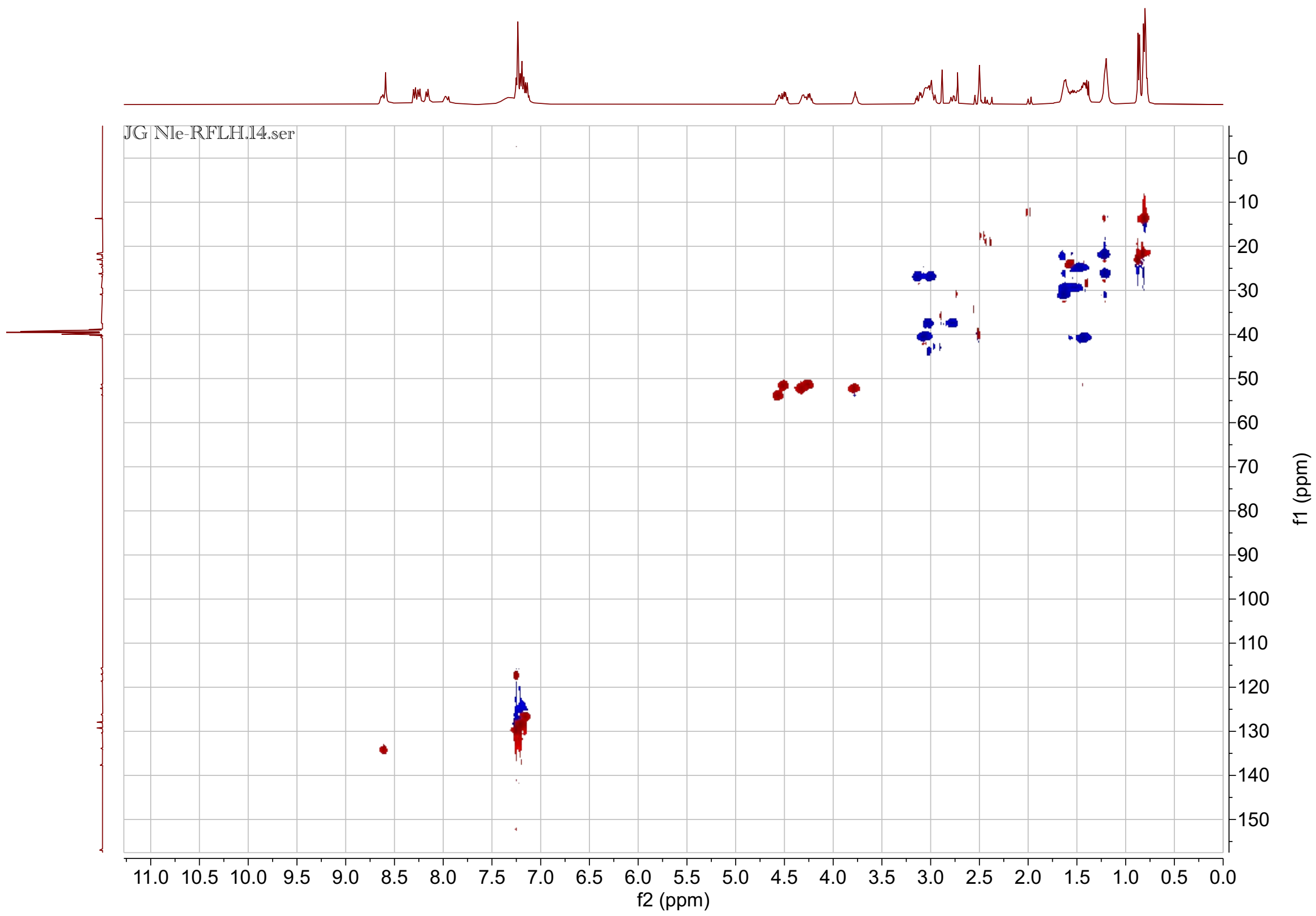

Figure S35. HSQC spectrum of Nle-R**F**LH in DMSO-d6.

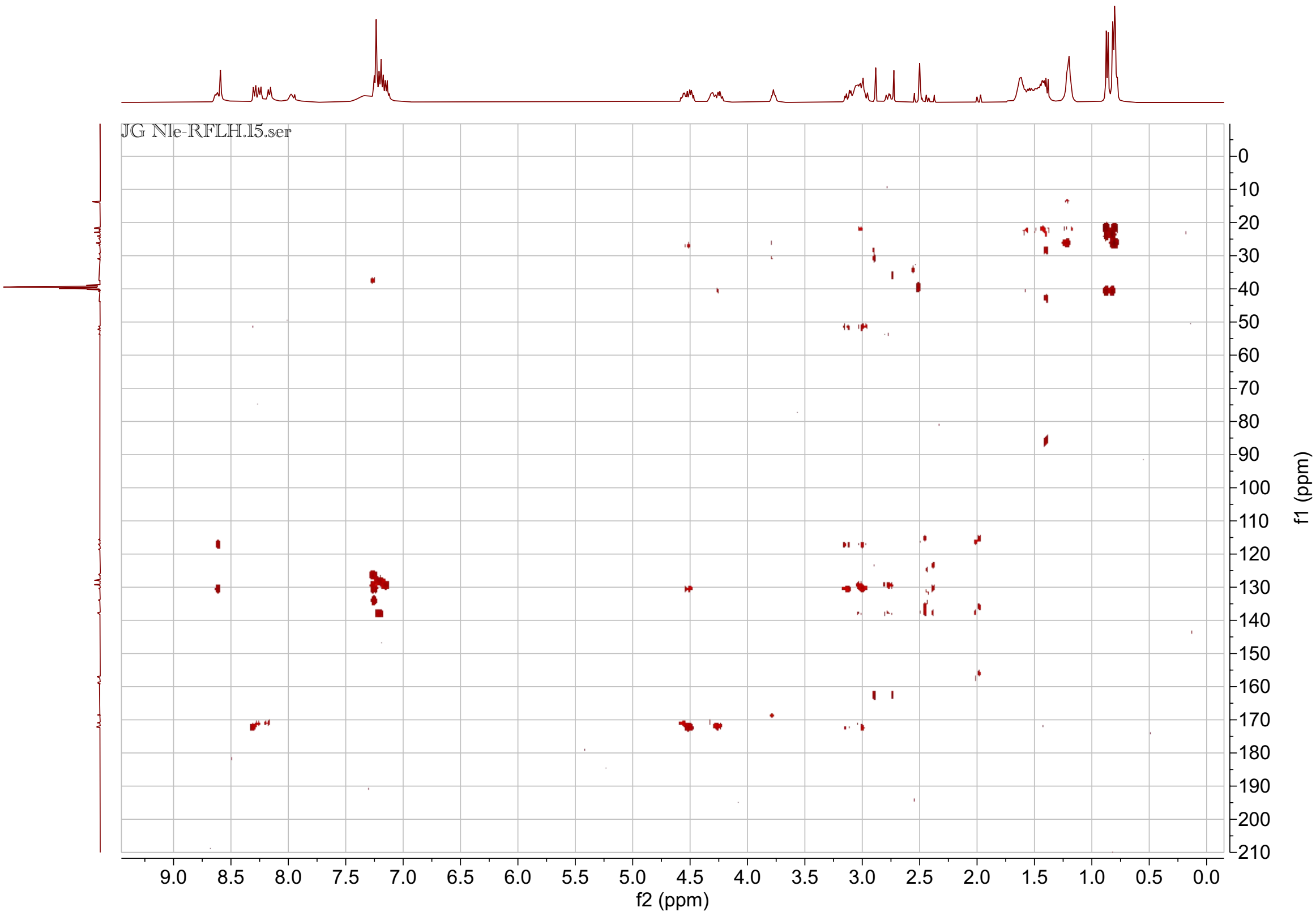

Figure S36. HMBC spectrum of Nle-R**F**LH in DMSO-d_6.

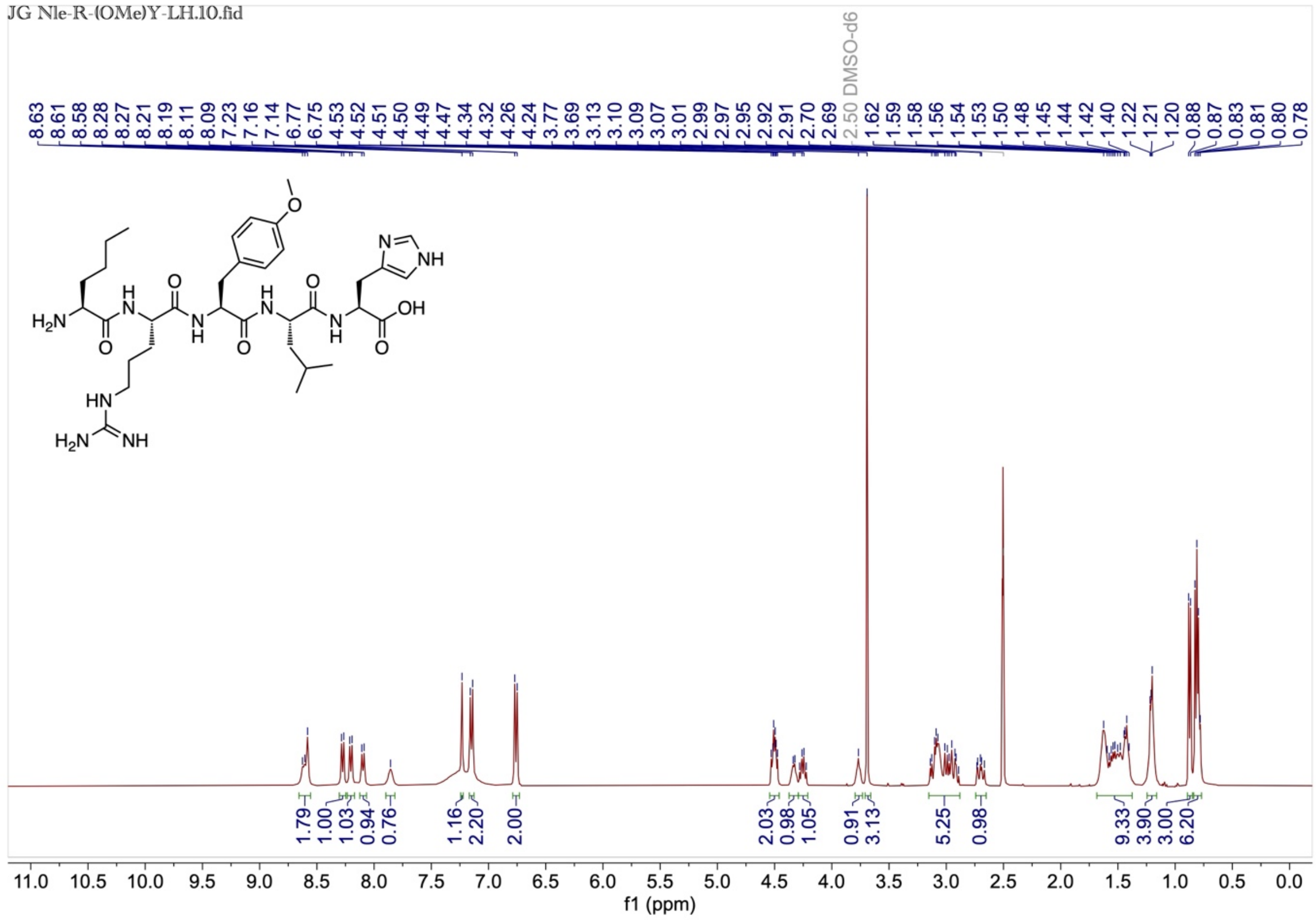

Figure S37. ^{1}H NMR of Nle-R-**(OMe)Tyr**-LH in DMSO-d$_6$.

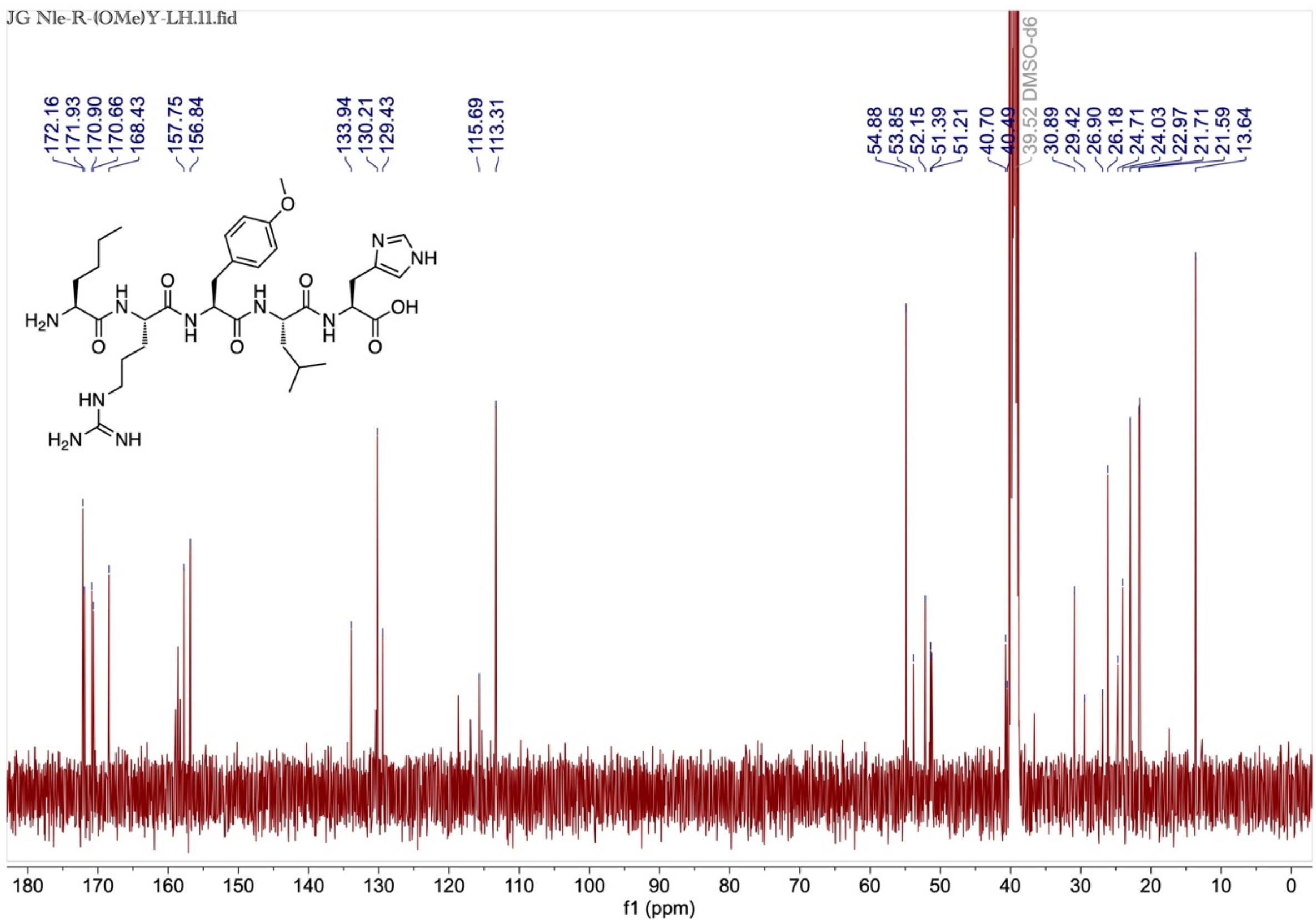

Figure S38. ^{13}C NMR of Nle-R-**(OMe)Tyr**-LH in DMSO-d$_6$.

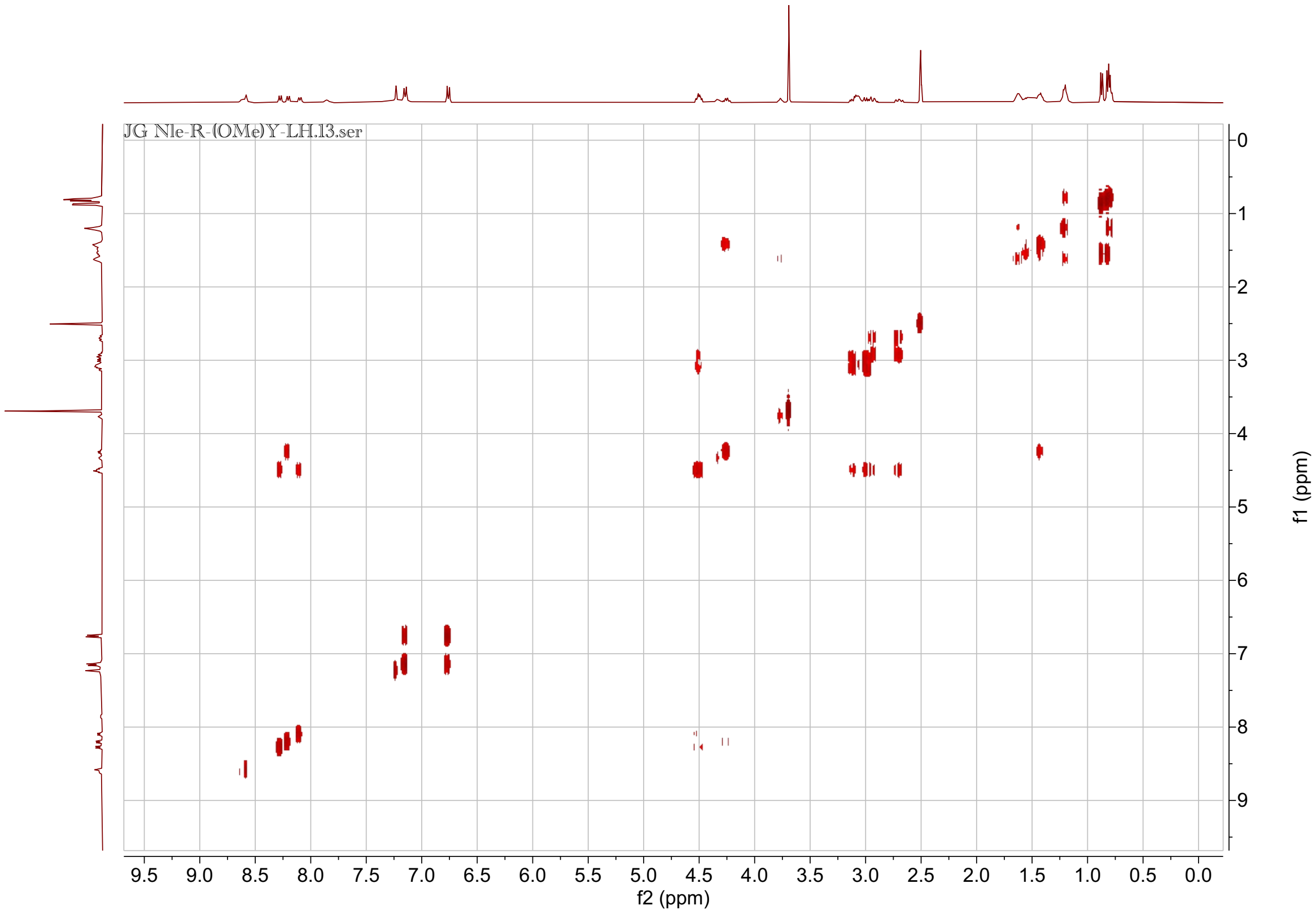

Figure S39. ^{1}H-^{1}H correlated spectroscopy spectrum of Nle-R-**(OMe)Tyr**-LH in DMSO-d$_6$.

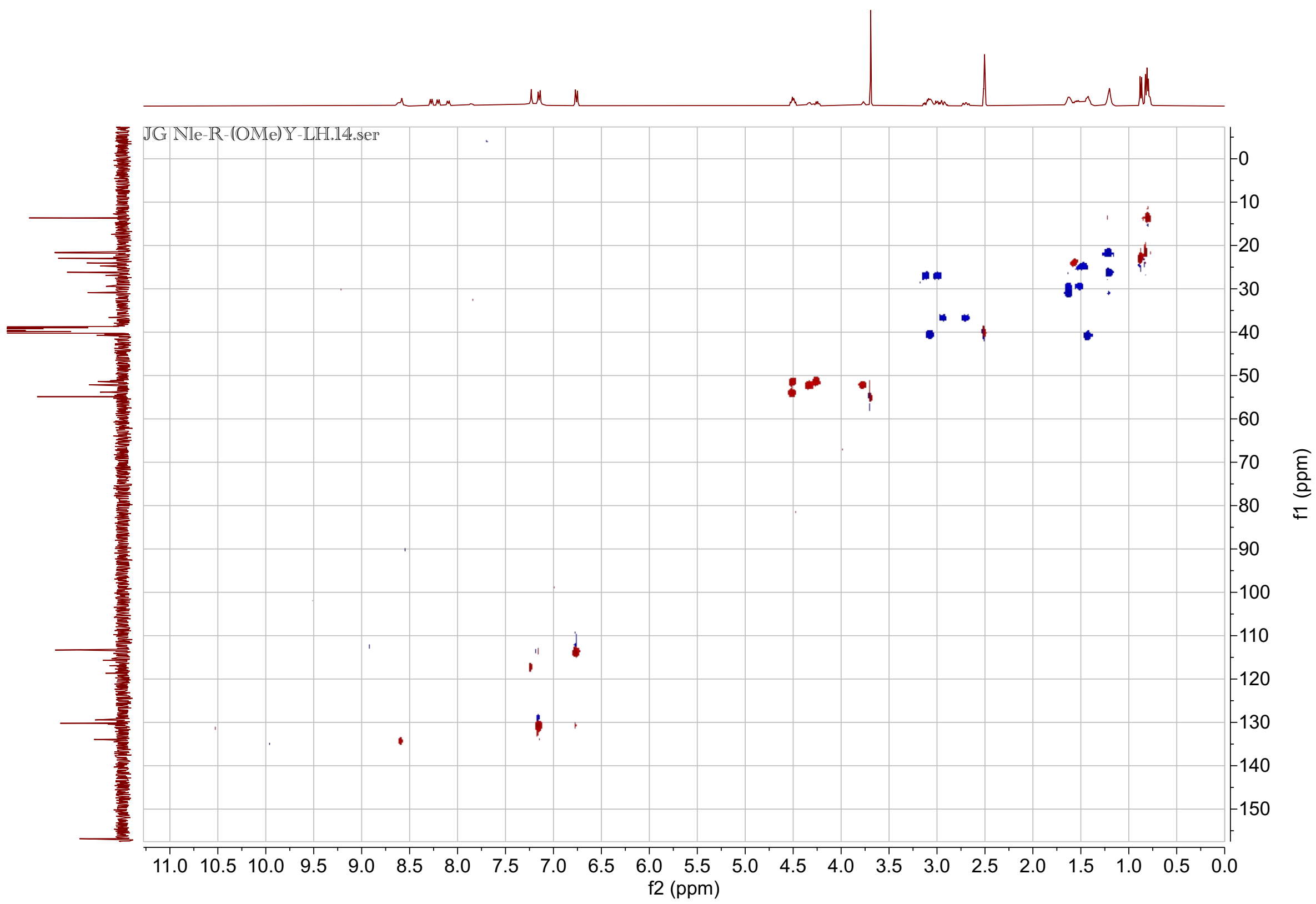

Figure S40. HSQC spectrum of Nle-R-**(OMe)Tyr**-LH in DMSO-d$_6$.

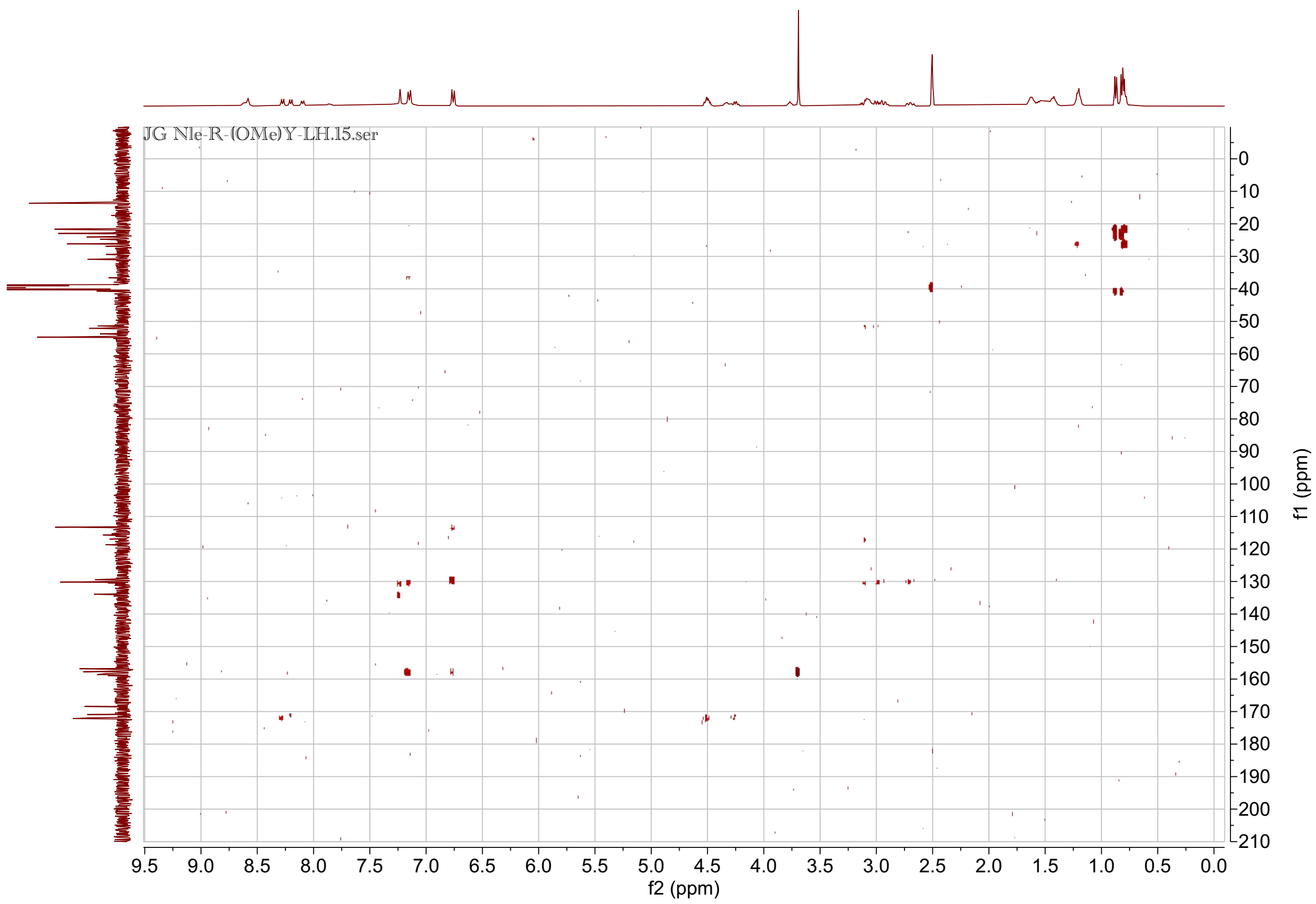

Figure S41. HMBC spectrum of Nle-R-(OMe)Tyr-LH in DMSO-d_6.

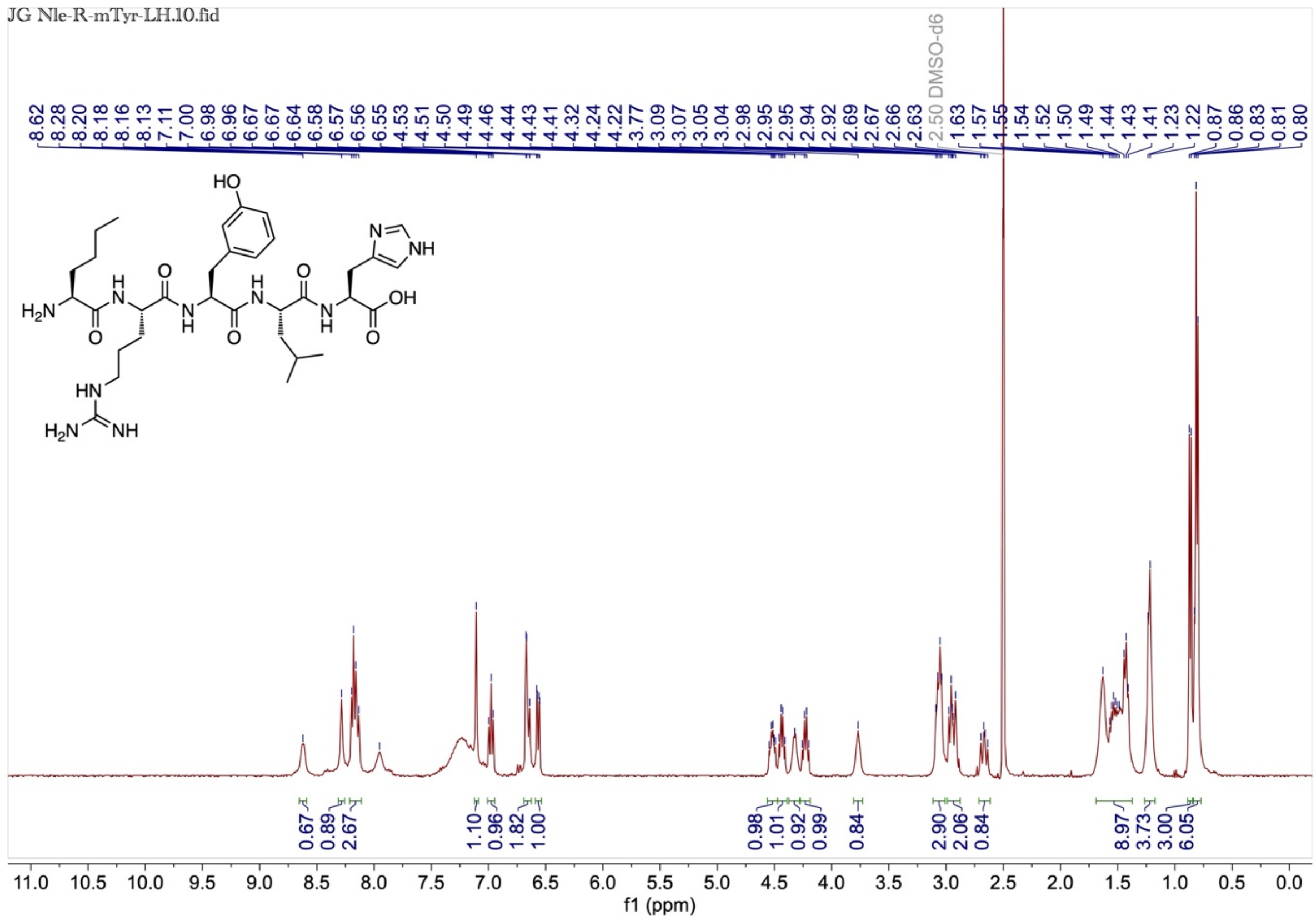

Figure S42. ^{1}H NMR of Nle-R-**mTyr**-LH in DMSO-d_6.

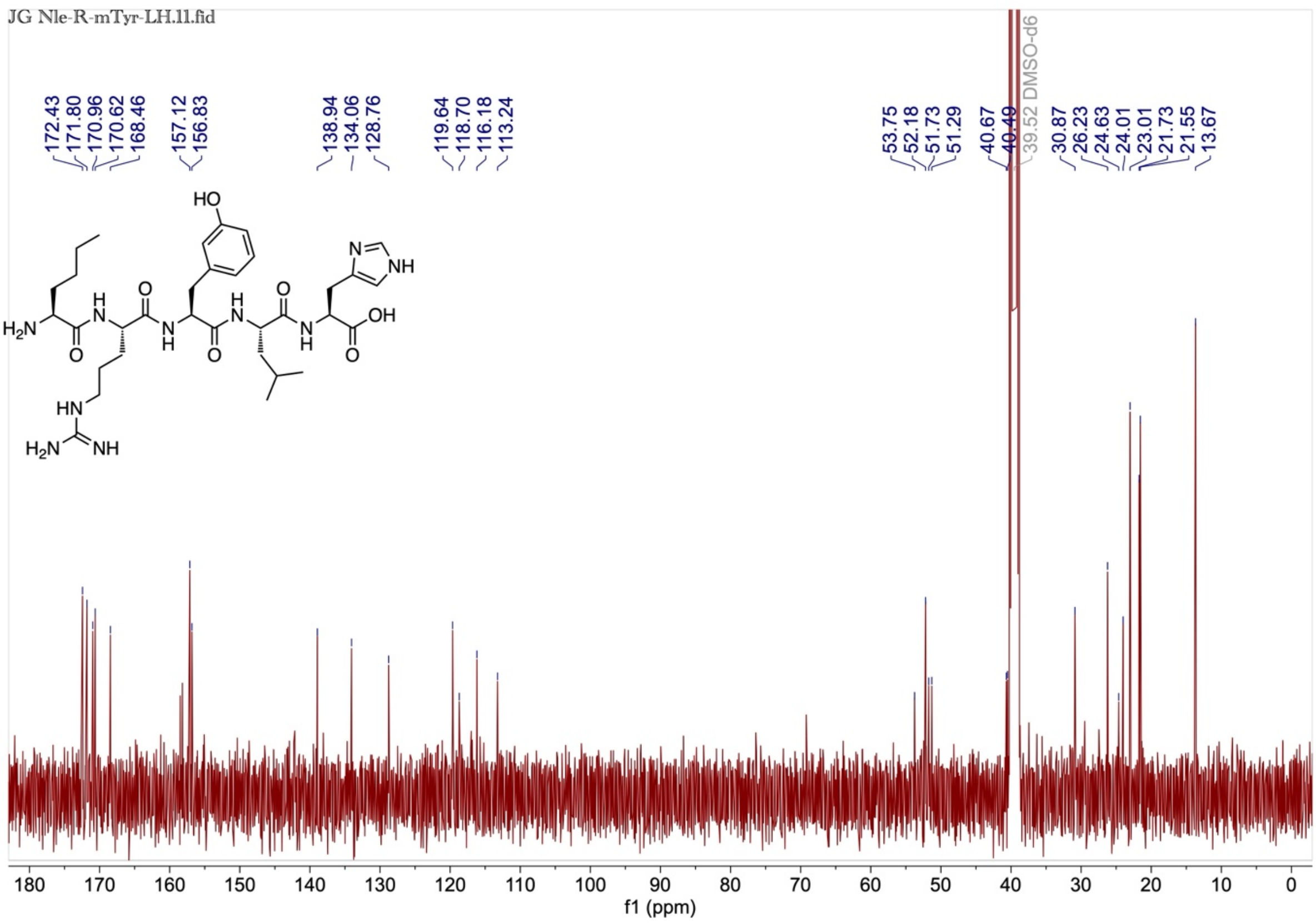

Figure S43. ^{13}C NMR of Nle-R-mTyr-LH in DMSO-d_6.

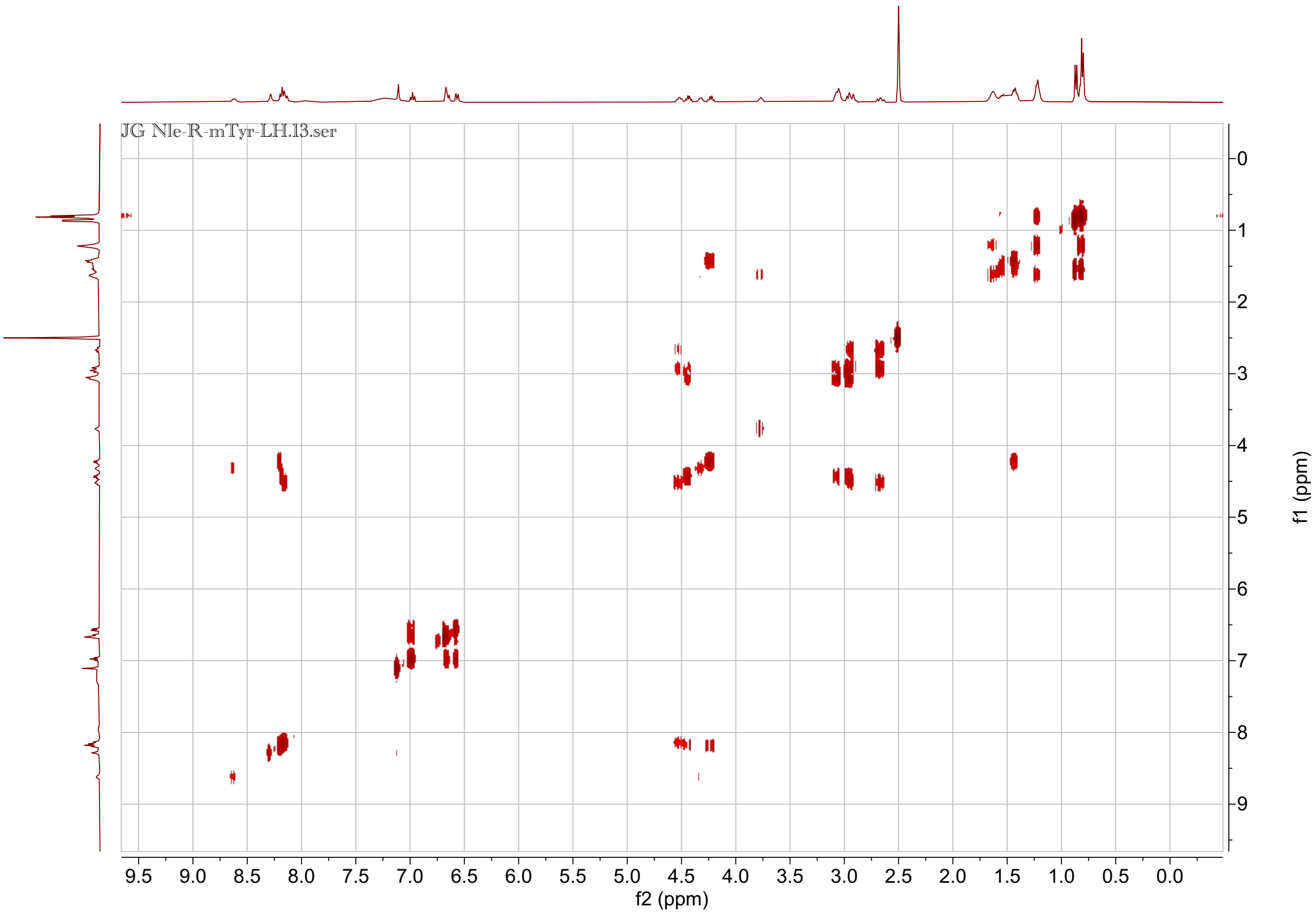

Figure S44. ^{1}H-^{1}H correlated spectroscopy spectrum of Nle-R-**mTyr**-LH in DMSO-d6.

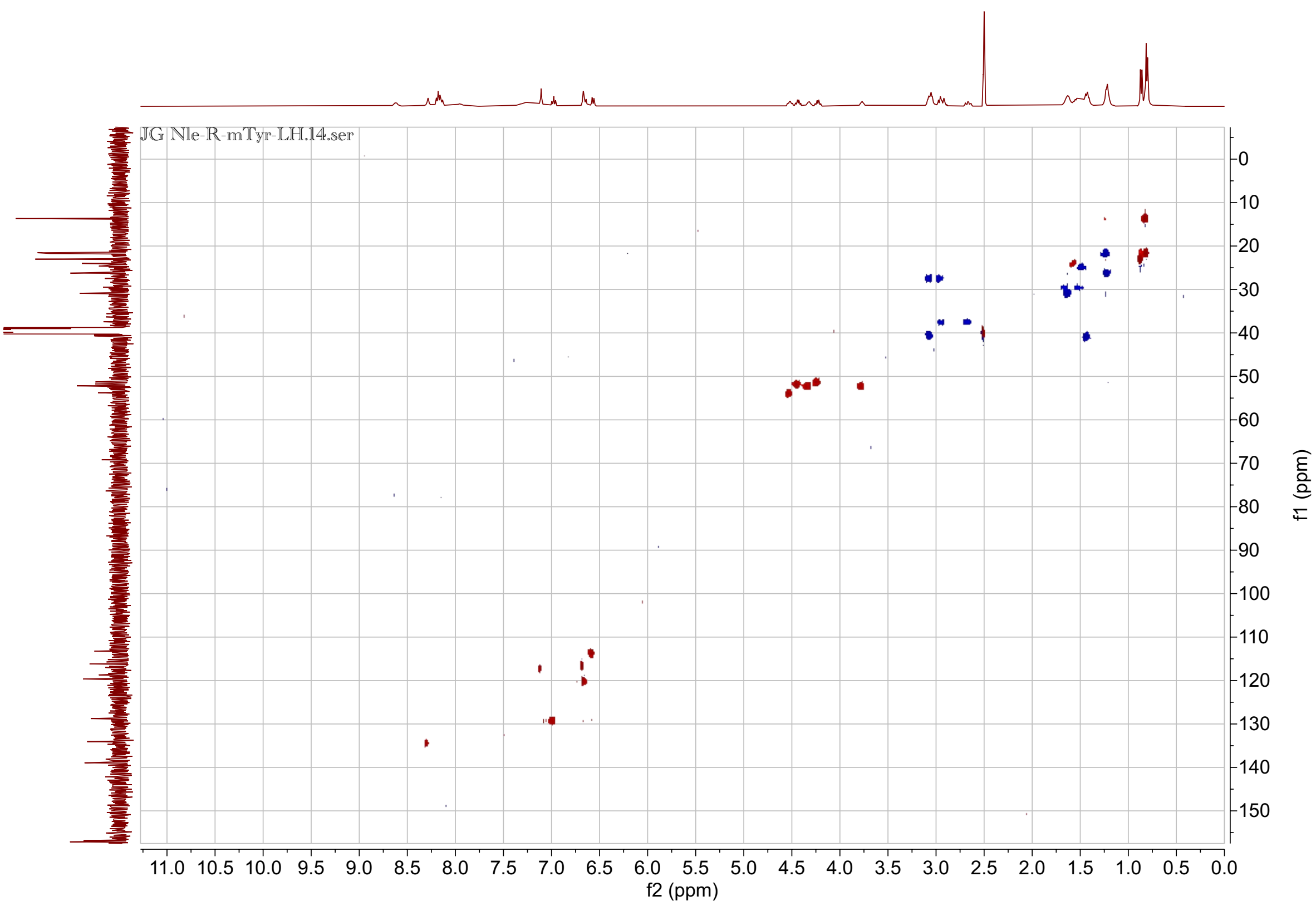

Figure S45. HSQC spectrum of Nle-R-**mTyr**-LH in DMSO-d6.

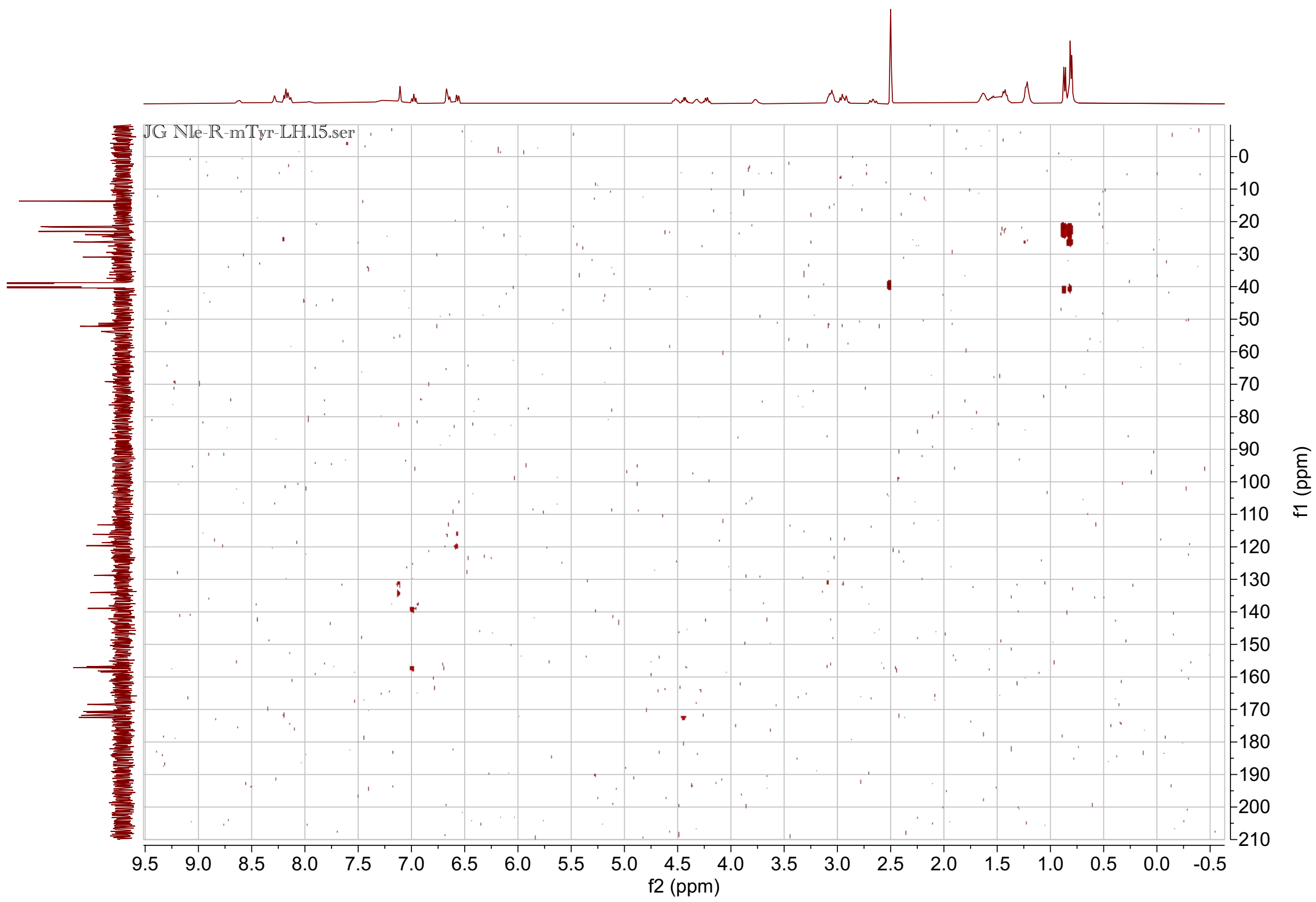

Figure S46. HMBC spectrum of Nle-R-**mTyr**-LH in DMSO-d_6.

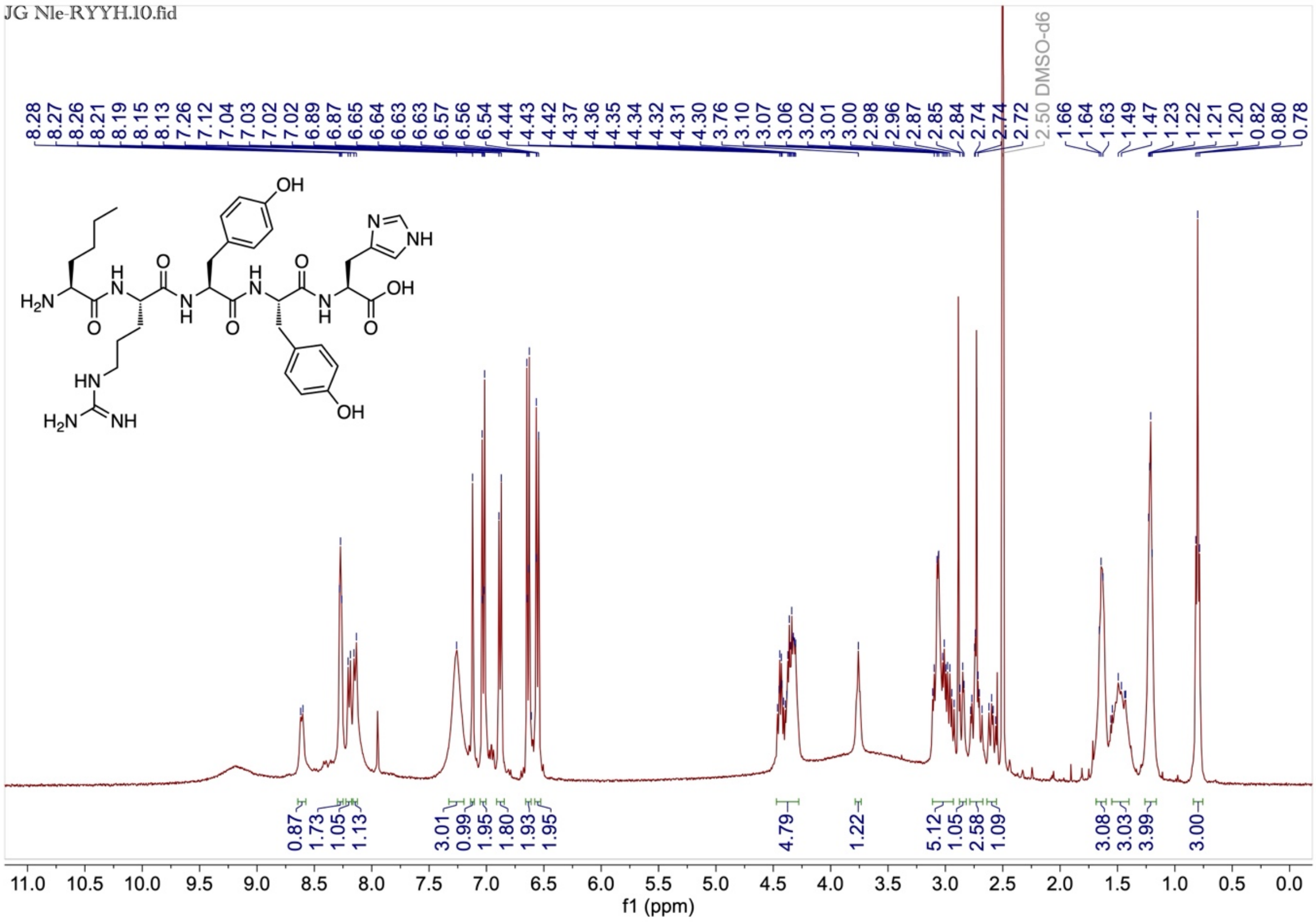

Figure S47. ^{1}H NMR of Nle-RY**Y**H in DMSO-d$_6$.

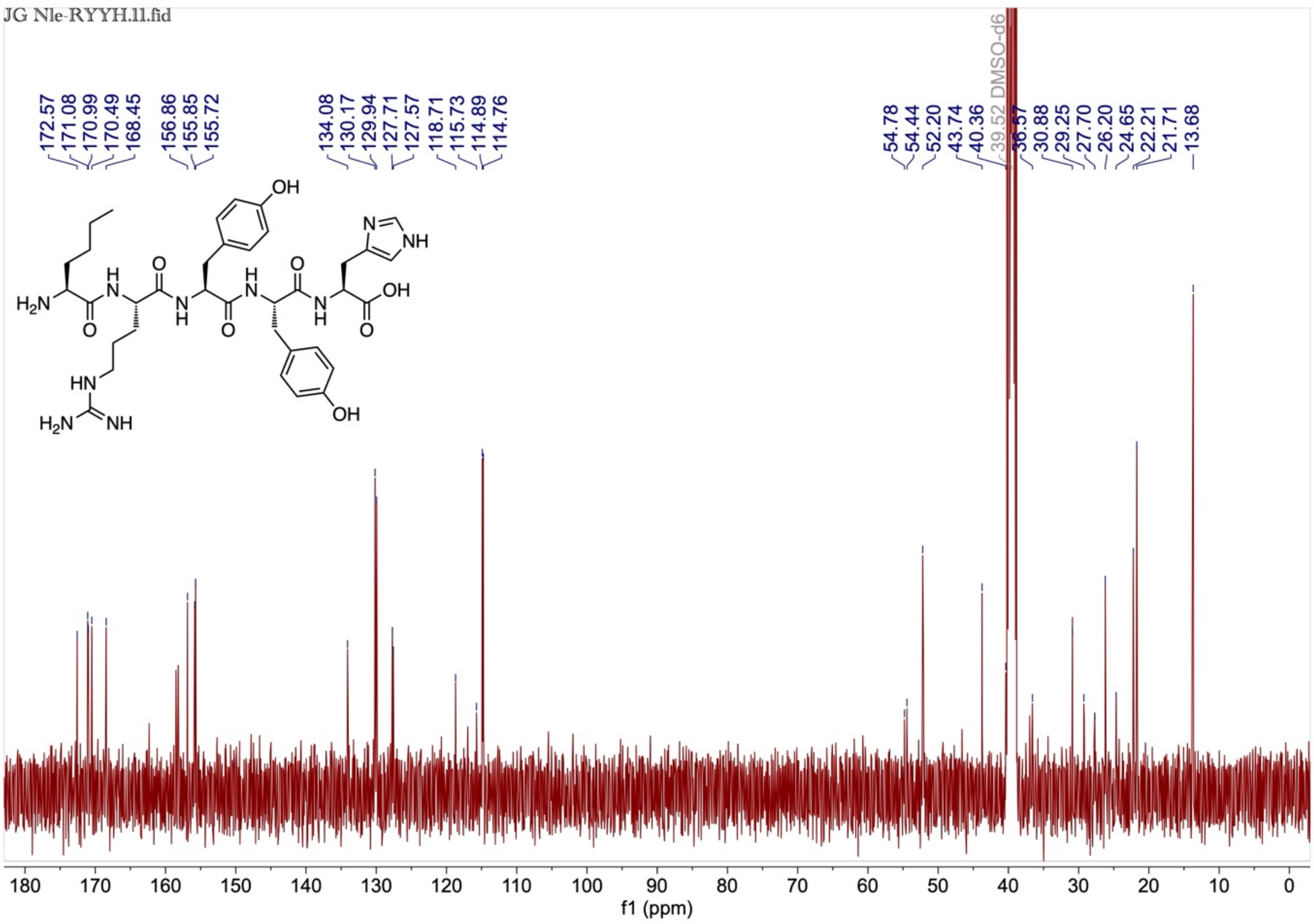

Figure S48. ^{13}C NMR of Nle-RY**Y**H in DMSO-d$_6$.

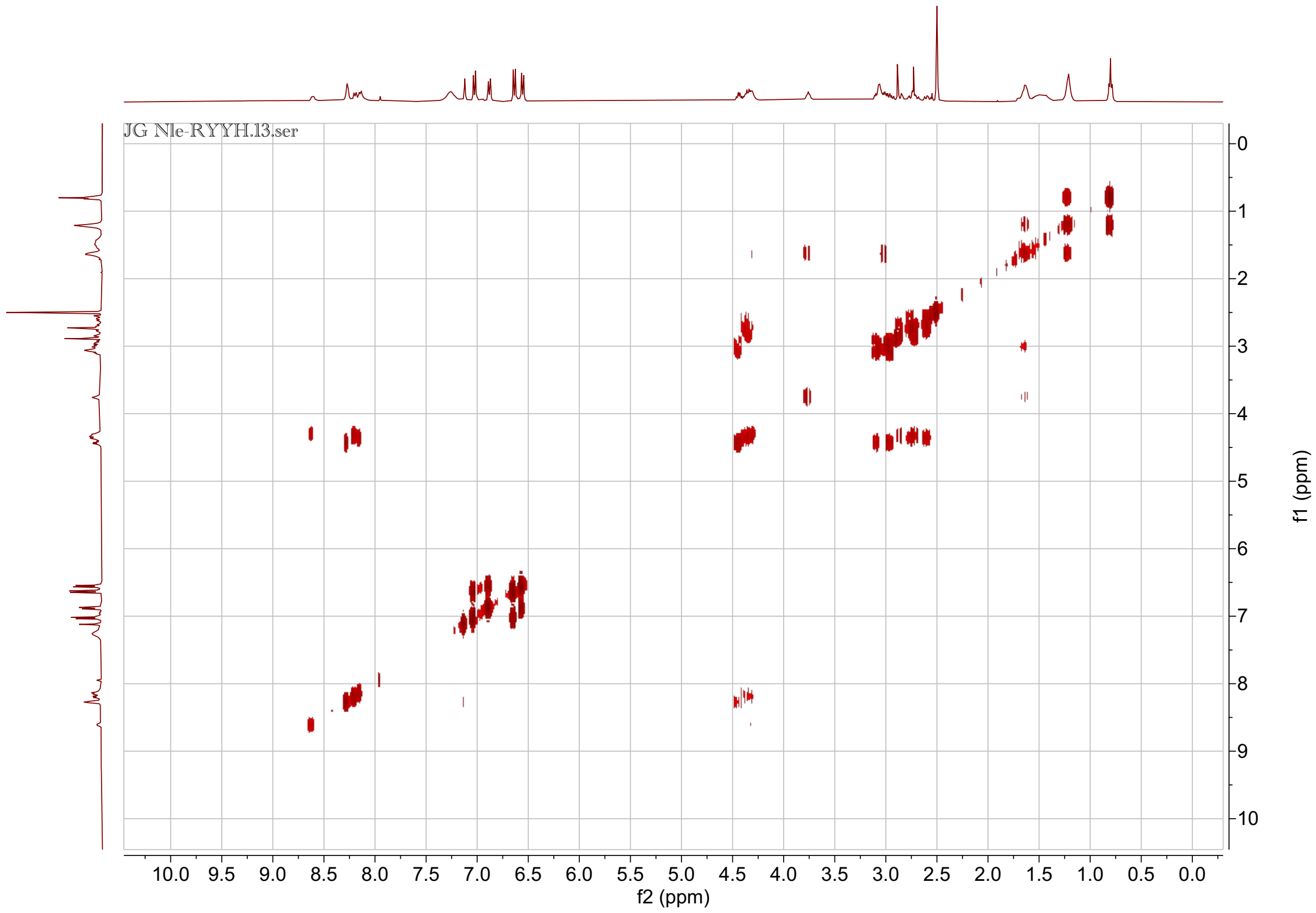

Figure S49. ^{1}H-^{1}H correlated spectroscopy spectrum of Nle-RY**Y**H in DMSO-d_6.

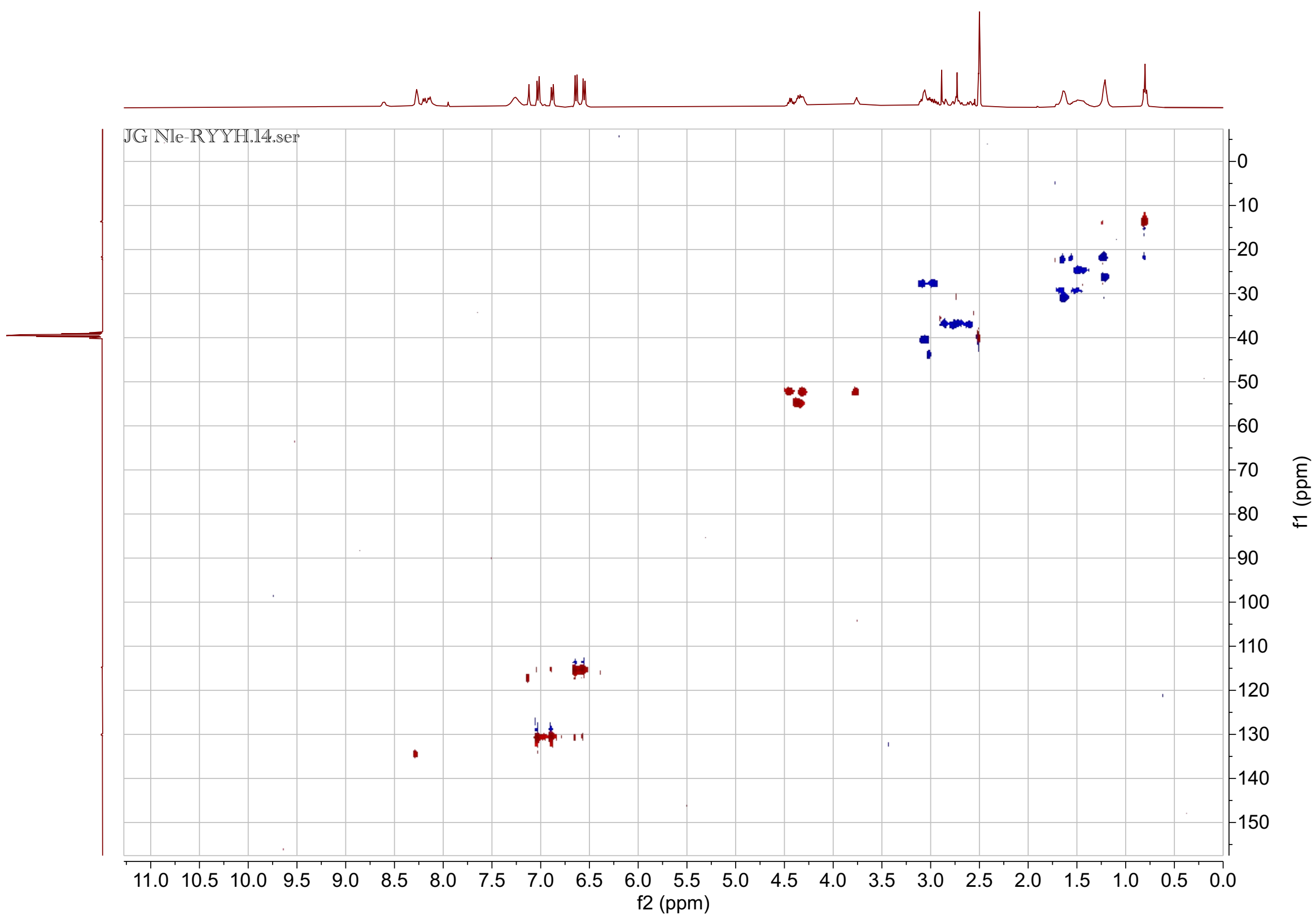

Figure S50. HSQC spectrum of Nle-RY**Y**H in DMSO-d_6.

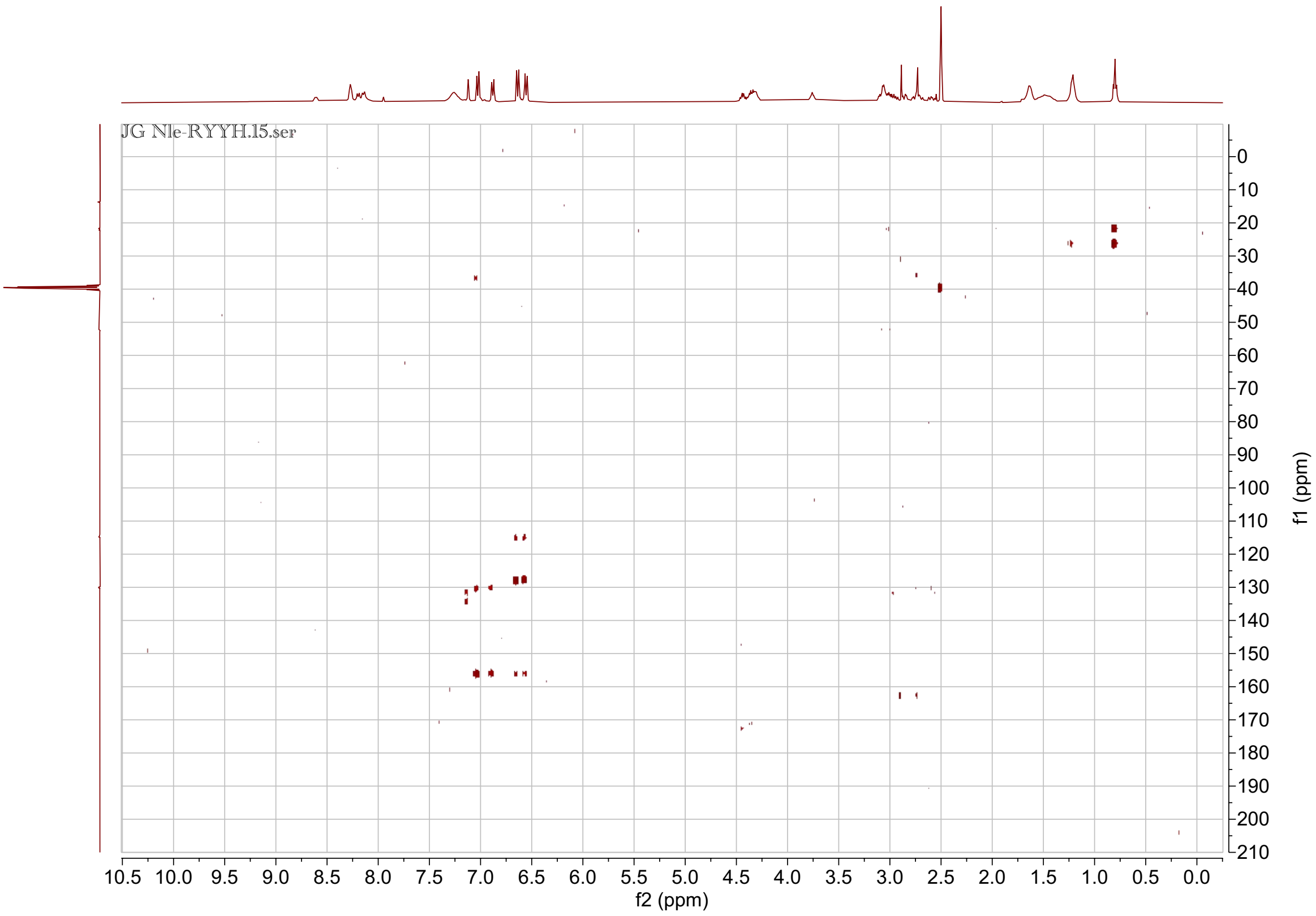

Figure S51. HMBC spectrum of Nle-RY**Y**H in DMSO-d_6.

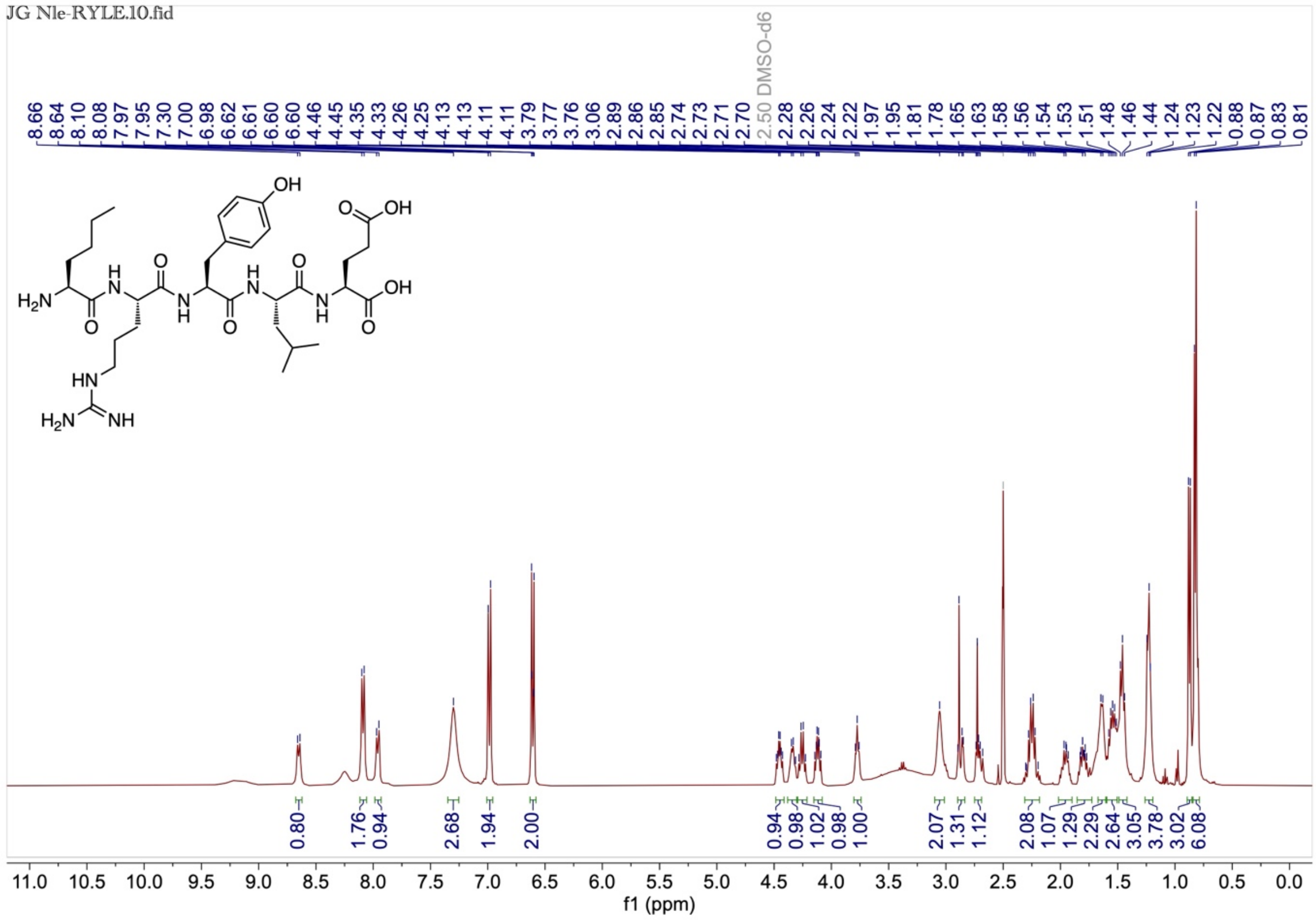

Figure S52. ^{1}H NMR of Nle-RYL**E** in DMSO-d_6.

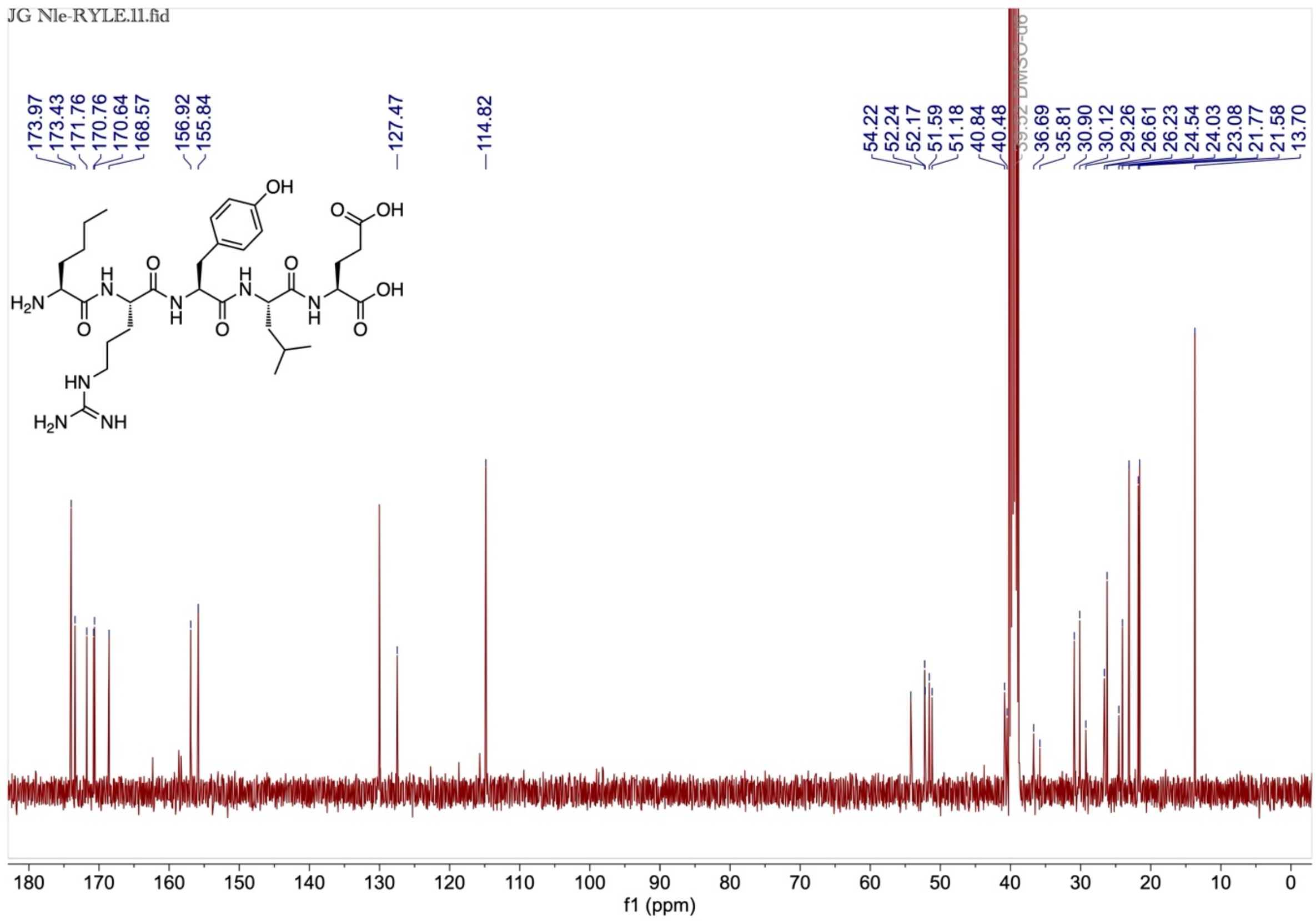

Figure S53. ^{13}C NMR of Nle-RYL**E** in DMSO-d_6.

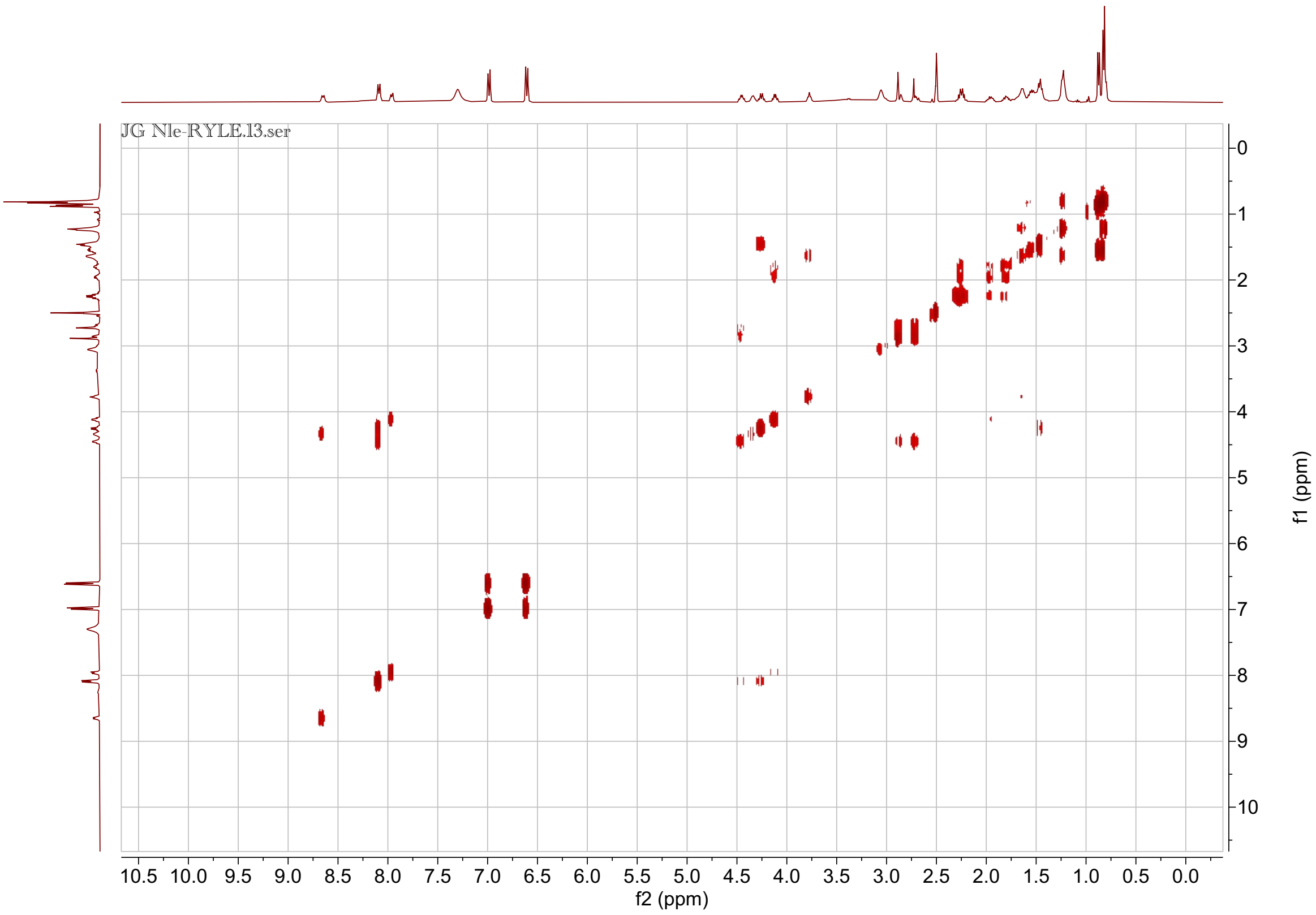

Figure S54. ^{1}H-^{1}H correlated spectroscopy spectrum of Nle-RYL**E** in DMSO-d_6.

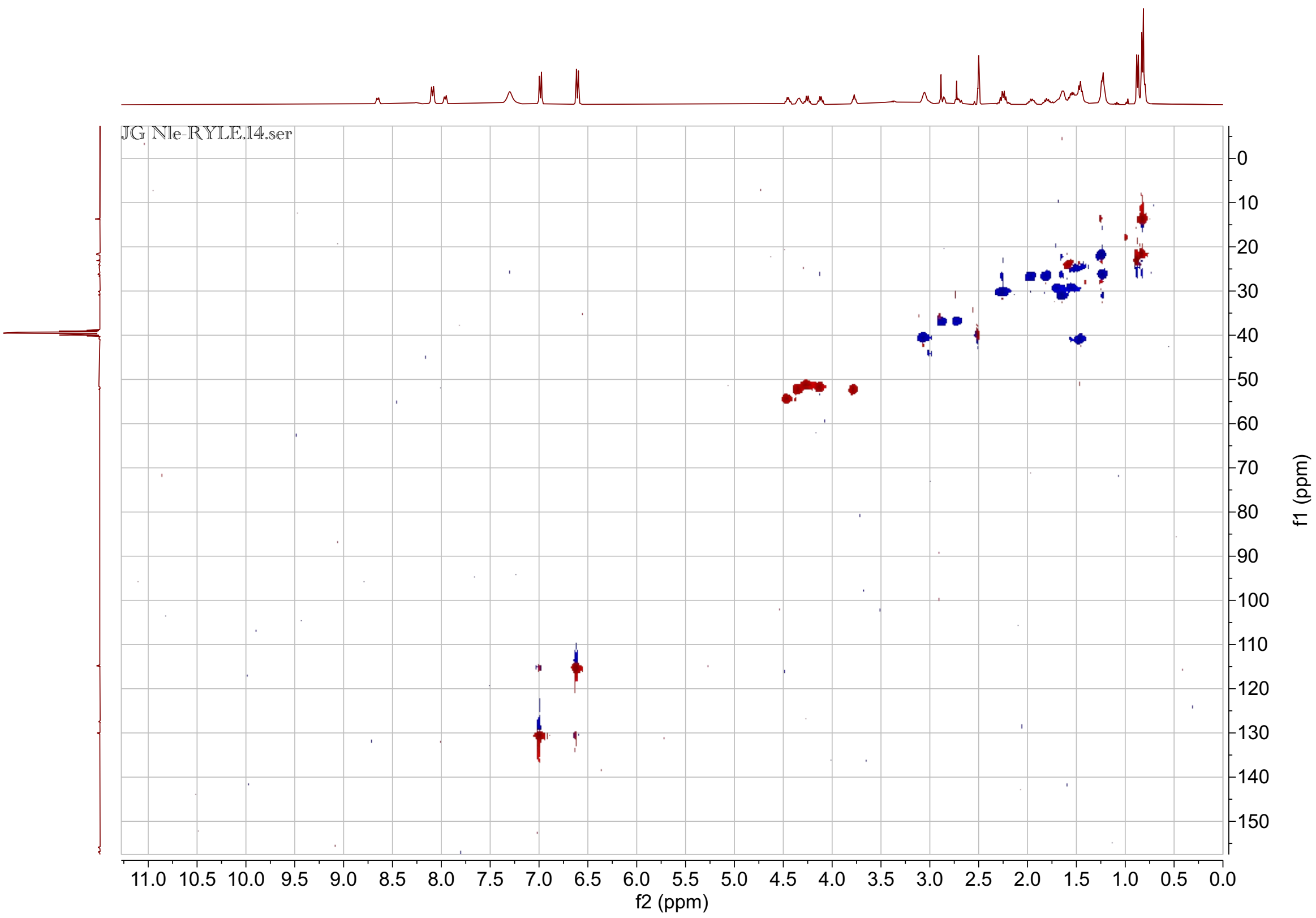

Figure S55. HSQC spectrum of Nle-RYL**E** in DMSO-d_6.

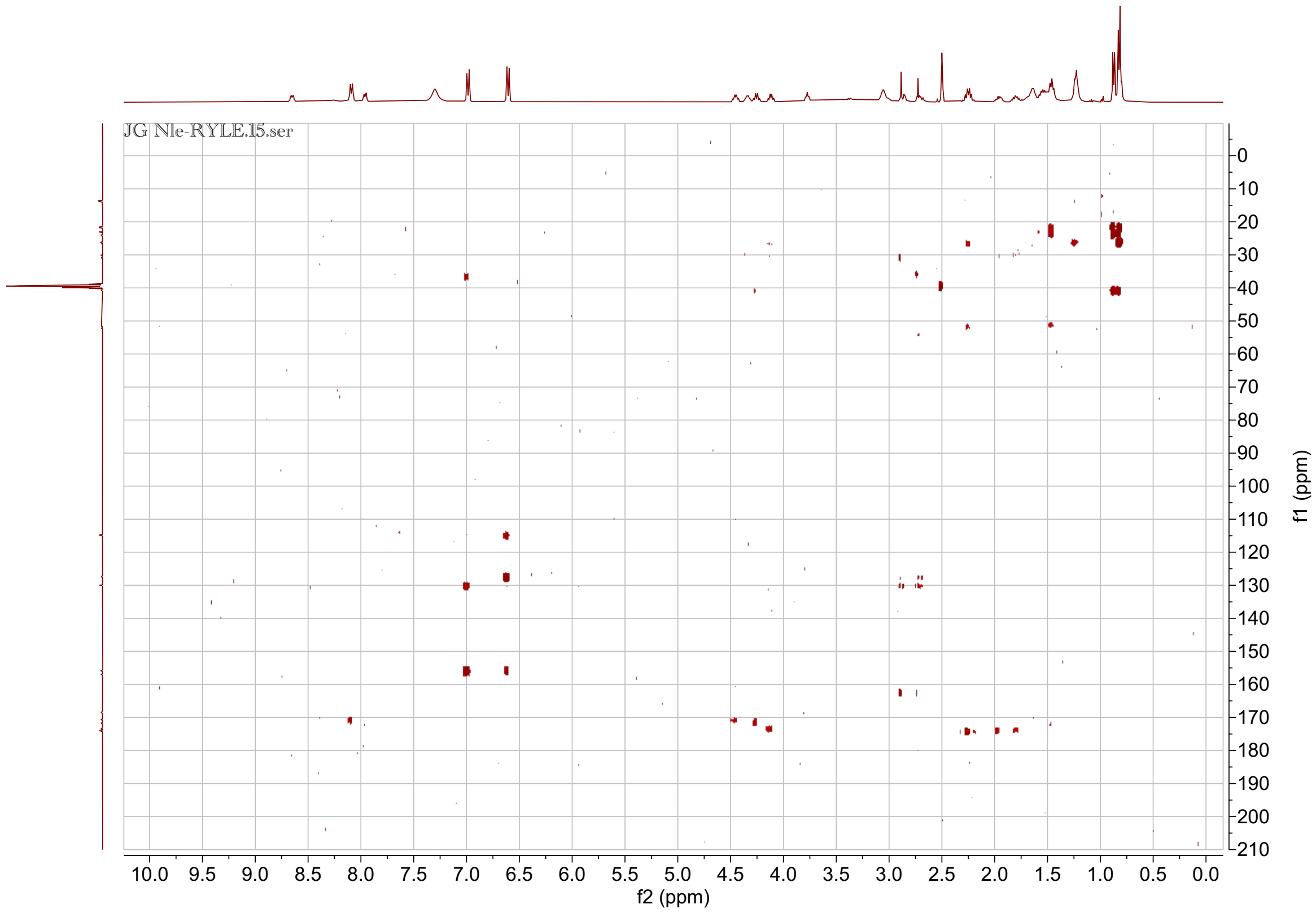

Figure S56. HMBC spectrum of Nle-RYL**E** in DMSO-d_6.

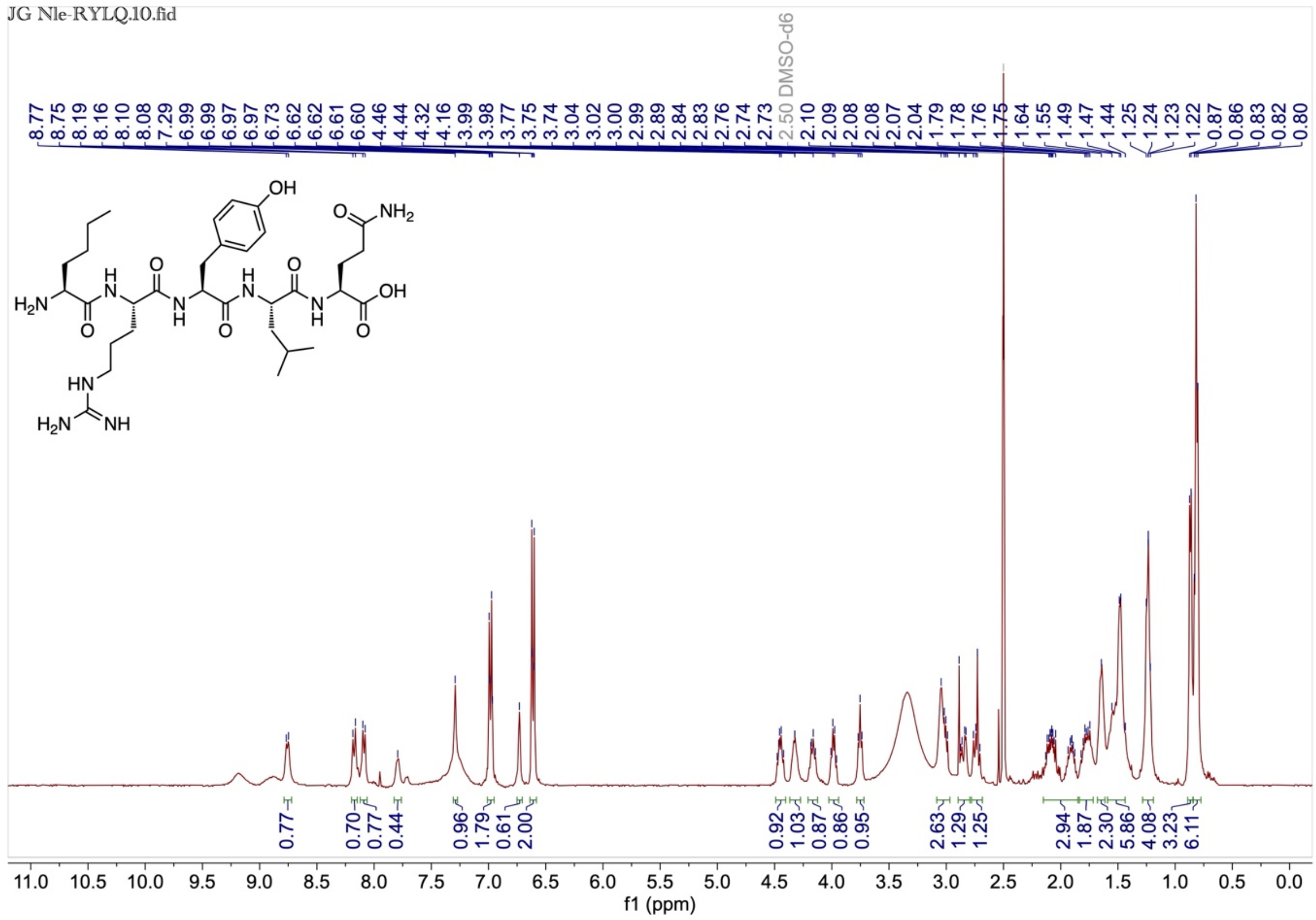

Figure S57. ^{1}H NMR of Nle-RYL**Q** in DMSO-d_6.

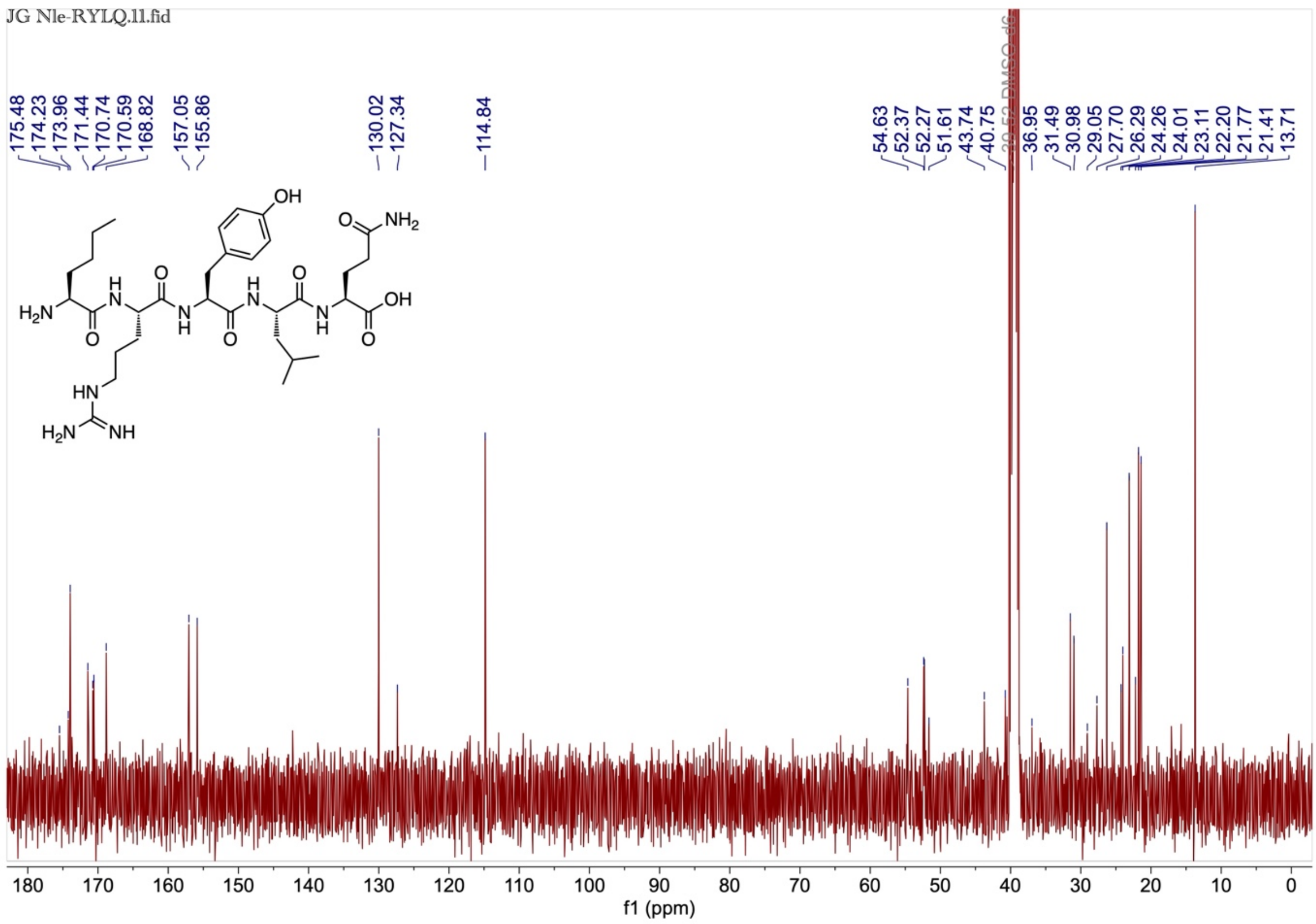

Figure S58. ^{13}C NMR of Nle-RYL**Q** in DMSO-d_6.

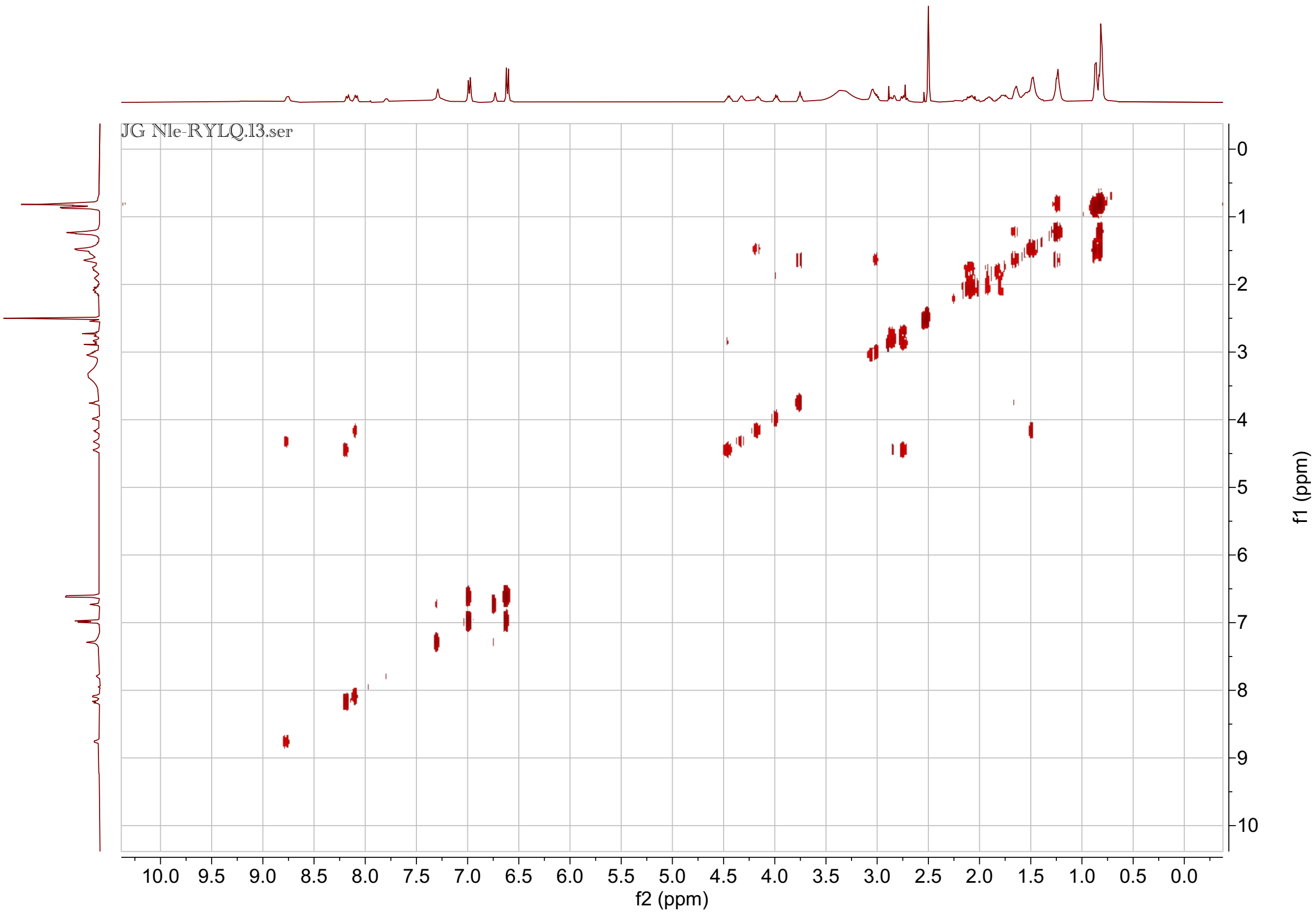

Figure S59. ^{1}H-^{1}H correlated spectroscopy spectrum of Nle-RYL**Q** in DMSO-d_6.

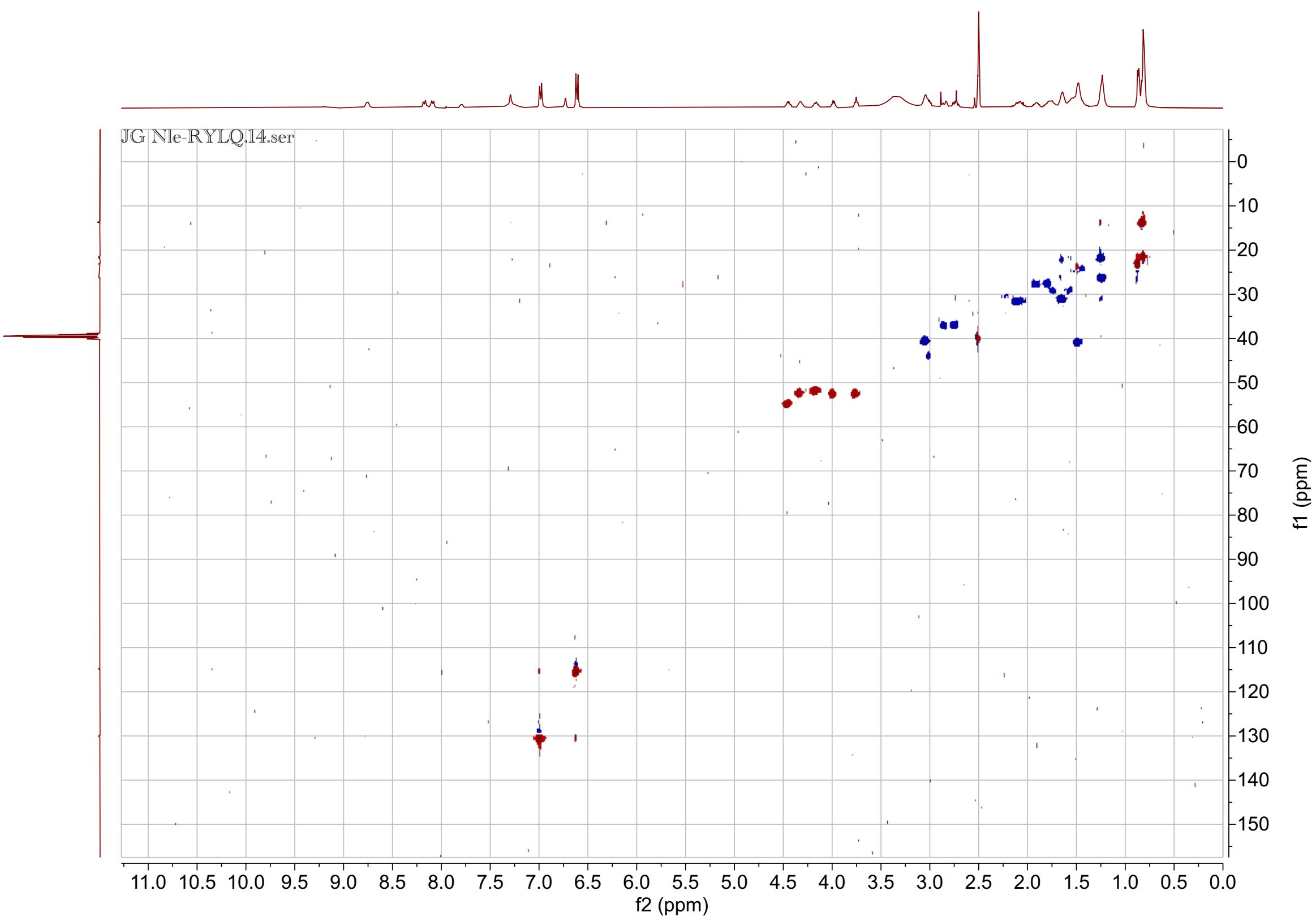

Figure S60. HSQC spectrum of Nle-RYL**Q** in DMSO-d_6.

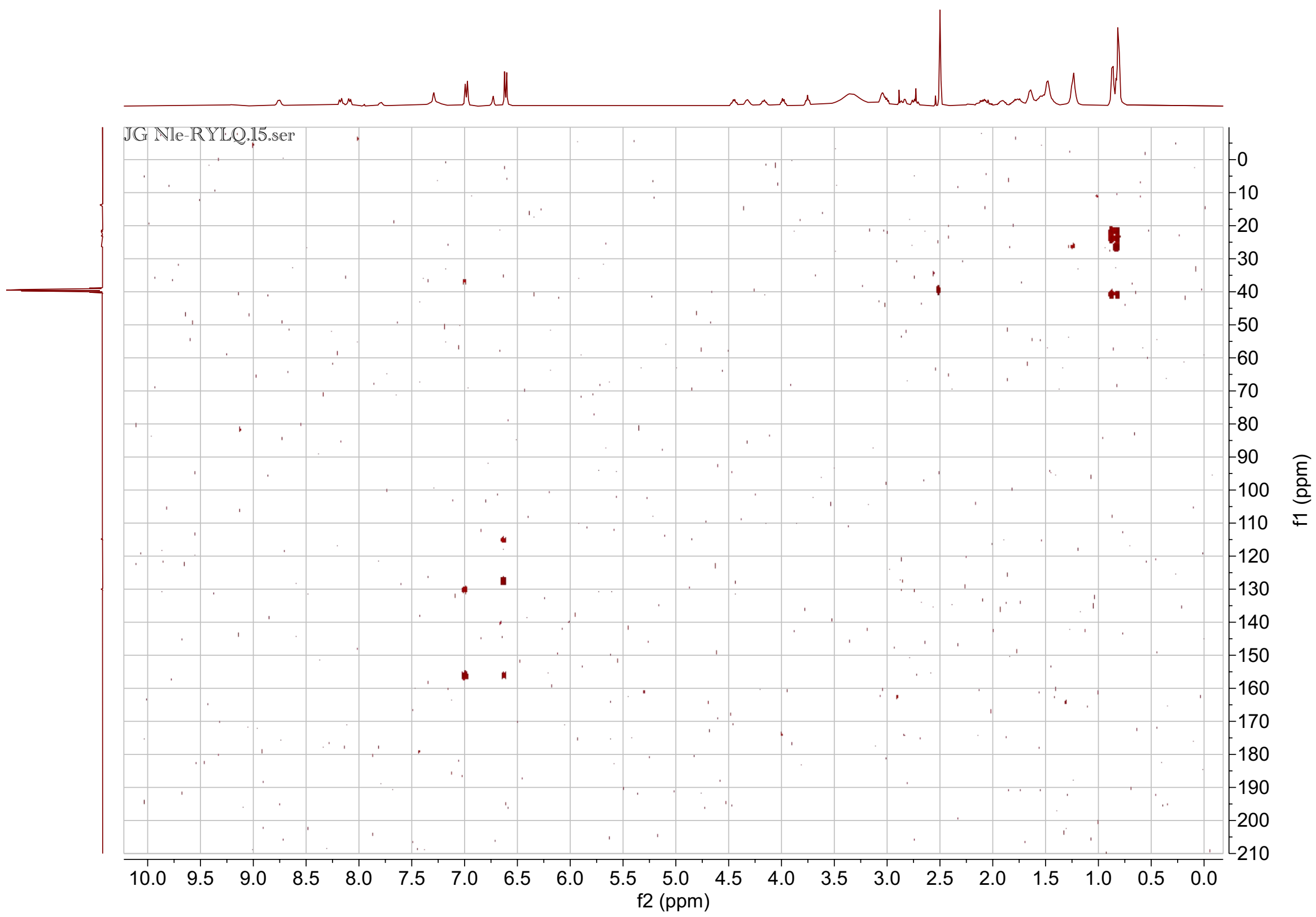

Figure S61. HMBC spectrum of Nle-RYL**Q** in DMSO-d6.

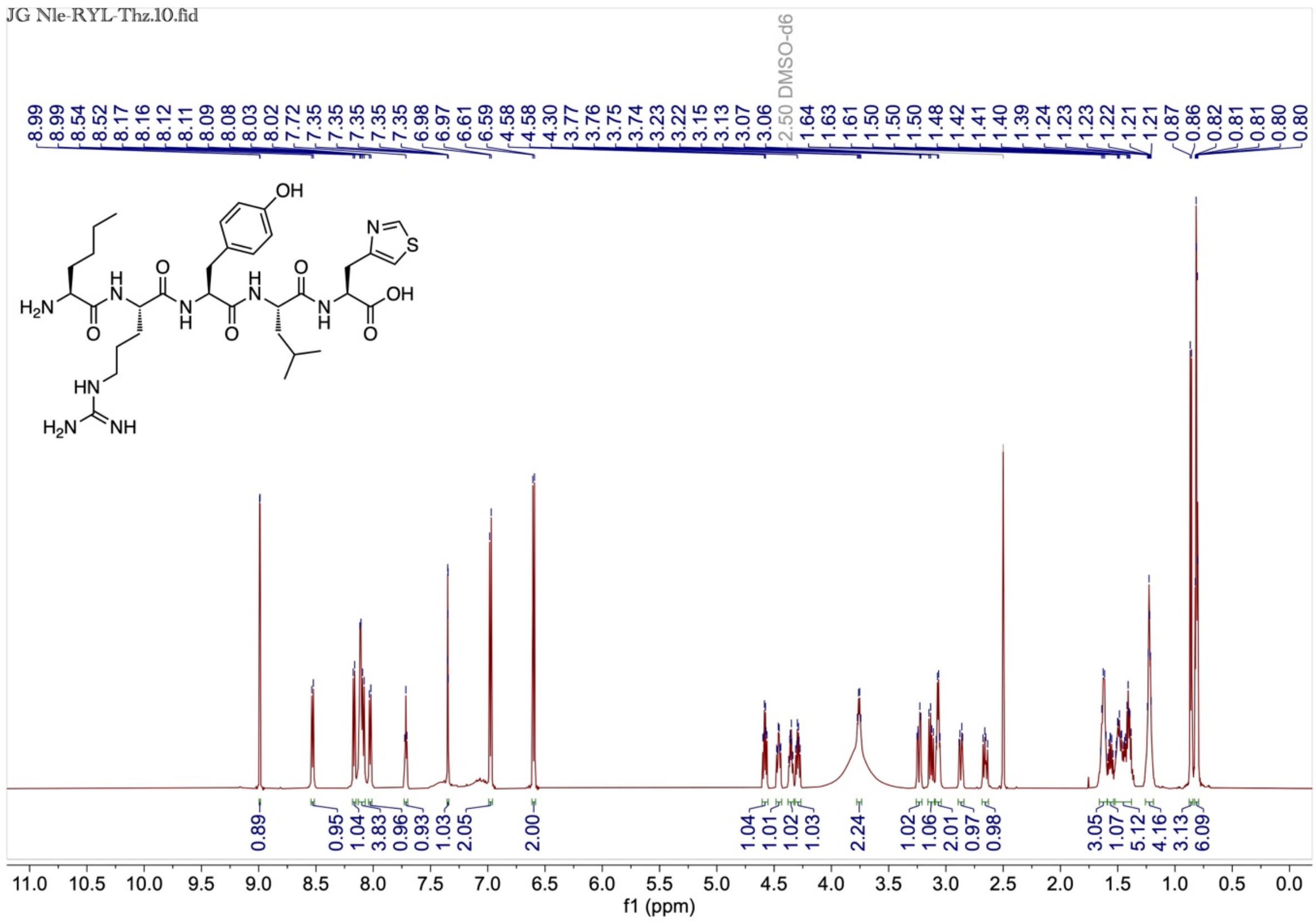

Figure S62. ^1H NMR of Nle-RYL-**Thz** in DMSO-d_6.

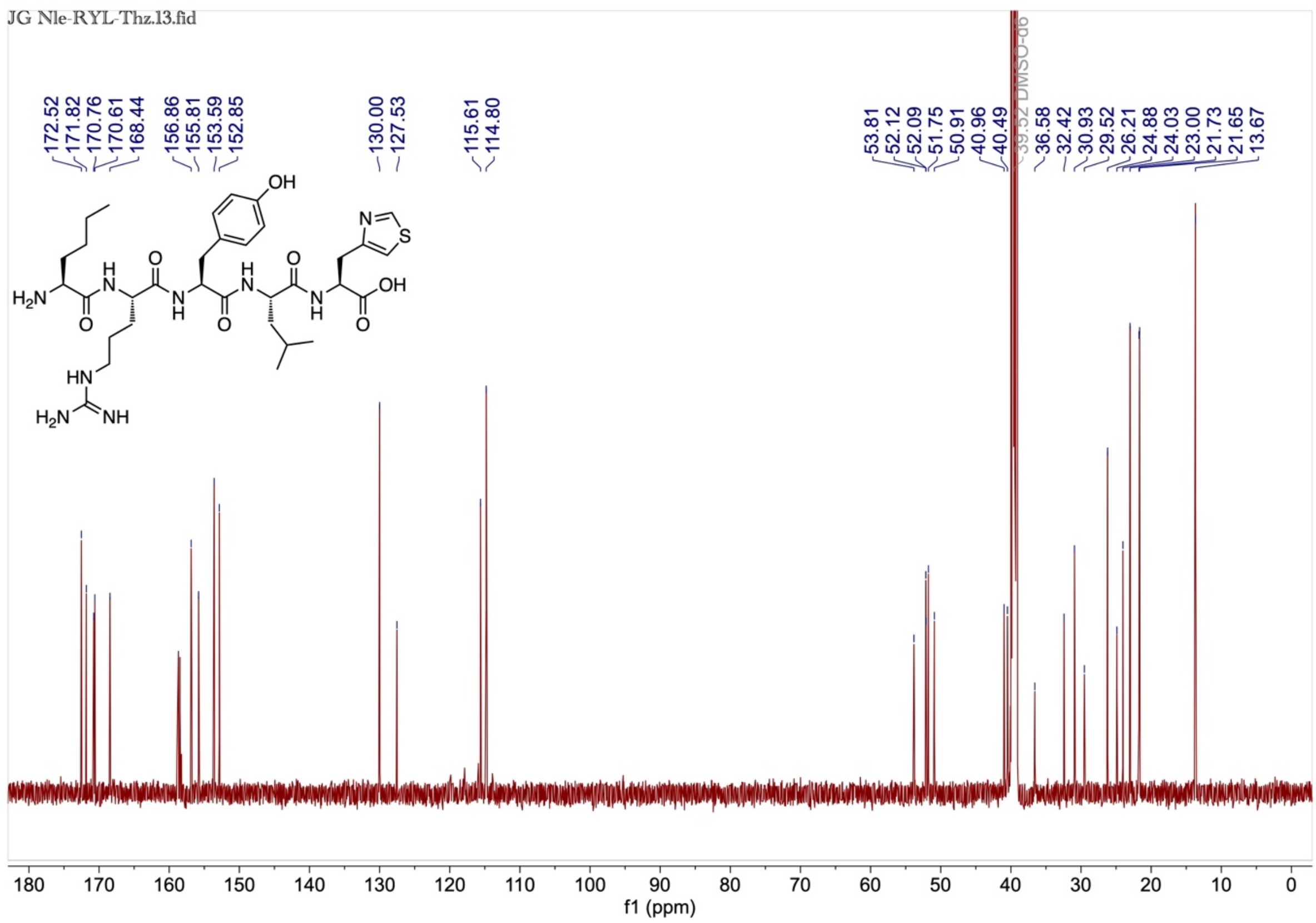

Figure S63. ^{13}C NMR of Nle-RYL-**Thz** in DMSO-d_6.

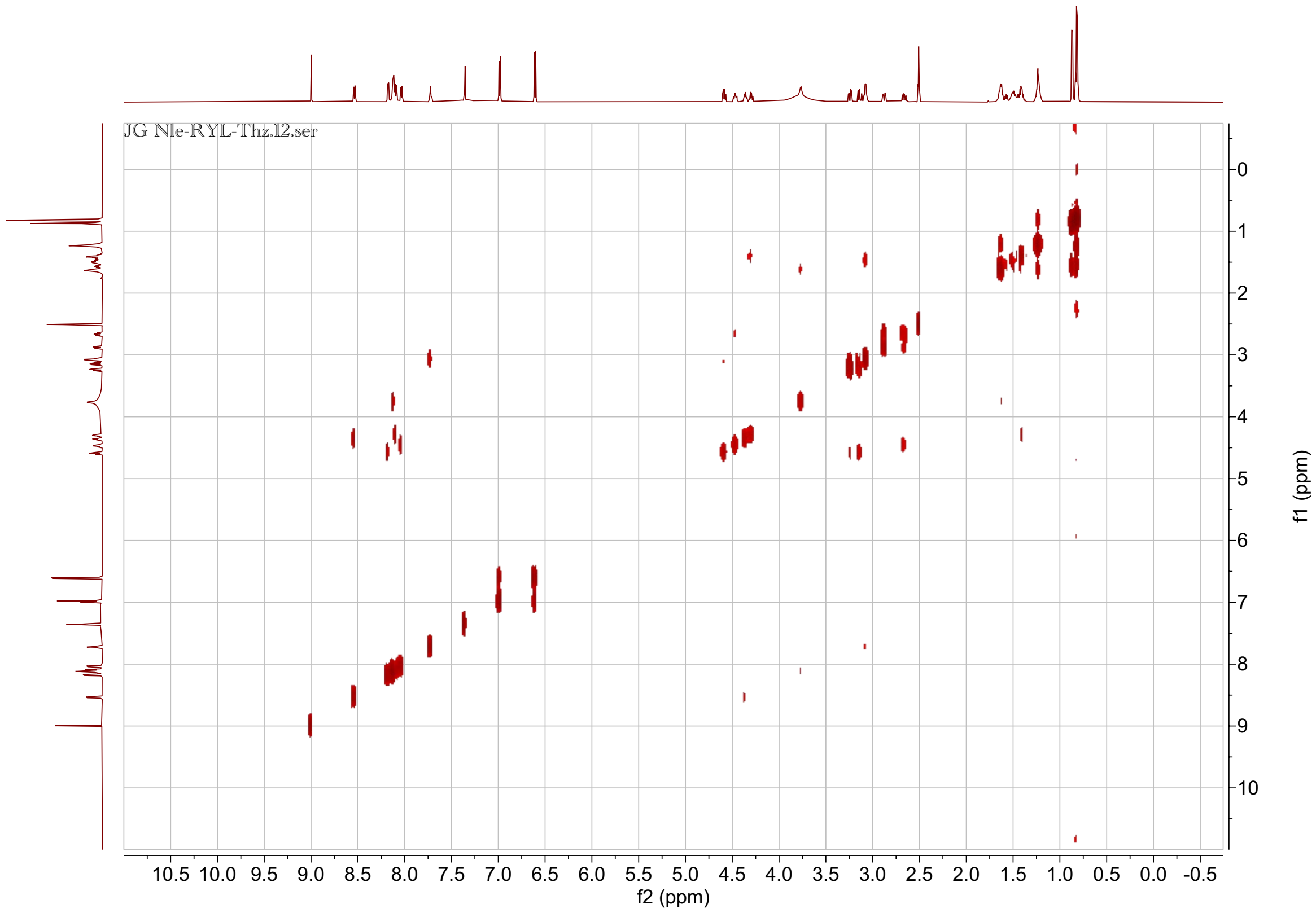

Figure S64. ^{1}H-^{1}H correlated spectroscopy spectrum of Nle-RYL-**Thz** in DMSO-d_6.

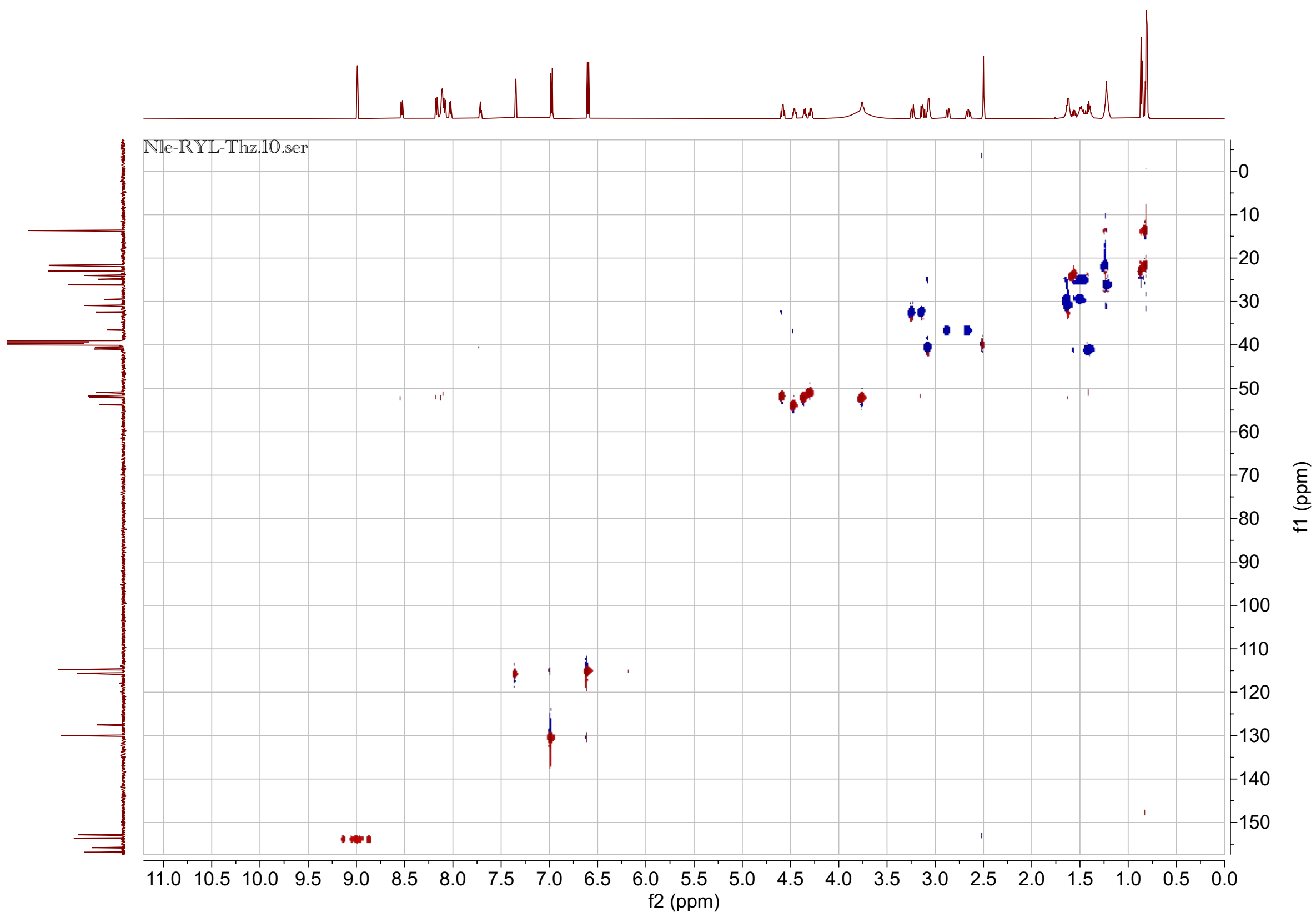

Figure S65. HSQC spectrum of Nle-RYL-**Thz** in DMSO-d_6.

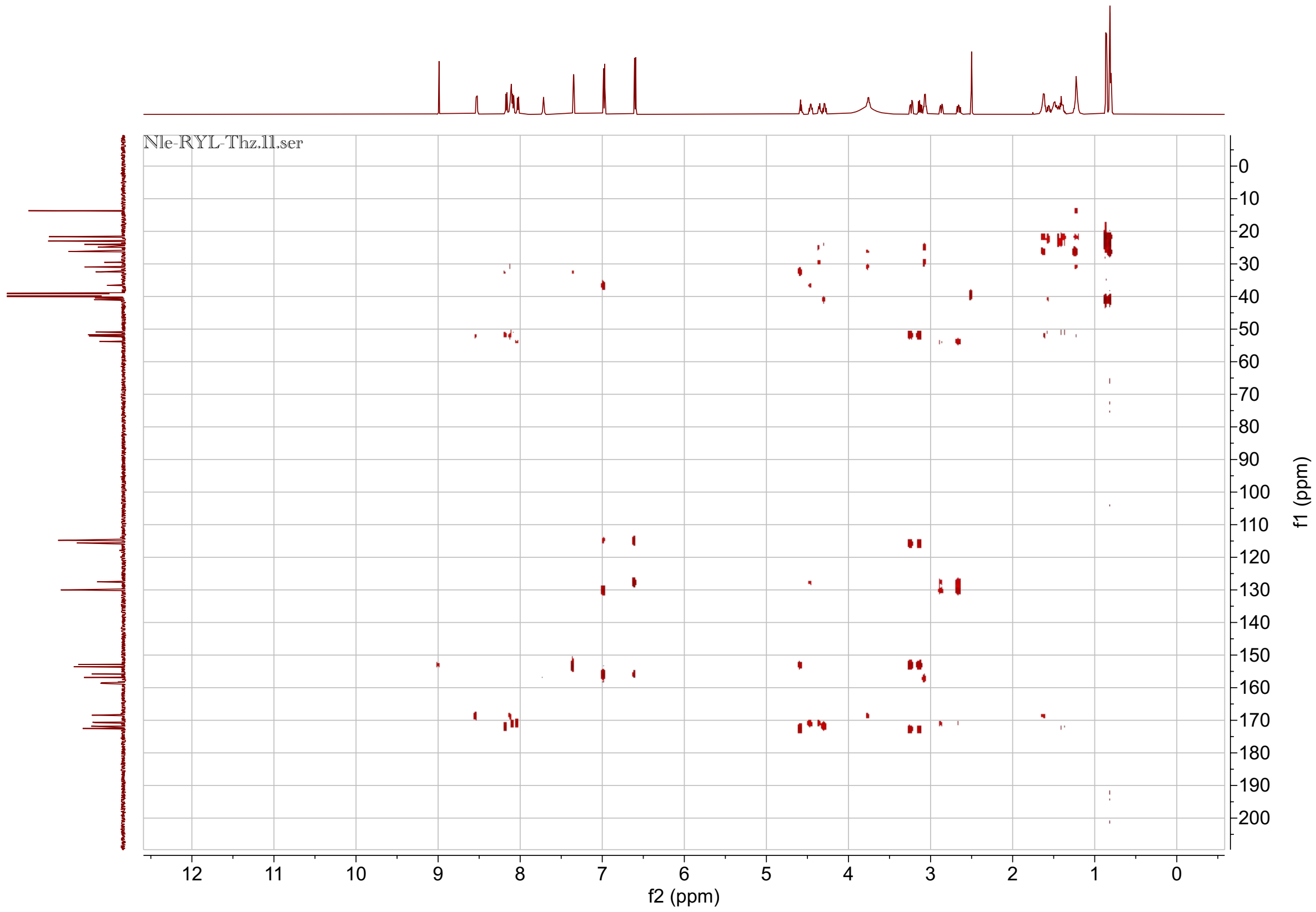

Figure S66. HMBC spectrum of Nle-RYL-**Thz** in DMSO-d$_6$.

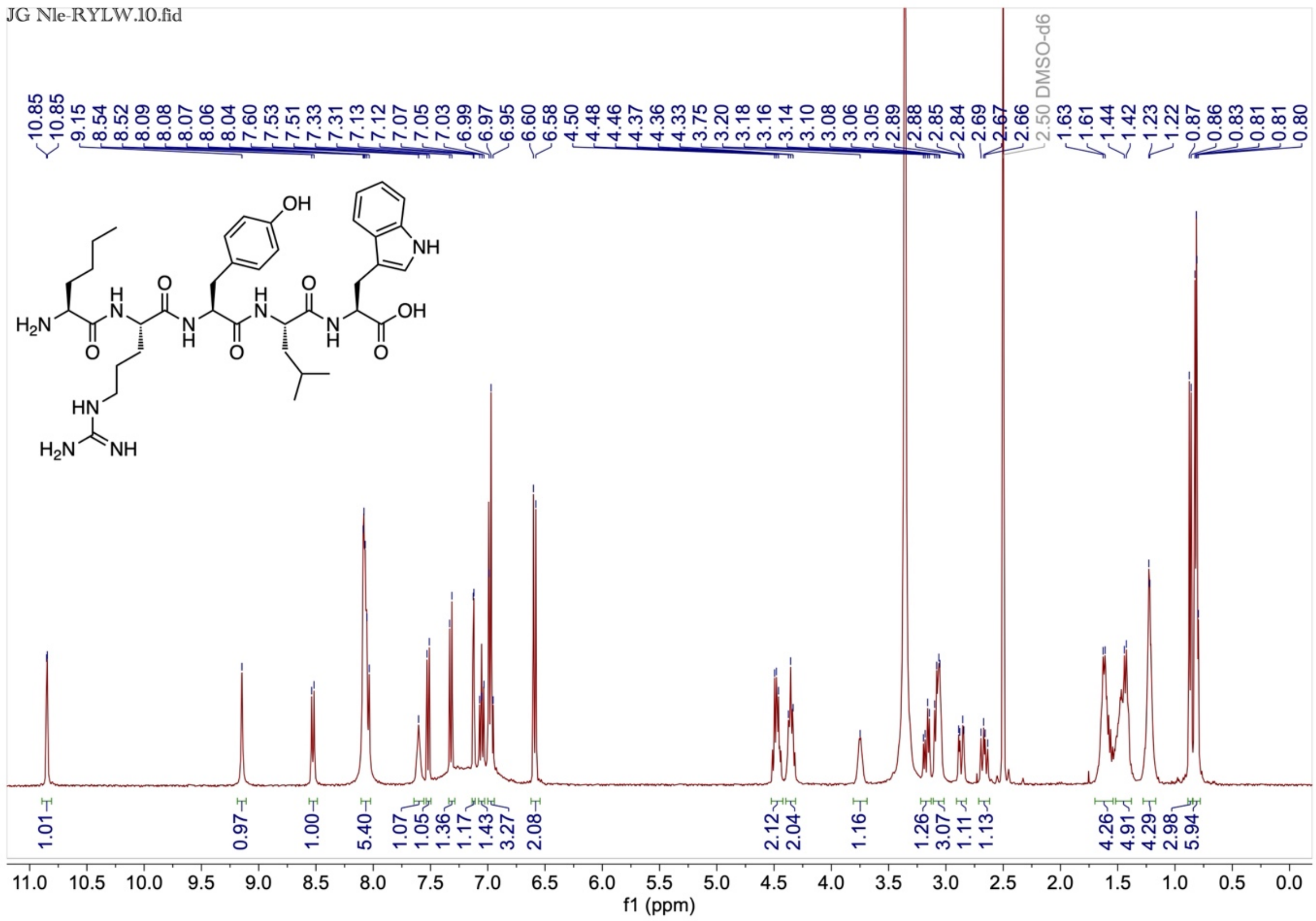

Figure S67. ^{1}H NMR of Nle-RYL**W** in DMSO-d$_6$.

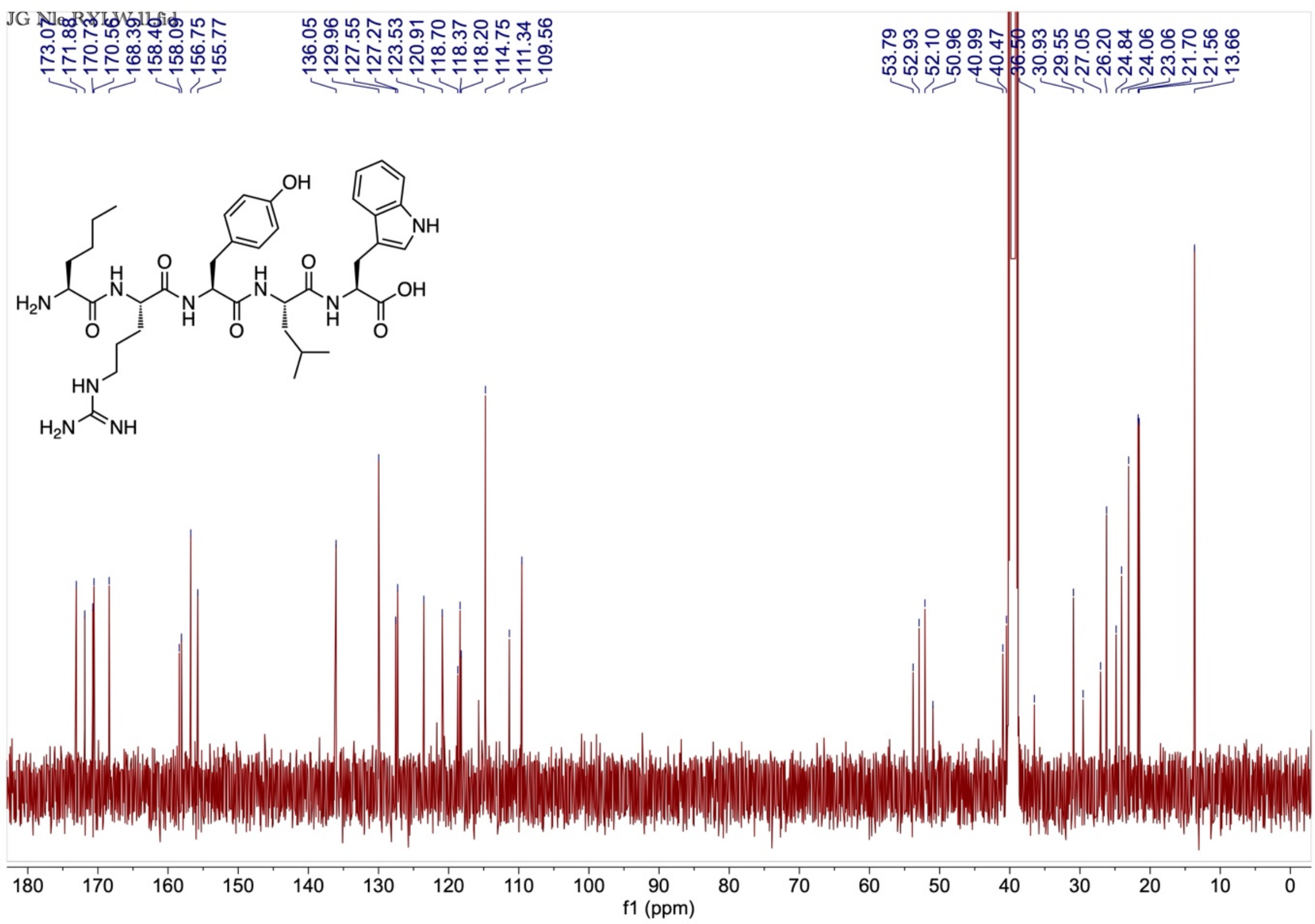

Figure S68. ^{13}C NMR of Nle-RYL**W** in DMSO-d$_6$.

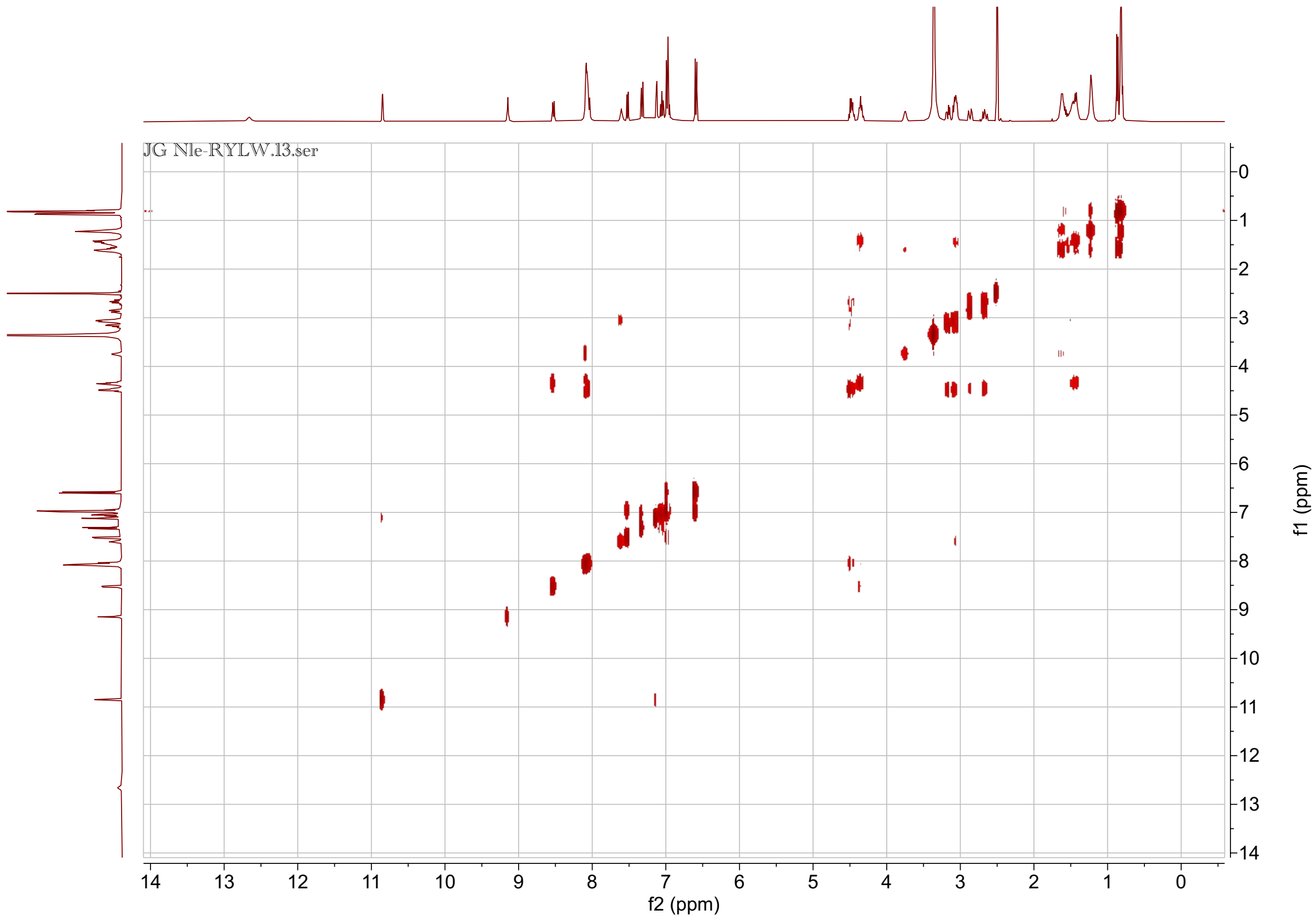

Figure S69. ^{1}H-^{1}H correlated spectroscopy spectrum of Nle-RYL**W** in DMSO-d_6.

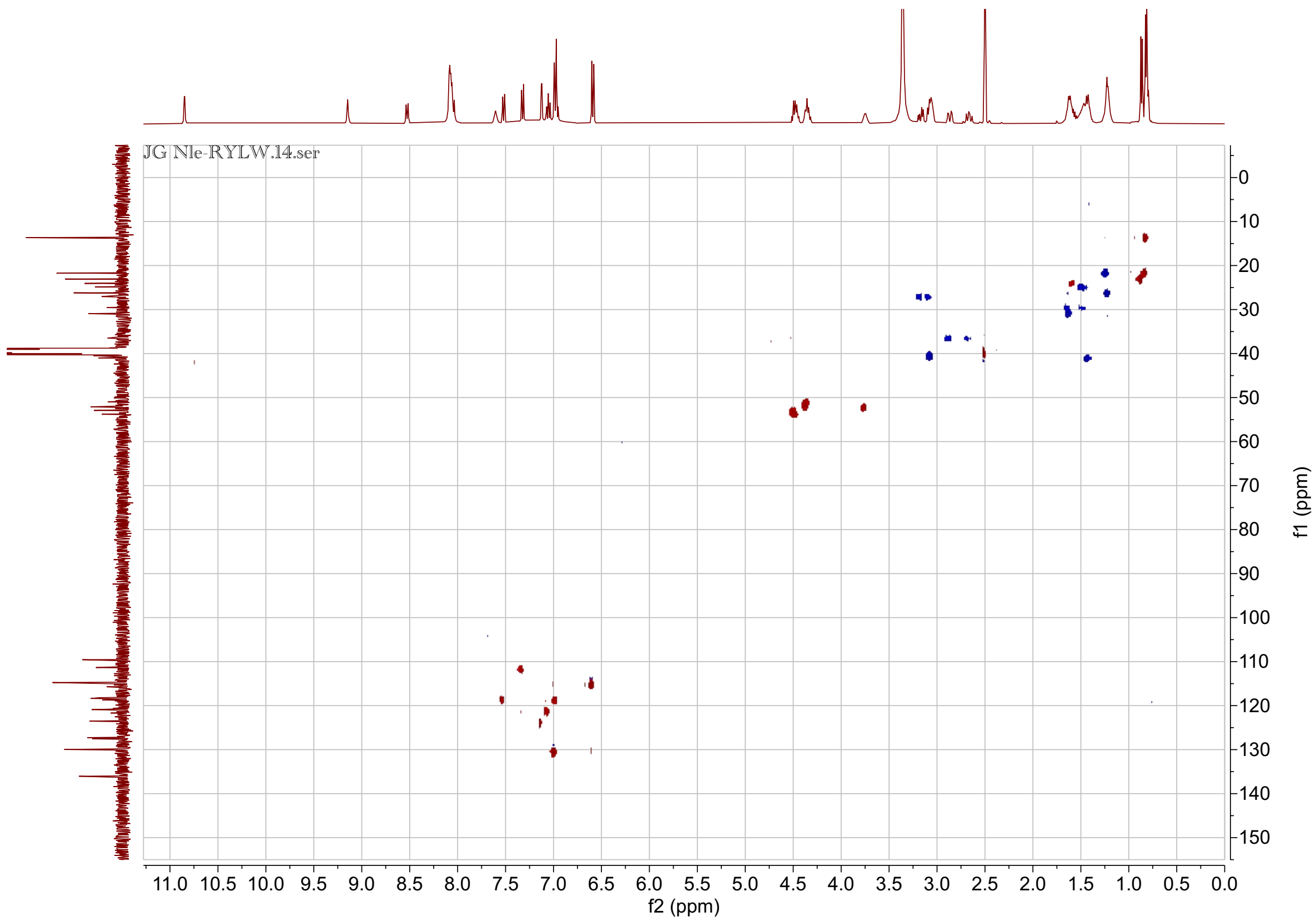

Figure S70. HSQC spectrum of Nle-RYL**W** in DMSO-d_6.

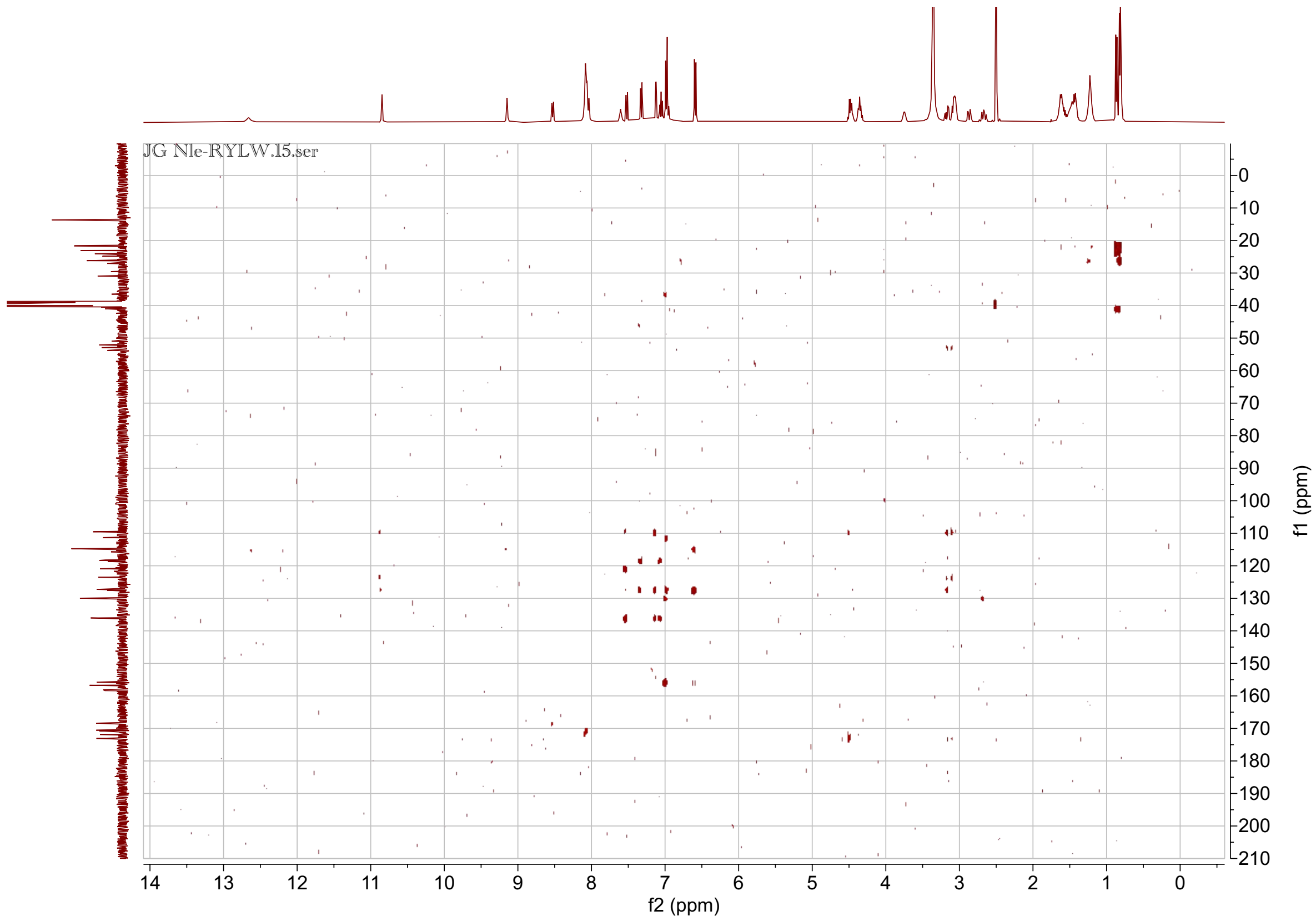

Figure S71. HMBC spectrum of Nle-RYL**W** in DMSO-d_6.

SDS-PAGE analysis of RufO and NsRufO

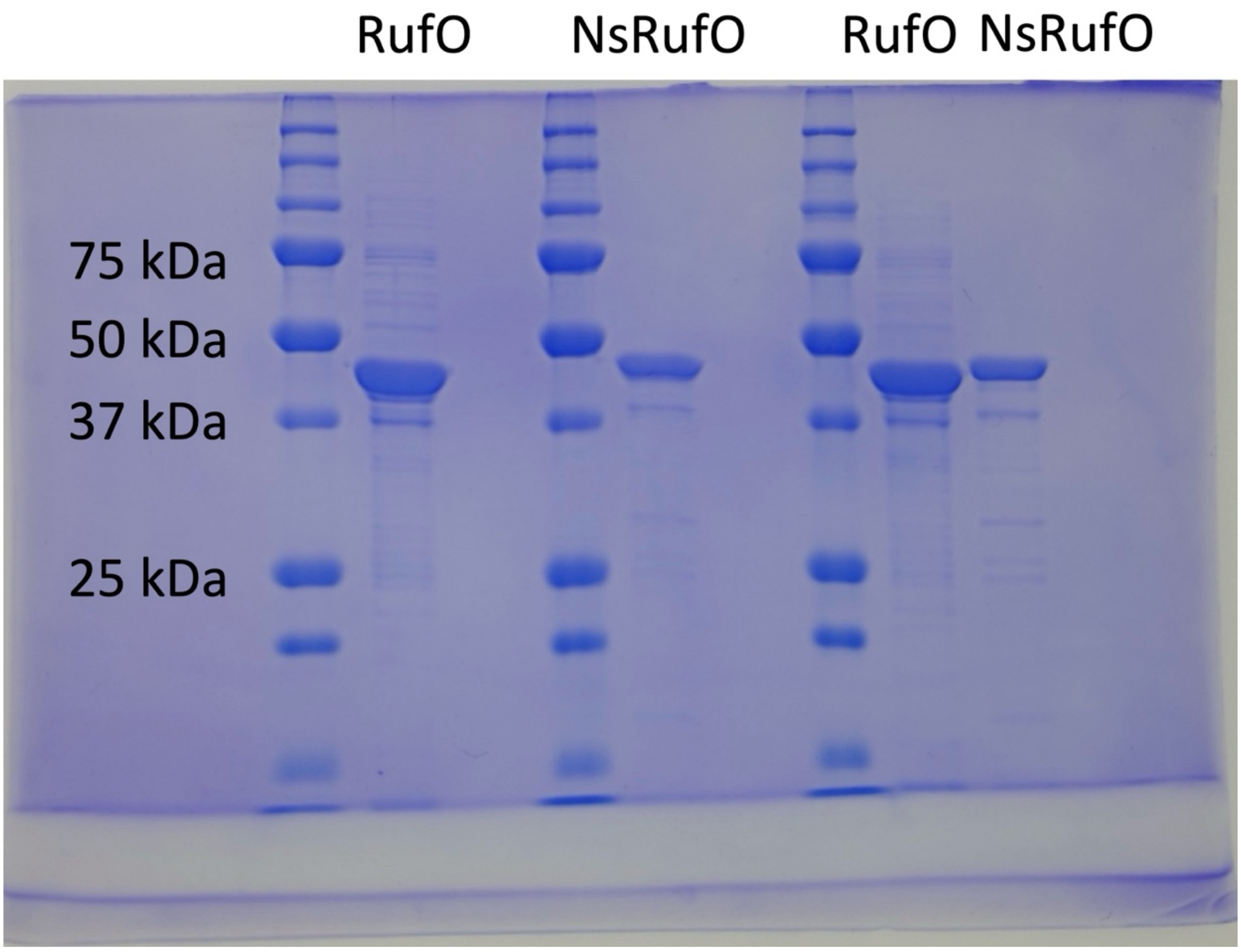

Figure S72. SDS-PAGE analysis of RufO and NsRufO.

Analysis of *in vitro* enzymatic assays with RufO and NsRufO

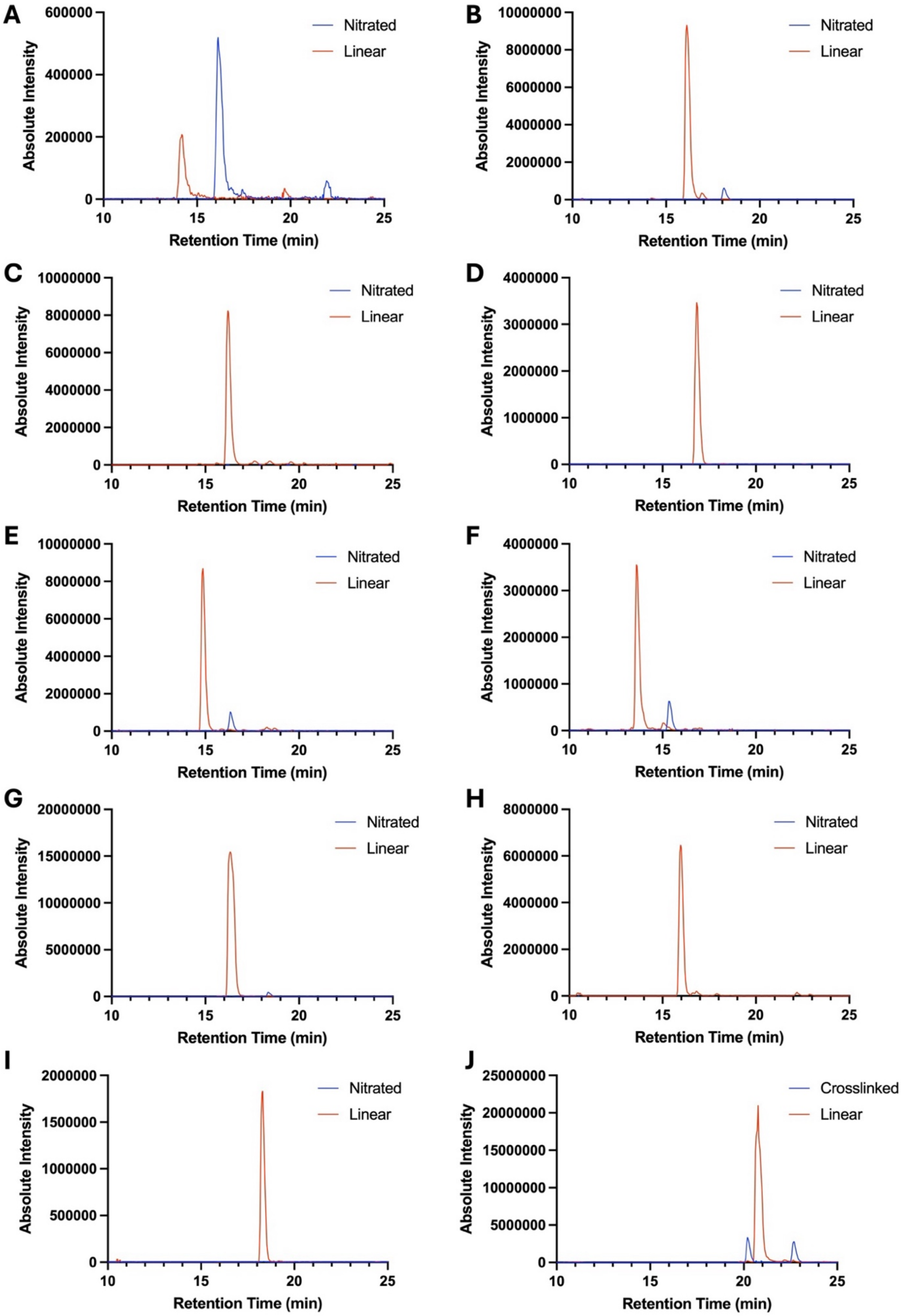

Figure S73. *In vitro* enzymatic nitration and crosslinking results for RufO with a) Nle-RYLH, b) Nle-**A**YLH, c) Nle-R**F**LH, d) Nle-R-**(OMe)Tyr**-LH, e) Nle-R-**mTyr**-LH, f) Nle-RY**Y**H, g) Nle-RYL**E**, h) Nle-RYL**Q**, i) Nle-RYL-**Thz** and j) Nle-RYL**W**. Data shown as absolute intensity of TIC for m/z corresponding to the appropriate peptide.

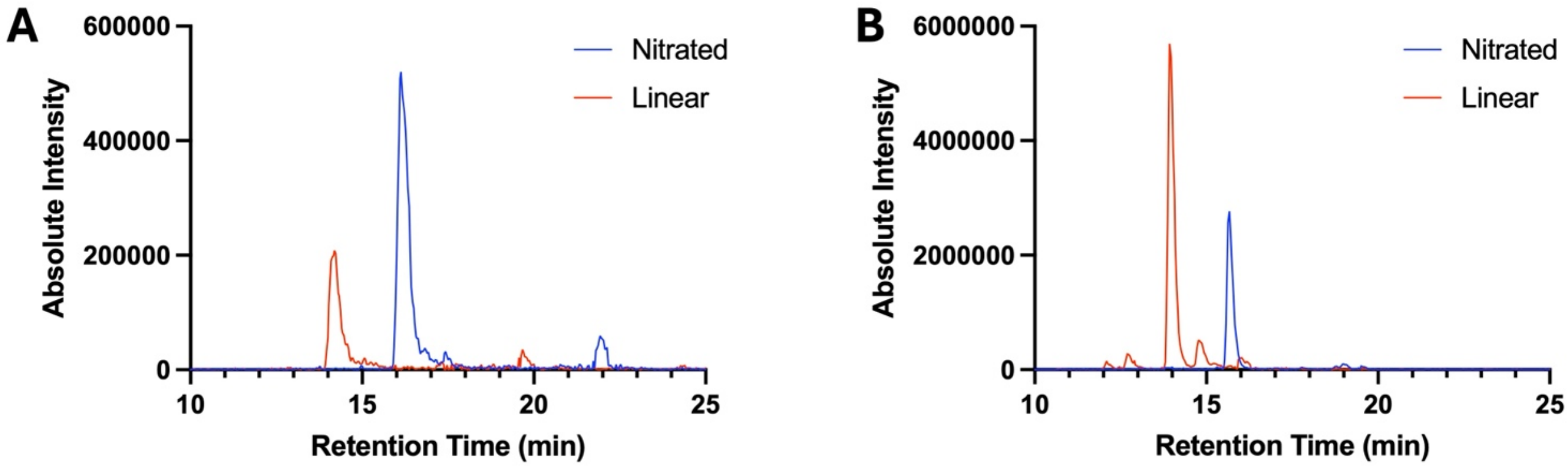

Figure S74. *In vitro* nitration results for Nle-RYLH with a) RufO and b) NsRufO. Data shown as absolute intensity of TIC for m/z corresponding to the appropriate peptide.

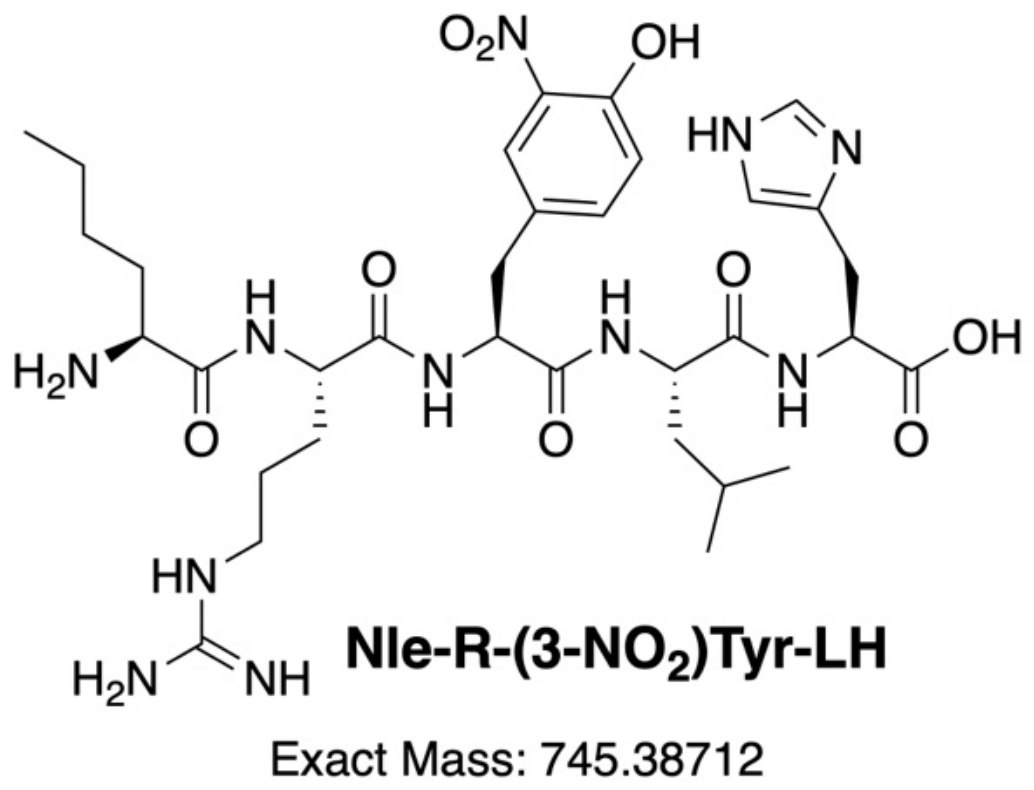

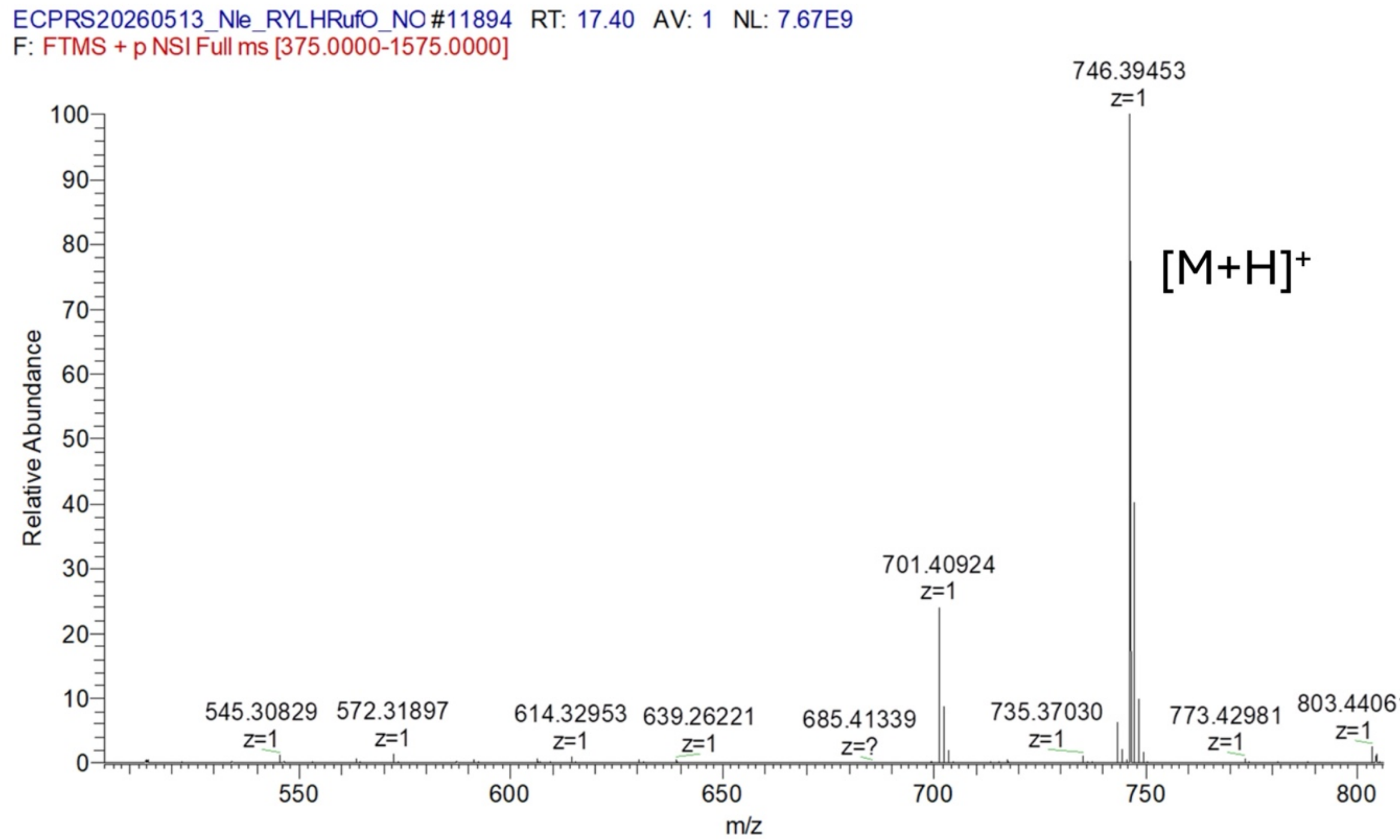

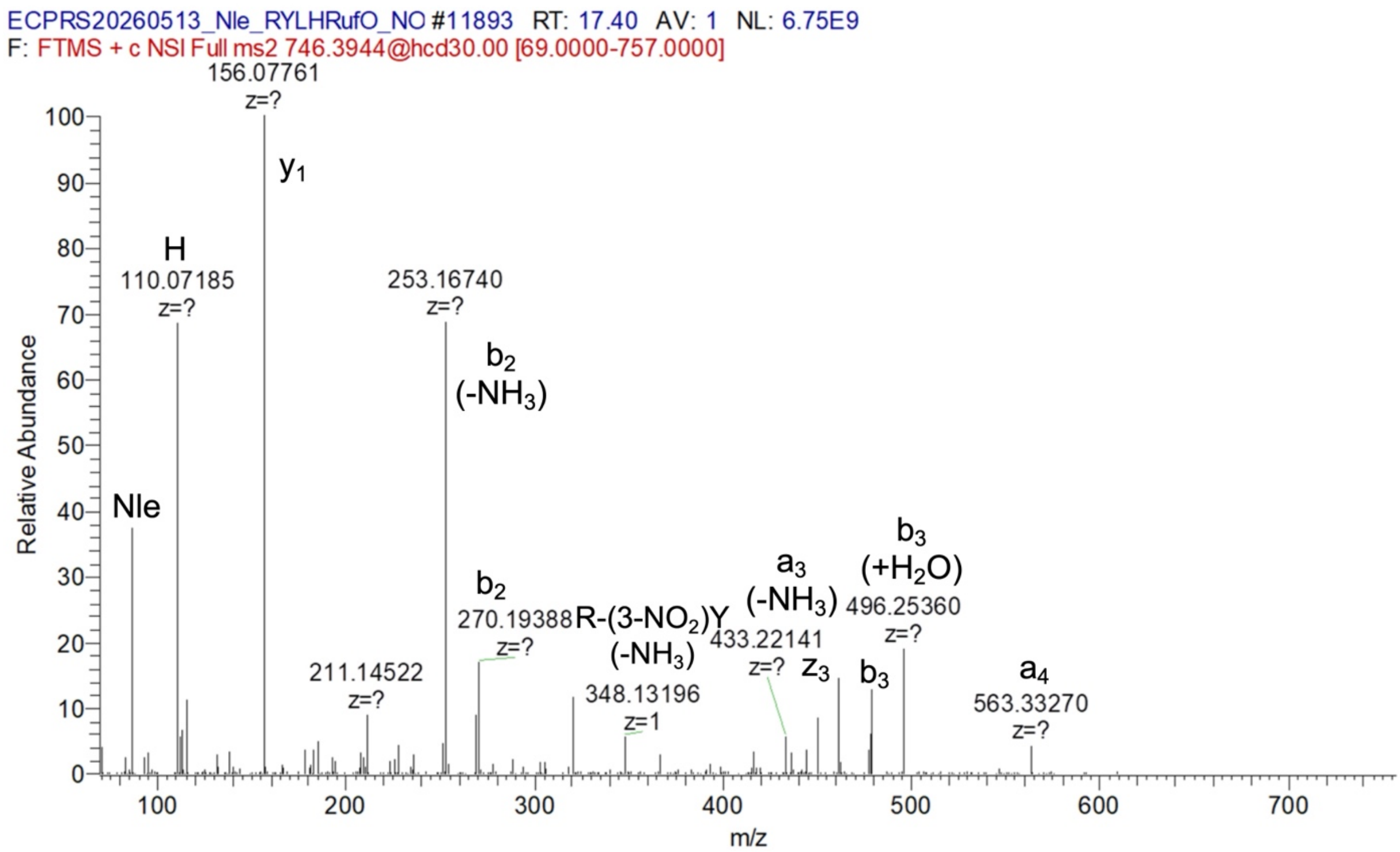

Figure S75. HRMS and MS[2] spectra for the nitrated product of the turnover of Nle-RYLH with RufO.

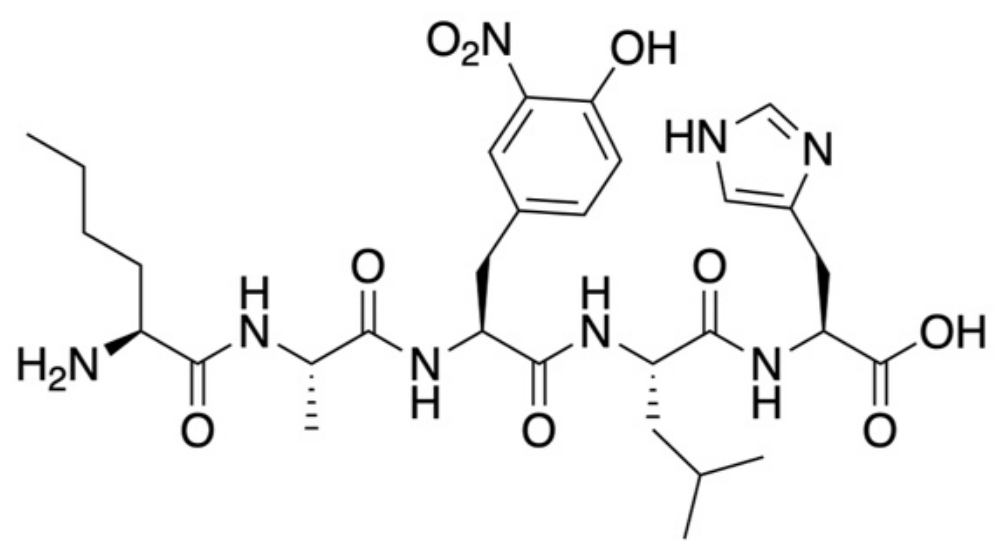

Nle-A-(3-NO$_2$)Tyr-LH

Exact Mass: 660.32313

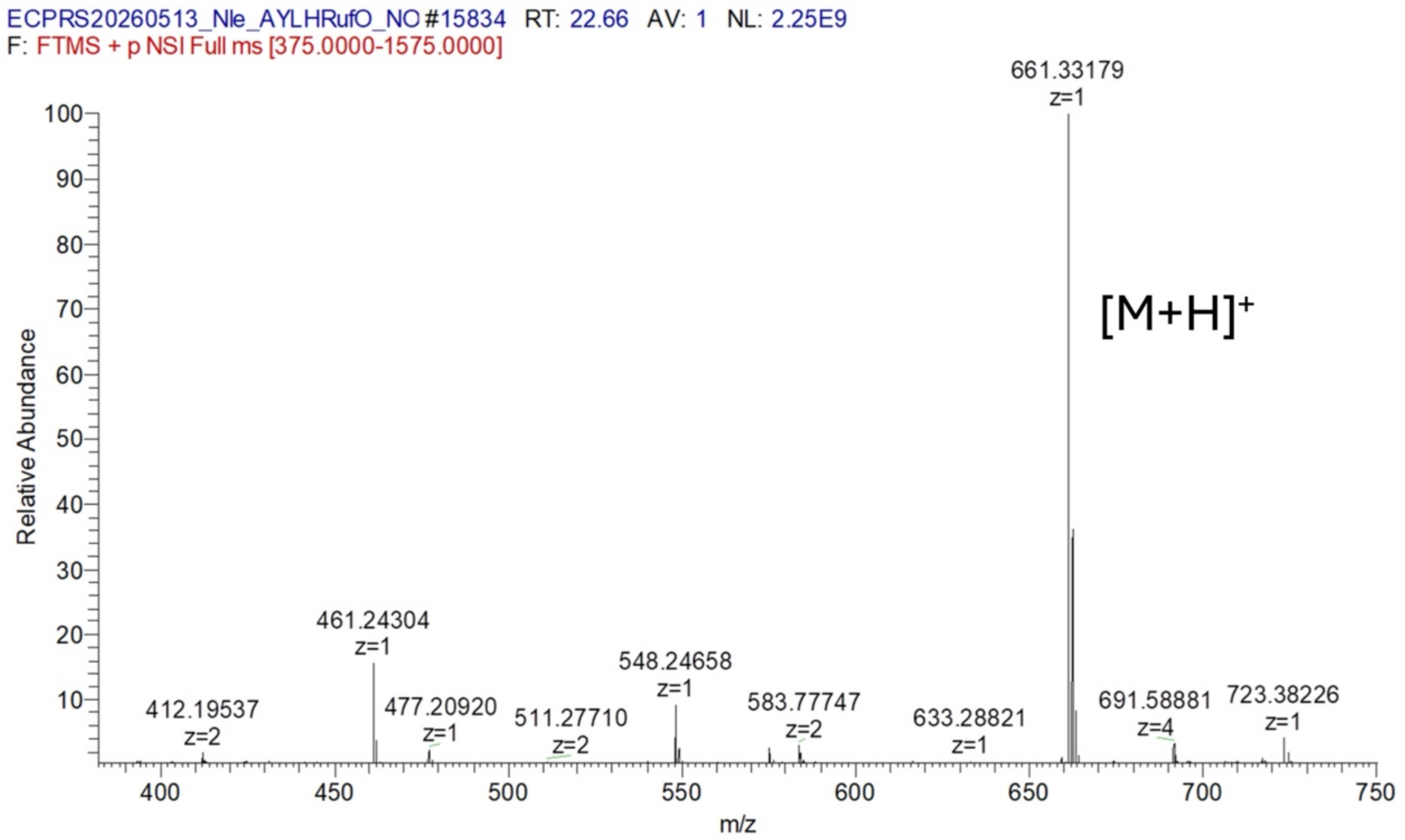

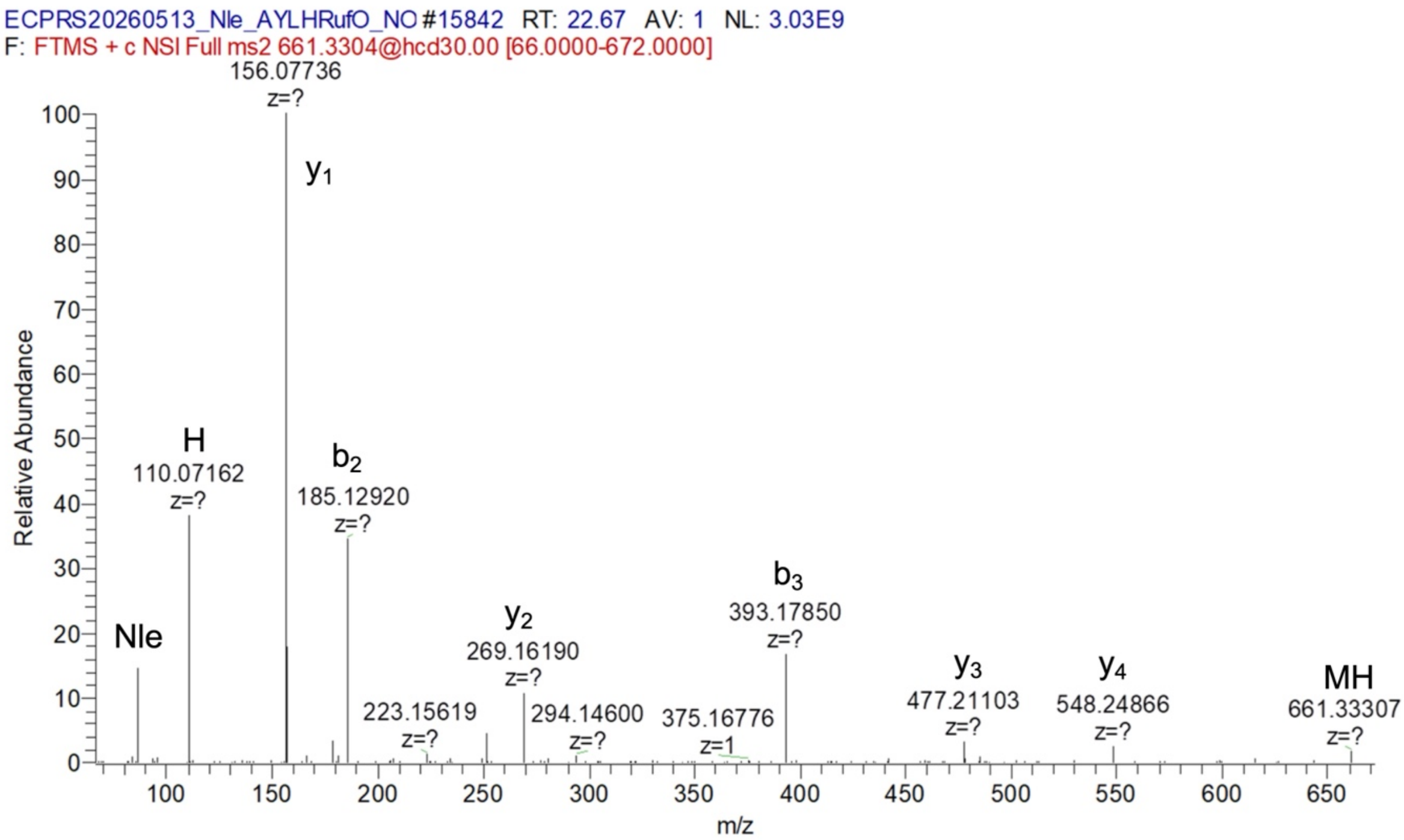

Figure S76. HRMS and MS^2 spectra for the nitrated product of the turnover of Nle-**A**YLH with RufO.

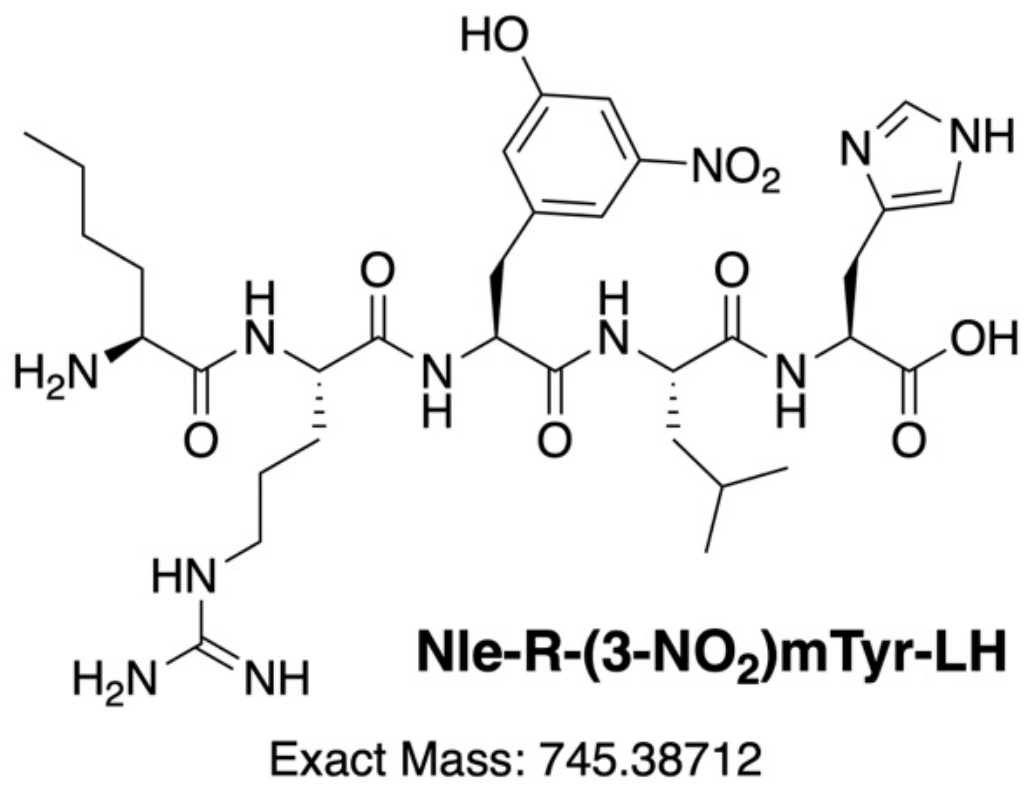

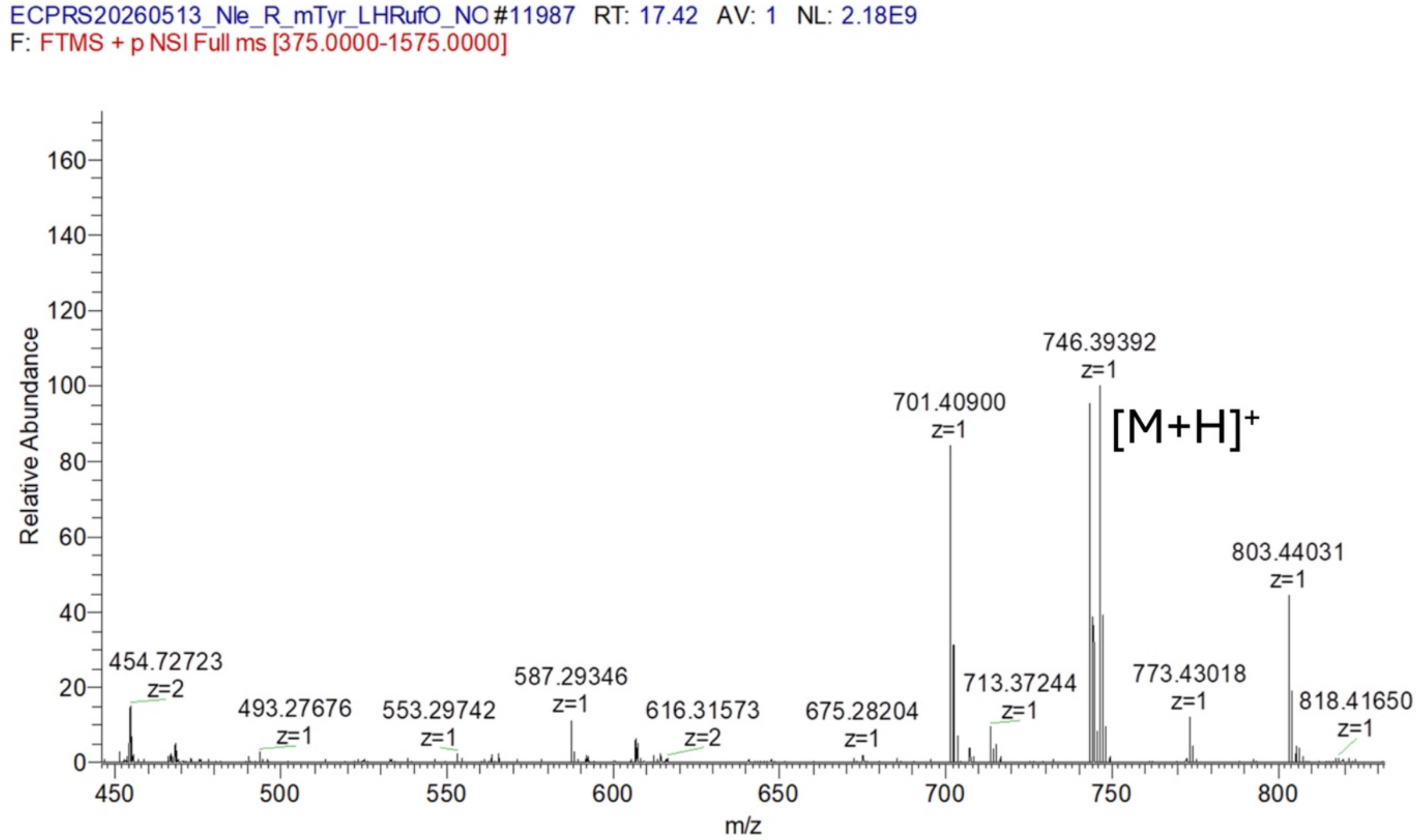

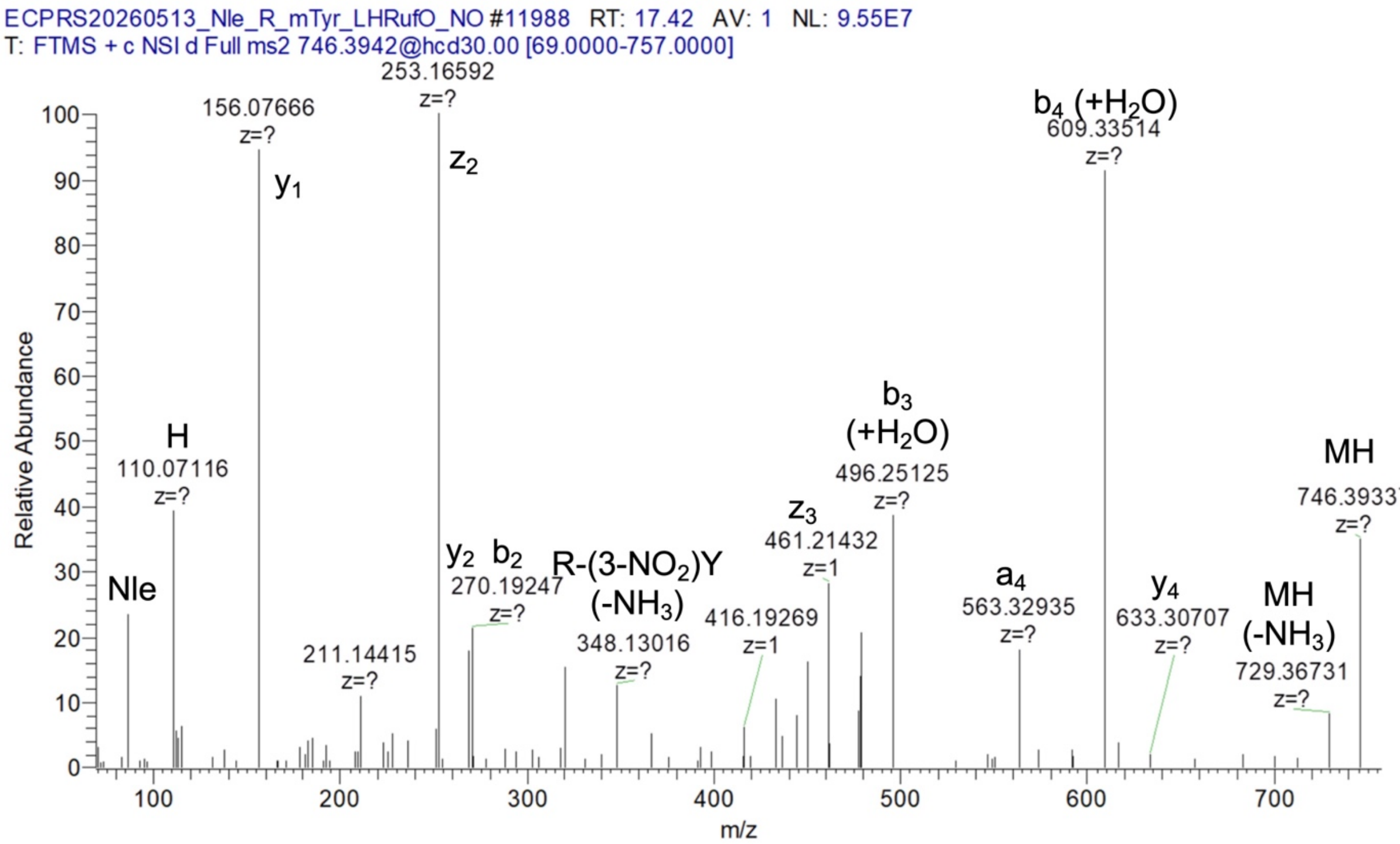

Figure S77. HRMS and MS[2] spectra for the nitrated product of the turnover of Nle-R-**mTyr**-LH with RufO.

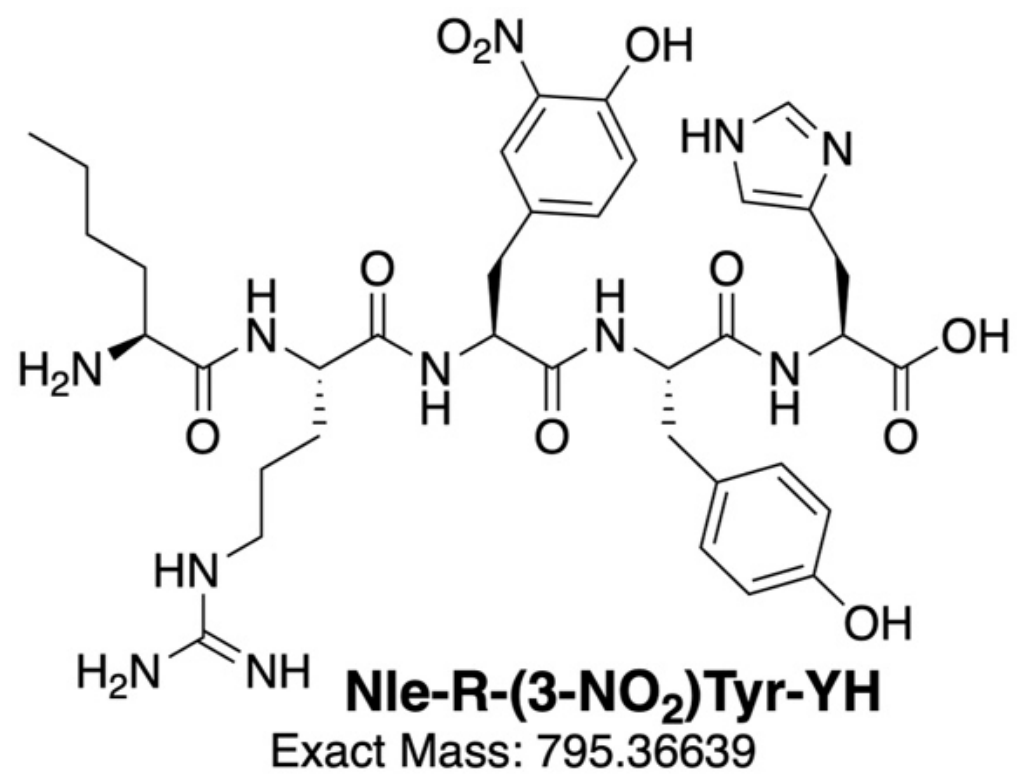

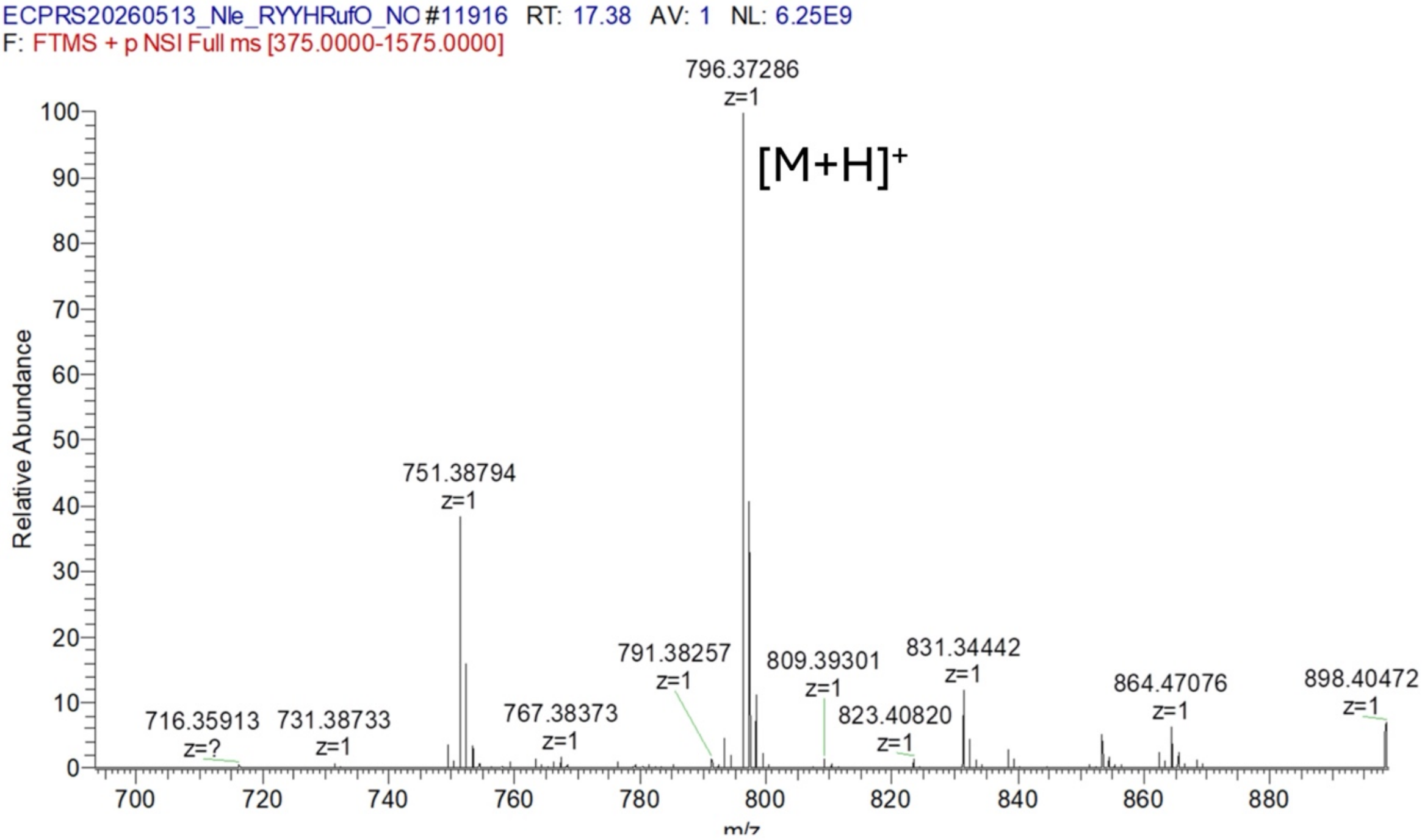

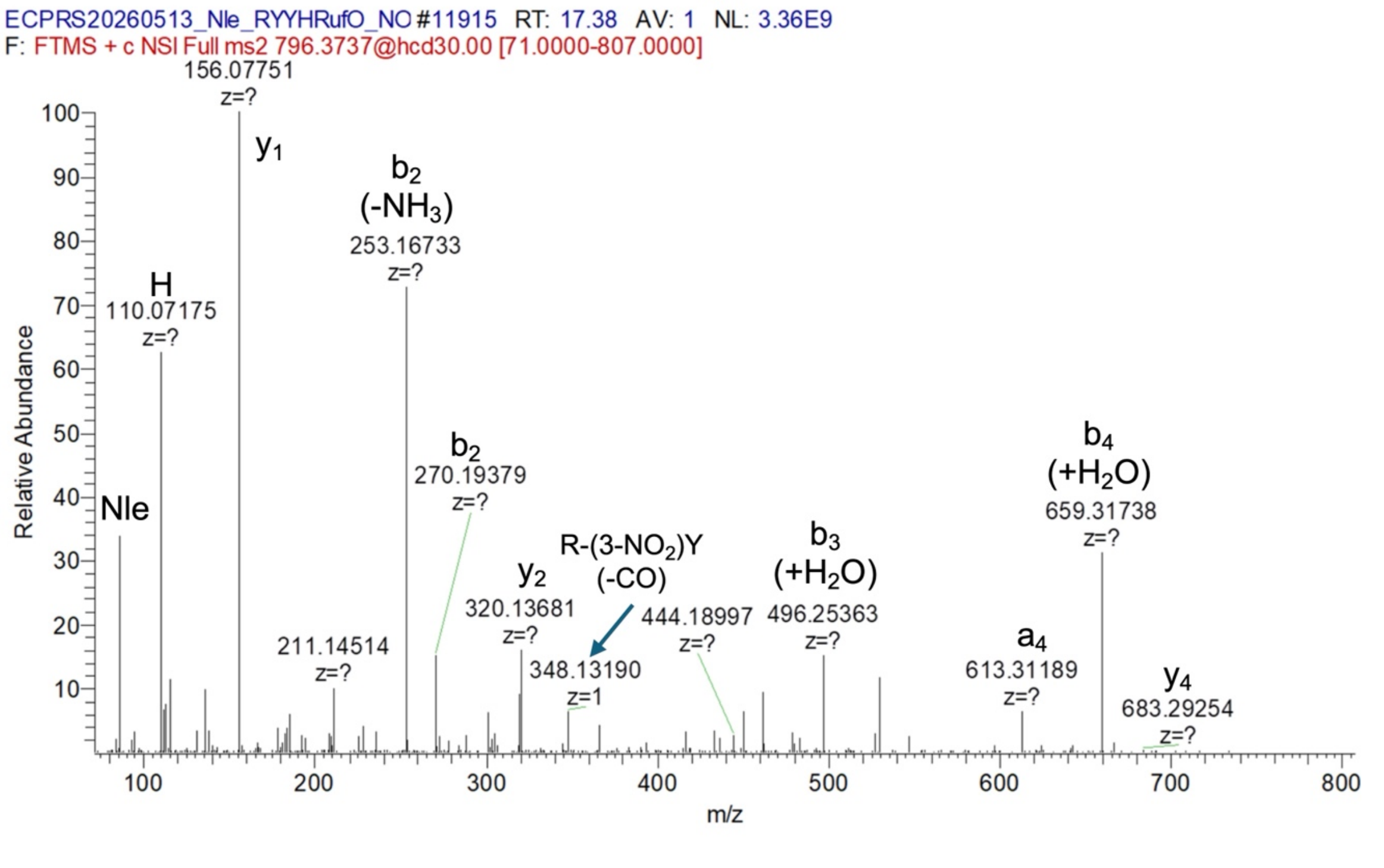

Figure S78. HRMS and MS[2] spectra for the nitrated product of the turnover of Nle-RY**Y**H with RufO.

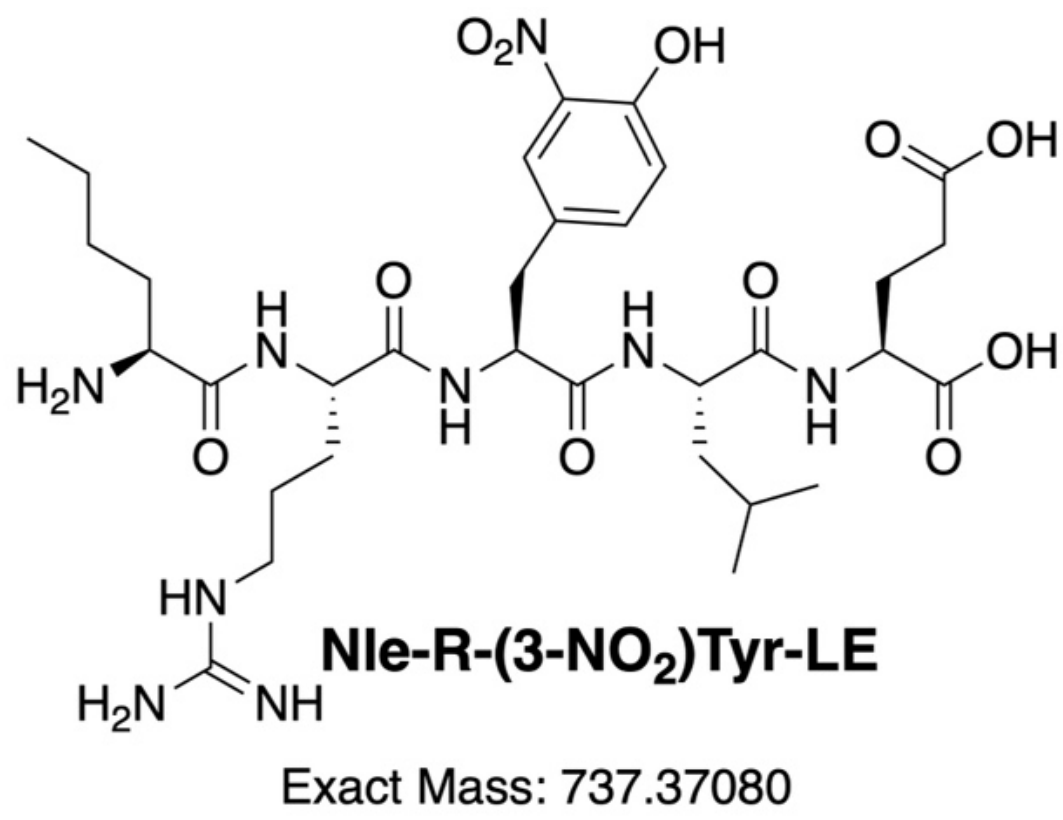

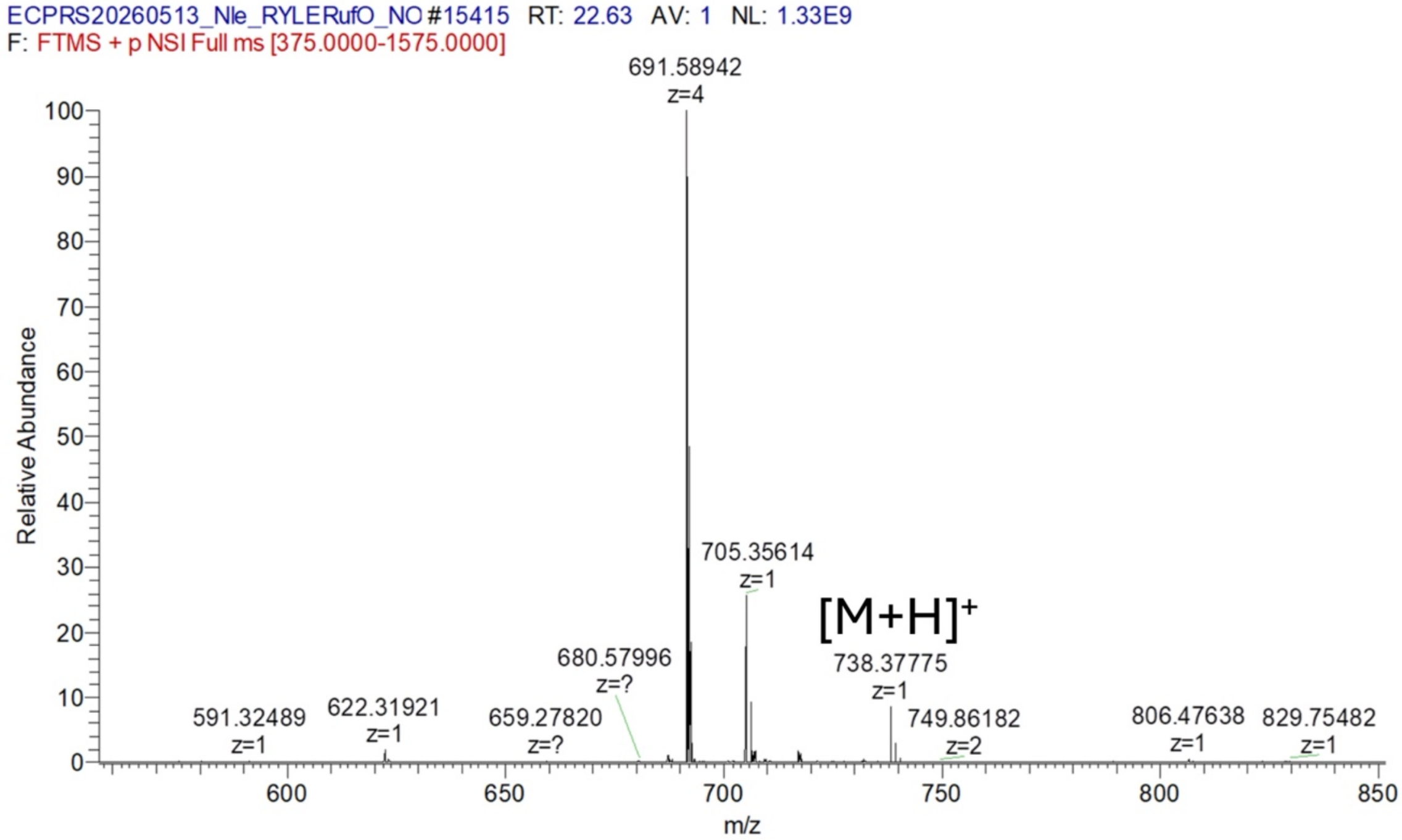

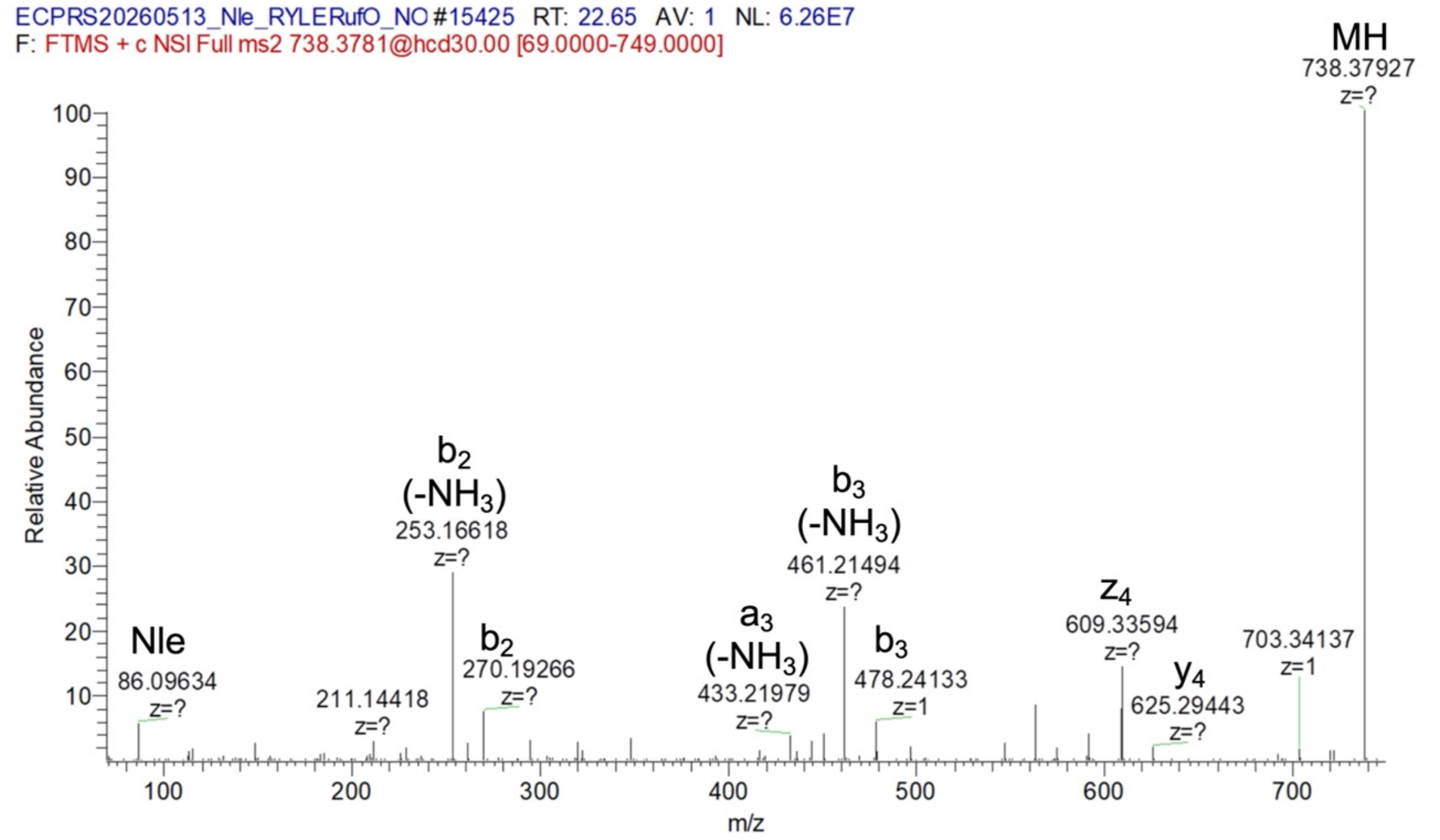

Figure S79. HRMS and MS[2] spectra for the nitrated product of the turnover of Nle-RYL**E** with RufO.

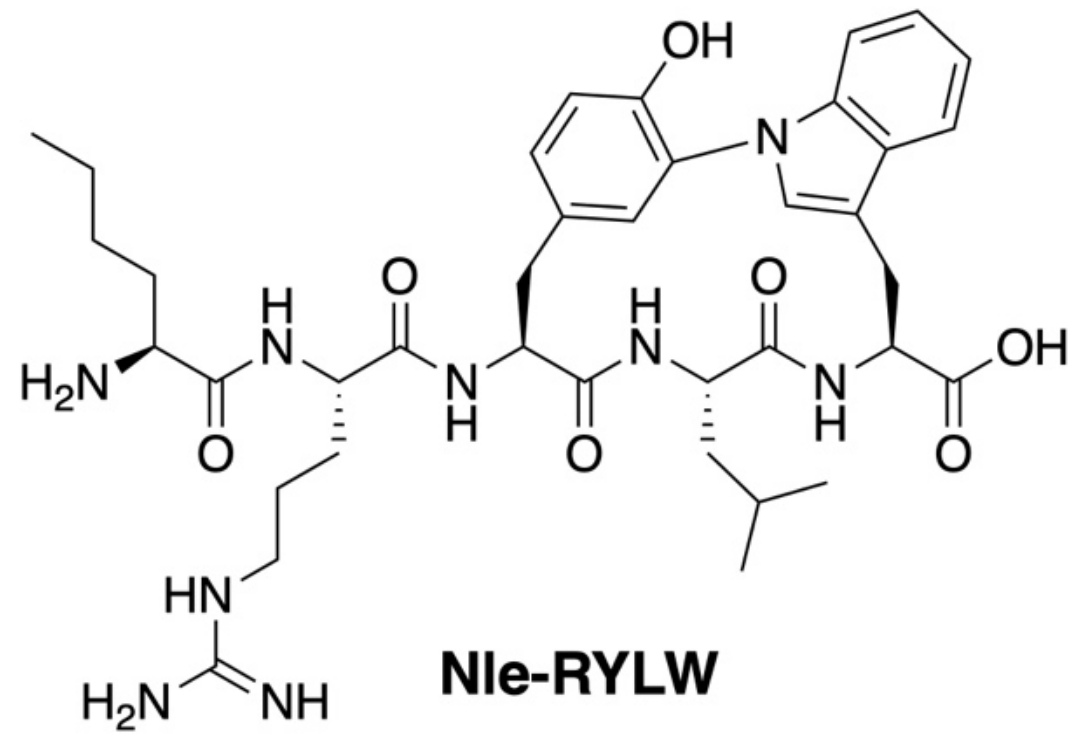

Exact Mass: 747.40680

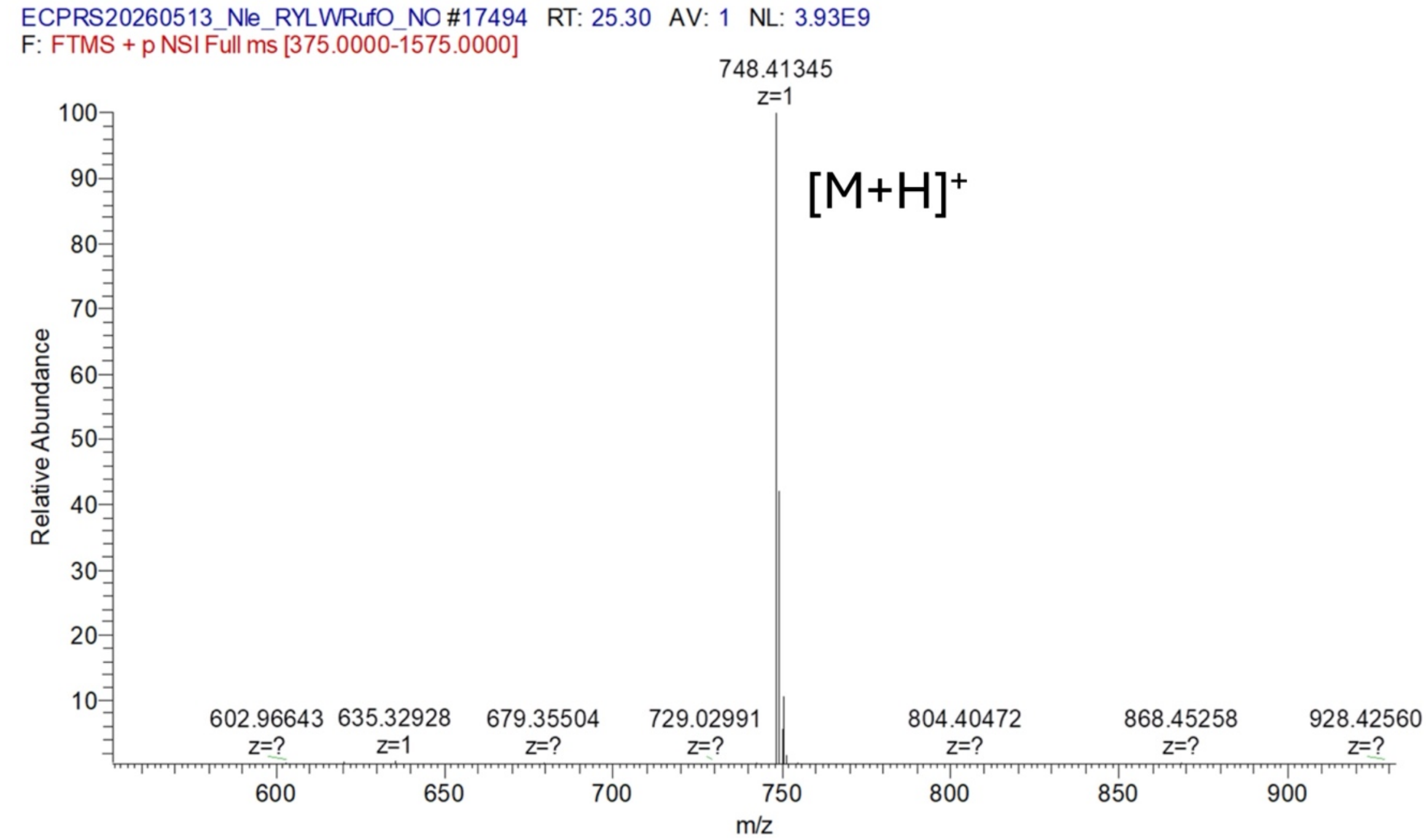

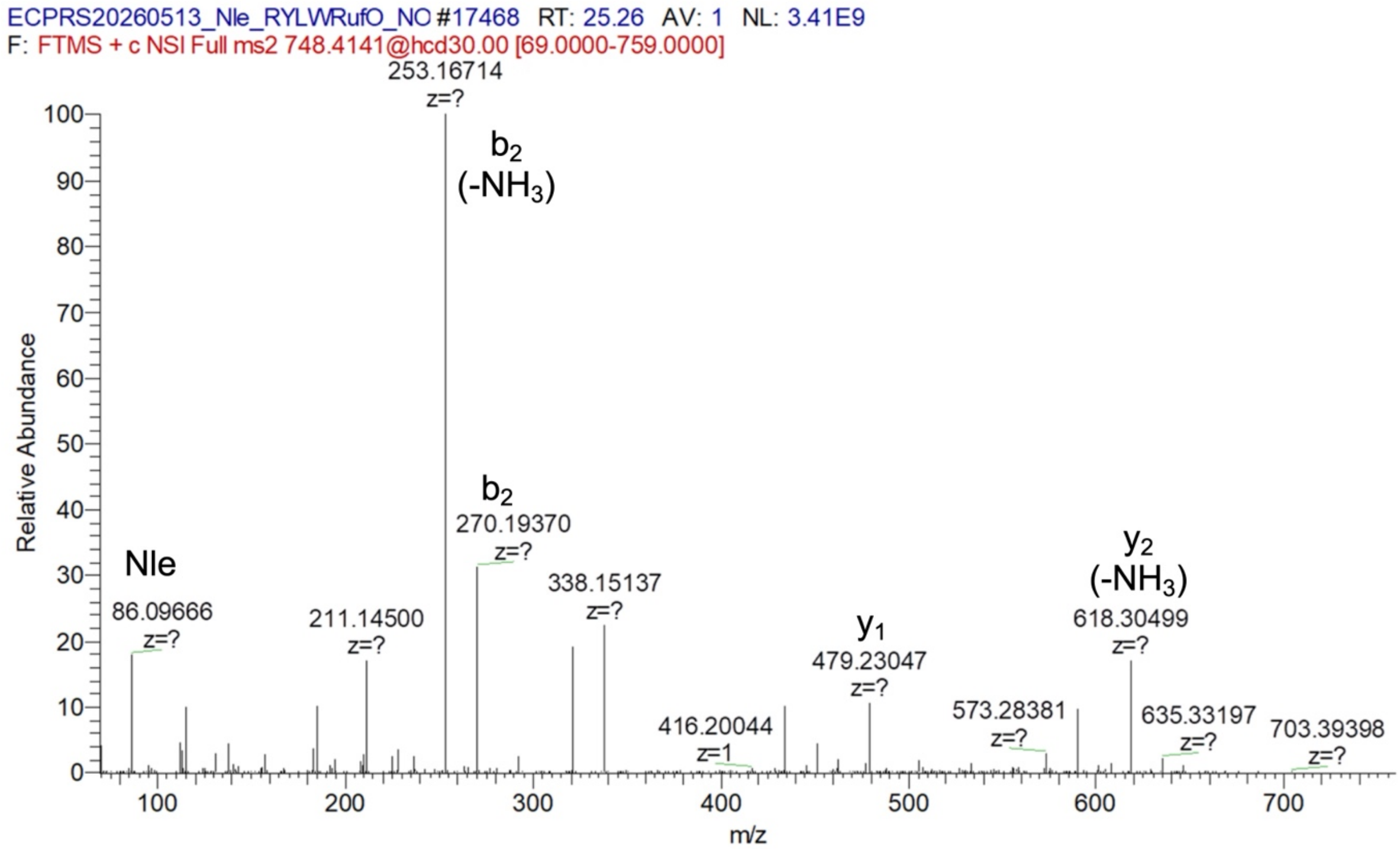

Figure S80. HRMS and MS[2] spectra for the crosslinked product of the turnover of Nle-RYL**W** with RufO.

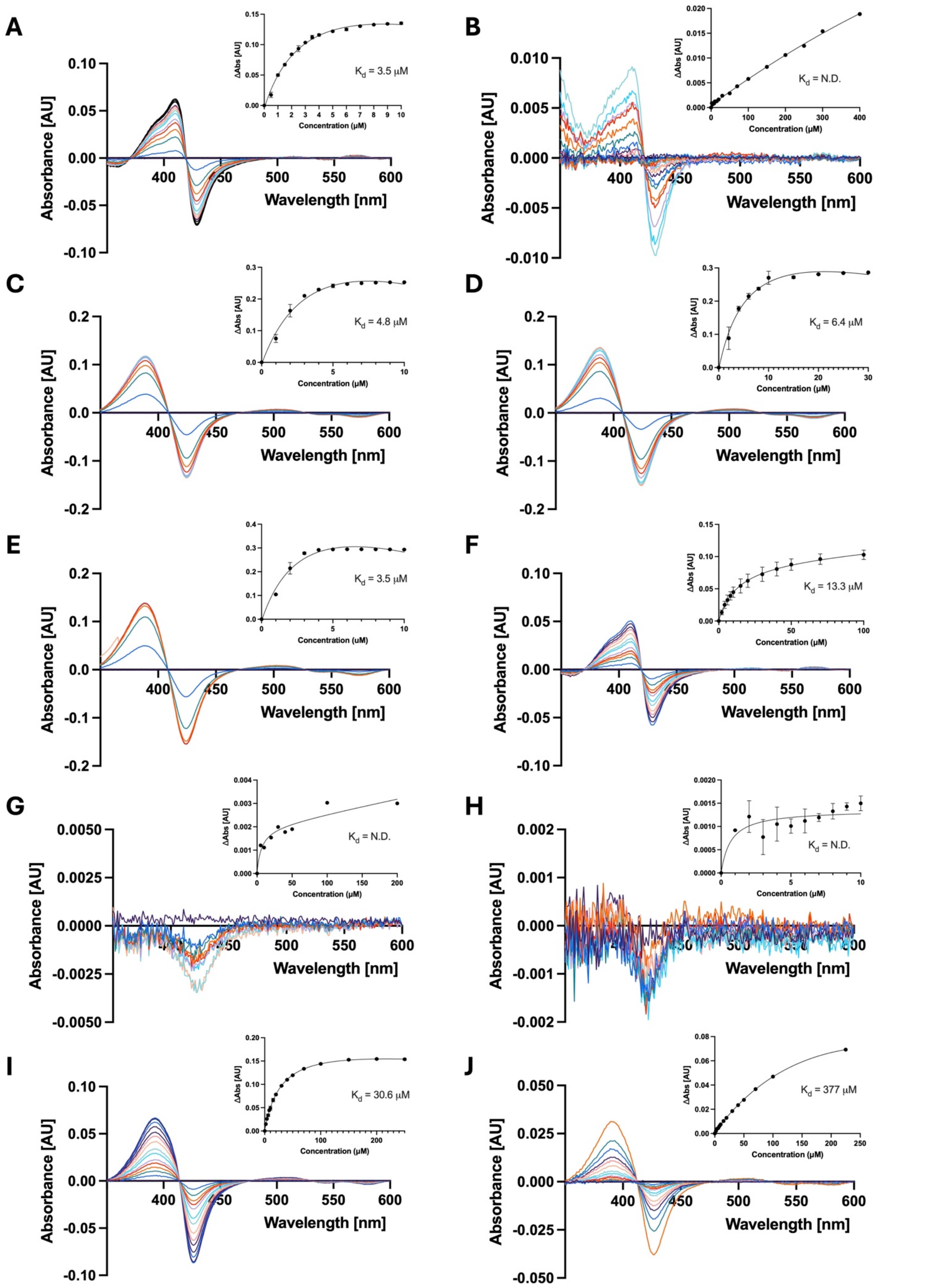

Figure S81. Binding curves to RufO for a) Nle-RYLH, b) Nle-**A**YLH, c) Nle-R**F**LH, d) Nle-R-**(OMe)Tyr**-LH, e) Nle-R-**mTyr**-LH, f) Nle-RY**Y**H, g) Nle-RYL**E**, h) Nle-RYL**Q**, i) Nle-RYL-**Thz** and j) Nle-RYL**W**. Data shown as UV-visible spectrum of titration of varying substrate concentrations with inset of ΔAbs (Abs_{max} – Abs_{min}) against concentration with error bars and K_d values shown, n = 2 for a, c, d, e, f, h, i otherwise n = 1. N.D. indicates where K_d could not be determined.

Table S1. Comparison of ΔAbs_{max}, K_d and $\Delta Abs_{max}/K_d$ as an indication of binding efficiency of peptides to RufO.

**Peptide**	**ΔAbs_{max}**	**K_d (μM)**	**$\Delta Abs_{max}/K_d$ ($\times 10^{-3}$)**
Nle-RYLH	0.135	3.53	38.2
Nle-**A**YLH	0.019	N.D.	-
Nle-R**F**LH	0.253	4.80	52.7
Nle-R-**(OMe)Tyr**-LH	0.287	6.42	44.7
Nle-R-**mTyr**-LH	0.293	3.59	81.6
Nle-RY**Y**H	0.102	13.33	7.7
Nle-RYL**E**	0.003	N.D.	-
Nle-RYL**Q**	0.001	N.D.	-
Nle-RYL-**Thz**	0.153	30.61	5.0
Nle-RYL**W**	0.069	377.4	0.2

Analysis of structural prediction of RufO

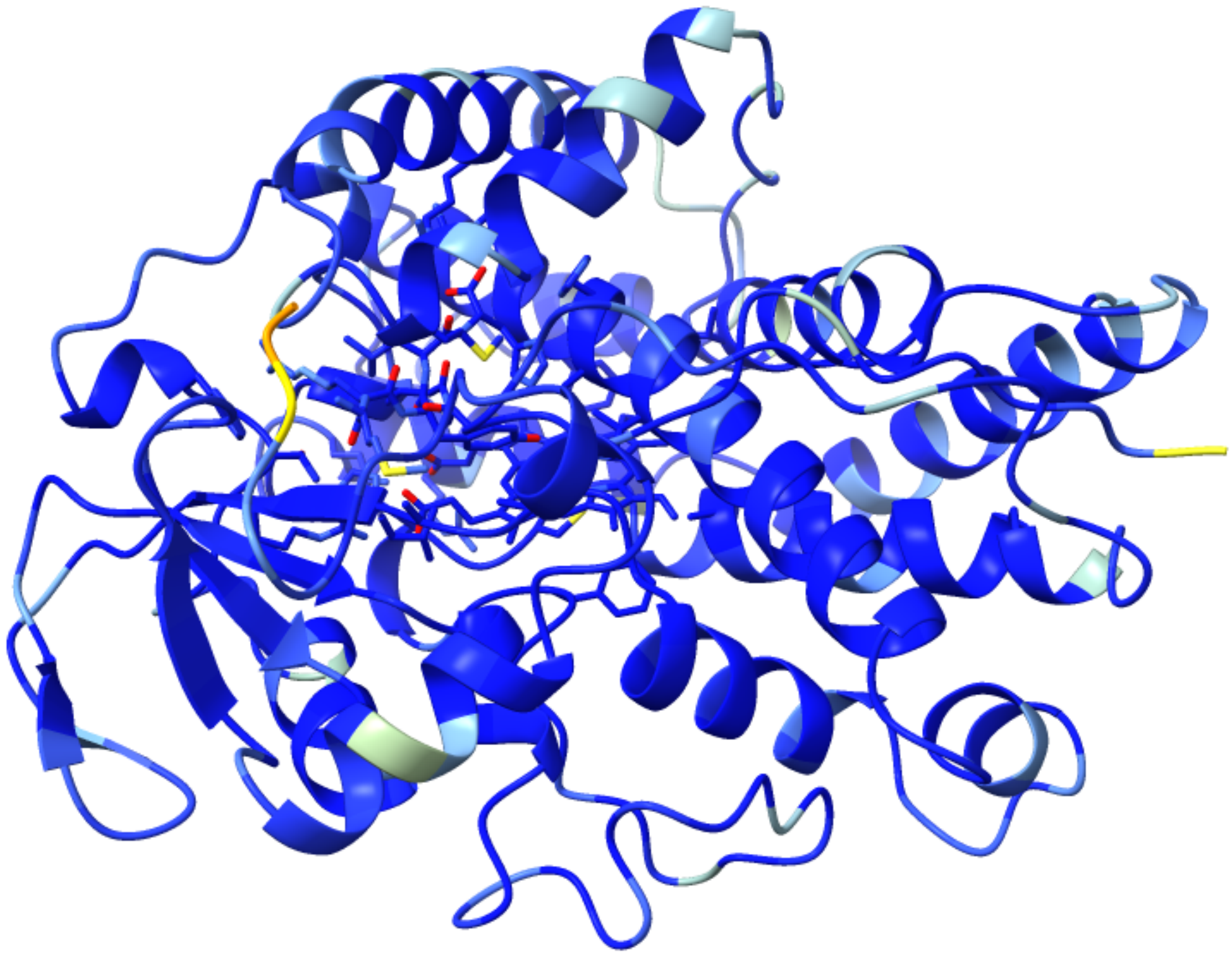

Figure S82. Predicted structure of MRYLH bound RufO with heme cofactor present obtained using the Alphafold Server (Alphafold3, 2024). Colour map showing pLDDT confidence measure of the prediction: red (0), orange (50), yellow (70), light blue (90), blue (100). Note that without the substrate present, the confidence of the F-G region was much lower. Residues 1-29 of the N-terminus truncated for visual clarity.

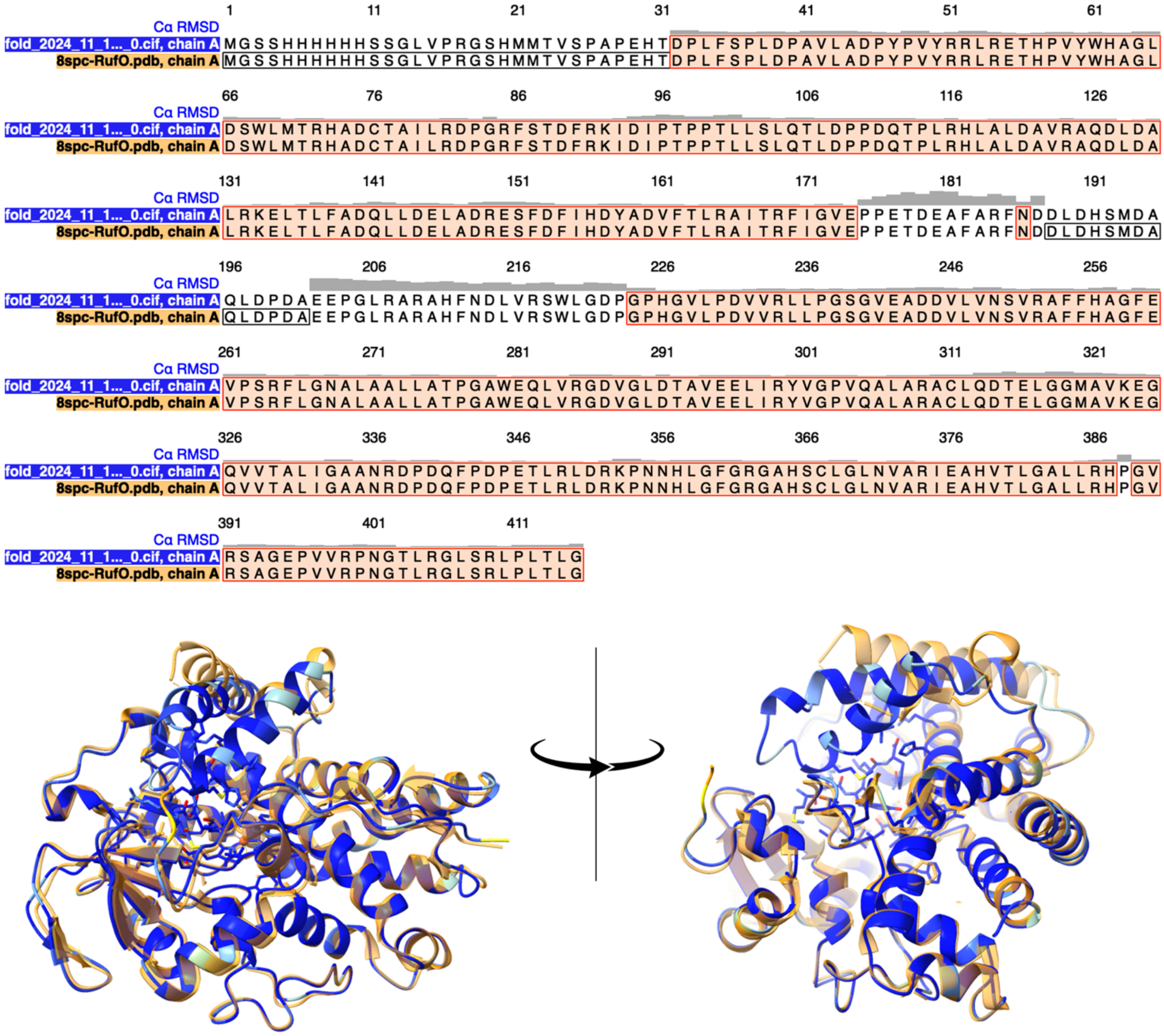

Figure S83. Comparison of alphafold3 structure (blue) and PDB 8SPC (orange). Matchmaker: RMSD between 335 pruned atom pairs is 0.652 angstroms; (across all 370 pairs: 1.603)

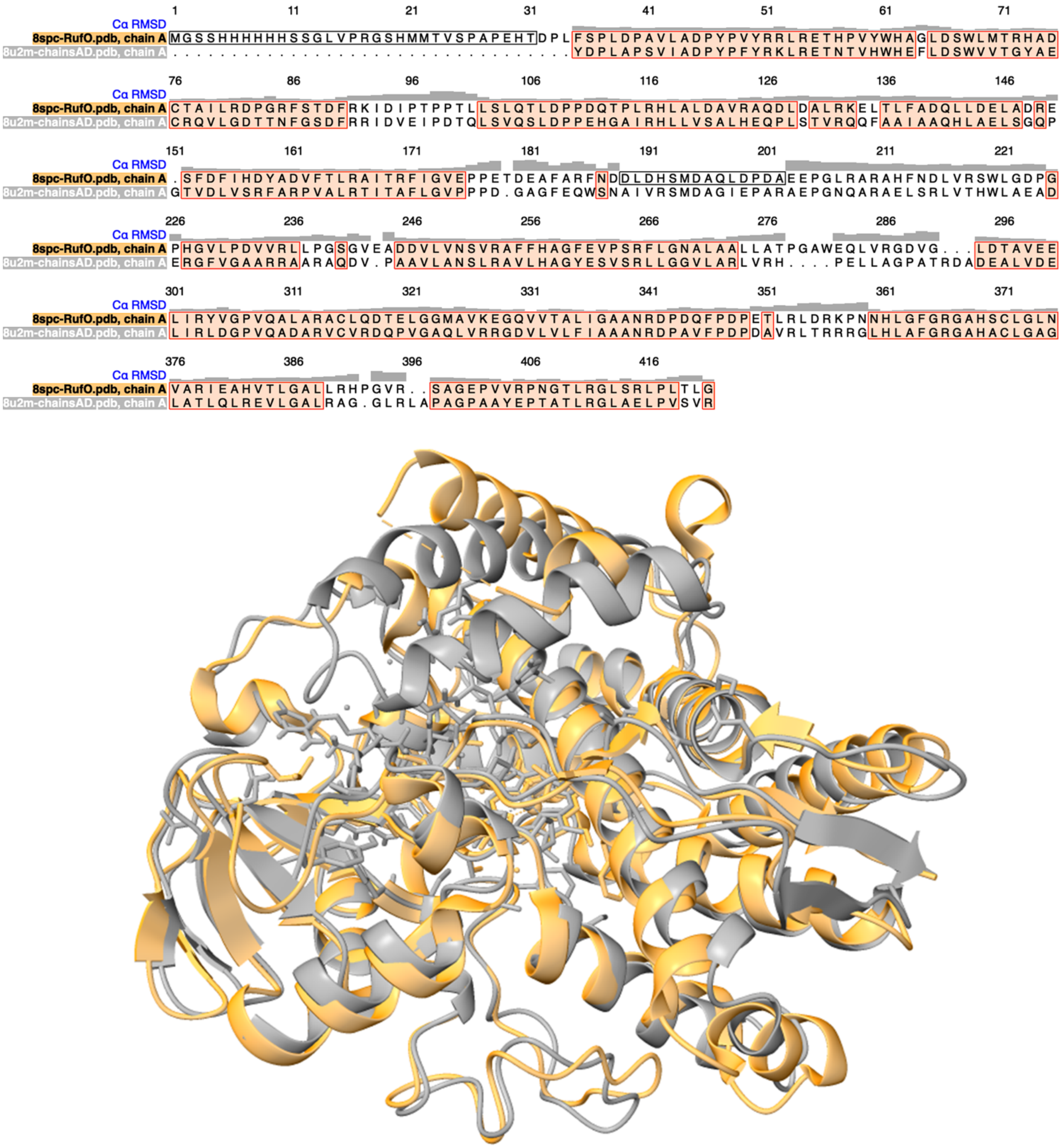

Figure S84. Comparison of $P450_{Blt}$ PDB 8U2M (grey) and RufO PDB 8SPC (orange). Matchmaker: RMSD between 273 pruned atom pairs is 1.016 angstroms; (across all 360 pairs: 2.128)

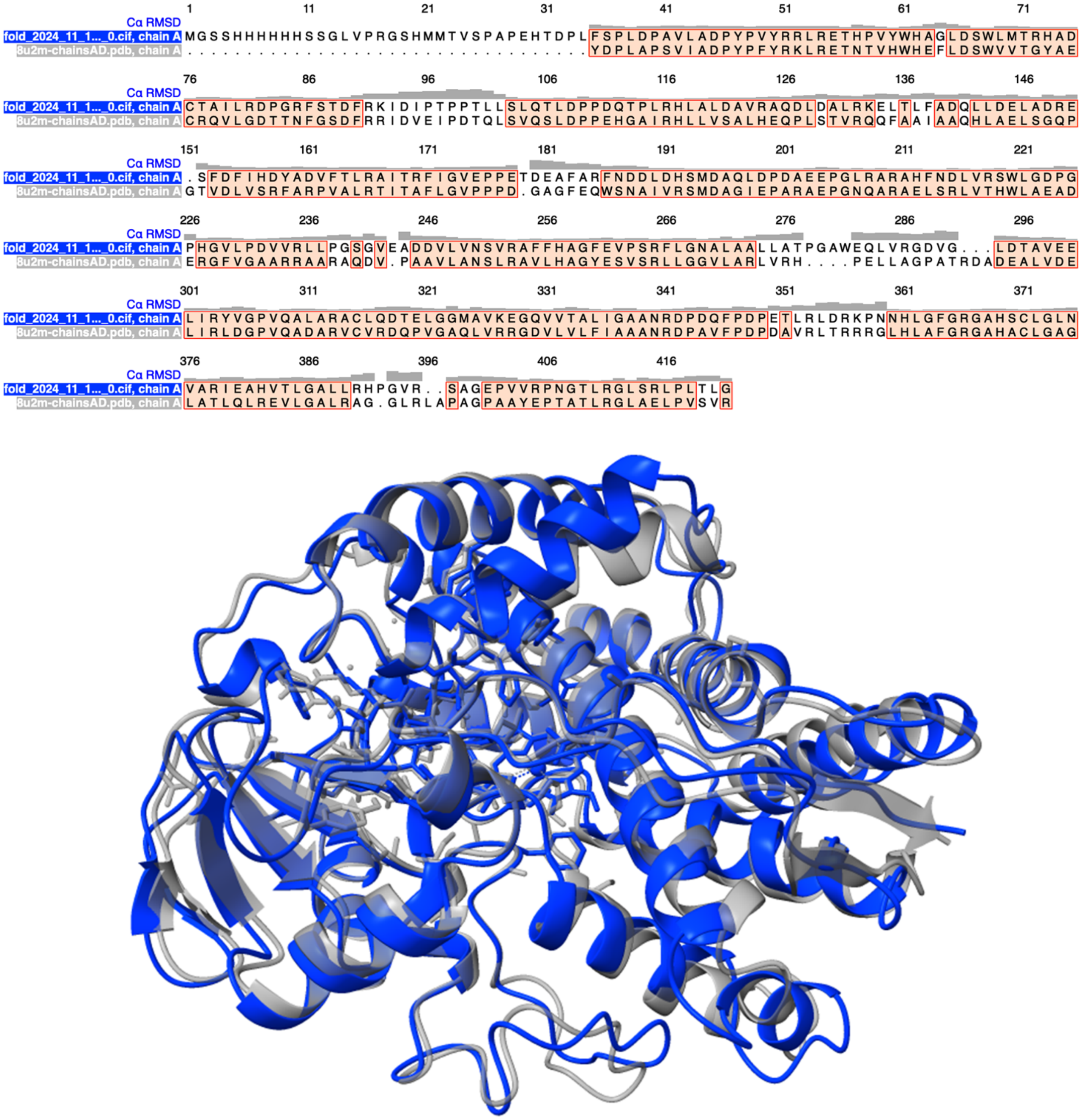

Figure S85. Comparison of $P450_{Blt}$ PDB 8U2M (grey) and RufO alphafold prediction (blue). Matchmaker: RMSD between 312 pruned atom pairs is 0.967 angstroms; (across all 374 pairs: 1.746)

Small DFT models of nitration catalysed by RufO

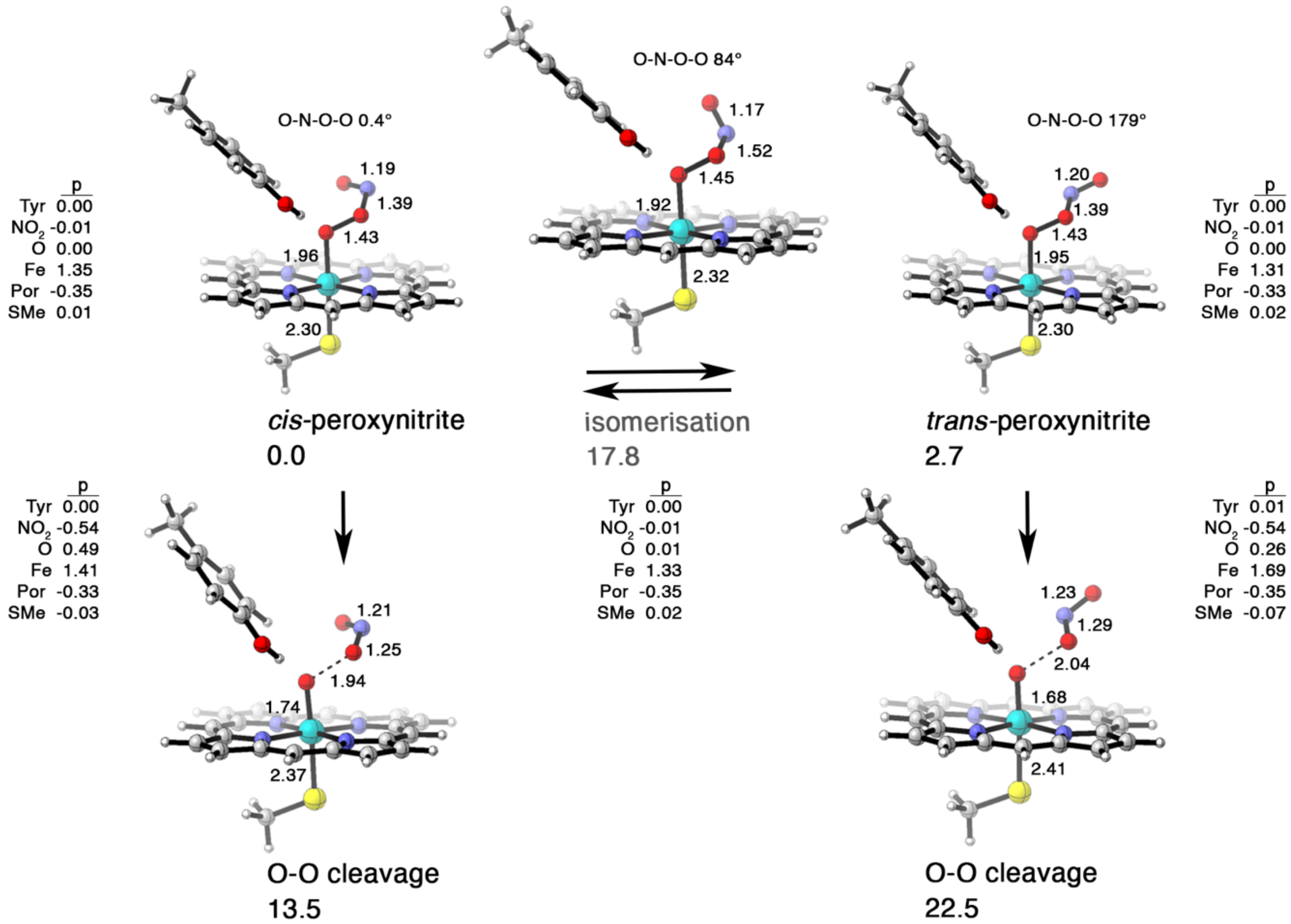

Figure S86. Barrier for isomerisation of *cis*-peroxynitrite to *trans*-peroxynitrite found to be substantial but lower in energy than O-O cleavage of the *trans*-peroxynitrite. Thus, the s*cis*-peroxynitrite is expected to be the key reactive intermediate. E+ZPE given in kcal mol^{-1} relative to the cis-peroxynitrite species and calculated using a truncated model at the B3LYP-D3BJ/6-311+G(d,p)LanL2DZ(Fe)//B3LYP/6-31G(d)LanL2DZ(Fe) level of theory. Mulliken spin densities (ρ) listed. Key bond distances (Å) and the O-N-O-O dihedral angle (º) annotated in black.

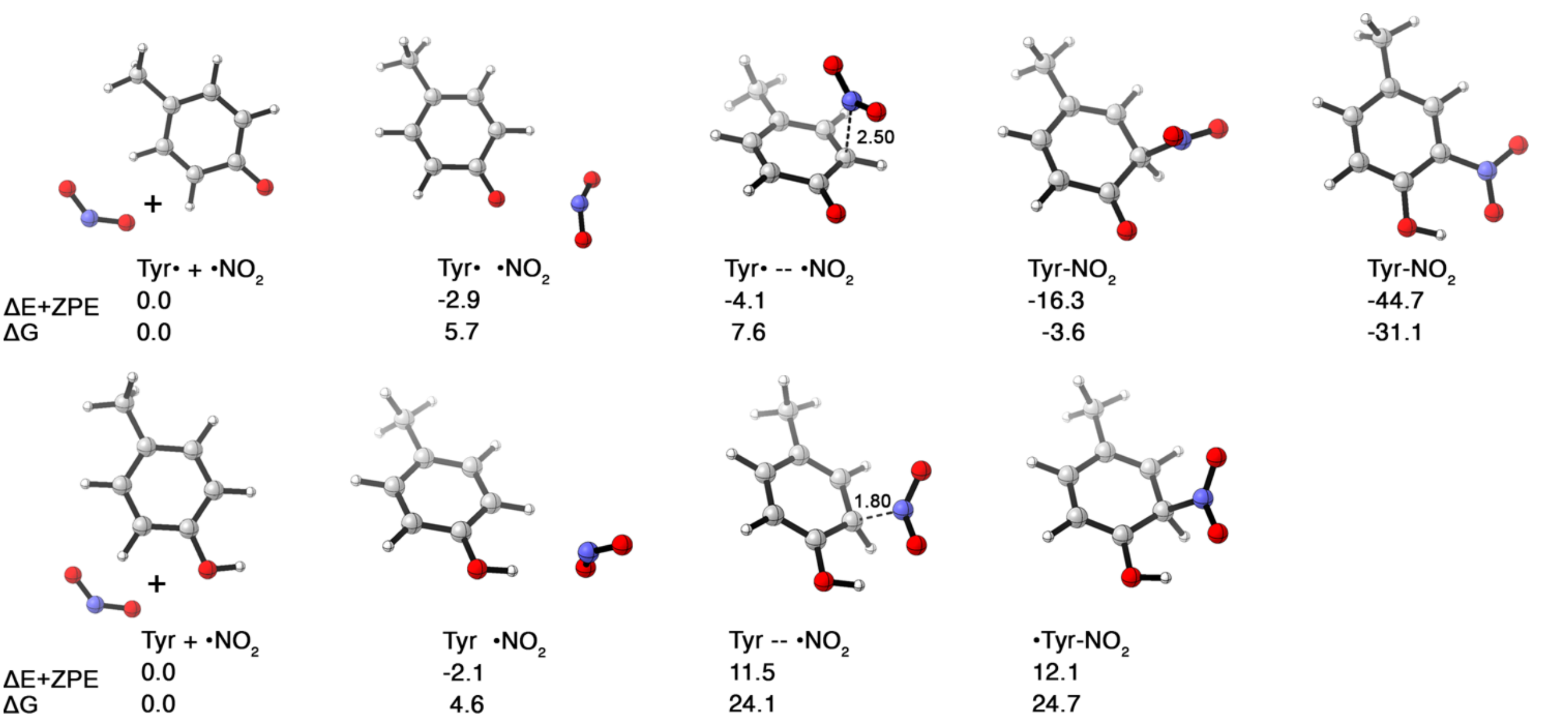

Figure S87. Gas phase radical recombination and isomerization compared with radical addition. E+ZPE and G given in kcal mol^{-1} relative to the respective intermediates and calculated using a truncated model at the B3LYP-D3BJ/6-311+G(d,p)LanL2DZ(Fe)//B3LYP/6-31G(d)LanL2DZ(Fe) level of theory.

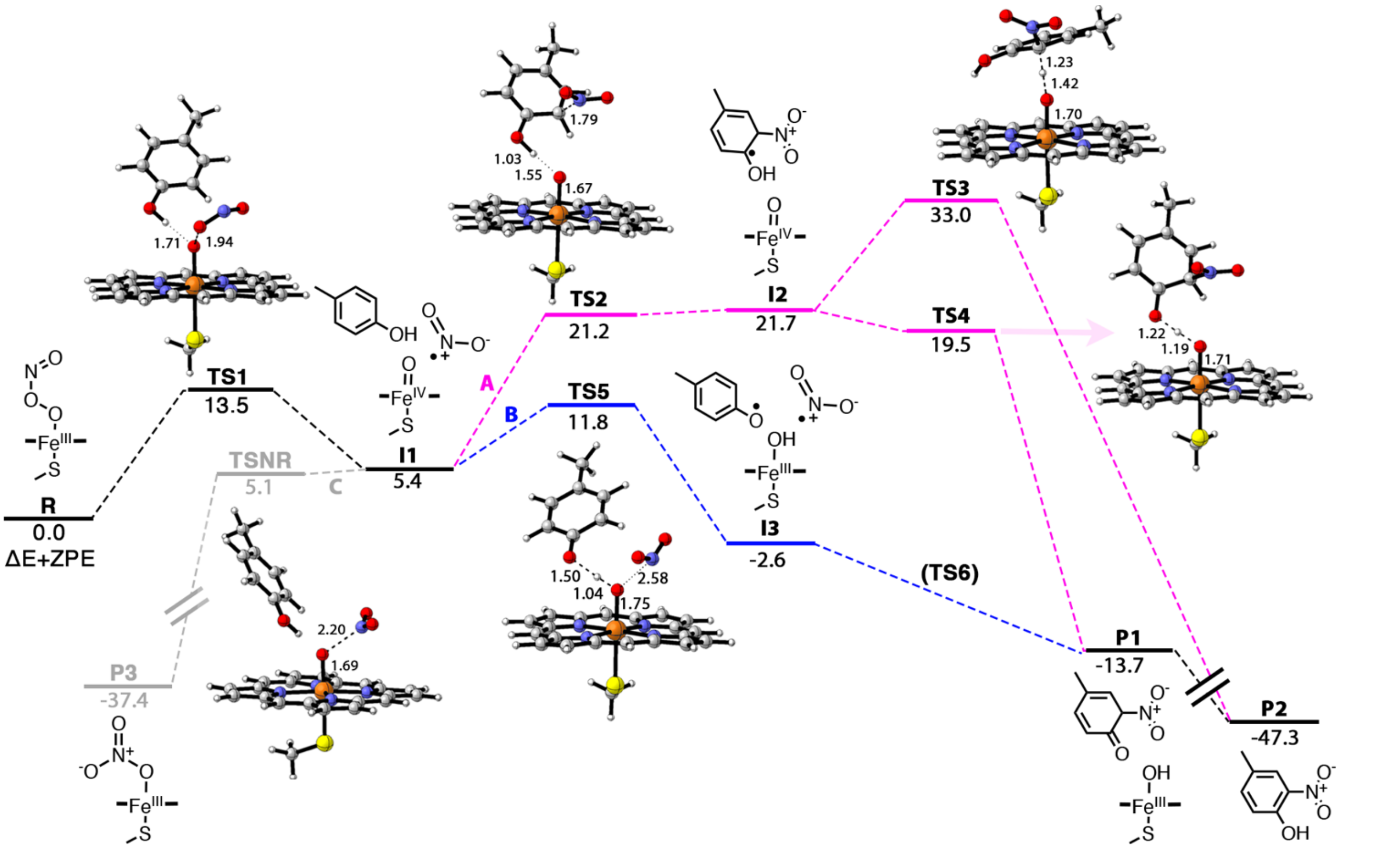

Figure S88. Potential mechanisms of nitration following formation of $^{\bullet}NO_2$ from homolytic cleavage of the ferric peroxynitrite. Path A (pink) Radical addition (RA) of $^{\bullet}NO_2$ followed by hydrogen atom abstraction (HAA). Path B (blue) Phenolic hydrogen atom abstraction (HAA) followed by radical recombination (RR). Path C (grey) N-O radical rebound to form nitrate as a byproduct. Potential energies calculated using a truncated model at the B3LYP-D3BJ/BS2//B3LYP/BS1 level of theory, S=1/2, with zero-point-energy corrections (E+ZPE) are given in kcal mol^{-1}. Key Mulliken spin densities and geometries for these structures listed in Tables S2 and S3.

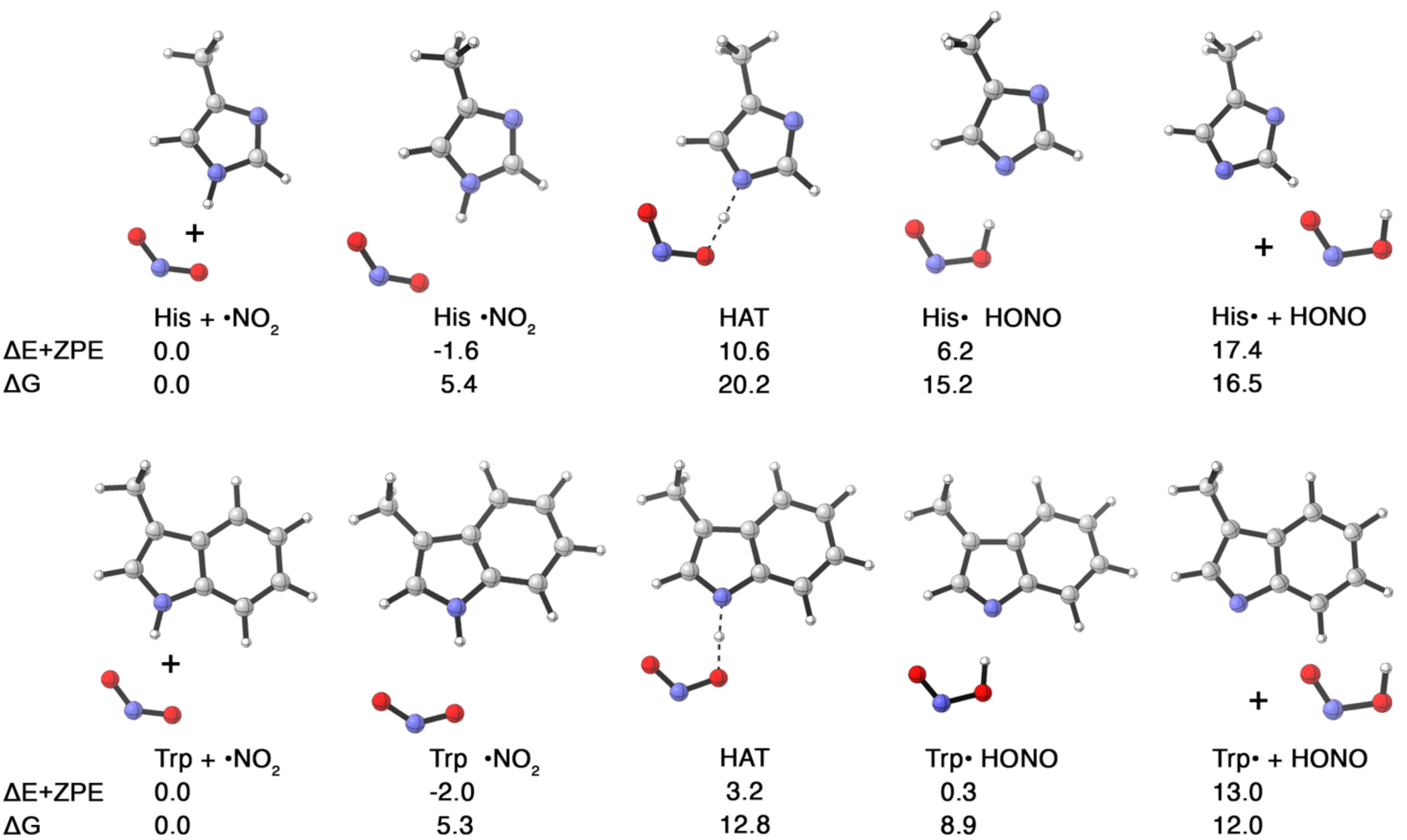

Figure S89. Abstraction of N-**H** by $^{\bullet}NO_2$ radical, comparing methylated indole and imidazole as representative of tryptophan and histidine. E+ZPE and G given in kcal mol^{-1} relative to the respective reactants at the B3LYP-D3BJ/6-311+G(d,p)LanL2DZ(Fe)//B3LYP/6-31G(d)LanL2DZ(Fe) level of theory in the gas phase.

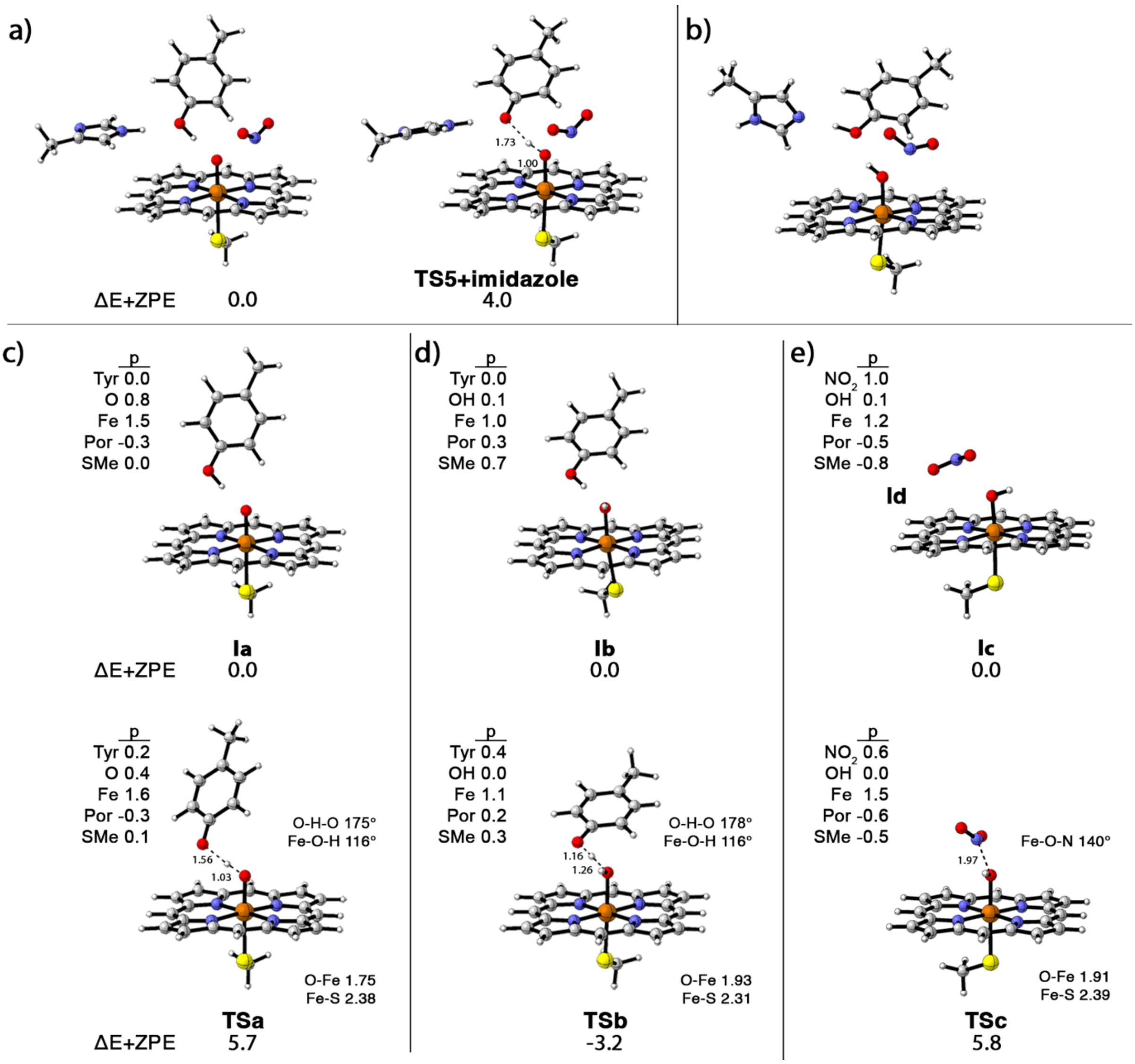

Figure S90. Phenol hydrogen abstraction by a) Cpd II with hydrogen bonding from imidazole, c) deprotonated Cpd II or d) Cpd II. b) Inclusion of a hydrogen bonding protonated imidazole results in barrierless proton transfer from protonated imidazole to dp-CpdII through phenol forming CpdII. e) Radical rebound of ·NO_2 with Cpd II to form nitrate. E+ZPE given in kcal mol^{-1} relative to the respective intermediates and calculated using a truncated model at the B3LYP-D3BJ/6-311+G(d,p)LanL2DZ(Fe)//B3LYP/6-31G(d)LanL2DZ(Fe) level of theory in the doublet spin state. Key bond distances (Å) and angles (°) labelled. Mulliken spin densities (ρ) listed for thiolate (SMe), porphine (Por), Fe, proximal oxygen/hydroxyl (O/OH), $^{\bullet}NO_2$, and 4-methylphenol (Tyr), else refer to Table S2.

Here, modifying the model by including imidazole as a H-bond donor to the phenol reduces the barrier of phenolic H-abstraction by 2.4 kcal mol^{-1} compared to **TS5** and elongates the breaking O-H bond from 1.50 Å to 1.73 Å giving the TS high proton transfer character (Figure S90a, see also spin density in Table S2). Inclusion of protonated imidazole in the gas phase resulted in electronically barrierless proton transfers to protonate Cpd II via phenol (Figure S90b), and subsequent H-abstraction of the phenolic hydrogen by protonated Cpd II in the doublet spin state was calculated to be barrierless (Figure S90d) while the barrier for rebound of $^{\bullet}NO_2$ to form nitric acid increased to $\Delta E+ZPE^{\ddagger} = 5.8$ kcal mol^{-1} (Figure S90e).

Table S2. Key Mulliken spin densities for the different calculated species using the truncated model with DFT at the B3LYP-D3BJ/BS2//B3LYP/BS1 level of theory. This includes the thiolate (SMe), porphine (Por), Fe, proximal oxygen (O), $^{\bullet}NO_2$, and 4-methylphenol (Tyr).

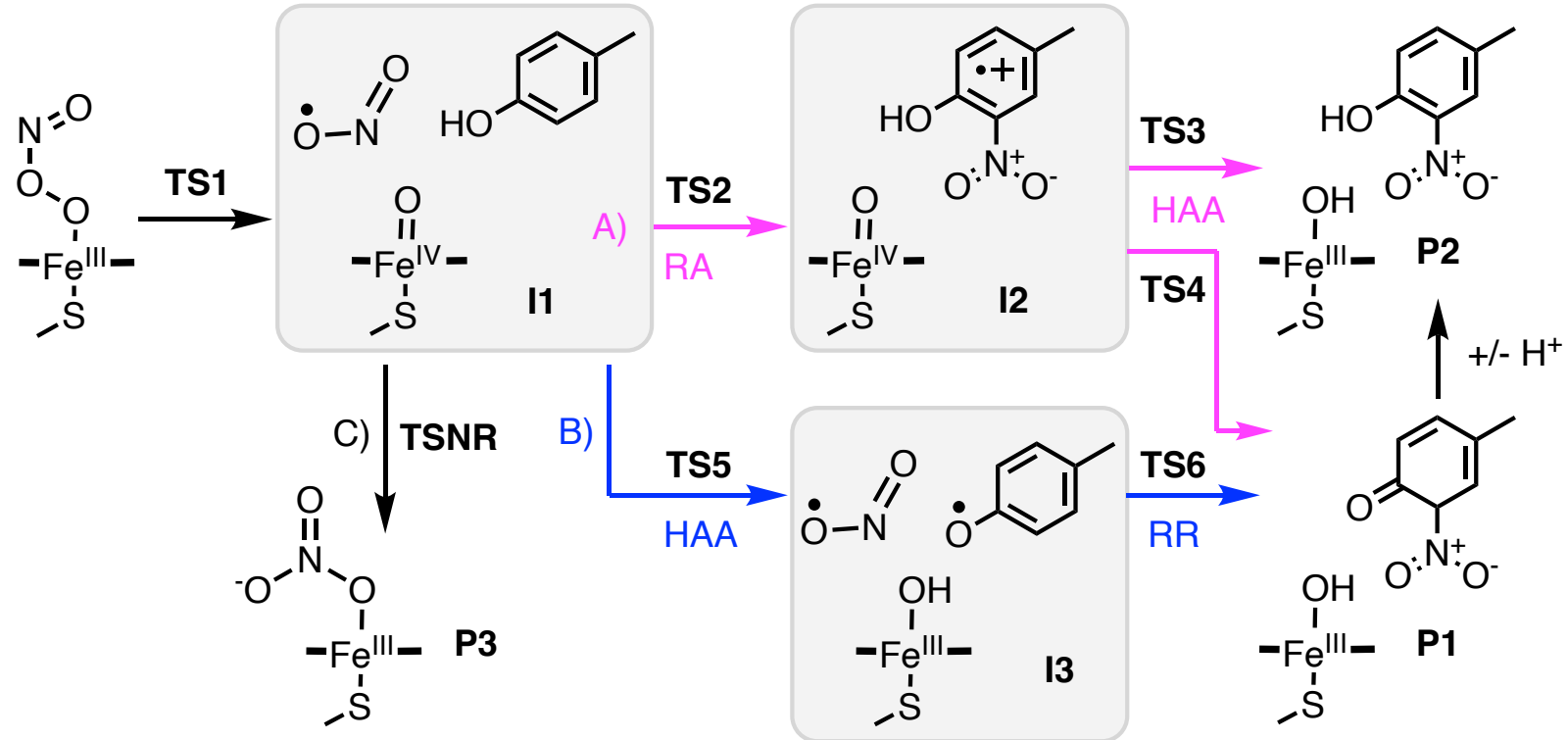

	SMe	Por	Fe	O	NO2	Tyr
***cis* reactant (R)**	0.0	-0.4	1.3	0.0	0.0	0.0
**TS1**	0.0	-0.3	1.4	0.5	-0.5	0.0
**I1**	-0.1	-0.3	1.6	0.7	-0.9	0.0
**TS2**	0.0	-0.3	1.5	0.8	-0.1	-0.9
**I2**	0.0	-0.3	1.5	0.8	-0.2	-0.8
**TS3**	0.0	-0.4	1.7	0.6	-0.1	-0.9
**TS4**	0.0	-0.3	1.4	0.7	0.0	-0.8
**P1**	0.0	-0.3	1.2	0.0	0.0	0.0
**TS5**	0.1	-0.3	1.7	0.4	-0.9	0.1
**I3**	0.0	-0.3	1.3	-0.1	-0.8	1.0
**NR**	0.0	-0.3	1.5	0.6	-0.8	0.0
**P3**	0.0	-0.4	1.4	0.0	0.0	0.0
**TS5+imidazole**	0.1	-0.4	1.9	0.3	-1.0	0.0

Table S3. Key bond distances (Å) and bond angles (°) for the structures calculated using the truncated model with DFT at the B3LYP-D3BJ/BS2//B3LYP/BS1 level of theory.

	S-Fe	Fe-O	O-O	FeO-N	N-C	FeO-H	H-O(Tyr)	Fe-O-X	X-H-O
**R**	2.30	1.96	1.43		4.22	1.71	1.00	123 (H)	178 (O)
**TS1**	2.37	1.74	1.94			1.71	1.00		
**I1**	2.44	1.67		2.47	4.05	1.69	1.00	124 (N)	
**TS2**	2.43	1.68			1.79	1.55	1.03	130 (H)	173 (O)
**I2**	2.43	1.68			1.63	1.55	1.03	139 (H)	173 (O)
**TS3**	2.39	1.71			1.62	1.19	1.23	122 (H)	176 (O)
**TS4**	2.44	1.70			1.54	1.42	1.23	129 (H)	169 (C)
**P1**	2.34	1.89			1.52	0.97		108 (H)	
**TS5**	2.39	1.75		2.58	4.29	1.04	1.50	118 (H)	177 (O)
**I3**	2.33	1.90		2.38	5.74	0.97		105 (H)	
**TSNR**	2.41	1.69		2.20		1.70		123 (N)	
**P3**	2.28	2.09		1.32		1.85		129 (N)	

Analysis of molecular dynamics simulations

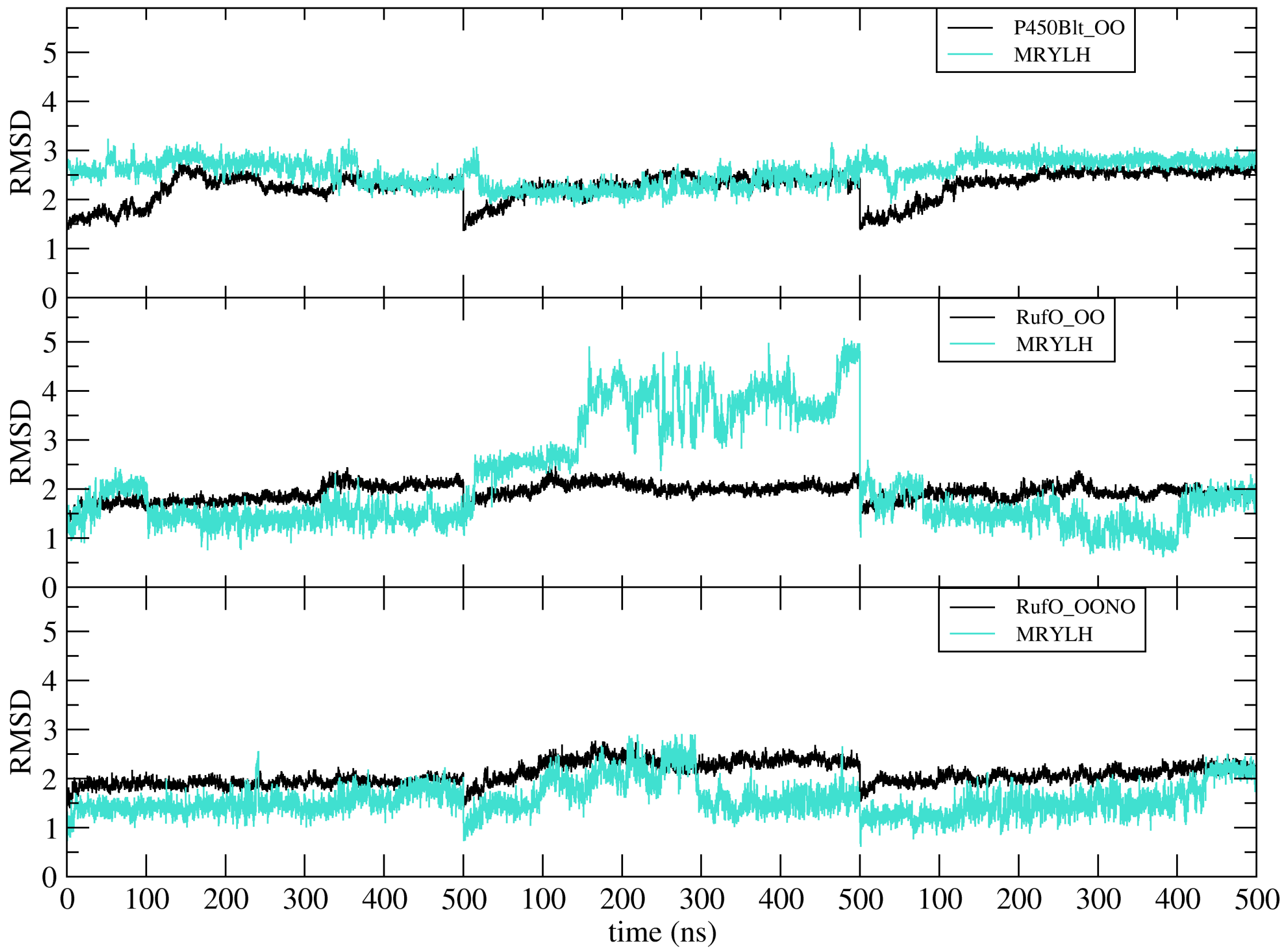

Figure S91. RMSD (Å) of protein (black) and substrate MRYLH (turquoise) for triplicate MD simulations of $P450_{Blt}$ (top) and RufO (middle) in the ferric-superoxo intermediate state and RufO in the ferric-peroxoynitrite state (bottom). The minimized structures from the set-up of the MD simulations are used as the reference structures.

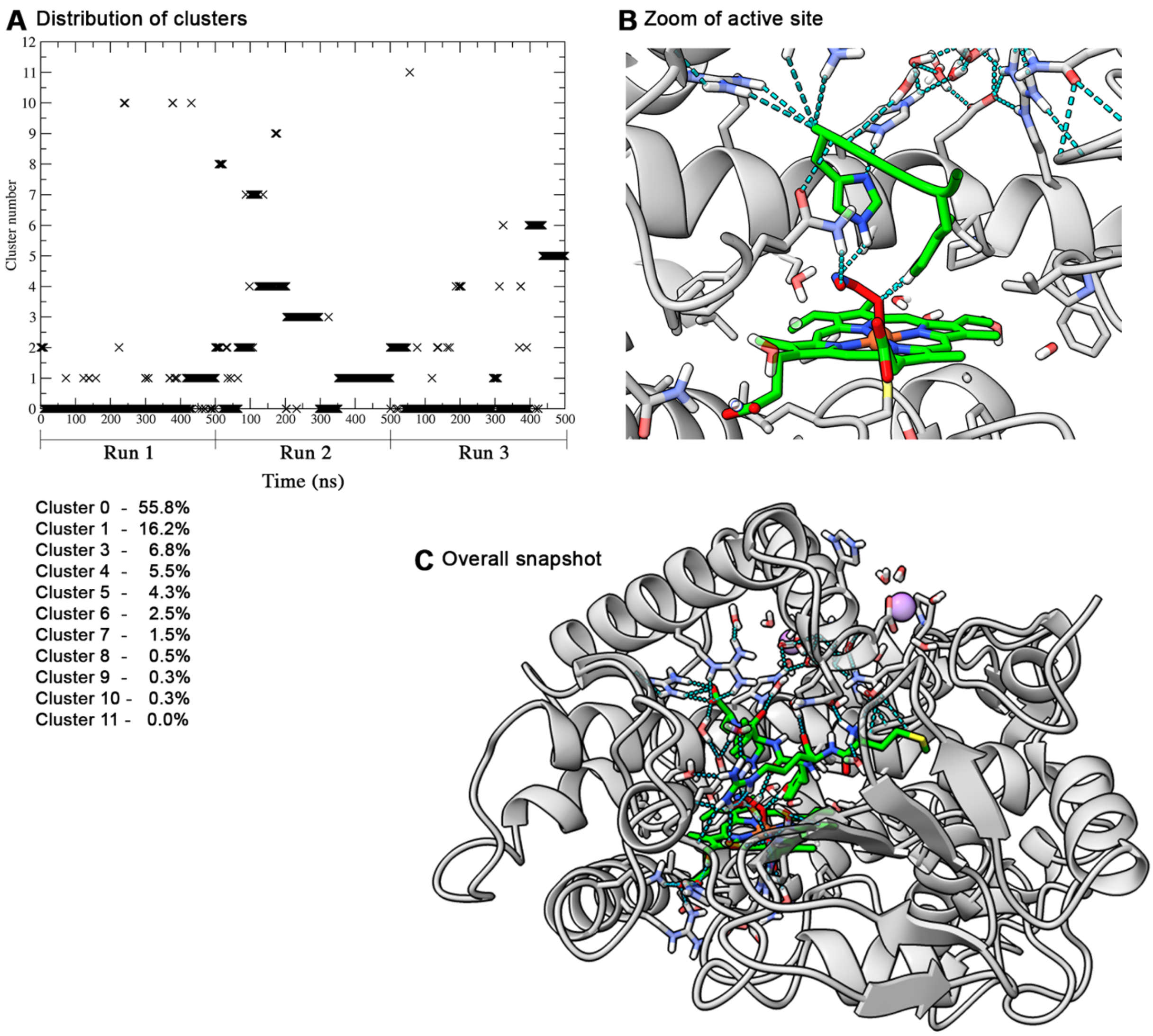

Figure S92. Representative snapshot from the major cluster in MD simulations chosen for ONIOM model.a) Distribution of clusters over time (ns) in triplicate MD runs. b) Zoomed in view of the active site residues in the representative snapshot of cluster 0 at 276.4 ns. c) Overall view of the protein for the selected snapshot. MRYLH substrate and heme highlighted in green. Note sodium ions interacting near the F-helix shown in purple. Hydrogen bonds shown with blue dashed lines.

Table S4. Percentage of MD snapshots in which specified hydrogen bonds are present based on an acceptor donor distance ≤3.0 Å and angle ≥135°. Mean % ± standard deviation for triplicate runs reported. RufO was modelled with both the ferric peroxynitrite intermediate and ferric superoxol. $P450_{Blt}$ modelled with the ferric superoxol is provided for comparison. Note a/b used to denote H-bonds to alternate carboxylate oxygens while only one is shown diagrammatically. In * denotes $P450_{Blt}$ alternate conformation of R230 which involves a different nitrogen in the h-bonds. Greyed residues and numbering in brackets refers to $P450_{Blt}$.

H-bond definitions	H-bond	RufO Fe(III)OONO	RufO Fe(III)OO	$P450_{Blt}$ Fe(III)OO
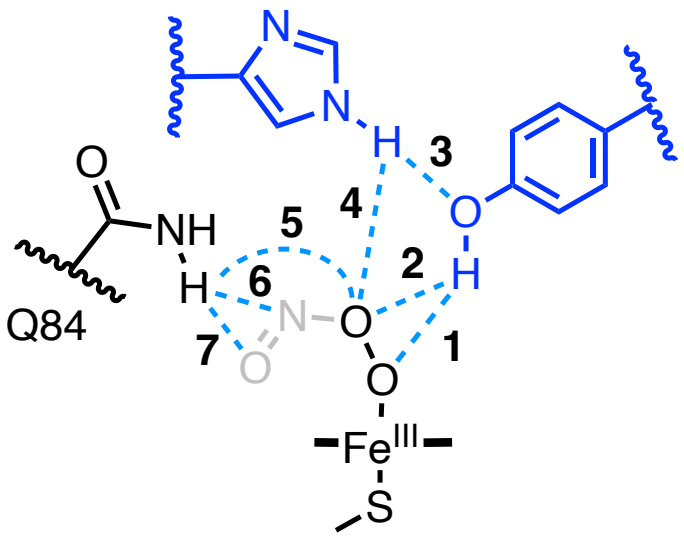	**1**	60 ± 13	15 ± 2	11 ± 0.5
	**2**	3 ± 1	18 ± 3	3 ± 0.1
	**3**	12 ± 6	16.0 ± 0.4	3.7 ± 0.6
	**4**	1 ± 1	9 ± 1	11 ± 0.3
	**5**	0	10.5 ± 0.4	0
	**6**	1 ± 1	na	na
	**7**	9 ± 1	na	na
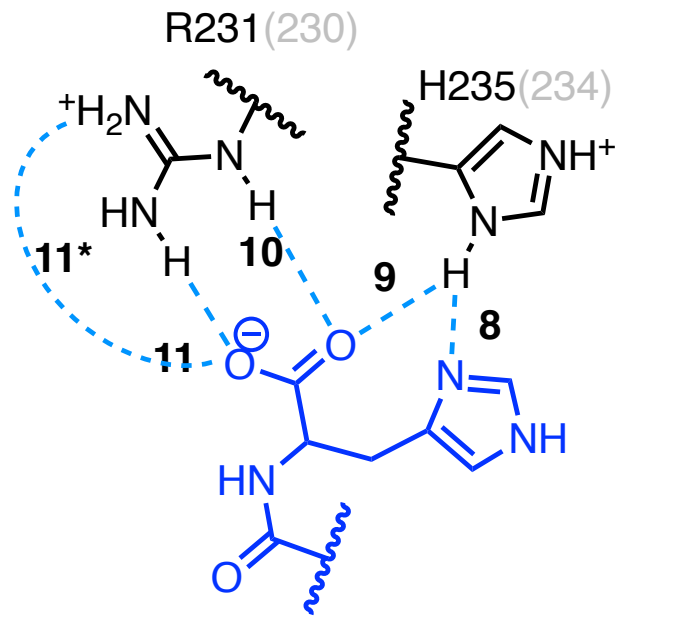	**8**	60 ± 12	56 ± 3	82 ± 1
	**9a**	4 ± 4	7 ± 3	4 ± 1
	**9b**	2 ± 2	7 ± 1	4 ± 1
	**10a**	37 ± 5	31 ± 2	0
	**10b**	36 ± 6	32 ± 1	0
	**11a**	52 ± 2	50 ± 1	*36 ± 6
	**11b**	49 ± 2	46 ± 1	*45 ± 7
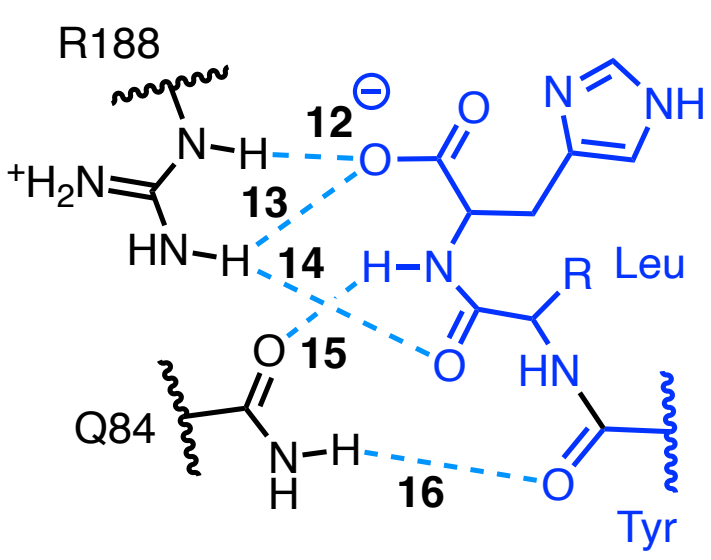	**12a**	41 ± 7	33 ± 0.2	43 ± 2
	**12b**	41 ± 1	36 ± 2	37 ± 2
	**13a**	31 ± 2	27 ± 1	16 ± 1
	**13b**	35 ± 7	31 ± 2	11 ± 3
	**14**	0	0	47 ± 3
	**15**	21 ± 7	30 ± 5	64 ± 2
	**16**	10 ± 6	12 ± 2	51 ± 5
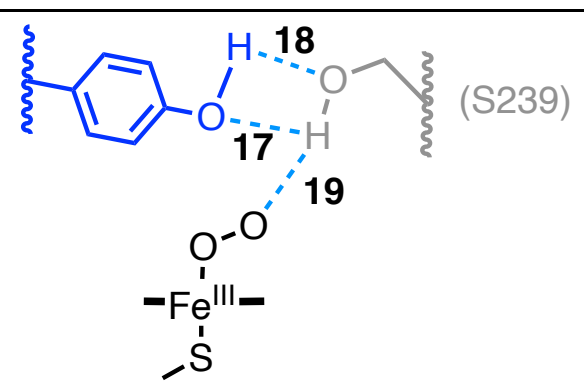	**17**	na	na	70 ± 4
	**18**	na	na	0
	**19**	na	na	4 ± 1
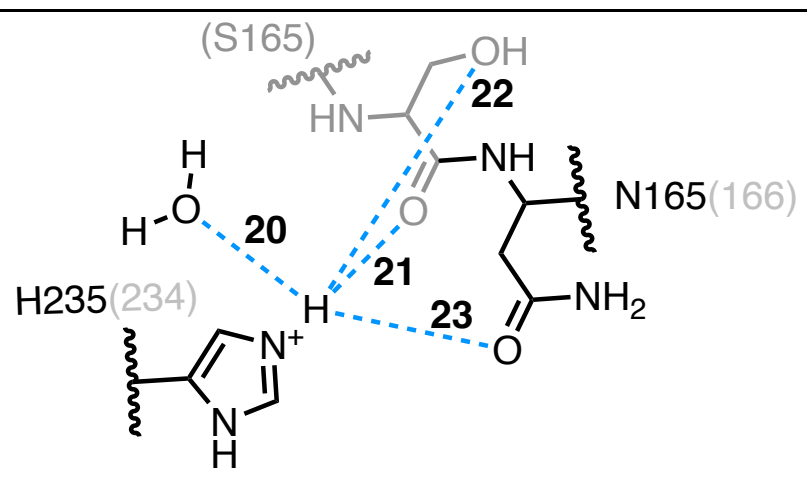	**20**	37 ± 27	29 ± 2	< 0.01
	**21**	na	na	65 ± 2
	**22**	na	na	2 ± 0.5
	**23**	24 ± 26	47 ± 3	0

Table S5. Summary of combination of protonation states explored in MD simulations of RufO and NsP450 using both predicted structures and crystal structures for initial conformations as noted. Standard HIP, HIE, HID, GLU, GLH nomenclature used

	RufO								NsP450
Initial structure:	AlphaFold3					X-ray			X-ray
	**1**	**2**	**3**	**4**	**5**	**6**	**7**	**8**	**9**
H235	HIE	HIE	HIP	HIP	HIP	HIP	HIE	HIP	HIP
His-5	HID	HIP	HIE	HIP	HIE	HIE	HIP	HIP	HIP
E239	GLU	GLU	GLU	GLU	GLH	GLH	GLH	GLU	GLU

ONIOM model of nitration catalysed by RUFO

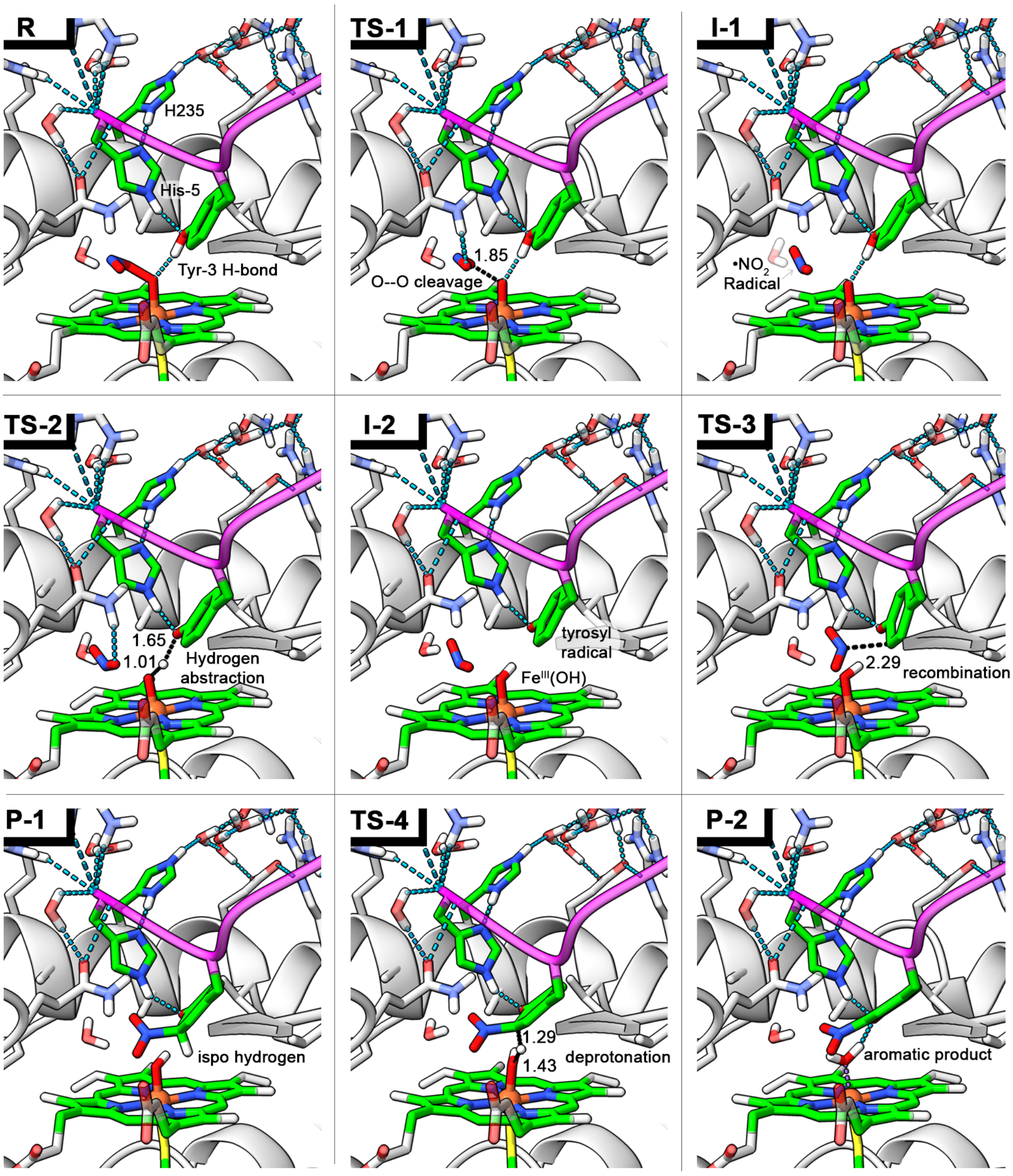

Figure S93. Geometries of stationary points calculated using ONIOM(DFT:MM) for the potential mechanisms of RufO catalyzed nitration (black pathway). High layer shown in green. MM layer shown in grey. MRYLH substrate highlighted in pink. H-bonds shown in blue dotted lines, TS bonds in black dotted lines. Key bond distances labelled in Å. Annotations added for clarity.

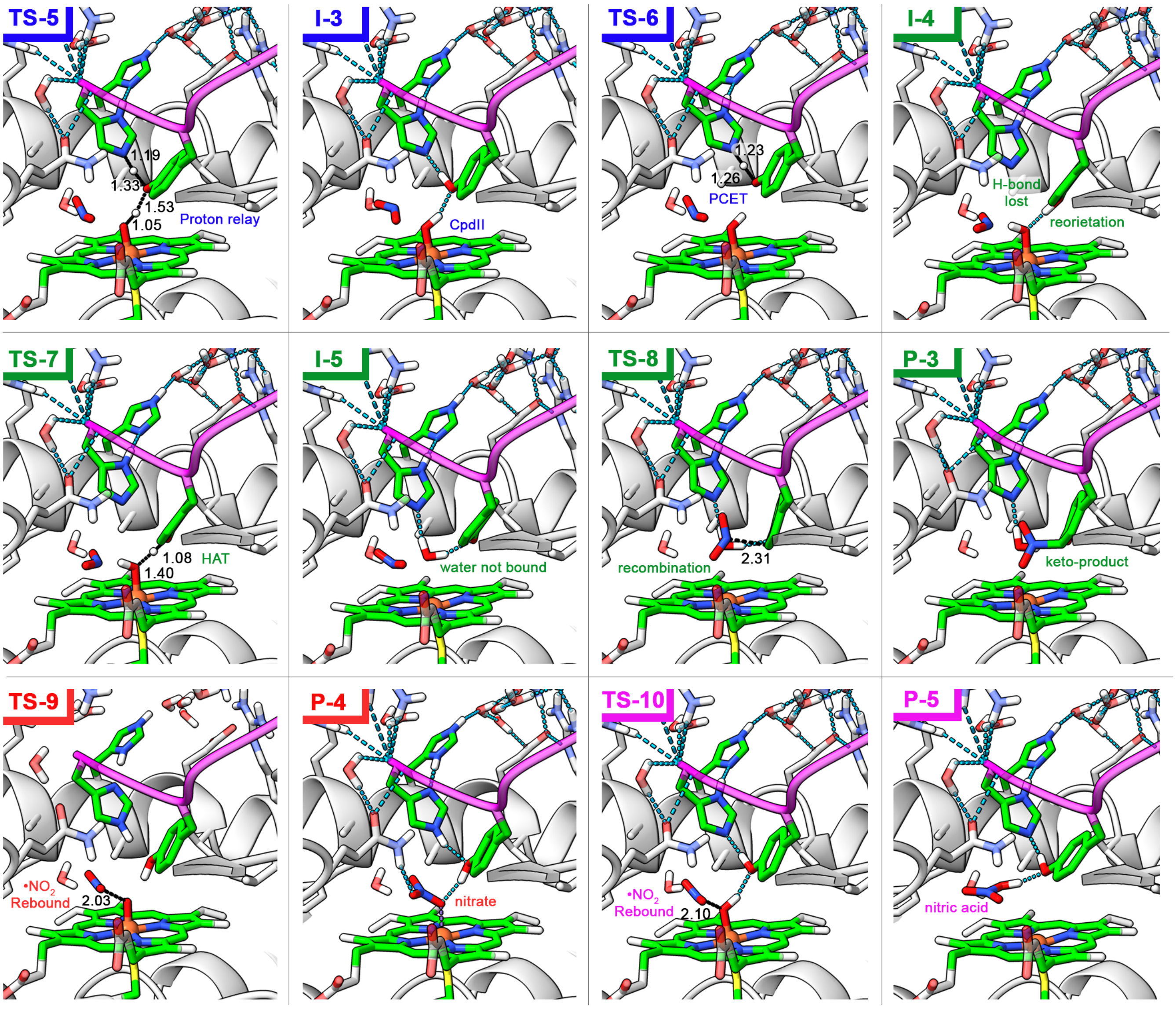

Figure S94. Geometries of stationary points calculated using ONIOM(DFT:MM) for alternate mechanisms of RufO catalyzed nitration involving a proton relay and for nitrate and nitric acid formation. High layer shown in green. MM layer shown in grey. MRYLH substrate highlighted in pink. H-bonds shown in blue dotted lines, TS bonds in black dotted lines. Key bond distances labelled in Å. Annotations added for clarity.

Table S6. Key Mulliken spin densities and energies (Hartree) for the different calculated species using ONIOM(DFT:MM) at the B3LYP/BS4//B3LYP/BS3:Amber FF14SB level of theory. Spin densities from the high layer are assorted into the thiolate (SMe), porphyrin (Por), Fe, proximal oxygen (O, unprotonated or protonated), $^{\bullet}NO_2$, I-helix histidine sidechain (H235), substrate histidine sidechain (His-5) and tyrosine sidechain (Tyr-3). PES from Figure 7 shown for clarity.

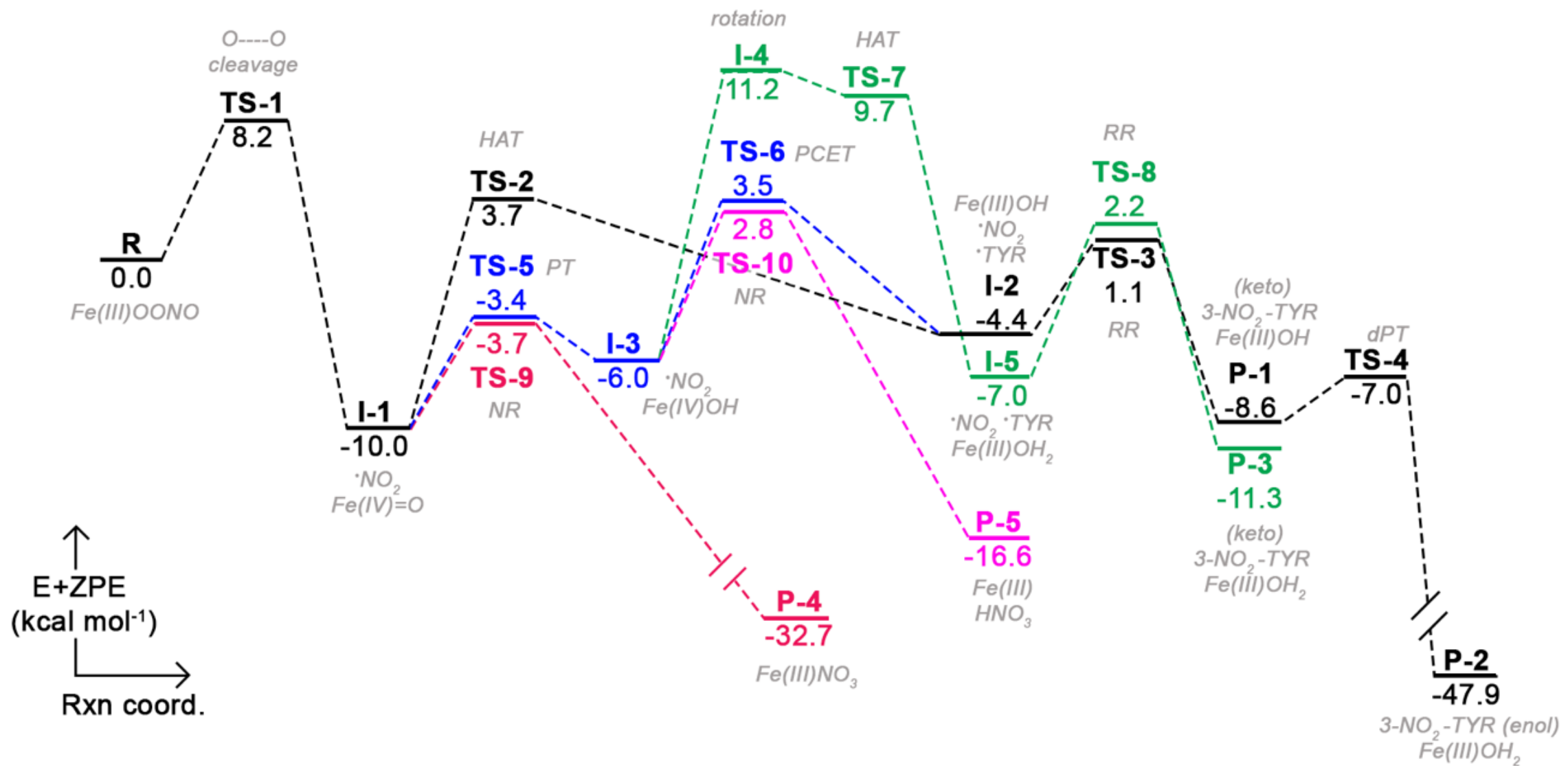

	SMe	Por	Fe	O/OH	NO2	Tyr-3	His-5	H235	Imaginary frequencies	ZPE	E	H	G
**R**	-0.1	-0.1	1.1	0.0	0.0	0.0	0.0	0.0	-	12.468025	-3885.672811	-3872.487130	-3873.897191
**TS-1**	-0.1	-0.1	1.3	0.2	-0.4	0.0	0.0	0.0	-576.1	12.466565	-3885.658213	-3872.474103	-3873.884461
**I-1**	0.0	-0.1	1.5	0.6	-0.9	0.0	0.0	0.0	-	12.467484	-3885.688273	-3872.502337	-3873.914225
**TS-2**	0.0	-0.1	1.7	0.3	0.9	0.2	0.0	0.0	-1185.1	12.461044	-3885.659984	-3872.480561	-3873.893337
**I-2**	0.0	0.0	1.0	0.1	0.9	0.9	0.0	0.0	-	12.465323	-3885.677148	-3872.493138	-3873.905107
**TS-3**	0.0	-0.1	1.0	0.1	0.7	-0.7	0.0	0.0	-241.9	12.465233	-3885.668265	-3872.485181	-3873.894874
**P-1**	0.0	-0.1	1.0	0.1	0.0	0.0	0.0	0.0	-	12.470104	-3885.688593	-3872.501602	-3873.907955
**TS-4**	0.0	-0.1	1.1	0.0	0.0	0.0	0.0	0.0	-1166.1	12.466716	-3885.682707	-3872.500287	-3873.903092
**P-2**	0.0	-0.1	1.1	0.0	0.0	0.0	0.0	0.0	-	12.469735	-3885.750875	-3872.564577	-3873.970857
**TS-5**	-0.1	-0.1	1.9	0.3	1.0	0.0	0.0	0.0	-545.8	12.459371	-3885.669520	-3872.492069	-3873.904800
**I-3**	-0.1	-0.1	2.0	0.2	-1.0	0.0	0.0	0.0	-	12.465106	-3885.679389	-3872.495638	-3873.909255
**TS-6**	-0.1	-0.1	2.1	0.2	1.0	0.0	0.0	0.0	-1106.6	12.459105	-3885.658319	-3872.480688	-3873.894177
**I-4**	0.0	0.5	1.0	0.1	1.0	0.3	0.0	0.0	-	12.462942	-3885.649864	-3872.468134	-3873.881878
**TS-7**	0.0	0.3	1.1	0.0	1.0	0.6	0.0	0.0	-387.9	12.460864	-3885.650128	-3872.471192	-3873.882736
**I-5**	0.3	0.0	2.8	0.0	1.0	-1.0	0.0	0.0	-	12.464422	-3885.680306	-3872.496172	-3873.912857
**TS-8**	0.3	0.0	2.8	0.0	-0.7	0.7	0.0	0.0	-231.6	12.465169	-3885.666464	-3872.482934	-3873.894848
**P-3**	0.3	0.0	2.8	0.0	0.0	0.0	0.0	0.0	-	12.469226	-3885.692044	-3872.504599	-3873.917371
**TS-9**	0.0	-0.1	1.4	0.4	-0.7	0.0	0.0	0.0	-310.0	12.467720	-3885.678413	-3872.493340	-3873.901871
**P-4**	-0.1	-0.1	1.2	0.0	0.0	0.0	0.0	0.0	-	12.471620	-3885.728548	-3872.539898	-3873.947997
**TS-10**	0.0	0.3	1.2	0.1	-0.6	0.0	0.0	0.0	-350.8	12.464405	-3885.664772	-3872.482529	-3873.893160
**P-5**	-0.1	-0.2	1.3	0.0	0.0	0.0	0.0	0.0	-	12.469462	-3885.700735	-3872.513935	-3873.924306

Table S7. Crystallographic data summary for NsRufO binding with the substrate (Nle-RYLH).

Data collection	
Space group	*P* 2_1 2_1 2_1
Unit-cell parameters (Å, °)	43.9760, 78.9890, 110.5420, 90.0000, 90.0000, 90.0000
Resolution range (Å)	45.29 - 1.59 (1.62 - 1.59)
Wavelength	1.007
Total observations (AIMLESS)	643578
Total unique (AIMLESS)	52525
Overall multiplicity	12.3
Mosaicity	0.112
Rmerge	0.055 (0.658)
I/sigma(I)	28.6 (2.3)
Completeness (%)	99.7
CC(1/2) total	1.0
Structure refinement	
R_{work}/R_{free} (%)	17.91/20.41
RMSD bonds (Å)	0.005
RMSD angles (deg)	0.886
Ramachandran angles g	
Favored	97.62
Allowed	2.38
Outliers	0.00
B-factors	
Average B-factor	25.4
Ligands	30.9
Solvent	31.7

Complementation of rufomycin production

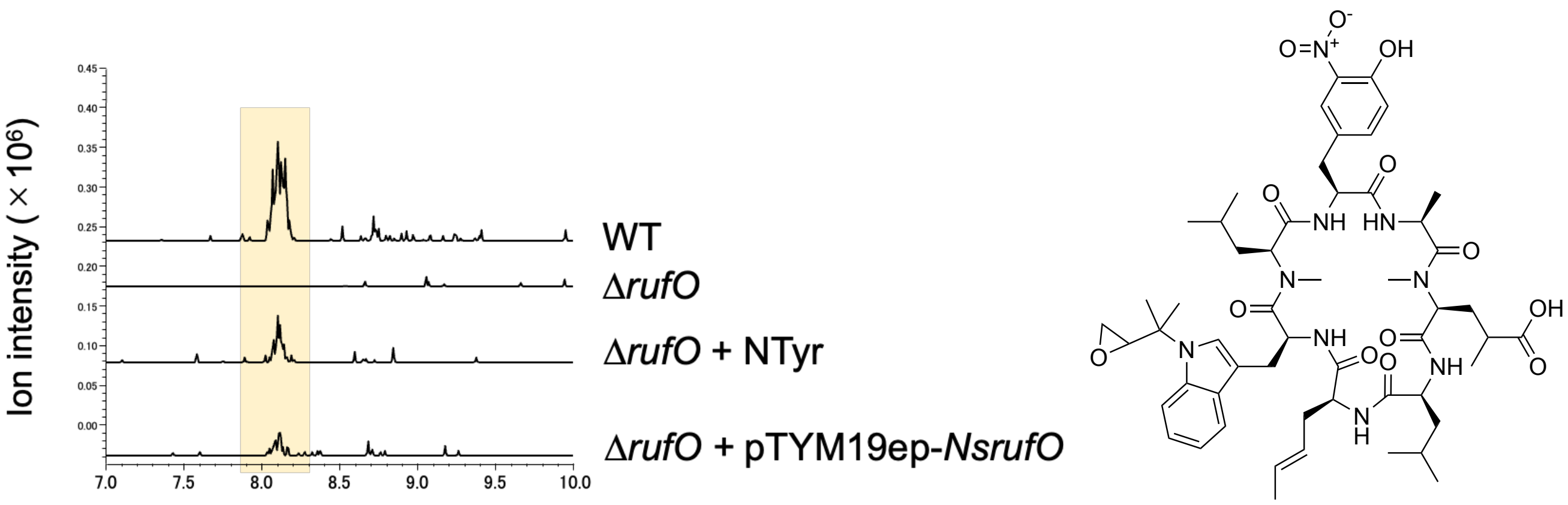

Figure S95. Complementation of rufomycin production in the *Streptomyces atratus* ATCC 14046 Δ*rufO* strain using *NsrufO*. Extracted ion chromatogram at *m/z* = 1059 (+) are shown. Production of rufomycin was restored by supplementation of nitrotyrosine (NTyr) or introduction of the *NsrufO* gene.

Table S8. Primers used in the production of NsRufO and complementation of the ΔRufO in this study.

Name	Sequence
NsP450-Nde-F	5'-AACATATGACGGCTTCCACGACGGCGC-3'
NsP450-Eco-R	5'-AAGAATTCTCATGGCCGCGTCACCGTGACC-3'
NsP450-pTYM-IF-F	5'-TAGGATTCCACATATGACGGCTTCCACGACGGCGCATGC-3'
NsP450-pTYM-IF-R	5'-TGCATGCCTGCAGGTTCATGGCCGCGTCACCGTGACC-3'
NsP450-pTYM-IF-F2	5'-ACCTGCAGGCATGCAAGCTTGGCG-3'
NsP450-pTYM-IF-R2	5'-CATATGTGGATCCTACCAACCGGC-3'

Coordinates

R

C	1.953333	0.658793	3.577079
S	2.785673	0.373145	1.970836
H	1.333811	1.560288	3.554942
H	2.732517	0.787969	4.336963
C	−7.297710	−1.257993	−0.736837
C	−5.971710	−0.542952	−0.869723
C	−5.906720	0.831332	−1.134013
C	−4.757117	−1.234569	−0.760396
C	−4.688300	1.488719	−1.287813
C	−3.526981	−0.598365	−0.910051
C	−3.479573	0.781913	−1.178199
O	−2.328292	1.465862	−1.336544
H	−8.098741	−0.566446	−0.448386
H	−7.255402	−2.050195	0.021101
H	−6.830939	1.402344	−1.220036
H	−4.769169	−2.303588	−0.551151
H	−4.647580	2.556090	−1.487179
H	−2.597760	−1.154159	−0.826970
H	−1.536389	0.872804	−1.204511
C	1.246235	−3.261063	0.912818
C	3.744509	−0.250249	−1.933552
C	1.150003	3.447481	−0.200583
C	−1.481846	0.411257	2.495429
C	2.164925	−2.758504	−0.000379
C	3.138305	−3.565607	−0.701861
C	3.834965	−2.722854	−1.511875
C	3.289681	−1.400995	−1.301423
C	3.246193	1.027574	−1.713079
C	3.744192	2.223239	−2.355061
C	3.019685	3.262465	−1.859002
C	2.077595	2.699914	−0.916280
C	0.216279	2.941548	0.695876
C	−0.762361	3.747724	1.390544
C	−1.513508	2.895487	2.138722
C	−0.989414	1.569023	1.906442
C	−0.962662	−0.863281	2.298739
C	−1.466176	−2.060875	2.932069
C	−0.688387	−3.088938	2.495328
C	0.285223	−2.520153	1.590230
N	2.276476	−1.449547	−0.382862
N	2.234544	1.343081	−0.848126
N	0.064681	1.623615	1.032957
N	0.101725	−1.167441	1.492766
Fe	1.184613	0.089462	0.342175
H	4.557517	−0.356622	−2.646270
H	1.143535	4.520962	−0.368303
H	−2.333563	0.509567	3.162139
H	1.269889	−4.330907	1.101234
H	−2.307890	−2.090884	3.613439
H	−2.353486	3.122121	2.784086
H	−0.856878	4.822439	1.291677
H	3.097020	4.316298	−2.098581
H	4.543259	2.243585	−3.086503
H	4.643182	−2.955236	−2.194991
O	−0.191942	−0.168675	−1.024290
O	0.322146	−0.337733	−2.343521
N	0.053163	−1.576704	−2.910665
O	−0.569443	−2.343189	−2.242344
H	−7.609128	−1.735172	−1.678125
H	1.325807	−0.191281	3.861154
H	3.252211	−4.636398	−0.582316
H	−0.758340	−4.141468	2.742850

0 imaginary frequencies
ZPE 0.460946
E −2177.776179
H −2177.279082
G −2177.386505

TS1

C	2.357970	0.982266	3.395364
S	2.975428	0.580143	1.718327
H	1.719961	1.871534	3.389349
H	3.226222	1.181954	4.033971
C	−7.292874	−1.371098	−0.586501
C	−5.975901	−0.653167	−0.779530
C	−5.927678	0.710383	−1.097666
C	−4.752854	−1.320103	−0.622476
C	−4.717134	1.383243	−1.245869
C	−3.530154	−0.668086	−0.765524
C	−3.499533	0.703055	−1.079337
O	−2.356618	1.399657	−1.243586
H	−8.104226	−0.874665	−1.132830
H	−7.594819	−1.409448	0.470958
H	−6.858917	1.259655	−1.234611
H	−4.751938	−2.383189	−0.384741
H	−4.689581	2.440244	−1.496332
H	−2.594460	−1.207742	−0.651508
H	−1.559794	0.825930	−1.064633
C	1.472853	−3.179410	1.087776
C	3.438073	−0.271601	−2.246183
C	0.937249	3.442045	−0.409828
C	−1.160132	0.498785	2.806520
C	2.233708	−2.702915	0.028352
C	3.144163	−3.516910	−0.745038
C	3.692293	−2.704360	−1.688746
C	3.119771	−1.392609	−1.490189
C	2.918417	0.999478	−2.038931
C	3.278350	2.168154	−2.809796
C	2.580153	3.211370	−2.285896
C	1.791597	2.679647	−1.196065
C	0.156250	2.966634	0.636748
C	−0.753899	3.780358	1.411754
C	−1.354745	2.951797	2.307674
C	−0.807604	1.632195	2.084534
C	−0.620336	−0.768665	2.619643
C	−0.980737	−1.936148	3.393199
C	−0.230040	−2.966710	2.917243
C	0.583930	−2.430177	1.848888
N	2.236163	−1.418283	−0.445653
N	2.014744	1.335707	−1.069717
N	0.113241	1.672025	1.072473
N	0.334060	−1.095655	1.695419
Fe	1.147317	0.112521	0.281409
H	4.156691	−0.395979	−3.051228
H	0.862743	4.500614	−0.642392
H	−1.913096	0.612963	3.581055
H	1.571264	−4.233076	1.333636
H	−1.715414	−1.945401	4.189384
H	−2.105272	3.189872	3.051670
H	−0.908908	4.842230	1.263685
H	2.582239	4.249921	−2.594378
H	3.976345	2.168898	−3.638409
H	4.417419	−2.950558	−2.455144
O	−0.244515	−0.202018	−0.706639
O	0.062114	−0.681854	−2.556364
N	−0.275390	−1.841958	−2.857848
O	−0.757626	−2.646760	−2.092597
H	−7.242269	−2.408611	−0.939570
H	−0.220266	−4.001058	3.239546
H	3.322117	−4.571965	−0.575105
H	1.793376	0.150277	3.827802

1 imaginary frequency
ZPE 0.458833
E −2177.752582
H −2177.257408
G −2177.366190

I1

C	2.369677	0.968943	3.431639
S	2.950091	0.867052	1.695870
H	1.567731	1.705601	3.547495
H	3.215148	1.279201	4.056806
C	−7.220159	−1.360059	−0.341038
C	−5.901534	−0.696301	−0.669157
C	−5.850409	0.574802	−1.256403
C	−4.679876	−1.317148	−0.374143
C	−4.638901	1.204192	−1.531682
C	−3.456409	−0.706604	−0.640295
C	−3.422032	0.572726	−1.224829
O	−2.278018	1.221379	−1.519715
H	−8.025128	−0.997110	−0.991853
H	−7.535696	−1.169101	0.695742
H	−6.780525	1.084711	−1.506465
H	−4.681016	−2.309905	0.074406
H	−4.609698	2.188466	−1.991106
H	−2.521384	−1.208885	−0.411452
H	−1.480534	0.688710	−1.233550
C	1.903634	−3.064057	0.968388
C	3.289994	0.184579	−2.339782
C	0.499703	3.512579	−0.208037
C	−1.139115	0.174368	2.888033
C	2.545413	−2.457480	−0.102505
C	3.504197	−3.119532	−0.958559
C	3.880831	−2.212196	−1.899947
C	3.158616	−0.992946	−1.615367
C	2.647080	1.380099	−2.044428
C	2.835937	2.613245	−2.775850
C	2.065783	3.552444	−2.163745
C	1.400360	2.891533	−1.062755
C	−0.186746	2.891980	0.827698
C	−1.164222	3.548410	1.666438
C	−1.641020	2.606333	2.524065
C	−0.947825	1.375440	2.216078
C	−0.443247	−1.002009	2.638013
C	−0.620424	−2.230510	3.378891
C	0.250012	−3.134284	2.851571
C	0.952121	−2.461029	1.782145
N	2.355474	−1.168979	−0.522716
N	1.773942	1.577303	−1.012676
N	−0.067680	1.578876	1.188893
N	0.520366	−1.168351	1.680653
Fe	1.085603	0.181749	0.283192
H	3.964459	0.175755	−3.191105
H	0.295539	4.565510	−0.379472
H	−1.878337	0.158888	3.683702
H	2.148848	−4.102248	1.173458
H	−1.322842	−2.363037	4.193028
H	−2.392888	2.712969	3.296577
H	−1.444777	4.591380	1.583567
H	1.940586	4.597428	−2.420392
H	3.480291	2.724263	−3.639668
H	4.581968	−2.335907	−2.716525
O	−0.169305	−0.246592	−0.734851
O	0.118585	−1.735914	−3.446083
N	−0.058453	−2.139597	−2.322818
O	−0.752056	−3.034483	−1.902029
H	−7.163880	−2.449194	−0.460054
H	0.410135	−4.166426	3.139272
H	3.829014	−4.146280	−0.840270
H	2.008029	0.001911	3.796395

0 imaginary frequencies
ZPE 0.459354
E −2177.765949
H −2177.268978
G −2177.383293

TS2

C	3.111310	−0.494232	−3.092203
S	3.409784	−0.359700	−1.288432
H	2.468009	−1.346992	−3.333521
H	4.077325	−0.640079	−3.589503
C	−6.944334	0.681913	−0.563622
C	−5.631738	0.171220	−0.015503
C	−5.554231	−1.154268	0.506008
C	−4.498102	0.944987	−0.036002
C	−4.344574	−1.691431	0.939089
C	−3.245898	0.495919	0.568225
C	−3.174599	−0.930247	0.917320
O	−2.016208	−1.488658	1.246405
H	−7.331741	0.032184	−1.360180
H	−6.838064	1.690754	−0.975764
H	−6.451624	−1.769327	0.525733
H	−4.522942	1.953790	−0.437612
H	−4.275034	−2.725144	1.265623
H	−2.326568	0.898342	0.140605
H	−1.231021	−0.850725	1.080848
C	−0.519022	−0.210183	−3.075904
C	1.687579	3.327267	−0.615516
C	3.042820	0.147685	2.768496
C	1.125825	−3.433643	0.139760
C	−0.047737	1.039399	−2.690747
C	−0.294056	2.267006	−3.413393
C	0.343581	3.257091	−2.730742
C	0.971821	2.636380	−1.586019
C	2.248356	2.765514	0.523628
C	2.948083	3.517996	1.542467
C	3.313786	2.627886	2.503267
C	2.844313	1.330602	2.067822
C	2.639283	−1.111521	2.341968
C	2.903256	−2.340757	3.056820
C	2.381016	−3.348726	2.307707
C	1.790802	−2.735000	1.138520
C	0.512137	−2.864846	−0.969333
C	−0.223718	−3.610418	−1.965228
C	−0.704060	−2.703700	−2.859019
C	−0.253765	−1.403739	−2.415022
N	0.727540	1.291453	−1.591731
N	2.206020	1.443512	0.865712
N	1.965472	−1.379828	1.184174
N	0.485565	−1.531090	−1.270453
Fe	1.279417	−0.034132	−0.164988
H	1.797521	4.399867	−0.747871
H	3.573344	0.209133	3.714371
H	1.059255	−4.512547	0.246362
H	−1.124827	−0.260980	−3.976162
H	−0.353062	−4.685939	−1.957550
H	2.378848	−4.412623	2.511822
H	3.424235	−2.402021	4.004730
H	3.852033	2.810455	3.425588
H	3.119123	4.587245	1.509876
H	0.388472	4.315133	−2.959302
O	−0.148398	0.187747	0.681666
N	−3.106248	1.412609	2.099184
O	−3.132366	2.628521	1.969041
O	−3.049149	0.801099	3.152032
H	−7.715955	0.721538	0.217559
H	2.649569	0.413106	−3.495443
H	−1.307320	−2.878312	−3.741794
H	−0.879240	2.340594	−4.322243

1 imaginary frequency
ZPE 0.459657
E −2177.741132
H −2177.245795
G −2177.353459

I2

C	3.094369	0.217598	3.127073
S	3.392542	0.280392	1.319346
H	2.429345	1.023032	3.456046
H	4.057315	0.335898	3.637489
C	−6.962818	−0.742619	0.407872
C	−5.643212	−0.192624	−0.083552
C	−5.567292	1.170231	−0.506241
C	−4.515595	−0.966807	−0.098983
C	−4.347136	1.742614	−0.875569
C	−3.226061	−0.477406	−0.650777
C	−3.177325	0.994311	−0.899542
O	−2.013230	1.579419	−1.158343
H	−7.353439	−0.160145	1.253317
H	−6.865841	−1.783597	0.733101
H	−6.467143	1.781007	−0.494991
H	−4.542691	−2.001938	0.228092
H	−4.277316	2.799801	−1.116291
H	−2.343192	−0.832900	−0.110095
H	−1.228184	0.932842	−1.030472
C	−0.521859	−0.235873	3.077544
C	1.802559	−3.342295	0.182661
C	3.018550	0.293604	−2.767074
C	0.989096	3.433511	0.304119
C	−0.005727	−1.407050	2.535193
C	−0.202436	−2.725815	3.094212
C	0.468227	−3.595143	2.289851
C	1.066898	−2.809262	1.234178
C	2.335425	−2.618418	−0.875540
C	3.055931	−3.207346	−1.983573
C	3.382368	−2.188403	−2.822889
C	2.868181	−0.976131	−2.223871
C	2.571631	1.471588	−2.181569
C	2.787739	2.791651	−2.731546
C	2.234304	3.674836	−1.857754
C	1.672629	2.894271	−0.777358
C	0.401958	2.704260	1.330699
C	−0.356649	3.287670	2.413809
C	−0.799865	2.256257	3.183057
C	−0.303802	1.041862	2.575865
N	0.772920	−1.486431	1.412947
N	2.242227	−1.265999	−1.045181
N	1.894798	1.563472	−0.998244
N	0.425706	1.342697	1.457580
Fe	1.267606	0.030682	0.167934
H	1.952579	−4.418158	0.175684
H	3.545453	0.373986	−3.713555
H	0.882903	4.513974	0.337763
H	−1.124847	−0.323910	3.976841
H	−0.525618	4.349685	2.544575
H	2.193236	4.755427	−1.922410
H	3.301091	2.993684	−3.663943
H	3.921094	−2.231020	−3.761868
H	3.266356	−4.264819	−2.088786
H	0.553351	−4.671375	2.380109
O	−0.156259	−0.134589	−0.698954
N	−2.995450	−1.271951	−2.056492
O	−2.872259	−2.487100	−1.955917
O	−3.015806	−0.641540	−3.103127
H	−7.726575	−0.708915	−0.381110
H	−0.779971	−2.937531	3.986119
H	−1.405543	2.293474	4.080545
H	2.657708	−0.738982	3.432694

0 imaginary frequencies
ZPE 0.460669
E −2177.741356
H −2177.244726
G −2177.353274

TS3

C	1.782086	0.503632	−3.650988
S	2.659703	0.505311	−2.040688
H	1.282783	−0.452451	−3.842288
H	2.518442	0.671080	−4.445968
C	−4.939404	2.394780	0.735456
C	−4.277414	1.035153	0.731296
C	−4.725318	0.032117	−0.165599
C	−3.230251	0.757936	1.576180
C	−4.086284	−1.217807	−0.217619
C	−2.483241	−0.508440	1.534960
C	−3.015098	−1.517833	0.600626
O	−2.315978	−2.677977	0.502831
H	−4.602701	2.997759	−0.118429
H	−4.697041	2.952817	1.645825
H	−5.571978	0.229428	−0.818723
H	−2.879786	1.493334	2.292492
H	−4.421909	−1.969229	−0.930531
H	−1.309708	−0.294175	1.231249
H	−2.332013	−2.980624	−0.423487
C	−1.604943	0.233113	−2.433216
C	0.865708	3.512634	0.130857
C	3.768962	0.042102	1.834767
C	1.334328	−3.236277	−0.751993
C	−1.182289	1.435068	−1.881777
C	−1.795114	2.718750	−2.143890
C	−1.097675	3.637893	−1.422006
C	−0.059215	2.916548	−0.719128
C	1.878357	2.852610	0.815052
C	2.823702	3.501671	1.700394
C	3.636385	2.525408	2.184713
C	3.188084	1.281340	1.593611
C	3.359624	−1.159479	1.268472
C	3.987822	−2.439810	1.523657
C	3.307746	−3.360372	0.790871
C	2.262988	−2.641728	0.089805
C	0.315027	−2.573651	−1.429549
C	−0.644484	−3.227850	−2.289254
C	−1.475367	−2.251748	−2.765556
C	−1.018509	−1.006104	−2.194535
N	−0.131613	1.583951	−1.016505
N	2.127059	1.511509	0.767209
N	2.324126	−1.311683	0.393857
N	0.063465	−1.230027	−1.386602
Fe	1.046243	0.128731	−0.256712
H	0.791447	4.587972	0.268184
H	4.613654	0.010493	2.517337
H	1.403900	−4.311674	−0.890401
H	−2.464649	0.260673	−3.096136
H	−0.654836	−4.289347	−2.508106
H	3.476253	−4.428284	0.719901
H	4.835292	−2.590386	2.181779
H	4.465767	2.616096	2.876027
H	2.842575	4.564450	1.910524
H	−1.254278	4.708258	−1.359471
O	0.097760	−0.106936	1.130936
N	−2.239969	−1.022862	2.966783
O	−1.833148	−0.199831	3.782257
O	−2.462101	−2.202854	3.216395
H	−6.031854	2.312034	0.668567
H	−2.643561	2.874814	−2.799486
H	−2.306568	−2.348748	−3.454224
H	1.032161	1.300390	−3.705300

1 imaginary frequency
ZPE 0.455876
E −2177.718518
H −2177.227214
G −2177.329798

TS4

C	3.476436	0.429275	2.768952
S	3.485435	0.563305	0.942473
H	2.775274	1.138079	3.220509
H	4.485177	0.659898	3.129822
C	−7.096551	−0.962073	0.478361
C	−5.746316	−0.402681	0.091423
C	−5.609110	1.003180	−0.129714
C	−4.650833	−1.214767	−0.018139
C	−4.367995	1.571408	−0.404562
C	−3.330729	−0.705845	−0.470830
C	−3.207836	0.793239	−0.525791
O	−2.034390	1.327250	−0.683321
H	−7.486510	−0.486477	1.389005
H	−7.046041	−2.041184	0.658685
H	−6.487314	1.639920	−0.041775
H	−4.727888	−2.284393	0.157957
H	−4.256295	2.648201	−0.501326
H	−2.477961	−1.158299	0.047429
H	−1.097936	0.539843	−0.631384
C	−0.004738	−0.532940	3.241515
C	2.261228	−3.194831	−0.106893
C	2.468476	0.648200	−3.036978
C	0.606758	3.398230	0.481499
C	0.578703	−1.602791	2.574058
C	0.644901	−2.951772	3.086928
C	1.303911	−3.692779	2.154238
C	1.630460	−2.799825	1.066207
C	2.511815	−2.376139	−1.199426
C	3.108780	−2.829630	−2.436092
C	3.146418	−1.755430	−3.269395
C	2.581958	−0.642993	−2.538483
C	1.979925	1.736564	−2.326380
C	1.935492	3.089315	−2.833700
C	1.435741	3.865949	−1.835431
C	1.163350	2.987177	−0.720875
C	0.276654	2.562011	1.539662
C	−0.380870	3.000806	2.748189
C	−0.577528	1.893067	3.513838
C	−0.028102	0.776927	2.779787
N	1.191291	−1.536622	1.351973
N	2.209143	−1.046155	−1.287489
N	1.506655	1.703077	−1.044054
N	0.488946	1.210893	1.587909
Fe	1.291809	0.072108	0.131506
H	2.551806	−4.238211	−0.188776
H	2.810467	0.825744	−4.052332
H	0.372657	4.453201	0.588433
H	−0.456113	−0.731048	4.209336
H	−0.661838	4.025736	2.957387
H	1.249554	4.932967	−1.836597
H	2.251908	3.384079	−3.826857
H	3.517263	−1.699572	−4.285656
H	3.438278	−3.844201	−2.624552
H	1.543997	−4.748908	2.176456
O	−0.246657	−0.280250	−0.532074
N	−3.089576	−1.324275	−1.948430
O	−2.880654	−2.533768	−1.985862
O	−3.215386	−0.597283	−2.924323
H	−7.841173	−0.790751	−0.311551
H	3.211738	−0.580911	3.096514
H	−1.048563	1.817841	4.486413
H	0.234619	−3.270352	4.037609

1 imaginary frequency
ZPE 0.455894
E −2177.740016
H −2177.248528
G −2177.355375

P1

C	−4.078260	−1.471397	1.317826
S	−2.782903	−0.348676	1.967471
H	−4.571669	−1.052521	0.434952
H	−4.831053	−1.612903	2.101880
C	5.019248	−1.320786	1.415826
C	4.400485	−0.850904	0.122624
C	4.625735	−1.635676	−1.083867
C	3.652247	0.269930	0.040752
C	4.104983	−1.306812	−2.289231
C	2.958114	0.675790	−1.209746
C	3.259427	−0.121161	−2.472414
O	2.764774	0.160014	−3.558282
H	4.662891	−2.324744	1.683212
H	4.763438	−0.650378	2.240784
H	5.255012	−2.521742	−0.997435
H	3.463685	0.888352	0.912395
H	4.301781	−1.893593	−3.182257
H	1.814741	0.521320	−1.027628
H	−0.042100	−0.113905	−1.771030
C	0.645855	−2.534319	1.808168
C	0.329846	2.257380	2.401429
C	−2.993033	2.414719	−1.108174
C	−2.532322	−2.349854	−1.841873
C	0.835196	−1.243304	2.290855
C	1.731288	−0.902587	3.375773
C	1.656149	0.447980	3.534559
C	0.708055	0.927110	2.551629
C	−0.612825	2.720458	1.492504
C	−1.012982	4.105813	1.359992
C	−1.956069	4.145657	0.382167
C	−2.127136	2.784449	−0.085188
C	−3.159733	1.128933	−1.612622
C	−4.047761	0.790829	−2.703091
C	−3.912873	−0.546755	−2.920097
C	−2.946361	−1.026938	−1.958044
C	−1.623794	−2.822064	−0.898954
C	−1.216319	−4.203521	−0.768275
C	−0.331168	−4.252276	0.265148
C	−0.193274	−2.900425	0.761263
N	0.228518	−0.116390	1.812170
N	−1.305943	1.943354	0.608991
N	−2.505562	0.008757	−1.176055
N	−0.991479	−2.053682	0.041002
Fe	−1.148591	−0.054175	0.326036
H	0.802102	2.989449	3.050325
H	−3.582527	3.204489	−1.566240
H	−2.960740	−3.072155	−2.531377
H	1.209835	−3.329431	2.289011
H	−1.571502	−5.014527	−1.393074
H	−4.411309	−1.165358	−3.657054
H	−4.679214	1.498907	−3.226601
H	−2.490286	5.003801	−0.007962
H	−0.607844	4.923618	1.943658
H	2.175676	1.075265	4.249323
O	0.231092	0.267013	−0.920982
N	3.082183	2.179775	−1.389235
O	2.766035	2.865661	−0.418112
O	3.496626	2.629299	−2.450705
H	6.114540	−1.377851	1.341712
H	−3.665121	−2.450250	1.053499
H	2.327938	−1.616103	3.931804
H	0.193008	−5.112575	0.664535

0 imaginary frequencies
ZPE 0.460264
E −2177.797333
H −2177.300942
G −2177.408837

P2 (Tyr-NO2)

C	3.249096	−1.119712	0.000037
C	1.964969	−0.325092	−0.000011
C	1.988570	1.087767	−0.000070
C	0.726407	−0.945123	0.000033
C	0.829266	1.841778	−0.000026
C	−0.461082	−0.192716	0.000015
C	−0.433931	1.222135	0.000013
O	−1.518607	2.005640	0.000042
H	3.860313	−0.892489	−0.882158
H	3.050463	−2.195446	−0.000285
H	2.947891	1.600470	−0.000092
H	0.639491	−2.025279	0.000065
H	0.856786	2.926421	−0.000040
H	−2.301140	1.401270	0.000015
N	−1.717164	−0.908444	−0.000018
O	−2.780105	−0.243070	0.000018
O	−1.710456	−2.134708	−0.000053
H	3.859930	−0.892958	0.882622

0 imaginary frequencies
ZPE 0.135813
E −551.489444
H −551.343347
G −551.388719

TS5

C	1.923443	1.265263	3.514435
S	2.679627	1.130076	1.854067
H	1.035496	1.904751	3.490567
H	2.659828	1.711845	4.191589
C	−7.228001	−1.461898	−0.559367
C	−5.873302	−0.842448	−0.810444
C	−5.684269	0.548870	−0.774618
C	−4.739949	−1.639141	−1.057097
C	−4.434012	1.123032	−0.962640
C	−3.484149	−1.089270	−1.266131
C	−3.275913	0.327362	−1.229886
O	−2.126383	0.877923	−1.454749
H	−8.037171	−0.744499	−0.744790
H	−7.344476	−1.812999	0.478253
H	−6.544309	1.193960	−0.588734
H	−4.854234	−2.723316	−1.090983
H	−4.302390	2.201671	−0.931889
H	−2.624303	−1.726166	−1.454908
H	−0.888863	0.124325	−1.068233
C	2.090348	−2.899992	1.310251
C	3.478822	0.267693	−2.069560
C	0.228623	3.442901	−0.403952
C	−1.440135	0.113276	2.684659
C	2.763714	−2.305408	0.251603
C	3.845904	−2.926731	−0.479024
C	4.224124	−2.043629	−1.442771
C	3.378775	−0.880249	−1.294335
C	2.718040	1.416758	−1.897897
C	2.871104	2.619674	−2.683504
C	1.972974	3.520863	−2.201129
C	1.263613	2.866005	−1.126656
C	−0.514476	2.802783	0.577306
C	−1.643352	3.394981	1.256361
C	−2.130754	2.450657	2.104509
C	−1.290230	1.284802	1.955431
C	−0.602036	−0.991646	2.599410
C	−0.743454	−2.190371	3.391886
C	0.266781	−3.025288	3.022322
C	1.017717	−2.342043	1.994796
N	2.498645	−1.067285	−0.265236
N	1.739643	1.594679	−0.958778
N	−0.312583	1.526279	1.027029
N	0.481437	−1.103202	1.770686
Fe	1.062143	0.226428	0.363969
H	4.224016	0.275555	−2.859629
H	−0.051883	4.460693	−0.658268
H	−2.268819	0.061648	3.384495
H	2.405048	−3.896922	1.605633
H	−1.523402	−2.355132	4.125381
H	−2.981690	2.513403	2.771427
H	−2.010996	4.397887	1.077173
H	1.783855	4.533622	−2.535596
H	3.578538	2.737718	−3.495408
H	5.001840	−2.148920	−2.189655
O	−0.060016	−0.446710	−0.791903
O	0.764404	−1.534226	−3.646723
N	0.430568	−2.150740	−2.667411
O	−0.369912	−3.038907	−2.508910
H	−7.403660	−2.332250	−1.205615
H	0.487974	−4.021304	3.386670
H	4.245809	−3.911495	−0.268967
H	1.635635	0.283964	3.903726

1 imaginary frequency
ZPE 0.453856
E −2177.750288
H −2177.259173
G −2177.372638

I3

C	3.046836	2.475466	1.985764
S	2.452793	0.755671	2.208563
H	3.633408	2.585203	1.068063
H	3.686170	2.728195	2.839369
C	−3.021204	−2.581477	−0.430760
C	−3.936757	−1.420582	−0.695883
C	−5.340071	−1.601690	−0.788556
C	−3.402441	−0.110454	−0.822073
C	−6.187335	−0.536184	−0.985952
C	−4.231429	0.970422	−1.015815
C	−5.673077	0.817749	−1.106673
O	−6.443355	1.798606	−1.283396
H	−3.471969	−3.531170	−0.741167
H	−2.801163	−2.651768	0.644608
H	−5.743475	−2.609189	−0.699080
H	−2.319279	0.007400	−0.768254
H	−7.264133	−0.663472	−1.059559
H	−3.833389	1.977597	−1.104310
H	−0.034597	0.344633	−1.650502
C	0.953322	−3.002231	1.651989
C	3.787585	−0.624464	−1.464529
C	1.101349	3.371236	−0.944758
C	−1.607897	1.046243	2.327269
C	1.957346	−2.703842	0.739425
C	2.960097	−3.644525	0.287122
C	3.763404	−2.970765	−0.578421
C	3.247334	−1.619672	−0.657874
C	3.301911	0.672555	−1.594445
C	3.871460	1.671115	−2.471810
C	3.111666	2.792804	−2.336740
C	2.081412	2.481605	−1.371642
C	0.126195	3.088887	0.007616
C	−0.882293	4.023380	0.456290
C	−1.634646	3.369279	1.383856
C	−1.088789	2.034406	1.497997
C	−1.144703	−0.261393	2.428993
C	−1.735628	−1.269237	3.284552
C	−1.023797	−2.413216	3.088311
C	0.001668	−2.099897	2.115689
N	2.156084	−1.485682	0.153667
N	2.218598	1.188737	−0.935689
N	−0.019166	1.891982	0.655624
N	−0.095530	−0.792954	1.733302
Fe	1.072698	0.201638	0.411948
H	4.658367	−0.887695	−2.058950
H	1.104015	4.366952	−1.379596
H	−2.464475	1.316161	2.938817
H	0.915412	−4.015738	2.041730
H	−2.582439	−1.103820	3.939930
H	−2.487478	3.736809	1.941761
H	−0.986998	5.040123	0.096833
H	3.223037	3.748481	−2.835074
H	4.735345	1.513751	−3.106597
H	4.623578	−3.335961	−1.126815
O	−0.120662	−0.336530	−0.962295
O	0.395579	−1.282330	−3.702359
N	0.452286	−1.901674	−2.659495
O	0.026202	−3.005352	−2.398284
H	−2.056843	−2.454303	−0.930505
H	−1.160970	−3.382929	3.552041
H	3.021387	−4.679735	0.600855
H	2.214527	3.186031	1.953914

0 imaginary frequencies
ZPE 0.457706
E −2177.777037
H −2177.281604
G −2177.394052

TSNR

C	2.377575	0.702139	3.493597
S	2.978112	0.478028	1.776654
H	1.725442	1.576409	3.586964
H	3.249989	0.851049	4.140543
C	−7.264178	−1.368261	−0.589228
C	−5.949609	−0.646399	−0.783789
C	−5.906527	0.706928	−1.144101
C	−4.724362	−1.297856	−0.585763
C	−4.699199	1.384799	−1.294158
C	−3.504859	−0.640103	−0.729873
C	−3.479044	0.720489	−1.086651
O	−2.338976	1.420776	−1.254426
H	−8.066490	−0.906688	−1.177847
H	−7.591947	−1.360053	0.461189
H	−6.839588	1.243817	−1.313609
H	−4.719429	−2.352677	−0.313583
H	−4.675930	2.433333	−1.578243
H	−2.566162	−1.165061	−0.580126
H	−1.539638	0.854714	−1.056807
C	1.800383	−3.240437	0.548573
C	3.308088	0.377116	−2.295169
C	0.569732	3.463895	0.229490
C	−1.095719	−0.185633	2.935203
C	2.463023	−2.515480	−0.432415
C	3.411906	−3.085443	−1.360805
C	3.830321	−2.071970	−2.167046
C	3.140394	−0.880634	−1.730144
C	2.680795	1.536098	−1.858307
C	2.893445	2.847121	−2.430473
C	2.132403	3.714819	−1.711680
C	1.451157	2.933676	−0.702307
C	−0.116357	2.738296	1.196238
C	−1.066343	3.309678	2.124420
C	−1.547589	2.281908	2.874666
C	−0.886405	1.083189	2.409834
C	−0.442386	−1.341815	2.523961
C	−0.654953	−2.651482	3.099071
C	0.168553	−3.508008	2.435228
C	0.879452	−2.722558	1.451155
N	2.313949	−1.175956	−0.679470
N	1.802602	1.616647	−0.814818
N	−0.018117	1.390581	1.397664
N	0.497695	−1.413715	1.533182
Fe	1.100064	0.089276	0.311634
H	3.991224	0.462017	−3.135192
H	0.385199	4.533462	0.186972
H	−1.821424	−0.280360	3.737914
H	2.012072	−4.304280	0.608254
H	−1.348279	−2.866252	3.903426
H	−2.284583	2.310616	3.668091
H	−1.326950	4.360113	2.169908
H	2.024418	4.785368	−1.837594
H	3.544820	3.053631	−3.271116
H	4.538913	−2.107408	−2.985745
O	−0.233764	−0.164647	−0.693600
O	0.201340	−0.610327	−3.569028
N	−0.106688	−1.258501	−2.597613
O	−0.581138	−2.366598	−2.518774
H	−7.194856	−2.420465	−0.891819
H	0.292057	−4.574783	2.578503
H	3.703511	−4.128630	−1.379434
H	1.832217	−0.178945	3.847266

1 imaginary frequency
ZPE 0.459331
E −2177.766407
H −2177.270443
G −2177.381416

P3

```
C     -1.785475    -1.041044     3.496658
S     -2.664292    -0.215003     2.120474
H     -1.460856    -2.048178     3.220644
H     -2.490123    -1.114738     4.332816
C      7.491299     1.271809    -0.559489
C      6.167898     0.572720    -0.775768
C      6.062876    -0.823223    -0.725659
C      4.991618     1.297992    -1.012244
C      4.840255    -1.469834    -0.892328
C      3.759268     0.672432    -1.183326
C      3.669716    -0.728871    -1.119681
O      2.514142    -1.407434    -1.275402
H      8.335824     0.609927    -0.786647
H      7.618099     1.609202     0.480088
H      6.958209    -1.419815    -0.552809
H      5.038591     2.384770    -1.070516
H      4.768311    -2.553377    -0.854509
H      2.864467     1.246990    -1.397597
H      1.743665    -0.793589    -1.354300
C     -0.219458     3.020902     1.471201
C     -3.861140     1.468332    -1.307351
C     -2.246019    -3.063708    -0.812569
C      1.403739    -1.512494     1.960796
C     -1.348968     2.989512     0.663042
C     -2.120631     4.157803     0.301804
C     -3.146677     3.721980    -0.477881
C     -2.997876     2.290244    -0.597069
C     -3.725852     0.092498    -1.428164
C     -4.628828    -0.754474    -2.172277
C     -4.172074    -2.028518    -2.034445
C     -2.992911    -1.958468    -1.200607
C     -1.120216    -3.034114     0.000787
C     -0.356722    -4.203128     0.372833
C      0.672995    -3.768751     1.149164
C      0.537945    -2.335110     1.252385
C      1.278293    -0.133719     2.068000
C      2.182097     0.715334     2.808015
C      1.718555     1.987999     2.675661
C      0.533506     1.917031     1.851962
N     -1.897118     1.869621     0.099293
N     -2.738470    -0.659960    -0.852342
N     -0.561979    -1.911489     0.552908
N      0.282449     0.617520     1.498590
Fe    -1.247600    -0.025258     0.350263
H     -4.699697     1.937642    -1.813795
H     -2.569405    -4.034773    -1.177139
H      2.248635    -1.980265     2.457109
H      0.103751     3.991894     1.836151
H      3.058291     0.366094     3.340323
H      1.467084    -4.346780     1.605811
H     -0.587801    -5.214457     0.060412
H     -4.579447    -2.942436    -2.449942
H     -5.489898    -0.400440    -2.726251
H     -3.935743     4.300207    -0.943368
O      0.126847     0.102536    -1.214861
O     -1.085995     1.144719    -2.742783
N     -0.001103     0.687192    -2.389602
O      1.025960     0.733070    -3.087282
H      7.587192     2.160863    -1.195265
H      2.133742     2.903438     3.079823
H     -1.889607     5.169361     0.613593
H     -0.914395    -0.464884     3.820920
0 imaginary frequencies
ZPE 0.463504
E   -2177.834099
H   -2177.334620
G   -2177.442720
```

Additional Coordinates relating to Figure S6

cis-trans ferric peroxynitrite Isomerisation

```
C      1.919698     0.500046     3.630548
S      2.769889     0.310916     2.018822
H      1.280568     1.388163     3.647018
H      2.687701     0.608153     4.405142
C     -7.269605    -1.203158    -0.609888
C     -5.940717    -0.515920    -0.830819
C     -5.869873     0.829346    -1.216887
C     -4.728698    -1.191086    -0.631323
C     -4.649177     1.476461    -1.391066
C     -3.496609    -0.563334    -0.798967
C     -3.441166     0.789192    -1.182813
O     -2.289096     1.460170    -1.366580
H     -8.064234    -0.743278    -1.210255
H     -7.593924    -1.155468     0.440640
H     -6.792148     1.384270    -1.387748
H     -4.744926    -2.239749    -0.336442
H     -4.604849     2.518719    -1.695008
H     -2.568998    -1.104969    -0.643967
H     -1.496142     0.870264    -1.210031
C     -1.503302     0.217039     2.506581
C      1.329768    -3.302705     0.766945
C      3.752130    -0.090158    -1.919807
C      1.066537     3.451846    -0.012239
C     -0.950537    -1.033253     2.252149
C     -1.422474    -2.271735     2.828477
C     -0.615399    -3.257718     2.348428
C      0.344386    -2.621844     1.473638
C      2.238150    -2.731266    -0.115775
C      3.232218    -3.477474    -0.855406
C      3.906098    -2.577640    -1.621738
C      3.326503    -1.282167    -1.346130
C      3.223719     1.162695    -1.636301
C      3.695541     2.400969    -2.215442
C      2.947285     3.396637    -1.668534
C      2.016695     2.764522    -0.758197
C      0.138472     2.877811     0.848526
C     -0.865261     3.622722     1.575570
C     -1.598982     2.714571     2.273494
C     -1.038745     1.415171     1.978284
N      0.122396    -1.271868     1.434977
N      2.317367    -1.402137    -0.431854
N      2.205900     1.410708    -0.757995
N      0.018274     1.541173     1.116136
Fe     1.180704     0.070737     0.351168
H      1.384655    -4.379038     0.906526
H      4.569099    -0.140691    -2.634382
H      1.034258     4.532004    -0.125450
H     -2.359383     0.261005     3.173589
H     -0.986463     4.698406     1.531284
H      3.000175     4.462809    -1.854176
H      4.495045     2.476910    -2.942825
H      4.721106    -2.754558    -2.313517
H      3.375073    -4.549445    -0.787896
H     -0.655517    -4.321562     2.550489
O     -0.152955    -0.142466    -1.016820
O      0.360500    -0.180493    -2.370428
N      0.209092    -1.648635    -2.721939
O     -0.876049    -1.876948    -3.104354
H     -7.223744    -2.265495    -0.879821
H     -2.448225     2.886664     2.923623
H     -2.263085    -2.355724     3.506791
H      1.307114    -0.375400     3.867233
1 imaginary frequency
ZPE 0.459140
E   -2177.746001
H   -2177.250868
G   -2177.357771
```

trans-peroxynitrite

C	1.984080	1.207276	3.468753
S	2.785826	0.618625	1.930774
H	1.405558	2.119948	3.297053
H	2.775812	1.426395	4.194287
C	−7.288023	−1.443122	−0.603607
C	−5.974033	−0.723994	−0.811865
C	−5.929796	0.587811	−1.302515
C	−4.749448	−1.352746	−0.547833
C	−4.721862	1.243630	−1.526987
C	−3.529421	−0.716111	−0.764469
C	−3.501856	0.599518	−1.261949
O	−2.361274	1.278438	−1.496198
H	−8.106721	−0.737472	−0.416357
H	−7.240709	−2.129733	0.250670
H	−6.862727	1.110115	−1.513281
H	−4.745561	−2.370351	−0.159039
H	−4.697869	2.262565	−1.903603
H	−2.590936	−1.219784	−0.552319
H	−1.562054	0.725544	−1.265302
C	1.015403	−3.052100	1.544027
C	3.670480	−0.766134	−1.792682
C	1.324222	3.338149	−0.765098
C	−1.467280	1.008748	2.428298
C	1.955706	−2.780568	0.557911
C	2.870901	−3.760211	0.015822
C	3.611732	−3.122585	−0.930677
C	3.152079	−1.752806	−0.963148
C	3.255374	0.559609	−1.807184
C	3.820627	1.585893	−2.653855
C	3.166557	2.740842	−2.355602
C	2.199631	2.417837	−1.329149
C	0.367401	3.061391	0.204167
C	−0.554520	4.038880	0.737945
C	−1.350150	3.384183	1.626090
C	−0.910043	2.007814	1.639844
C	−1.029201	−0.309948	2.467698
C	−1.598580	−1.338743	3.307834
C	−0.889647	−2.475522	3.066658
C	0.107928	−2.142399	2.074432
N	2.145377	−1.571261	−0.054016
N	2.273496	1.088769	−1.015617
N	0.137720	1.838089	0.773709
N	0.006458	−0.821201	1.731417
Fe	1.158183	0.137970	0.375713
H	4.467096	−1.050703	−2.474164
H	1.382369	4.361804	−1.124655
H	−2.304631	1.279282	3.064929
H	0.976259	−4.069233	1.924355
H	−2.433407	−1.192113	3.982505
H	−2.168890	3.775560	2.217650
H	−0.583386	5.081863	0.446275
H	3.306404	3.726931	−2.782161
H	4.612027	1.423313	−3.375674
H	4.396868	−3.524683	−1.559587
O	−0.234906	−0.284060	−0.920635
O	0.246860	−0.676322	−2.206372
N	−0.037868	−2.027890	−2.393351
O	0.339276	−2.373678	−3.475307
H	−7.576697	−2.043794	−1.479166
H	1.321737	0.446992	3.893558
H	−1.020911	−3.458748	3.502629
H	2.918922	−4.797059	0.326441

0 imaginary frequencies
ZPE 0.460772
E −2177.771763
H −2177.274697
G −2177.382963

TS *trans*-peroxynitrite O-O cleavage

C	2.163818	1.732884	3.228974
S	2.866010	1.081112	1.668580
H	1.448884	2.540566	3.044064
H	2.992278	2.132671	3.825224
C	−7.233064	−1.514788	−0.174654
C	−5.921089	−0.876070	−0.571905
C	−5.880281	0.364092	−1.222151
C	−4.694977	−1.491144	−0.282571
C	−4.673582	0.970654	−1.563347
C	−3.476517	−0.903522	−0.614511
C	−3.453522	0.345164	−1.261098
O	−2.313463	0.971858	−1.620025
H	−7.498737	−1.302942	0.871929
H	−7.198261	−2.606434	−0.278315
H	−6.814410	0.867567	−1.469900
H	−4.688867	−2.461207	0.212926
H	−4.651856	1.931015	−2.071119
H	−2.537440	−1.401357	−0.391880
H	−1.517398	0.460621	−1.314114
C	−1.322297	1.148402	2.536583
C	1.443228	−2.777830	1.941203
C	3.510547	−0.762248	−1.941803
C	0.931139	3.262691	−1.185308
C	−0.756351	−0.109047	2.704012
C	−1.144843	−1.050881	3.728289
C	−0.355078	−2.148415	3.571141
C	0.510569	−1.880289	2.445778
C	2.243650	−2.563656	0.828967
C	3.184444	−3.530718	0.311574
C	3.756518	−2.972072	−0.788225
C	3.170516	−1.660889	−0.941229
C	2.982520	0.514429	−2.071088
C	3.373231	1.459855	−3.090277
C	2.657288	2.594576	−2.867307
C	1.824309	2.342247	−1.714186
C	0.096501	3.040356	−0.098069
C	−0.862869	3.996407	0.404511
C	−1.508908	3.396103	1.440116
C	−0.939076	2.075289	1.576188
N	0.255523	−0.634415	1.946030
N	2.257264	−1.436445	0.051642
N	2.043092	1.072667	−1.249221
N	0.039112	1.889396	0.636502
Fe	1.100605	0.190524	0.292195
H	1.539820	−3.734016	2.447224
H	4.252310	−1.076405	−2.669749
H	0.864733	4.229669	−1.675178
H	−2.121234	1.432200	3.215154
H	−1.016921	4.986852	−0.005897
H	2.671442	3.524314	−3.422878
H	4.101477	1.260135	−3.866794
H	4.503877	−3.392069	−1.450114
H	3.360963	−4.508041	0.743877
H	−0.350620	−3.066458	4.146184
O	−0.175666	−0.399385	−0.628470
O	0.204737	−1.304624	−2.421505
N	−0.262310	−2.496293	−2.274627
O	−0.124724	−3.212140	−3.263727
H	−8.060467	−1.149659	−0.795150
H	1.665831	0.949284	3.808222
H	−2.303866	3.789907	2.061513
H	−1.924181	−0.877126	4.460321

1 imaginary frequency
ZPE 0.458272
E −2177.737586
H −2177.242645
G −2177.353967

Additional Coordinates for Figures S7 & S8

·NO2

```
N      0.000000    -0.000000     0.328049
O      0.000000     1.106833    -0.143521
O     -0.000000    -1.106833    -0.143521
0 imaginary frequencies
ZPE 0.008824
E   -205.141927
H   -205.129223
G   -205.156489
```

Tyr·

```
C      2.818025    -0.000276     0.012008
H      3.227231    -0.806675    -0.606777
H      3.192573    -0.159794     1.033292
C      1.313384     0.003617    -0.012172
C      0.592666    -1.217359    -0.010379
C      0.590581     1.220863    -0.010297
H      1.148329    -2.152772    -0.017010
C     -0.782333    -1.235429    -0.001790
C     -0.785991     1.235146    -0.001814
H      1.143488     2.157675    -0.016633
H     -1.343549    -2.165029    -0.002087
C     -1.548071    -0.000828     0.003542
H     -1.349342     2.163432    -0.002130
O     -2.804862    -0.002595     0.009841
H      3.230587     0.949528    -0.341959
0 imaginary frequencies
ZPE 0.119346
E   -346.268648
H   -346.141188
G   -346.182737
```

Tyr· ·NO2

```
C      4.113262    -0.802481     0.084595
C      2.702751    -0.284259     0.011412
C      2.446927     1.109657    -0.006676
C      1.604570    -1.178993    -0.010923
C      1.162135     1.599215    -0.039062
C      0.307450    -0.721409    -0.044406
C      0.020897     0.701898    -0.059207
O     -1.157511     1.143256    -0.091996
H      4.817397    -0.125807    -0.410768
H      4.203315    -1.792673    -0.373633
H      3.290010     1.796996     0.004715
H      1.801373    -2.248812    -0.002962
H      0.954012     2.664724    -0.054834
H     -0.539727    -1.399511    -0.064375
N     -3.456182    -0.348264     0.301376
O     -3.179987    -1.400645    -0.220198
O     -4.277559     0.494854     0.068134
H      4.439402    -0.898566     1.130318
0 imaginary frequencies
ZPE 0.129294
E   -551.416384
H   -551.274098
G   -551.330209
```

Tyr· -- ·NO2

```
C      3.136750     0.724005     0.062467
C      1.806985     0.025356     0.113931
C      1.390684    -0.778908    -0.995574
C      0.957080     0.152672     1.217751
C      0.197448    -1.444485    -0.994647
C     -0.291464    -0.455527     1.227562
C     -0.703367    -1.368249     0.154981
O     -1.753614    -2.028374     0.224478
H      3.946526     0.019144    -0.164531
H      3.369948     1.223287     1.006853
H      2.057616    -0.860935    -1.851259
H      1.263168     0.773796     2.055725
H     -0.110926    -2.076371    -1.822014
H     -0.960126    -0.376686     2.079251
N     -1.477923     1.254934    -0.153562
O     -0.732838     2.102678    -0.584536
O     -2.679755     1.163393    -0.166972
H      3.144208     1.482470    -0.731668
1 imaginary frequency
ZPE 0.129632
E   -551.418546
H   -551.277196
G   -551.327190
```

Tyr-NO2 (keto)

```
C      3.082160    -1.202997     0.084106
C      1.808781    -0.410936    -0.058796
C      1.840785     1.008089     0.322139
C      0.665899    -0.951918    -0.518273
C      0.775585     1.831382     0.222829
C     -0.592754    -0.147984    -0.662488
C     -0.507430     1.348322    -0.292907
O     -1.479637     2.064433    -0.461661
H      3.886264    -0.769280    -0.524198
H      2.945579    -2.243419    -0.222795
H      2.783651     1.396812     0.702446
H      0.607117    -1.999946    -0.800522
H      0.821555     2.878148     0.506267
H     -1.001647    -0.230576    -1.673505
N     -1.662343    -0.823755     0.214021
O     -2.244183    -1.776481    -0.289322
O     -1.810515    -0.402023     1.352179
H      3.430413    -1.196636     1.124939
0 imaginary frequencies
ZPE 0.134413
E   -551.442799
H   -551.297496
G   -551.345039
```

Tyr

C	−2.892277	0.016132	0.000248
H	−3.286477	1.037526	−0.005950
H	−3.298454	−0.489631	0.885546
C	−1.380675	0.010730	−0.000203
C	−0.645766	1.199624	−0.000186
C	−0.663404	−1.196367	−0.000183
H	−1.169342	2.153032	−0.000323
C	0.751089	1.195316	−0.000030
C	0.727083	−1.220804	−0.000027
H	−1.206348	−2.139375	−0.000334
H	1.299481	2.136156	−0.000090
C	1.442223	−0.017711	0.000070
H	1.275262	−2.157888	−0.000076
O	2.810169	−0.095502	0.000156
H	3.173872	0.803390	0.000180
H	−3.298983	−0.500711	−0.878321

0 imaginary frequencies
ZPE 0.132279
E −346.911477
H −346.770820
G −346.812460

Tyr ·NO2

C	−4.138819	−1.118664	−0.296503
C	−2.798868	−0.444694	−0.108326
C	−2.623723	0.913322	−0.419018
C	−1.690803	−1.143882	0.378609
C	−1.398181	1.549162	−0.252518
C	−0.451711	−0.522997	0.552426
C	−0.300902	0.829771	0.236432
O	0.882449	1.500195	0.381336
H	−4.923748	−0.632002	0.296409
H	−4.101133	−2.170239	0.006166
H	−3.466940	1.486391	−0.799619
H	−1.791373	−2.197436	0.630405
H	−1.270535	2.599974	−0.494102
H	0.395013	−1.090574	0.933482
H	1.554539	0.889687	0.725976
N	3.800323	−0.264061	−0.372172
O	4.790331	−0.735429	−0.852116
O	3.312752	−0.304948	0.733804
H	−4.464298	−1.088031	−1.344318

0 imaginary frequencies
ZPE 0.142229
E −552.057875
H −551.902326
G −551.961566

Tyr -- ·NO2

C	3.267551	−0.930654	−0.015667
C	1.941699	−0.212232	−0.081894
C	1.810655	1.073320	0.522465
C	0.863126	−0.757264	−0.734201
C	0.611904	1.787211	0.466362
C	−0.427826	−0.091267	−0.771563
C	−0.491850	1.247149	−0.173245
O	−1.649683	1.939795	−0.240117
H	4.074912	−0.317157	−0.434154
H	3.239545	−1.875807	−0.565470
H	2.664733	1.505932	1.036228
H	0.949591	−1.718878	−1.230723
H	0.519124	2.769514	0.918740
H	−0.991439	−0.183548	−1.704491
H	−2.378556	1.289109	−0.331195
N	−1.630956	−1.052826	0.155231
O	−1.292632	−2.083087	0.692192
O	−2.764265	−0.561948	0.163874
H	3.539870	−1.155024	1.023326

1 imaginary frequency
ZPE 0.143512
E −552.037497
H −551.883079
G −551.930578

·Tyr-NO2

C	3.275926	−0.901599	−0.038401
C	1.936033	−0.206849	−0.081744
C	1.820536	1.115636	0.449144
C	0.840816	−0.813020	−0.631339
C	0.604268	1.809953	0.409469
C	−0.485657	−0.160534	−0.679425
C	−0.523377	1.230506	−0.131161
O	−1.682435	1.920859	−0.213164
H	4.042682	−0.318043	−0.562916
H	3.230198	−1.893012	−0.498141
H	2.692808	1.589864	0.889176
H	0.914603	−1.809160	−1.055760
H	0.523598	2.820847	0.797240
H	−0.959779	−0.199192	−1.671817
H	−2.411513	1.269074	−0.288164
N	−1.586954	−1.031691	0.131980
O	−1.247709	−2.048746	0.700445
O	−2.739090	−0.579306	0.099074
H	3.618676	−1.025565	0.996432

0 imaginary frequencies
ZPE 0.144610
E −552.037571
H −551.881830
G −551.929586

His

C	−0.071074	2.152833	0.000000
H	−0.608180	2.521994	0.881780
H	−0.608180	2.521994	−0.881780
C	0.000000	0.657440	0.000000
N	−1.145293	−0.118906	0.000000
C	1.112981	−0.148757	0.000000
C	−0.729618	−1.365585	0.000000
N	0.633027	−1.445763	0.000000
H	2.170433	0.070154	0.000000
H	−1.360190	−2.245137	0.000000
H	1.188386	−2.288585	0.000000
H	0.929863	2.596673	0.000000

0 imaginary frequencies
ZPE 0.099036
E −265.629648
H −265.524222
G −265.559350

His ·NO2

N	−3.404970	−0.302331	−0.000972
O	−3.045368	0.848117	−0.000589
O	−2.825878	−1.353186	0.000899
C	3.107107	−1.409042	−0.000533
H	3.753050	−1.322992	−0.882476
H	2.661699	−2.409273	−0.000448
C	2.051206	−0.347707	−0.000088
N	2.378507	0.996477	−0.000242
C	0.684551	−0.495374	0.000498
C	1.232953	1.642062	0.000244
N	0.169516	0.787367	0.000726
H	0.045241	−1.365771	0.000812
H	1.111355	2.717365	0.000274
H	−0.811320	1.034100	0.001093
H	3.753673	−1.323114	0.880963

0 imaginary frequencies
ZPE 0.108822
E −470.775165
H −470.655004
G −470.707299

HAT_His ·NO2

N	−3.145396	−0.368520	−0.034369
O	−2.607161	0.781663	0.216242
O	−2.393416	−1.310756	−0.239972
C	3.085460	−1.210142	0.046806
H	3.028625	−1.898804	−0.807389
H	3.064851	−1.828982	0.952783
C	1.953721	−0.245726	0.027871
N	2.109733	1.078533	−0.140472
C	0.540150	−0.538923	0.158806
C	0.859889	1.571894	−0.116176
N	−0.112649	0.619700	0.050037
H	0.015149	−1.473772	0.300999
H	0.622402	2.623368	−0.214892
H	−1.358007	0.712837	0.129519
H	4.034459	−0.672518	−0.001403

1 imaginary frequency
ZPE 0.102867
E −470.749741
H −470.636673
G −470.683716

His· HONO

N	−3.349797	−0.412518	−0.000113
O	−2.817894	0.836032	−0.000580
O	−2.535733	−1.300382	0.000676
C	3.116310	−1.311864	−0.000222
H	3.025340	−1.962391	−0.880554
H	3.025042	−1.962967	0.879654
C	2.056334	−0.271231	−0.000066
N	2.293706	1.040667	0.000256
C	0.596095	−0.478486	−0.000220
C	1.054699	1.590808	0.000296
N	0.001042	0.699647	0.000022
H	0.035421	−1.406128	−0.000456
H	0.891871	2.662147	0.000541
H	−1.807743	0.760722	0.000062
H	4.103800	−0.846523	0.000095

0 imaginary frequencies
ZPE 0.107210
E −470.760977
H −470.643008
G −470.691630

His·

C	−2.111848	0.000332	0.000033
H	−2.512575	−1.015426	−0.000121
H	−2.493420	0.535392	−0.880226
C	−0.625129	−0.031677	−0.000092
N	0.099325	−1.148002	−0.000025
C	0.304087	1.118222	0.000009
C	1.381210	−0.687930	0.000018
N	1.547215	0.681929	0.000030
H	0.051622	2.174061	−0.000132
H	2.231915	−1.360222	0.000040
H	−2.493244	0.535020	0.880602

0 imaginary frequencies
ZPE 0.084936
E −264.969467
H −264.878204
G −264.914031

HONO

N	−0.162337	−0.526507	−0.000001
O	−1.059364	0.257578	0.000001
O	1.087296	0.072572	0.000001
H	0.912909	1.044343	−0.000007

0 imaginary frequencies
ZPE 0.020331
E −205.771863
H −205.747463
G −205.775508

Trp

C	2.637559	−1.315326	0.000082
H	3.651667	−0.901637	0.000250
H	2.544904	−1.962619	−0.882064
C	1.607872	−0.225825	0.000002
C	1.835837	1.126304	0.000088
C	0.174307	−0.392241	−0.000082
H	2.774165	1.664995	−0.000260
N	0.633384	1.814219	−0.000096
C	−0.405568	0.907074	−0.000115
C	−0.675004	−1.512266	−0.000099
H	0.535746	2.816976	0.000452
C	−1.789946	1.107414	0.000023
C	−2.050884	−1.318657	0.000028
H	−0.260338	−2.517296	−0.000155
H	−2.216631	2.107399	0.000079
C	−2.602457	−0.020928	0.000073
H	−2.715669	−2.178288	0.000060
H	−3.682361	0.100334	0.000189
H	2.544539	−1.962690	0.882123

0 imaginary frequencies
ZPE 0.157714
E −403.277280
H −403.110501
G −403.152120

Trp ·NO2

N	0.611046	−0.111932	−0.275050
H	1.460031	1.852258	−0.250952
H	1.464115	−0.654507	−0.301420
H	−0.408668	3.803648	0.027323
C	−1.548026	0.479198	0.007914
C	−1.113961	−1.948295	−0.135138
C	−2.482347	−2.161320	0.008774
H	−2.864827	−3.178340	0.015544
C	−3.378493	−1.083209	0.145205
H	−4.440708	−1.285131	0.253161
C	−2.924050	0.231589	0.144173
H	−3.624977	1.056177	0.247496
H	−0.423156	−2.780869	−0.239801

0 imaginary frequencies
ZPE 0.166906
E −608.422740
H −608.241853
G −608.300218

Trp·

C	2.654112	−1.265711	−0.000032
H	3.656739	−0.827418	−0.000627
H	2.568088	−1.918078	−0.880169
C	1.597578	−0.215968	0.000029
C	1.784391	1.210728	0.000009
C	0.169535	−0.391191	−0.000003
H	2.749174	1.711076	0.000040
N	0.660045	1.902111	0.000014
C	−0.366523	0.929562	−0.000008
C	−0.680947	−1.498545	0.000019
C	−1.731287	1.146202	−0.000022
C	−2.067500	−1.277303	0.000008
H	−0.288040	−2.512527	0.000042
H	−2.131124	2.155759	−0.000037
C	−2.582479	0.020640	−0.000007
H	−2.746370	−2.125299	0.000020
H	−3.658961	0.168645	−0.000012
H	2.568897	−1.917422	0.880681

0 imaginary frequencies
ZPE 0.144042
E −402.624387
H −402.471431
G −402.514006

HAT_Trp ·NO2

N	3.921997	−0.650446	0.144071
O	2.859870	−1.393558	0.142811
O	3.757321	0.553827	−0.008316
C	−1.132564	3.107824	0.055010
H	−1.730453	3.284021	0.959247
H	−1.777511	3.350494	−0.800657
C	−0.658925	1.696571	−0.007182
C	0.689433	1.257558	−0.129569
C	−0.533767	−0.584220	−0.114902
N	0.766899	−0.066326	−0.231471
H	1.594224	1.852512	−0.151973
H	1.813075	−0.692219	−0.097376
H	−0.295913	3.811906	0.058409
C	−1.449802	0.496504	0.007144
C	−0.946408	−1.903629	−0.116149
C	−2.329524	−2.149766	−0.002432
H	−2.684660	−3.176049	0.001198
C	−3.249123	−1.102026	0.102914
H	−4.308668	−1.325352	0.183319
C	−2.821448	0.232383	0.105379
H	−3.543928	1.040734	0.180045
H	−0.233197	−2.717983	−0.197658

1 imaginary frequency
ZPE 0.161984
E −608.409577
H −608.234706
G −608.288170

Trp· HONO

N	4.202940	−0.539587	−0.000285
O	3.175746	−1.424219	−0.000340
O	3.865236	0.617338	0.000194
C	−1.160844	3.097449	−0.000139
H	−1.778111	3.323087	0.880304
H	−1.777877	3.322953	−0.880780
C	−0.723504	1.674307	0.000026
C	0.630866	1.184793	0.000235
C	−0.626201	−0.600692	0.000188
N	0.706596	−0.131428	0.000347
H	1.532778	1.787744	0.000292
H	2.294706	−0.912083	0.000059
H	−0.306801	3.780685	−0.000078
C	−1.538915	0.492656	−0.000006
C	−1.067497	−1.909106	0.000205
C	−2.461041	−2.133742	0.000026
H	−2.832457	−3.154558	0.000034
C	−3.368512	−1.071895	−0.000161
H	−4.434958	−1.276700	−0.000296
C	−2.917325	0.256252	−0.000179
H	−3.629192	1.077789	−0.000324
H	−0.364854	−2.736898	0.000350

0 imaginary frequencies
ZPE 0.166237
E −608.418428
H −608.238735
G −608.294378

Coordinates relating to Figure S9

Ia

```
C     -3.378831     0.836683     2.504919
S     -3.356720     0.051841     0.847740
H     -2.768747     0.280829     3.224928
H     -4.413369     0.848271     2.867883
C      7.444398     1.221118    -1.014307
C      6.145646     0.459785    -0.870508
C      6.130937    -0.921888    -0.638941
C      4.906697     1.112935    -0.936664
C      4.938686    -1.623001    -0.474139
C      3.702269     0.432618    -0.774951
C      3.703980    -0.955080    -0.538001
O      2.579460    -1.677369    -0.377285
H      8.257075     0.567165    -1.353602
H      7.770625     1.671459    -0.064725
H      7.075033    -1.463930    -0.587744
H      4.880226     2.186411    -1.121719
H      4.938347    -2.695489    -0.299212
H      2.749943     0.951820    -0.833186
H      1.768073    -1.091007    -0.446242
C     -1.640926     3.090655    -1.357757
C     -2.279934    -1.481545    -2.803356
C     -0.731610    -3.038514     1.507886
C      0.177203     1.523577     2.845877
C     -1.973258     1.977342    -2.118748
C     -2.505720     2.038708    -3.463018
C     -2.670507     0.753359    -3.876645
C     -2.244962    -0.092458    -2.782684
C     -1.915441    -2.307370    -1.746892
C     -2.004454    -3.751223    -1.768772
C     -1.584445    -4.187095    -0.550873
C     -1.232809    -3.009889     0.213168
C     -0.352779    -1.926887     2.250680
C      0.215778    -1.989651     3.578877
C      0.487901    -0.709166     3.949690
C      0.078681     0.137341     2.850836
C     -0.251171     2.353155     1.816095
C     -0.178143     3.797090     1.842699
C     -0.705843     4.236469     0.667419
C     -1.094022     3.060745    -0.080127
N     -1.830407     0.674912    -1.731868
N     -1.446999    -1.886261    -0.534764
N     -0.432833    -0.626310     1.837780
N     -0.816288     1.934924     0.643039
Fe    -1.057801     0.021365     0.026786
H     -2.639584    -1.959477    -3.710289
H     -0.605805    -4.012733     1.971648
H      0.607140     1.997310     3.723944
H     -1.806883     4.066964    -1.804826
H      0.225542     4.379192     2.662591
H      0.924481    -0.350861     4.874280
H      0.385110    -2.904785     4.133405
H     -1.507053    -5.204073    -0.185578
H     -2.348874    -4.334493    -2.614418
H     -3.046812     0.390792    -4.825850
O      0.486557    -0.039635    -0.598825
H      7.354447     2.040089    -1.738955
H     -3.015372     1.869293     2.469583
H     -0.824184     5.255442     0.318498
H     -2.715383     2.955162    -4.001674
0 imaginary frequencies
ZPE 0.449467
E   -1972.606347
H   -1972.124115
G   -1972.225693
```

TSa

```
C     -2.463740     0.672190     3.177307
S     -2.954158    -0.094902     1.590425
H     -1.603463     0.153894     3.612832
H     -3.306381     0.602171     3.873829
C      7.316607     1.105445    -1.639959
C      5.984868     0.437605    -1.391164
C      5.830582    -0.544532    -0.397959
C      4.837777     0.799902    -2.122096
C      4.601385    -1.132541    -0.134172
C      3.602740     0.214877    -1.890158
C      3.427301    -0.787320    -0.878873
O      2.299427    -1.373313    -0.661248
H      7.406006     2.072824    -1.120468
H      7.477214     1.307539    -2.707361
H      6.702445    -0.847454     0.183908
H      4.926545     1.560823    -2.898722
H      4.496057    -1.890307     0.638389
H      2.728056     0.510421    -2.463653
H      0.995361    -0.545977    -0.899898
C      1.017437     1.463445     2.356935
C     -2.198840     3.101315    -0.869186
C     -3.074825    -1.435192    -2.292421
C     -0.214519    -3.081980     1.247590
C      0.221589     2.310230     1.594249
C      0.257677     3.752509     1.663306
C     -0.667520     4.209824     0.774803
C     -1.259399     3.047263     0.153558
C     -2.704049     2.004336    -1.555179
C     -3.631989     2.090066    -2.662213
C     -3.860623     0.815199    -3.078403
C     -3.079932    -0.047562    -2.218365
C     -2.382523    -2.280812    -1.434592
C     -2.433660    -3.723886    -1.495895
C     -1.648439    -4.188015    -0.485955
C     -1.108394    -3.028911     0.186464
C      0.380271    -1.981162     1.847374
C      1.377847    -2.063834     2.889293
C      1.746465    -0.788229     3.181387
C      0.963203     0.076362     2.327887
N     -0.716115     1.908382     0.682612
N     -2.387900     0.698657    -1.307947
N     -1.576086    -1.886789    -0.402388
N      0.137886    -0.672225     1.531596
Fe    -1.095819     0.008846     0.105078
H     -2.536967     4.085249    -1.182302
H     -3.679938    -1.897528    -3.067242
H      0.080538    -4.064627     1.603628
H      1.725401     1.922320     3.040922
H      1.747675    -2.989091     3.313978
H     -1.431522    -5.213920    -0.214073
H     -3.002045    -4.288946    -2.224951
H     -4.497315     0.470278    -3.884445
H     -4.038185     3.013899    -3.056613
H     -0.926305     5.234486     0.535968
O      0.191098     0.069516    -1.072729
H      8.149531     0.480978    -1.293230
H      2.479626    -0.442740     3.899988
H      0.914763     4.322331     2.309262
H     -2.201773     1.726465     3.044742
1 imaginary frequency
ZPE 0.444854
E   -1972.592681
H   -1972.115412
G   -1972.216109
```

Ib

C	−1.933237	−0.152819	3.469898
S	−2.680788	0.407975	1.910898
H	−1.671583	−1.213011	3.423141
H	−2.642035	0.026662	4.283064
C	7.217483	1.218468	−1.212443
C	5.899009	0.478660	−1.229751
C	5.850239	−0.920440	−1.135409
C	4.679576	1.159410	−1.321824
C	4.642093	−1.610483	−1.126787
C	3.456946	0.487105	−1.315330
C	3.427025	−0.912702	−1.215350
O	2.277970	−1.629471	−1.209176
H	7.972213	0.715439	−1.829247
H	7.633611	1.293536	−0.197706
H	6.780271	−1.483091	−1.070303
H	4.681025	2.245168	−1.404312
H	4.615464	−2.694346	−1.058507
H	2.521160	1.033302	−1.394603
H	1.488383	−1.024560	−1.243255
C	−1.314862	3.416526	−0.287390
C	−3.854571	−0.346076	−1.960424
C	−0.844143	−3.344389	0.358632
C	1.422609	0.427938	2.352751
C	−2.239944	2.647114	−0.987023
C	−3.272560	3.189315	−1.836479
C	−4.009993	2.135493	−2.281585
C	−3.417531	0.947992	−1.718255
C	−3.245261	−1.503869	−1.480921
C	−3.665259	−2.843880	−1.803385
C	−2.800458	−3.684881	−1.169066
C	−1.864322	−2.853896	−0.453640
C	0.039074	−2.579780	1.106827
C	1.074062	−3.122354	1.955175
C	1.708215	−2.062003	2.523680
C	1.060522	−0.875955	2.016297
C	0.844924	1.578866	1.837449
C	1.283269	2.921342	2.133442
C	0.529185	3.758501	1.371960
C	−0.382435	2.933779	0.616309
N	−2.336793	1.280854	−0.932498
N	−2.148656	−1.530885	−0.661855
N	0.045369	−1.209201	1.166962
N	−0.186752	1.605730	0.926062
Fe	−1.113215	0.035096	0.072399
H	−4.722896	−0.468371	−2.600822
H	−0.736884	−4.423333	0.417108
H	2.249902	0.552464	3.044664
H	−1.353093	4.491690	−0.434095
H	2.079912	3.170547	2.822683
H	2.544565	−2.063612	3.210687
H	1.280135	−4.177713	2.079169
H	−2.788155	−4.767359	−1.172351
H	−4.506852	−3.093113	−2.437003
H	−4.873846	2.144221	−2.933980
O	0.104849	−0.032664	−1.280808
H	7.106329	2.240081	−1.592458
H	0.574755	4.838469	1.311940
H	−3.409410	4.242972	−2.043978
H	−1.019391	0.419677	3.669035
H	−0.380042	−0.096132	−2.120582

0 imaginary frequencies
ZPE 0.461625
E −1973.152045
H −1972.657128
G −1972.758684

TSb

C	−1.382302	0.496031	3.483509
S	−2.389259	0.006445	2.040628
H	−0.512112	−0.157377	3.599889
H	−2.011225	0.403099	4.374053
C	6.961845	0.977420	−1.129880
C	5.594444	0.345498	−1.189508
C	5.438833	−1.052249	−1.067548
C	4.437890	1.119813	−1.385893
C	4.195739	−1.651151	−1.145282
C	3.178403	0.545190	−1.470924
C	3.025633	−0.868356	−1.366345
O	1.868720	−1.470015	−1.475699
H	7.518994	0.816982	−2.063818
H	7.569084	0.550970	−0.322154
H	6.320427	−1.670230	−0.908056
H	4.533172	2.200218	−1.472162
H	4.076002	−2.726158	−1.052625
H	2.291495	1.152114	−1.621283
H	0.955921	−0.746727	−1.499996
C	−1.124118	3.442646	−0.036349
C	−4.005971	−0.078723	−1.679839
C	−1.043656	−3.352649	0.297050
C	1.855514	0.167353	1.908331
C	−2.164693	2.766615	−0.668567
C	−3.248586	3.416655	−1.365588
C	−4.061668	2.427608	−1.830755
C	−3.469381	1.180079	−1.414288
C	−3.451099	−1.286204	−1.281876
C	−4.016449	−2.583943	−1.563253
C	−3.177706	−3.502686	−1.012599
C	−2.101710	−2.765329	−0.392171
C	−0.011485	−2.677906	0.928714
C	1.068596	−3.320848	1.638435
C	1.884580	−2.334293	2.095303
C	1.305022	−1.083625	1.666519
C	1.294690	1.382601	1.512795
C	1.869853	2.678532	1.784957
C	1.022494	3.598510	1.247095
C	−0.062547	2.859494	0.643657
N	−2.315816	1.408233	−0.717132
N	−2.281168	−1.420531	−0.569943
N	0.146475	−1.311254	0.964330
N	0.122555	1.513438	0.826693
Fe	−1.079349	0.047209	0.137675
H	−4.935063	−0.116609	−2.240700
H	−1.031182	−4.437083	0.351054
H	2.791590	0.204177	2.456861
H	−1.139904	4.527508	−0.087878
H	2.798438	2.844036	2.316290
H	2.800692	−2.425329	2.664826
H	1.174349	−4.391790	1.756687
H	−3.265386	−4.581767	−1.013191
H	−4.935652	−2.751234	−2.110251
H	−4.977930	2.518293	−2.400264
O	−0.031090	0.033019	−1.486073
H	6.897999	2.058690	−0.970569
H	1.111215	4.677640	1.245003
H	−3.359480	4.488139	−1.474346
H	−1.037649	1.529813	3.393668
H	−0.620920	−0.207656	−2.218301

1 imaginary frequency
ZPE 0.456861
E −1973.152327
H −1972.662917
G −1972.760484

Ic

C	−0.227926	1.343538	−3.252904
S	0.592188	−0.082669	−2.481348
H	−1.303613	1.306233	−3.042694
H	−0.082061	1.293555	−4.335562
H	0.395162	−0.805405	2.076747
C	1.790802	2.995569	0.570290
C	3.289524	−1.598702	0.273059
C	−1.262856	−2.983596	−0.592900
C	−2.680044	1.639951	−0.668087
C	2.593613	1.858323	0.582464
C	4.010728	1.868198	0.852338
C	4.438336	0.579371	0.747127
C	3.278806	−0.214648	0.423968
C	2.172776	−2.389770	0.038030
C	2.184803	−3.829355	−0.056119
C	0.897665	−4.216697	−0.270831
C	0.103036	−3.012434	−0.322779
C	−2.034399	−1.843209	−0.747753
C	−3.443163	−1.843636	−1.061221
C	−3.840176	−0.543355	−1.103498
C	−2.677096	0.257762	−0.804224
C	−1.584892	2.416901	−0.298135
C	−1.623862	3.849921	−0.115940
C	−0.367487	4.230495	0.240564
C	0.435855	3.029328	0.270191
N	2.168835	0.582551	0.323252
N	0.894221	−1.911956	−0.120882
N	−1.581277	−0.548096	−0.616165
N	−0.325645	1.939583	−0.064151
Fe	0.281173	0.020439	−0.047758
H	4.245731	−2.102837	0.376778
H	−1.761169	−3.939562	−0.723049
H	−3.623867	2.153231	−0.826043
H	2.270920	3.941065	0.804924
H	−2.505310	4.465442	−0.243609
H	−4.828672	−0.146603	−1.297148
H	−4.037987	−2.734490	−1.218236
H	0.507818	−5.218701	−0.397736
H	3.069158	−4.447298	0.034118
H	5.438443	0.186905	0.880601
O	−0.158249	−0.105504	1.688684
O	−2.131579	−1.491279	3.527236
N	−2.322487	−0.340784	3.228691
O	−3.195481	0.217431	2.619575
H	−0.001512	5.224406	0.465122
H	4.588415	2.753292	1.086974
H	0.165746	2.284081	−2.858921

0 imaginary frequencies
ZPE 0.337761
E −1831.363460
H −1830.996643
G −1831.087657

TSc

C	−0.603845	−0.897994	−3.324402
S	0.772346	−0.109825	−2.422329
H	−0.767080	−1.919994	−2.967010
H	−0.307643	−0.948693	−4.377627
H	0.584292	−0.502417	2.205362
C	−2.703667	1.858938	−0.593746
C	1.958432	2.956419	0.072313
C	3.079658	−1.742413	0.327131
C	−1.593143	−2.838903	−0.306828
C	−1.522075	2.570555	−0.430860
C	−1.427673	4.010308	−0.449507
C	−0.115621	4.316310	−0.262327
C	0.588115	3.062390	−0.128825
C	2.670233	1.771086	0.204862
C	4.096614	1.695383	0.412485
C	4.413759	0.374144	0.483247
C	3.181763	−0.357323	0.316627
C	1.900500	−2.455884	0.166714
C	1.803156	−3.893360	0.170475
C	0.488755	−4.199955	−0.007241
C	−0.221139	−2.949620	−0.118776
C	−2.303593	−1.650115	−0.435082
C	−3.731622	−1.574807	−0.634037
C	−4.045068	−0.253786	−0.722958
C	−2.809139	0.475624	−0.575996
N	−0.286605	2.013018	−0.232847
N	2.129800	0.511755	0.152913
N	0.654983	−1.895526	−0.009707
N	−1.758615	−0.392562	−0.406594
Fe	0.176467	0.068243	−0.112738
H	2.523210	3.882064	0.130100
H	3.994122	−2.311102	0.465054
H	−2.160899	−3.763138	−0.359285
H	−3.616266	2.429428	−0.736746
H	−4.392246	−2.430615	−0.692368
H	0.025957	−5.177368	−0.058931
H	2.641612	−4.566929	0.294190
H	5.385299	−0.080181	0.630491
H	4.754188	2.551948	0.490077
H	0.350193	5.292508	−0.215628
O	−0.046134	0.111187	1.786101
O	−1.358519	−0.909225	3.875707
N	−1.487767	0.024904	3.128468
O	−2.412150	0.733292	2.858846
H	−5.016502	0.201454	−0.867896
H	−2.265312	4.681938	−0.588052
H	−1.533152	−0.331159	−3.233745

1 imaginary frequency
ZPE 0.337832
E −1831.354198
H −1830.988272
G −1831.075254

References

[1] Y. Zhao, E. Marschall, M. Treisman, A. McKay, L. Padva, M. Crüsemann, D. R. Nelson, D. L. Steer, R. B. Schittenhelm, J. Tailhades, M. J. Cryle, "Cytochrome P450Blt Enables Versatile Peptide Cyclisation to Generate Histidine- and Tyrosine-Containing Crosslinked Tripeptide Building Blocks" *Angew. Chem. Int. Ed.* **2022**, *61*, e202204957.

[2] L. Padva, L. Zimmer, J. Gullick, Y. Zhao, V. M. Sasi, R. B. Schittenhelm, C. J. Jackson, M. J. Cryle, M. Crüsemann, "Ribosomal pentapeptide nitration for non-ribosomal peptide antibiotic precursor biosynthesis" *Chem* **2025**, *11*.

[3] S. G. Bell, F. Xu, E. O. D. Johnson, I. M. Forward, M. Bartlam, Z. Rao, L.-L. Wong, "Protein recognition in ferredoxin–P450 electron transfer in the class I CYP199A2 system from Rhodopseudomonas palustris" *J. Biol. Inorg. Chem.* **2010**, *15*, 315-328.

[4] P. R. Evans, G. N. Murshudov, "How good are my data and what is the resolution?" *Acta Crystallogr. Sect. D. Biol. Crystallogr.* **2013**, *69*, 1204-1214.

[5] A. J. McCoy, R. W. Grosse-Kunstleve, P. D. Adams, M. D. Winn, L. C. Storoni, R. J. Read, "Phaser crystallographic software" *J. Appl. Crystallogr.* **2007**, *40*, 658-674.

[6] J. Abramson, J. Adler, J. Dunger, R. Evans, T. Green, A. Pritzel, O. Ronneberger, L. Willmore, A. J. Ballard, J. Bambrick, S. W. Bodenstein, D. A. Evans, C.-C. Hung, M. O'Neill, D. Reiman, K. Tunyasuvunakool, Z. Wu, A. Žemgulytė, E. Arvaniti, C. Beattie, O. Bertolli, A. Bridgland, A. Cherepanov, M. Congreve, A. I. Cowen-Rivers, A. Cowie, M. Figurnov, F. B. Fuchs, H. Gladman, R. Jain, Y. A. Khan, C. M. R. Low, K. Perlin, A. Potapenko, P. Savy, S. Singh, A. Stecula, A. Thillaisundaram, C. Tong, S. Yakneen, E. D. Zhong, M. Zielinski, A. Žídek, V. Bapst, P. Kohli, M. Jaderberg, D. Hassabis, J. M. Jumper, "Accurate structure prediction of biomolecular interactions with AlphaFold 3" *Nature* **2024**, *630*, 493-500.

[7] P. V. Afonine, R. W. Grosse-Kunstleve, N. Echols, J. J. Headd, N. W. Moriarty, M. Mustyakimov, T. C. Terwilliger, A. Urzhumtsev, P. H. Zwart, P. D. Adams, "Towards automated crystallographic structure refinement with phenix.refine" *Acta Crystallogr. Sect. D. Biol. Crystallogr.* **2012**, *68*, 352-367.

[8] P. Emsley, K. Cowtan, "Coot: model-building tools for molecular graphics" *Acta Crystallogr. Sect. D. Biol. Crystallogr.* **2004**, *60*, 2126-2132.

[9] H. Tomita, Y. Katsuyama, H. Minami, Y. Ohnishi, "Identification and characterization of a bacterial cytochrome P450 monooxygenase catalyzing the 3-nitration of tyrosine in rufomycin biosynthesis" *J. Biol. Chem.* **2017**, *292*, 15859-15869.

[10] Gaussian 16, Revision C.01, M. J. Frisch, G. W. Trucks, H. B. Schlegel, G. E. Scuseria, M. A. Robb, J. R. Cheeseman, G. Scalmani, V. Barone, G. A. Petersson, H. Nakatsuji, X. Li, M. Caricato, A. V. Marenich, J. Bloino, B. G. Janesko, R. Gomperts, B. Mennucci, H. P. Hratchian, J. V. Ortiz, A. F. Izmaylov, J. L. Sonnenberg, D. Williams-Young, F. Ding, F. Lipparini, F. Egidi, J. Goings, B. Peng, A. Petrone, T. Henderson, D. Ranasinghe, V. G. Zakrzewski, J. Gao, N. Rega, G. Zheng, W. Liang, M. E. M. Hada, K. Toyota, R. Fukuda, J. Hasegawa, M. Ishida, T. Nakajima, Y. Honda, O. Kitao, H. Nakai, T. Vreven, K. Throssell, J. J. A. Montgomery, J. E. Peralta, F. Ogliaro, M. J. Bearpark, J. J. Heyd, E. N. Brothers, K. N. Kudin, V. N. Staroverov, T. A. Keith, R. Kobayashi, J. Normand, K. Raghavachari, A. P. Rendell, J. C. Burant, S. S. Iyengar, J. Tomasi, M. Cossi, J. M. Millam, M. Klene, C. Adamo, R. Cammi, J. W. Ochterski, R. L. Martin, K. Morokuma, O. Farkas, J. B. Foresman, D. J. Fox, Gaussian, Inc., Wallingford CT, **2016**.

[11] C. Lee, W. Yang, R. G. Parr, "Development of the Colle-Salvetti correlation-energy formula into a functional of the electron density" *Phys. Rev. B* **1988**, *37*, 785-789.

[12] A. D. Becke, "Density-functional thermochemistry. III. The role of exact exchange" *J. Chem. Phys.* **1993**, *98*, 5648-5652.

[13] P. J. Hay, W. R. Wadt, "Ab initio effective core potentials for molecular calculations. Potentials for the transition metal atoms Sc to Hg" *J. Chem. Phys.* **1985**, *82*, 270-283.

[14] W. J. Hehre, R. Ditchfield, J. A. Pople, "Self—Consistent Molecular Orbital Methods. XII. Further Extensions of Gaussian—Type Basis Sets for Use in Molecular Orbital Studies of Organic Molecules" *J. Chem. Phys.* **1972**, *56*, 2257-2261.

[15] P. C. Hariharan, J. A. Pople, "The influence of polarization functions on molecular orbital hydrogenation energies" *Theor. Chim. Acta* **1973**, *28*, 213-222.

[16] S. Grimme, S. Ehrlich, L. Goerigk, "Effect of the damping function in dispersion corrected density functional theory" *J. Comput. Chem.* **2011**, *32*, 1456-1465.

[17] A. D. McLean, G. S. Chandler, "Contracted Gaussian basis sets for molecular calculations. I. Second row atoms, Z=11–18" *J. Chem. Phys.* **1980**, *72*, 5639-5648.

[18] R. Krishnan, J. S. Binkley, R. Seeger, J. A. Pople, "Self-consistent molecular orbital methods. XX. A basis set for correlated wave functions" *J. Chem. Phys.* **1980**, *72*, 650-654.

[19] T. Clark, J. Chandrasekhar, G. W. Spitznagel, P. V. R. Schleyer, "Efficient diffuse function-augmented basis sets for anion calculations. III. The 3-21+G basis set for first-row elements, Li–F" *J. Comput. Chem.* **1983**, *4*, 294-301.

[20] M. J. Frisch, J. A. Pople, J. S. Binkley, “Self-consistent molecular orbital methods 25. Supplementary functions for Gaussian basis sets” *J. Chem. Phys.* **1984**, *80*, 3265-3269.

[21] M. H. Hansen, A. Keto, M. Treisman, V. M. Sasi, L. Coe, Y. Zhao, L. Padva, C. Hess, V. Leichthammer, D. L. Machell, R. B. Schittenhelm, C. J. Jackson, J. Tailhades, M. Crüsemann, J. J. De Voss, E. H. Krenske, M. J. Cryle, “Structural Insights into a Side Chain Cross-Linking Biarylitide P450 from RiPP Biosynthesis” *ACS Catal.* **2024**, *14*, 812-826.

[22] R. Anandakrishnan, B. Aguilar, A. V. Onufriev, “H++ 3.0: automating pK prediction and the preparation of biomolecular structures for atomistic molecular modeling and simulations” *Nucleic Acids Res.* **2012**, *40*, W537-W541.

[23] J. C. Gordon, J. B. Myers, T. Folta, V. Shoja, L. S. Heath, A. Onufriev, “H++: a server for estimating p Ka s and adding missing hydrogens to macromolecules” *Nucleic Acids Res.* **2005**, *33*, W368-W371.

[24] J. Myers, G. Grothaus, S. Narayanan, A. Onufriev, “A simple clustering algorithm can be accurate enough for use in calculations of pKs in macromolecules” *Proteins:Struct., Funct., Bioinf.* **2006**, *63*, 928-938.

[25] K. Shahrokh, A. Orendt, G. S. Yost, T. E. Cheatham Iii, “Quantum mechanically derived AMBER-compatible heme parameters for various states of the cytochrome P450 catalytic cycle” *J. Comput. Chem.* **2012**, *33*, 119-133.

[26] P. Li, K. M. Merz, Jr., “MCPB.py: A Python Based Metal Center Parameter Builder” *J. Chem. Inf. Model.* **2016**, *56*, 599-604.

[27] U. C. Singh, P. A. Kollman, “An approach to computing electrostatic charges for molecules” *J. Comput. Chem.* **1984**, *5*, 129-145.

[28] B. H. Besler, K. M. Merz Jr, P. A. Kollman, “Atomic charges derived from semiempirical methods” *J. Comput. Chem.* **1990**, *11*, 431-439.

[29] A. V. Marenich, C. J. Cramer, D. G. Truhlar, “Universal Solvation Model Based on Solute Electron Density and on a Continuum Model of the Solvent Defined by the Bulk Dielectric Constant and Atomic Surface Tensions” *J. Phys. Chem. B* **2009**, *113*, 6378-6396.

[30] J. M. Seminario, “Calculation of intramolecular force fields from second-derivative tensors” *Int. J. Quantum Chem* **1996**, *60*, 1271-1277.

[31] J. A. Maier, C. Martinez, K. Kasavajhala, L. Wickstrom, K. E. Hauser, C. Simmerling, “ff14SB: Improving the Accuracy of Protein Side Chain and Backbone Parameters from ff99SB” *J. Chem. Theory Comput.* **2015**, *11*, 3696-3713.

[32] W. L. Jorgensen, J. Chandrasekhar, J. D. Madura, R. W. Impey, M. L. Klein, “Comparison of simple potential functions for simulating liquid water” *J. Chem. Phys.* **1983**, *79*, 926-935.

[33] Amber 2024, D. A. Case, H. M. Aktulga, K. Belfon, I. Y. Ben-Shalom, J. T. Berryman, S. R. Brozell, D. S. Cerutti, T. E. C. III, G. A. Cisneros, V. W. D. Cruzeiro, T. A. Darden, N. Forouzesh, M. Ghazimirsaeed, G. Gi-amba¸su, T. Giese, M. K. Gilson, H. Gohlke, A. W. Goetz, J. Harris, Z. Huang, S. Izadi, S. A. Izmailov, K. Kasavajhala, M. C. Kaymak, I. Kolossváry, A. Kovalenko, T. Kurtzman, T. S. Lee, P. Li, Z. Li, C. Lin, J. Liu, T. Luchko, R. Luo, M. Machado, M. Manathunga, K. M. Merz, Y. Miao, O. Mikhailovskii, G. Monard, H. Nguyen, K. A. O’Hearn, A. Onufriev, F. Pan, S. Pantano, A. Rahnamoun, D. R. Roe, A. Roitberg, C. Sagui, S. Schott-Verdugo, A. Shajan, J. Shen, C. L. Simmerling, N. R. Skrynnikov, J. Smith, J. Swails, R. C. Walker, J. Wang, J. Wang, X. Wu, Y. Wu, Y. Xiong, Y. Xue, D. M. York, C. Zhao, Q. Zhu, P. A. Kollman, University of California, San Francisco, **2024**.

[34] R. Salomon-Ferrer, A. W. Götz, D. Poole, S. Le Grand, R. C. Walker, “Routine Microsecond Molecular Dynamics Simulations with AMBER on GPUs. 2. Explicit Solvent Particle Mesh Ewald” *J. Chem. Theory Comput.* **2013**, *9*, 3878-3888.

[35] T. Darden, D. York, L. Pedersen, “Particle mesh Ewald: An N·log(N) method for Ewald sums in large systems” *J. Chem. Phys.* **1993**, *98*, 10089-10092.

[36] S. Miyamoto, P. A. Kollman, “Settle: An analytical version of the SHAKE and RATTLE algorithm for rigid water models” *J. Comput. Chem.* **1992**, *13*, 952-962.

[37] D. R. Roe, T. E. Cheatham, III, “PTRAJ and CPPTRAJ: Software for Processing and Analysis of Molecular Dynamics Trajectory Data” *J. Chem. Theory Comput.* **2013**, *9*, 3084-3095.

[38] H. S. Fernandes, M. J. Ramos, N. M. F. S. A. Cerqueira, “molUP: A VMD plugin to handle QM and ONIOM calculations using the gaussian software” *J. Comput. Chem.* **2018**, *39*, 1344-1353.

[39] W. Humphrey, A. Dalke, K. Schulten, “VMD: Visual molecular dynamics” *J. Mol. Graph.* **1996**, *14*, 33-38.

[40] T. Vreven, K. S. Byun, I. Komáromi, S. Dapprich, J. A. Montgomery, Jr., K. Morokuma, M. J. Frisch, “Combining Quantum Mechanics Methods with Molecular Mechanics Methods in ONIOM” *J. Chem. Theory Comput.* **2006**, *2*, 815-826.

[41] S. Dapprich, I. Komáromi, K. S. Byun, K. Morokuma, M. J. Frisch, “A new ONIOM implementation in Gaussian98. Part I. The calculation of energies, gradients, vibrational frequencies and electric field derivatives” *J. Mol. Struct. THEOCHEM* **1999**, *461-462*, 1-21.

[42] M. Dolg, U. Wedig, H. Stoll, H. Preuss, “Energy-adjusted ab initio pseudopotentials for the first row transition elements” *J. Chem. Phys.* **1987**, *86*, 866-872.

[43] S. Gergel, J. Soler, A. Klein, K. H. Schülke, B. Hauer, M. Garcia-Borràs, S. C. Hammer, "Engineered cytochrome P450 for direct arylalkene-to-ketone oxidation via highly reactive carbocation intermediates" *Nat. Catal.* **2023**, *6*, 606-617.
[44] F. Weigend, R. Ahlrichs, "Balanced basis sets of split valence, triple zeta valence and quadruple zeta valence quality for H to Rn: Design and assessment of accuracy" *Phys. Chem. Chem. Phys.* **2005**, *7*, 3297-3305.
[45] E. F. Pettersen, T. D. Goddard, C. C. Huang, E. C. Meng, G. S. Couch, T. I. Croll, J. H. Morris, T. E. Ferrin, "UCSF ChimeraX: Structure visualization for researchers, educators, and developers" *Protein Sci.* **2021**, *30*, 70-82.
[46] E. C. Meng, T. D. Goddard, E. F. Pettersen, G. S. Couch, Z. J. Pearson, J. H. Morris, T. E. Ferrin, "UCSF ChimeraX: Tools for structure building and analysis" *Protein Sci.* **2023**, *32*, e4792.
[47] V. M. Ingman, A. J. Schaefer, L. R. Andreola, S. E. Wheeler, "QChASM: Quantum chemistry automation and structure manipulation" *Wiley Interdicip. Rev. Comput. Mol. Sci.* **2021**, *11*, e1510.
[48] A. J. Schaefer, V. M. Ingman, S. E. Wheeler, "SEQCROW: A ChimeraX bundle to facilitate quantum chemical applications to complex molecular systems" *J. Comput. Chem.* **2021**, *42*, 1750-1754.
[49] M. Álvarez-Moreno, C. de Graaf, N. López, F. Maseras, J. M. Poblet, C. Bo, "Managing the Computational Chemistry Big Data Problem: The ioChem-BDPlatform" *J. Chem. Inf. Model.* **2015**, *55*, 95–103.
[50] C. Bo, F. Maseras, N. López, "The Role of Computational Results Databases in Accelerating the Discovery of Catalysts" *Nat. Catal.* **2018**, *1*, 809–810.
